# Establishing the selective phospholipid membrane coordination, permeation and lysis properties for a series of ‘druggable’ supramolecular self-associating antimicrobial amphiphiles†

**DOI:** 10.1039/d2sc02630a

**Published:** 2022-08-10

**Authors:** Jessica E. Boles, Charlotte Bennett, Jennifer Baker, Kira L. F. Hilton, Hiral A. Kotak, Ewan R. Clark, Yifan Long, Lisa J. White, Hin Yuk Lai, Charlotte K. Hind, J. Mark Sutton, Michelle D. Garrett, Anne Cheasty, Jose L. Ortega-Roldan, Mark Charles, Cally J. E. Haynes, Jennifer R. Hiscock

**Affiliations:** School of Chemistry and Forensics, University of Kent Canterbury CT2 7NH UK J.R.Hiscock@Kent.ac.uk; School of Biosciences, University of Kent Canterbury CT2 7NJ UK; Cancer Research Horizons 2 Redman Place London E20 1JQ UK Mark.Charles@cancer.org.uk; Chemistry Department, UCL 20 Gordon Street London WC1H 0AJ UK cally.haynes@ucl.ac.uk; Research and Evaluation Porton Down, UKHSA, Porton Down Salisbury SP4 0JG UK; Exscientia The Schrödinger Building, Heatley Road, Oxford Science Park Oxford OX4 4GE UK

## Abstract

The rise of antimicrobial resistance remains one of the greatest global health threats facing humanity. Furthermore, the development of novel antibiotics has all but ground to a halt due to a collision of intersectional pressures. Herein we determine the antimicrobial efficacy for 14 structurally related supramolecular self-associating amphiphiles against clinically relevant Gram-positive methicillin resistant *Staphylococcus aureus* and Gram-negative *Escherichia coli*. We establish the ability of these agents to selectively target phospholipid membranes of differing compositions, through a combination of computational host:guest complex formation simulations, synthetic vesicle lysis, adhesion and membrane fluidity experiments, alongside our novel ^1^H NMR CPMG nanodisc coordination assays, to verify a potential mode of action for this class of compounds and enable the production of evermore effective next-generation antimicrobial agents. Finally, we select a 7-compound subset, showing two lead compounds to exhibit ‘druggable’ profiles through completion of a variety of *in vivo* and *in vitro* DMPK studies.

## Abstract 2

A combination of computational and synthetic phospholipid vesicle/nanodisc assays are used to investigate the mode of action for a class of antimicrobial agents, while a range of DMPK studies establish agent druggability.

## Graphical abstract

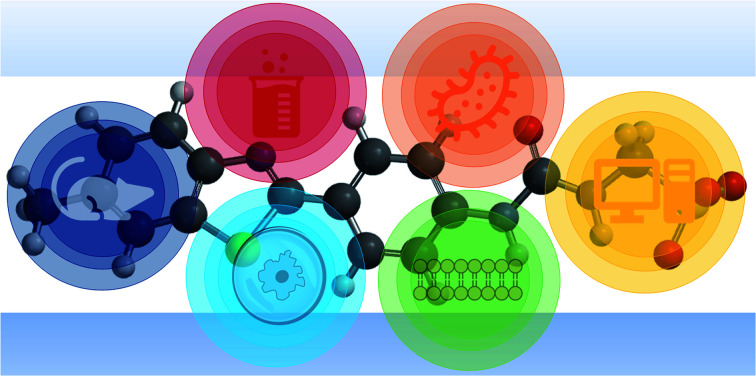

## Introduction

Antimicrobial resistance (AMR), termed by some as the ‘silent pandemic’,^1^ remains one of the greatest global health threats within today's society. In 2019 AMR was found to be indirectly and directly responsible for 4.95 million and 1.27 million deaths worldwide respectively. This means that AMR is directly responsible for the same, if not a greater, number of global deaths per year than those directly attributed to either malaria or HIV/AIDs over that same time period.^2^

This rise in AMR has traditionally been attributed to the ongoing use and misuse of antimicrobial/antibiotic/antiseptic agents across the clinical^3,4^ and agricultural/veterinary sectors,^5^ alongside the decisions of the individual to abide by a treatment regime as advised to them by an appropriately qualified clinician.^6^ However, the recent COVID-19 pandemic, although restricting the movement of individuals to limit the spread of infection,^7^ has also resulted in the increased use of antimicrobial agents, reported to have further driven the rise of AMR.^1,7,8^ In addition, AMR strains have now been identified that are resistant to all antimicrobial agents currently marketed,^9^ including commonly used antiseptics^10^ and antibiotics of last resort, such as colistin.^11^ Furthermore, poor market returns, combined with high developmental costs,^9,12,13^ have halted the identification of any new classes of antimicrobials approved for clinical use.^9^ However, raising the awareness of this unmet need has spurred a wide range of novel approaches to the design of next generation antimicrobial strategies. This includes work by Koksch and co-workers, who used a 3,5-diaminobenzoic acid scaffold appended with ultrashort amino acid sequences to produce novel antimicrobial agents, demonstrating efficacy similar to that of commercialised antibiotics against *Staphylococcus aureus* (*S. aureus*) and *Micrococcus Luteus.*^14^ Likewise, DeGrado and co-workers have produced acrylamide foldamers that are able to overcome the limitations commonly associated with antimicrobial peptides.^15,16^ Finally, Muthuvijayan and co-workers have developed a series of poly(aryl ether) based supramolecular amphiphilic dendrimers which have been shown both to act as hydrogels and to demonstrate broad spectrum activity against *Escherichia coli* (*E. coli*) and *S. aureus*.^17^

In addition, supramolecular chemistry has offered several non-traditional physicochemical strategies to produce novel antimicrobial agents.^18–20^ These innovations include the effective targeting of the bacterial phospholipid membrane itself, as the phospholipids present within bacterial membranes differ significantly not only from eukaryotic cell membranes but from each other.^21^ Specific small molecule innovation in this area includes work by Busschaert and co-workers, who have successfully developed an antibacterial supramolecular host system for phosphatidylglycerol (PG) lipids,^22^ and a bactericidal urea functionalised crown ether construct, which is able to target phosphatidylethanolamine (PE).^23^ In addition, Jolliffe and co-workers have developed a synthetic peptide based fluorescent probe enabling the selective co-ordination of phosphatidylserine (PS),^24^ while Pfeffer and co-workers have produced guanidine based synthetic receptors to enable the co-ordination of Lipid A.^25^

**Fig. 1:**
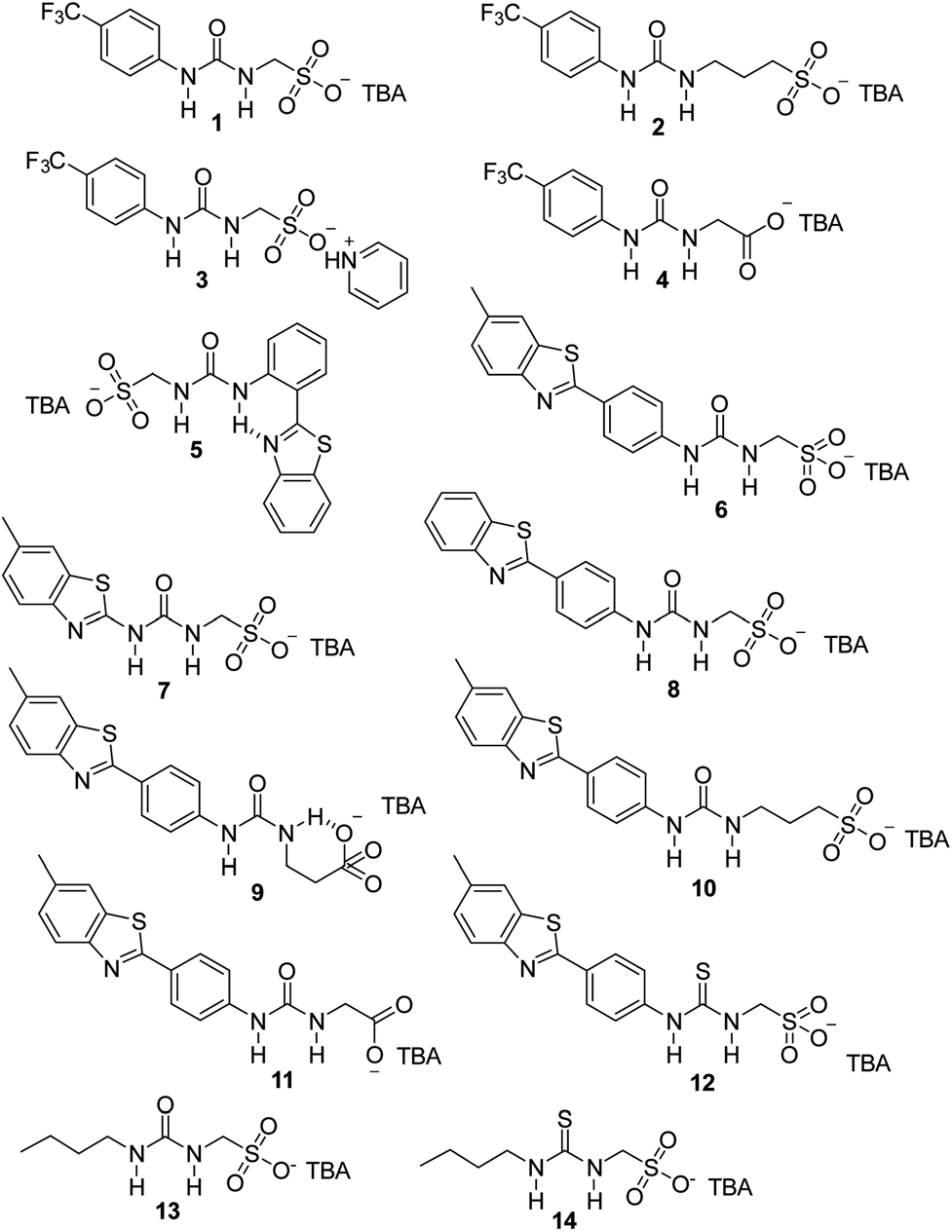
Chemical structures of SSAs 1–14. TBA = Tetrabutylammonium.

Our own work in this area has focused on the development of the Supramolecular Self-associating Amphiphile (SSA) technology. To date, these SSAs, 14 of which are included in Fig. 1, have been shown to: (i) exhibit antimicrobial efficacy against both clinically relevant Gram-positive methicillin resistant *Staphylococcus aureus* (MRSA) and Gram-negative *Escherichia coli* (*E. coli*);^26–29^ (ii) have the potential to act as drug delivery vehicles;^27,30^ (iii) enhance the activity of octenidine, ampicillin and cisplatin against *E. coli* and novobiocin and rifampicin against *Pseudomonas aeruginosa*;^31,32^ and (iv) enhance the activity of the anticancer agent cisplatin against ovarian cancer cells.^33^

**Fig. 2:**
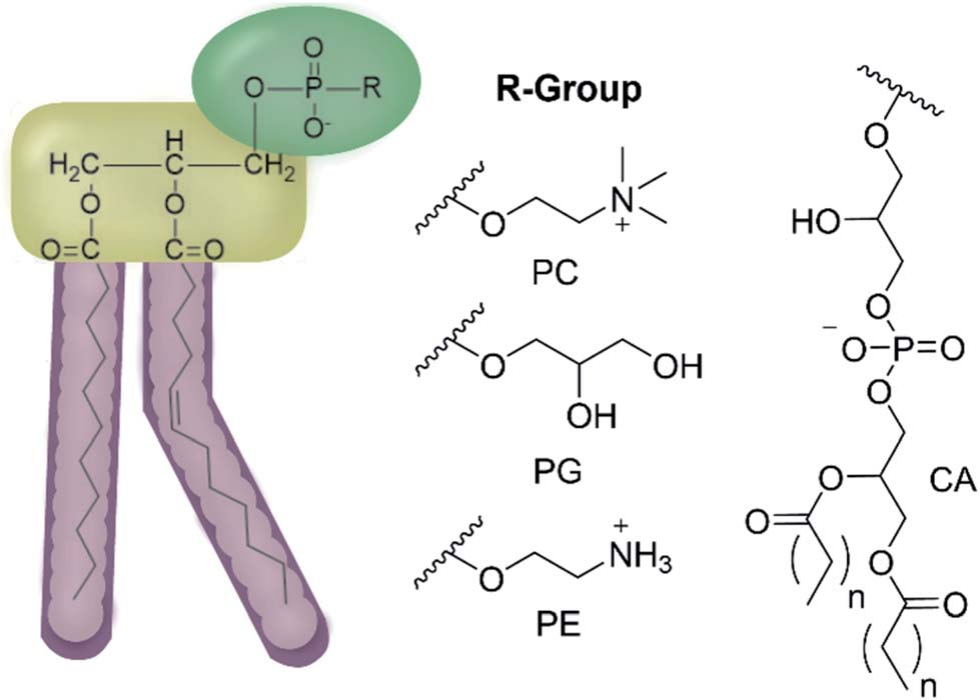
General structure of glycerophospholipids phosphatidylcholine (PC), phosphatidylglycerol (PG), phosphatidylethanolamine (PE) and of cardiolipin (CA). The hydrocarbon chain length and the degree of saturation can differ. Pink = hydrophobic residue. Yellow = glycerol linking group. Green = hydrophilic residue. For CA, here again the carbon chain length and degree of saturation can change.^36^

We have previously hypothesised that SSA therapeutic activity is related to the ability of these agents to selectively coordinate with and permeate membranes of differing phospholipid composition.^34,35^ Herein we move to validate this hypothesis through completion of complementary computational host:guest complex simulations and a combination of synthetic phospholipid vesicle and nanodisc studies using a variety of different homogeneous/heterogeneous phospholipid mixtures. We derive quantitative structure activity relationships to guide next-generation SSA innovation and establish the ‘druggable’ potential of these compounds through completion of a variety of *in vivo* and *in vitro* DMPK studies. The structures of those phospholipids relevant to the work discussed herein are shown in Fig. 2.

## Results and discussion

The 14 SSAs included within this study (Fig. 1) were specifically selected from the SSA molecular library to enable the derivation of the effects of altering key components of the SSA modular molecular structure, such as: (i) changing the counter cation (1*vs.*3); (ii) altering the anionic group (1*vs.*4, 6*vs.*11); (iii) lengthening the anion-(thio)urea alkyl spacer group (1*vs.*2, 6*vs.*9*vs.*10); (iv) exchanging the principle hydrogen bond donating urea group for a thiourea (6*vs.*12, 13*vs.*14); (v) altering the SSA hydrophobic, electron withdrawing/donating functionality (1*vs.*6*vs.*7*vs.*8*vs.*13); and (vi) the formation of intramolecular hydrogen bonds (5*vs.*8, 6*vs.*9). All of which are structural variations which have been previously identified to impact on SSA molecular self-association, aggregate formation, and biological efficacy properties.^28,38,39^

### Synthesis

SSAs 1–6, 10, 13, and 14 were prepared in line with previously published syntheses.^37–41^ SSA 7 was obtained as a white solid in a yield of 91%, through the reaction of tetrabutylammonium (TBA) aminomethane sulfonate with 2-aminobenzothiazole and 1,1′-carbonyldiimidazole (CDI). SSAs 8, 9, and 12 were synthesised through the reaction of the appropriate TBA amino-ate salt with the appropriate (belzothiazol-2-yl)aniline and either triphosgene or thiophosgene, and obtained as either a white or yellow solid in yields of 67%, 64% or 28% respectively. SSA 11 was obtained through the reaction of *tert*-butyl 2-aminoacetate with 4-(6-methylbenzothiazol-2-yl)aniline and triphosgene. This was followed by the deprotection of the resultant crude intermediate with trifluoroacetic acid and the addition of TBA hydroxide (1N) in methanol to afford the final product as a white solid in a yield of 27%. Full synthetic details can be found in the ESI (Section 5).†

### Antimicrobial activity

The antimicrobial activity of SSAs 1–14 was established against both a model Gram-positive, clinically relevant methicillin resistant *Staphylococcus aureus* (MRSA), and a model Gram-negative, *E. coli* bacteria, Table 1. Here MIC_50_ values are given in mM to enable the derivation of molecular structure: antimicrobial efficacy: physical property relationships.

**Table 1:** MIC_50_ values (mM) determined for 1–14 against clinically relevant Gram-positive MRSA USA300 and model Gram-negative *E. coli* DH10B bacteria (*n* = 3) at an initial calibrated cell concentration equal to the 0.5 McFarland standard, after 900 min. Here the number included within the bracket represents the ranking of the SSAs antimicrobial efficacy, with 1 = most active antimicrobial agent. Please see ESI Section 3 for further experimental details and Table S3 for conversion to μg mL^−1^ values

SSA	MRSA	*E. coli*
1 (ref. ^27^)	0.46(2)	3.85(7)
2 (ref. ^28^)	a	1.48(3)
3 (ref. ^28^)	0.35(1)	c
4 (ref. ^28^)	1.14(6)	1.25(2)
5 (ref. ^27^)	0.99(5)	3.57(5)
6 (ref. ^27^)	0.93(4)	5.02(8)
7	c	c
8	1.15(7)	3.66(6)
9	1.20(8)	2.16(4)
10	0.59(3)	1.16(1)
11	0.31–0.63^b^	a
12	a	c
13 (ref. ^28^)	4.41(10)	c
14 (ref. ^28^)	3.07(9)	6.03(9)

a MIC_50_ value could not be determined due to compound solubility.

b MIC_50_ values are estimated due to data quality.

c SSA did not pass initial antimicrobial screening, exhibited <10% inhibition of growth at 3.3 mM after 900 min and therefore excluded from further study.

In general, the SSAs demonstrated an enhanced level of activity against Gram-positive MRSA over Gram-negative *E. coli*. We attribute these observations to the presence of the double membrane – characteristic of Gram-negative bacteria – meaning that a greater concentration of active agent is required to permeate the bacterial cell wall. In addition, we believe that the differences in SSA antimicrobial specificity could be attributed to the differences in the compositions of phospholipids present in the microbial membranes,^27,28,34,35^ supporting the hypothesis that selective interaction/permeation of the microbial phospholipid membranes is key for SSA antimicrobial efficacy. For example, the replacement of conjugated ring systems (1–12) with a butyl group (13 and 14) was found in general to decrease antimicrobial efficacy, possibly due to the deactivation of the hydrogen bond donating/accepting (thio)urea group and/or removal of possible π-π stacking interactions and changes in molecular lipophilicity/hydrophobicity, decreasing the ability of these compounds to self-associate, associate with a biological target and permeate a biological membrane. Introduction of the trifluoromethyl appended phenyl group with a methyl anion-urea alkyl linking group, pyridinium counter cation and a sulfonate anionic component was found to promote SSA activity against MRSA however, the presence of a urea functionality, combined with a longer urea-anion alkyl spacer was found to promote SSA activity against *E. coli*.

### SSA phospholipid membrane interactions

To derive molecular structure:phospholipid bilayer interaction/permeation/lysis relationships a series of computational phospholipid headgroup interaction studies were performed in combination with a selection of synthetic vesicle studies, and complementary nanodisc assays. All SSAs 1–14 (except 3) contain an anionic component with a chemically distinct molecular structure and the same TBA counter cation.

### Computational phospholipid headgroup SSA interaction studies

Initially, computational modelling methods were used to provide some insight into the antibacterial activity of the SSAs, through the provision of evidence towards the selective coordination of the anionic component of an SSA towards different phospholipid headgroups. Here, we studied the binding interactions of the anionic component of two of the simplest SSA structures, SSAs 1 (or 3) and 4 (Fig. 1), which differ only in the nature of the anionic moiety – with model phospholipid headgroups (m-lipids). By modelling various SSA-phospholipid binding conformations and energies, we could determine whether SSAs interact preferentially with the headgroups of bacterially prevalent lipids (PE and PG) over lipids more commonly found in mammalian cells (phosphatidylcholine – PC), as well as compare the effect of varying the structure of the SSA on the binding energy. A full account of the computational methods used, choice of molecular modelling studies undertaken and data produced is presented within the ESI (Section 15).†

**Fig. 3:**
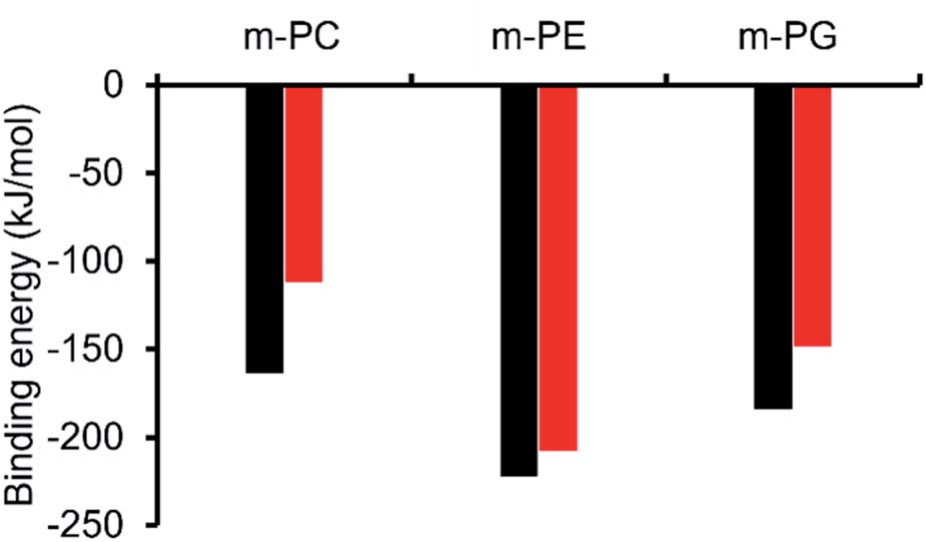
Comparison of the lowest energy SSA-phospholipid binding energies with model lipid headgroups representative of bacterial lipids (m-PE, m-PG) and mammalian lipids (m-PC) (kJ mol^−1^) with the anionic components of SSA 1/SSA 3 (Black) and SSA 4 (Red), calculated using HF/3-21G.

Using *ab initio* methods (HF/3-21G), we calculated the binding energies of the SSA-phospholipid headgroup pair for SSAs 1 and 4 with m-PC, m-PE, and m-PG lipid headgroups. We chose to model only a simplified headgroup, as this is the portion of the lipid expected to be presented on the surface of cells and to initially interact with the SSAs; this also reduced computational complexity. A comparison of the lowest binding energies is shown in Fig. 3, from which two main conclusions can be drawn. Firstly, SSA 1 (or 3) exhibits a slightly decreased binding energy, when compared to SSA 4, for all three lipids, indicating the greater affinity of this SSA towards the phospholipid headgroups included within the scope of this work. However, the difference in these binding energies is decreased for the bacterial phospholipids, in particular m-PE, reducing the likelihood of any real-world difference in binding affinity between these two SSAs towards the same phospholipid headgroup. Secondly, both SSAs exhibit the same lipid preferences – both demonstrating a higher affinity for the headgroups of bacterially prevalent lipids m-PE and m-PG, over m-PC. This suggests that these SSAs have the potential to exhibit a greater affinity towards bacterial membranes over those of normal mammalian cells. This is clearly demonstrated when considering the binding affinity of SSA 4 towards m-PC and m-PE headgroups. Therefore, the findings of these simple computational studies support SSA technology as a plausible approach for the selective targeting of bacterial cell membranes.

**Fig. 4:**
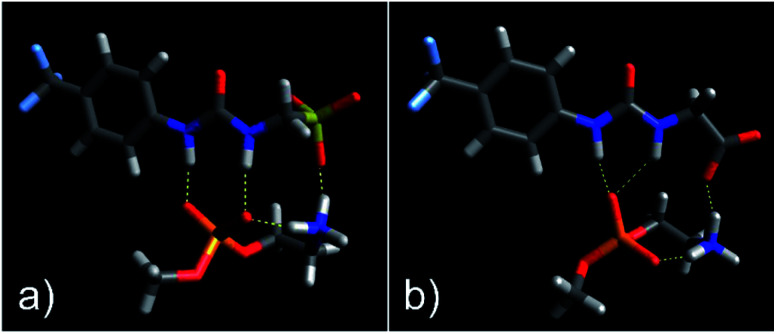
Graphic illustrating the hydrogen bonded complexes formed between the model m-PE phospholipid headgroup of (a) SSA 1 (or SSA 3) and (b) SSA 4, calculated using HF/3-21G. Here: grey = carbon; light blue = fluorine; dark bule = nitrogen; white = hydrogen; red = oxygen; orange = phosphorus; and yellow = sulfur.

As shown in Fig. 3, the most negative binding energies for the SSA:phospholipid combinations tested were obtained for the anionic component of SSAs 1 (or SSA 3) and 4 with the m-PE phospholipid headgroup. The complexes formed by these interactions, as predicted by our computational modelling studies, are shown in Fig. 4a and Fig. 4b respectively. Here interestingly, a predicted intramolecular hydrogen bond is formed between the ammonium and phosphate portions of the headgroup itself, stabilising the geometry of the phosphate guest species. The SSA anions then bind to the phosphate guest through the formation of three hydrogen bonds, two from the SSA urea NH hydrogen bond donating groups to the central phosphate moiety, utilising two hydrogen bond accepting oxygen atoms for SSA 1 (or SSA 3) and a single hydrogen bond accepting oxygen atom for SSA 4. This difference in complex formation can perhaps explain the slight difference in binding energies observed for these two SSA anions. The anionic functionality contained within the SSA structure then forms a hydrogen bond with the ammonium functionality of the m-PE headgroup, with the SSA now acting as the hydrogen bond acceptor (A), while the phosphate guest species acts as the hydrogen bond donor (D). Therefore, the low binding energy observed for the SSA:phosphate complex formed in this instance can be attributed to the complementary DDA groups of the SSA anion with the AAD groups of the PE phospholipid headgroup.

### Synthetic phospholipid vesicle lysis assay

To explore the interaction of SSAs 1–14 with phospholipid membranes, synthetic vesicles were prepared from a variety of homogeneous and heterogenous phospholipid mixtures, used to simulate the membranes of human (PC)^42^ and bacterial cells (homogeneous/heterogenous combinations including PE and PG – Table 2).^43,44^ In addition, we also chose to include phospholipid mixtures directly extracted from *E. coli* (total and polar), enabling comparison with those antimicrobial efficacy data summarised in Table 1.^21^ However, as reported by Bose and co-workers, the phospholipid membrane composition of MRSA USA300 is predominantly PG with an absence of PE, thus when identifying analogous phospholipid systems to represent this bacteria, we believe 100% PG acts as an appropriate substitute for those phospholipid mixtures derived from natural sources.^45^

**Table 2:** Lipid compositions (%) of the five types of homogenous/heterogenous lipid vesicles used within the scope of these studies. For lipid structures see Fig. 2

Lipid vesicle	PC	PG	PE	CA	Unknown
PC	100	x	x	x	x
PG	x	100	x	x	x
PE-PG mix	x	25	75	x	x
*E. coli* total^46^	x	15	57	10	18
*E. coli* polar^47^	x	23	67	10	x

SSAs are amphiphilic compounds and thus exhibit surfactant type properties. As the mode of antimicrobial action for this class of compounds may relate to their inherent membrane disruption properties, we first chose to establish the lysis activity of SSAs 1–14 against vesicles composed of the five different phospholipid mixtures detailed in Table 2.

**Fig. 5:**
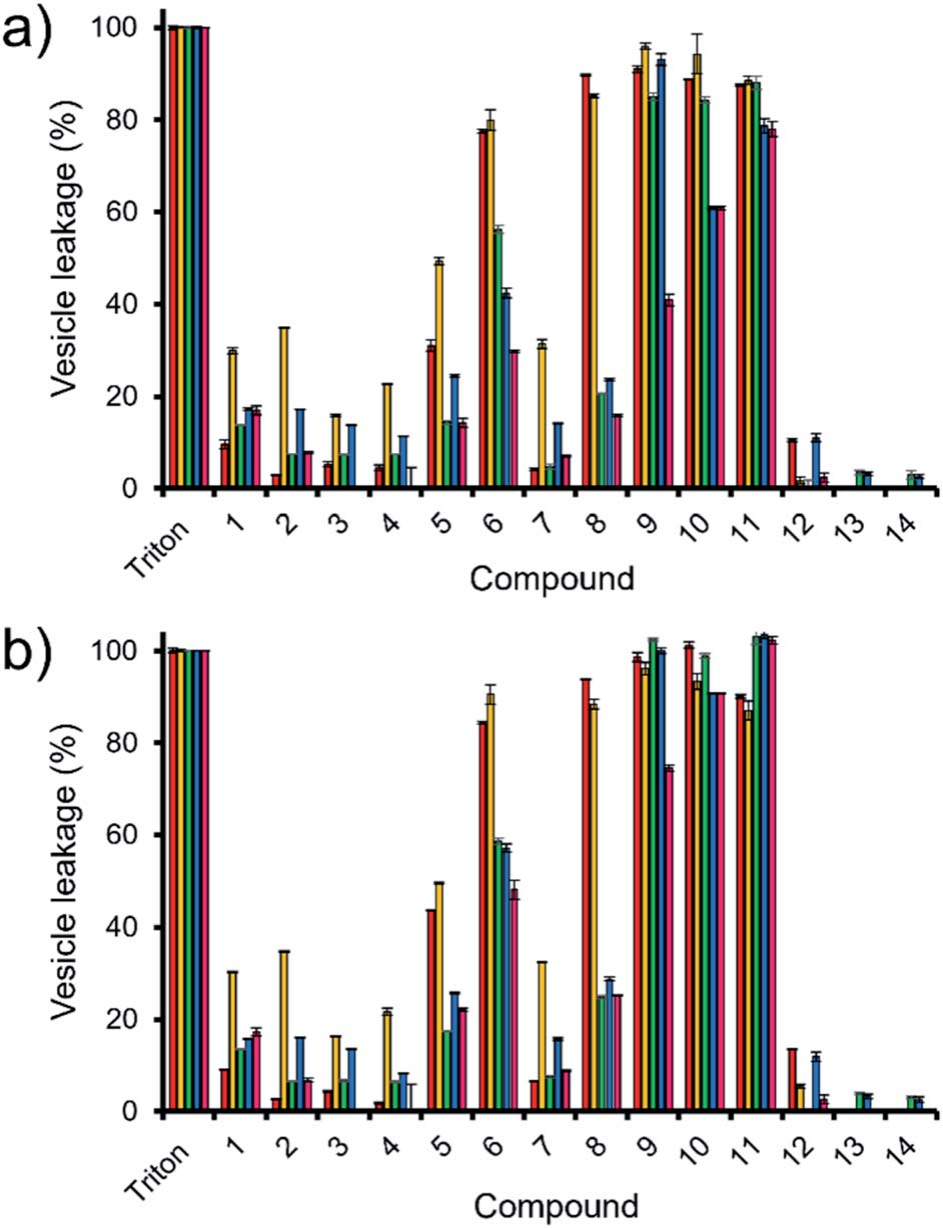
The average (*n* = 3) percentage lysis (%) of vesicles (30 μM) composed of five different lipid compositions (Table 2), with an internal calcein concentration of 70 mM at 298 K. The % vesicle lysis was calculated through observation of increasing fluorescence emission (*λ*_em_ = 515 nm), following the addition of SSAs 1–4 (1.5 mM) in a H_2_O/EtOH 95 : 5 solution after: (a) 30 s and; (b) 15 min. Triton X-100 (1%) was used as a positive control for 100% vesicle lysis. Data normalised to the addition of a H_2_O/EtOH 95 : 5 solution. Red = PC, yellow = PG, green = PE-PG mix, blue = E. *coli* total, pink = *E. coli* polar. Error = standard error.

Here, samples of phospholipid vesicles containing the fluorescent dye calcein were prepared. At internal vesicle concentrations, this dye undergoes self-quenching. However, should the vesicle membranes be disrupted to a point that results in dye leakage, the effective concentration of the dye will be dramatically reduced, resulting in the amplification of calcein fluorescence emission. The increase in fluorescence output is proportional to the % of vesicle lysis observed upon the addition of SSAs 1–14, when calibrated against a solution of vesicles know to have undergone 100% lysis, see Fig. 5.

Thirty seconds after SSA addition (Fig. 5a), SSAs 1–5 and 7 showed vesicle lysis selectivity for the PG vesicles over the other phospholipid vesicles tested. SSAs 11–14 showed no real selectivity for one phospholipid membrane over another, with little activity observed at all for SSA 12–14 and general surfactant type properties, similar to that of Triton, observed for SSA 11. Of those SSAs exhibiting a lysis selectivity towards PG vesicles, SSAs 1–4 all contain a urea-anion alkyl linking group and a *para* substituted trifluoromethyl hydrophobic phenyl ring system. The selectivity for the PG phospholipid vesicles was found to be independent of either anion (sulfonate or carboxylate) or alkyl linking group (methylene or propylene). However, the substitution of the TBA (1, 2 and 4) for a pyridinium (3) counter cation was found to decrease SSA vesicle selectivity and lysis properties. We believe that this is due to both the enhanced lipophilic and the reduced ion pairing properties of the TBA over the pyridinium cation, supporting both SSA anion phospholipid interaction and SSA anion self-association. Replacement of the phenyl ring systems with an alkyl chain (13 and 14) resulted in an almost complete loss of any vesicle lysis activity. We hypothesise that this is principally due to the deactivation of the hydrogen bond donating (thio)urea substituent, as a result of the presence of an adjoining electron donating butyl, rather than the presence of the electron withdrawing aromatic ring systems, resulting in decreased SSA self-association/complexation properties.^28,39^

However, the addition of the benzothiazole functionality (5–12) to the SSA structure results in an increase in vesicle lysis when compared to the analogous trifluoromethyl substituted SSAs with exception of 7 and 12. We hypothesise that this is due to: (i) the loss of molecular planarity caused by the exchange of urea (6) for the thiourea (12) functionality preventing optimal SSA self-association/integration despite the increased lipophilicity and acidity of the hydrogen bond donating thiourea over the urea moiety; and (ii) the removal of a benzene ring system (7) limiting preferential π–π stacking interactions.

The removal of the methyl group from SSA 6 gives rise to SSA 8; which exhibits a decrease in comparative vesicle lysis properties against all phospholipid membranes apart from those containing 100% PC and PG, leading to increased selectivity. Increasing the chain length of the urea-anion alkyl linker (ethylene – 9, propylene – 10) results in a stepwise increase in *E. coli* polar vesicle lysis with respect to alkyl chain length, supporting trends observed within *E. coli* antimicrobial efficacy experiments (Table 1), where increasing urea-anion alkyl chain length resulted in increased antimicrobial efficacy. This is a trend that was also observed for the lysis of PC and PE-PG mixed vesicles however, this effect was enhanced for the polar bacterial PE-PG mix vesicles over the non-polar model human PC phospholipid membranes.

Leaving the SSAs in contact with these vesicle samples for an extended period (15 min – Fig. 5b) did show some time dependent enhancement of vesicle lysis for those benzothiazole substituted urea SSAs 6, 8, 9 and 10 against *E. coli*-based vesicles. This observation correlates with the time dependent internalisation of SSA 6 previously observed against *E. coli* DH10B.^28^ From this, we conclude that the presence of cardiolipin (CA) is slowing the lysis effects of these SSAs against the bacterial derived membranes. Thus, to accurately model the permeation properties of small molecules within a synthetic phospholipid mixture (3 : 1 ratio of PE : PG), 10% CA should also be added to this simple heterogeneous phospholipid mixture.

**Fig. 6:**
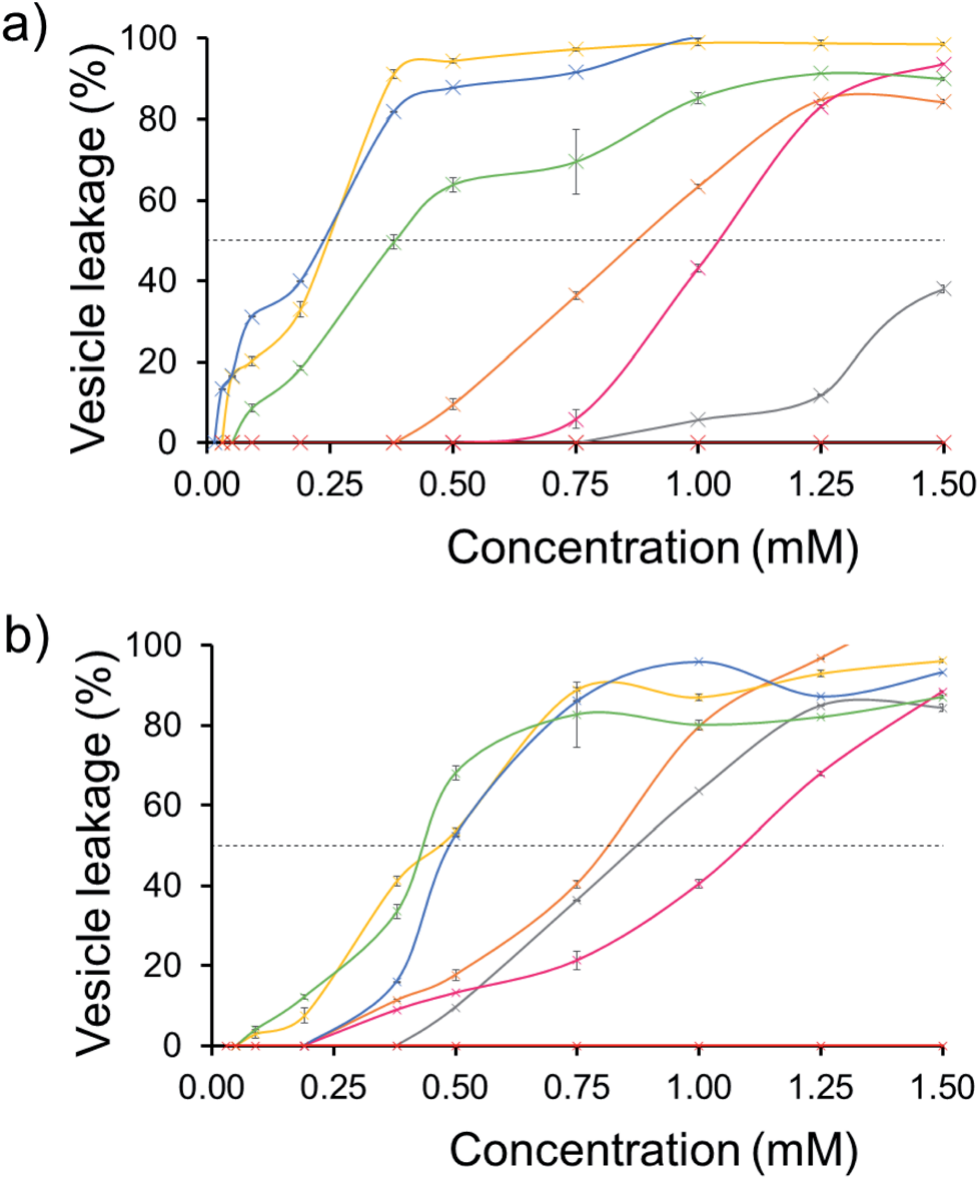
The average (*n* = 3) percentage lysis (%) of vesicles (30 μM) composed of five different lipid compositions (Table 2), with an internal calcein concentration of 70 mM at 298 K. The % (a) PC and (b) PG vesicle lysis was calculated through observation of increasing fluorescence emission (*λ*_em_ = 515 nm), following addition of SSA 5 (grey), SSA 6 (orange), SSA 8 (pink), SSA 9 (yellow), SSA 10 (blue), SSA 11 (green) and control solution 5% EtOH (red) after 15 min. Error = standard error. Black dotted line = 50% vesicle lysis.

The effect of SSA concentration on the percentage lysis of the five different types of phospholipid vesicle was also studied for those SSAs (5, 6, 8–11) which exhibited the greatest degree of lysis activity. These titration experiments focused on determining the percentage vesicle lysis at SSA concentrations ≤1.5 mM. The results of these studies conducted against the PE : PG mix and *E. coli* total and *E. coli* polar lipid vesicles were found to give relatively similar results (Section 10).† However, the results of these SSA titrations conducted against both PC and PG vesicles did show some interesting, SSA concentration-dependent selectivity, as shown in Fig. 6.

The results of these titration studies showed SSAs 5, 6 and 8 to exhibit enhanced levels of vesicle lysis against PG (a bacterial lipid prevalent in MRSA) over PC (used to mimic model human phospholipid membranes) vesicles at concentrations ≤0.5 mM (Fig. 6). This indicates that the use of a sulfonate-urea group, containing a methyl linking functionality may enable SSA antimicrobial activity against MRSA while decreasing toxicity of these SSAs against normal human cells. Elongation of this urea-anion linking group and substitution of the sulfonate for the carboxylate ion was found to invert this trend in membrane lysis selectivity at concentrations ≤0.5 mM. Thus, by this same reasoning, these SSA functionalities may want to be avoided if unwanted toxicity against mammalian systems should be observed. These trends in selectivity for PG over PC are also in part supported by the binding energies calculated within the scope of our computational models, which are consistently lower (thus more favourable) for the sulfonate (1 and 3) over the carboxylate (4) substituted SSAs.

### Synthetic vesicle membrane fluidity assay

To further explore the effects of SSAs on physical phospholipid membrane characteristics, membrane fluidity experiments were performed. Here a dye (1,6-diphenyl-1,3,5-hexatriene – DPH) was inserted into the synthetic vesicle membrane to enable the study of increased/decreased membrane fluidity through competitive fluorescence polarisation (FP) measurements. In these studies, FP values are found to decrease with increasing membrane fluidity and *vice versa*. However, these studies could only be performed with SSAs 1–4 and 7 due to overlapping SSA/DHP fluorescence emission. In addition, SSAs 13 and 14 were also excluded from this study due to the lack of activity demonstrated within the scope of the phospholipid vesicle lysis experiments.

**Fig. 7:**
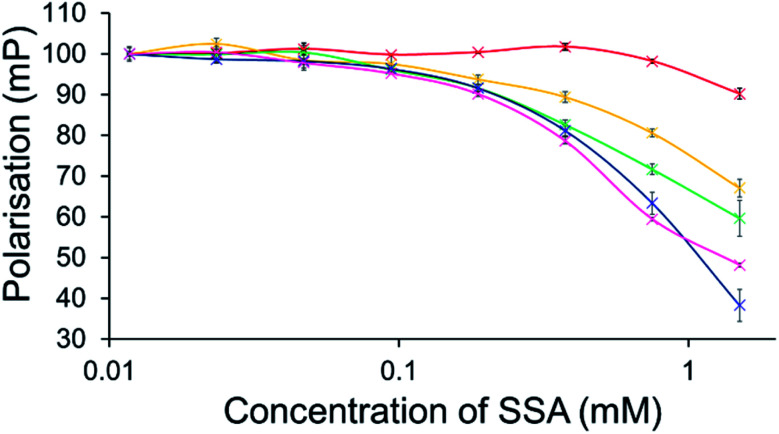
Effect of increasing SSA 4 concentrations on average (*n* = 3) FP (mP) values, measured for five different DPH-labelled synthetic vesicles (1 mM) at 298 K. A FP value of 100 mP was initially set for each DPH-labelled vesicle in the presence of a 5% EtOH control solution. The DPH-labelled vesicle concentration was kept constant throughout the FP titration. Error = standard error. Red = PC; yellow = PG; green = PE-PG mix; blue = E. *coli* polar; pink = *E. coli* total. For phospholipid vesicle compositions please see Table 2.

Only SSA 4 was found to mediate an overall change in FP > 10% (Fig. 7). Interestingly, this increase in membrane fluidity was confined to those polar phospholipid membranes, excluding those containing PC only, our human model cell system. Here there is a clear structure activity relationship, the presence of a carboxylate ion results in selective increases in membrane fluidity for model bacterial cell membranes.

### Synthetic phospholipid vesicle membrane binding assay

For intrinsically fluorescent SSAs 5, 6 and 8–12, FP titration experiments enabled the observation of SSA: phospholipid vesicle interactions. Here, an increase in FP value indicates an increase in the apparent size of the SSA as it interacts with the phospholipid vesicle.

**Fig. 8:**
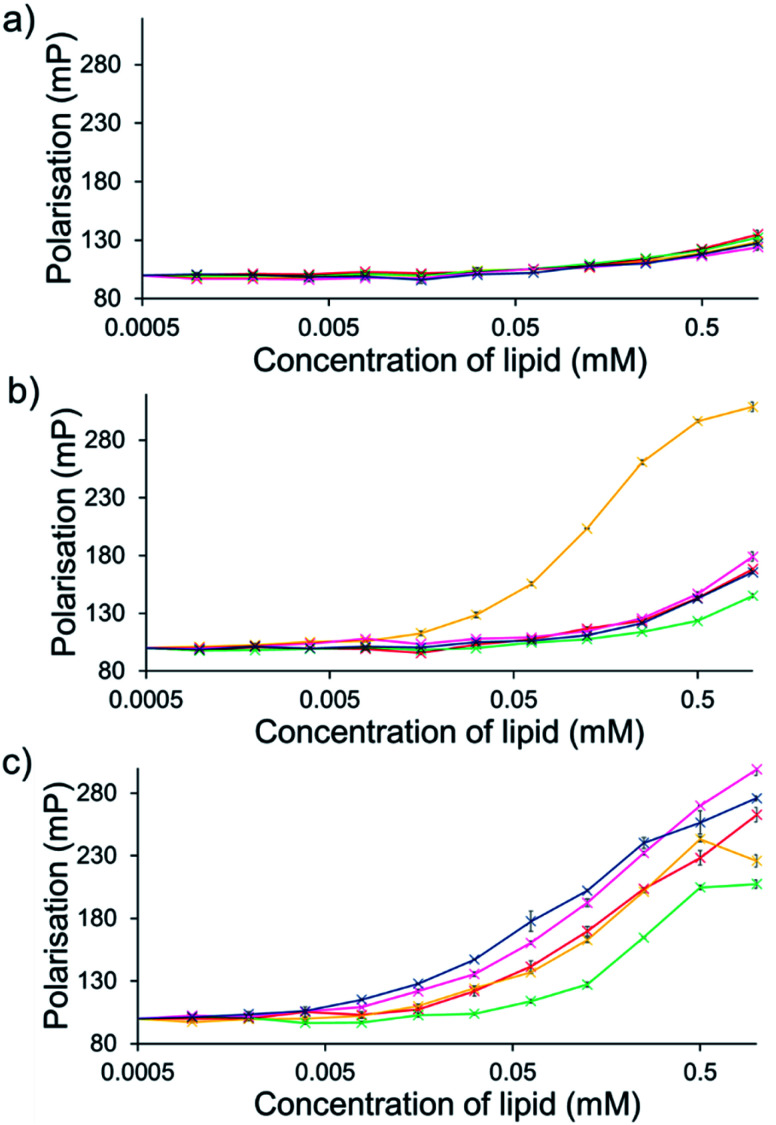
Average (*n* = 3) change in FP (mP) for solutions of SSA (0.15 mM) (a) 8, (b) 11 and (c) 12 upon the addition of synthetic vesicles of five different phospholipid compositions at 298 K. A target control value of 100 mP was set for the relevant fluorescent SSA alone, before the addition of any vesicle. The concentration of SSA was kept constant throughout the FP titration. Error = standard error. Red = PC, yellow = PG, green = PE-PG mix, blue = E. *coli* polar, pink = *E. coli* total.

The results of these studies have been summarised for convenience in Table 3 however, specific SSA: phospholipid vesicle interactions are also exemplified within Fig. 8. Both SSAs 8 (Fig. 8a) and 10 showed little selectivity for different phospholipid membranes, with only a slight increase in FP observed throughout the titration, suggesting weak SSA:phosphlipid interactions. However, both SSA 11 (Fig. 8b) and SSA 9 showed selectivity towards a single type of phospholipid vesicle, *E. coli* total and PG respectively. SSA 6 demonstrated limited selectivity with a preference for PC, PG and *E. coli* polar over the *E. coli* total and PG : PE mix. Finally, SSA 12 was shown to demonstrate a strong interaction with all phospholipid vesicles tested (Fig. 8c). Here phospholipid vesicle selectivity appears to be driven by the presence of a methyl substituent appended from the benzothiazole unit, in addition to a urea-methyl/ethyl spacer-anionic functionality. Interesting the presence of the thiourea group, increasing SSA lipophobicity and increasing hydrogen bond donor acidity, while increasing the levels of vesicle interaction overall, decreased any specific SSA selectivity. These data, as well as the fluidity data collected using DPH, show different trends from those seen in the phospholipid vesicle lysis studies conducted at 1.5 mM (Fig. 2), suggesting that the membrane interaction and vesicle lysis experiments rely on different SSA chemical/physicochemical properties, that are likely to be dependent on effective SSA concentration, and thus the self-associated structure present.

**Table 3:** The average (*n* = 3) increase in FP (mP) observed when lipid vesicles were added to SSAs 5, 6, 8–12 (0.15 mM). Error = standard error. CMC was derived from surface tension measurements for SSAs in a 19 : 1H_2_O : EtOH solution at 298 K. For lipid composition please see Table 2. A target control value of 100 mP was set to the relevant fluorescent SSA alone, before the addition of any vesicle. The concentration of SSA was kept constant throughout the FP titration, at concentrations well below the CMC to prevent the formation of larger SSA self-associated aggregate species^27,28,30,38,39^

Lipid	5	6	8	9	10	11	12
PC	87 ± 4	47 ± 8	35 ± 4	35 ± 10	26 ± 1	68 ± 1	163 ± 6
PG	43 ± 9	40 ± 4	29 ± 1	47 ± 1	29 ± 2	209 ± 4	126 ± 5
PE-PG mix	36 ± 3	9 ± 1	32 ± 1	35 ± 2	21 ± 2	45 ± 2	107 ± 3
*E. coli* total	32 ± 3	20 ± 1	24 ± 3	130 ± 5	15 ± 2	66 ± 4	199 ± 5
*E. coli* polar	40 ± 4	35 ± 3	27 ± 2	53 ± 3	25 ± 14	79 ± 1	176 ± 2
CMC	9.5 (ref. ^38^)	0.5 (ref. ^38^)	5.2	1.9	14.8	3.0	4.6

#### Phospholipid nanodisc ^1^H NMR adhesion assay

Finally, to establish how any single SSA structural variation affects the coordination of an individual salt component to the surface of a phospholipid membrane, the binding of the anionic and cationic components of SSAs 5–12 with nanodiscs comprised of PC, PG or the PE : PG mix (Table 2) was studied. Nanodiscs were synthesised from synthetic vesicles of the relevant phospholipid composition. For full synthetic procedures and nanodisc characterisation please see ESI (Section 13).†^34,35^

Styrene-maleic acid (SMA) nanodiscs are small biomimetic scaffolds with a hydrodynamic diameter ≈ 10 nm. They consist of a single disc-shaped planar phospholipid bilayer, ‘belted’ in this instance with a synthetic SMA co-polymer. Here nanodiscs of a specific phospholipid composition were titrated against a solution of SSAs 5–12 (0.10 mM) in a sodium phosphate buffer (20 mM) with NaCl (20 mM) at pH 7.4. This solution was supplemented with 5% D_2_O, to enable NMR locking and 4,4-dimethyl4-silapentane-1-sulfonic acid (DSS – 0.02 mM) to act as an internal standard. The nanodiscs were then titrated against the SSA, while the SSA concentration was maintained as constant, well below the CMC (Table 3).^38^ A 1D ^1^H NMR with a Carr–Purcell–Meiboom–Gill sequence (CPMG) filter (300 ms),^36,48^ modified with a watergate element was then obtained for each experimental data point. The watergate element was used to allow suppression of the water signal, whereas the CPMG filter enabled the suppression of those resonances from molecular species with long correlation times, which in this instance correlate to the signals of those SSAs coordinated to the nanodisc, rather than those which exist as free species in solution. Comparative integration of the resonances corresponding to the SSA cation or anion, standardised against the DSS internal standard, permits the percentage of the individual SSA components coordinated to the phospholipid nanodisc to be established with respect to increasing nanodisc concentration.

**Fig. 9:**
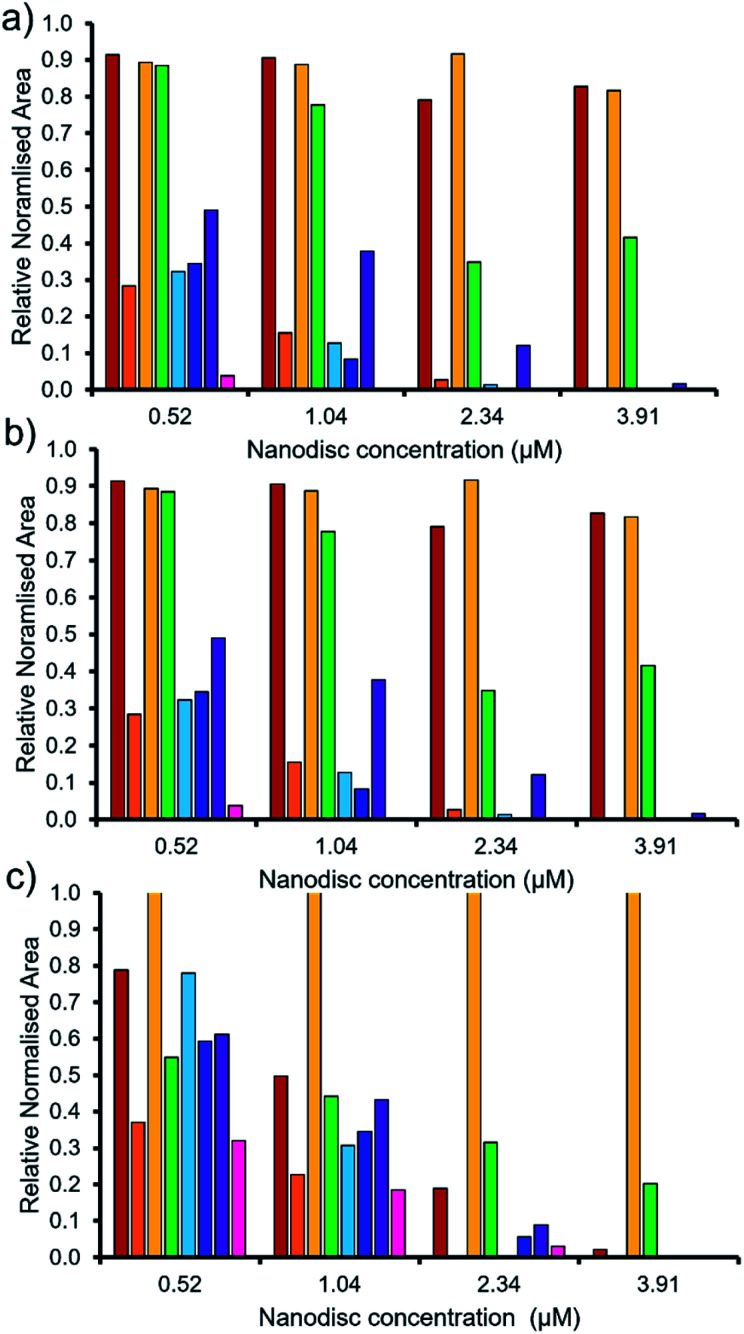
The relative normalised area obtained from 1D ^1^H CPMG NMR spectra for the anionic component of SSA (0.10 mM) 5 (red), 6 (orange), 7 (yellow), 8 (green), 9 (light blue), 10 (dark blue), 11 (purple) and 12 (pink) against (a) PC, (b) PG and (c) PE : PG mixed nanodiscs (Table 2). Here 1.0 = 100% of SSA anion ^1^H NMR signal available for integration and, 0.0 = 0% of the SSA anion ^1^H NMR signal available for integration (100% of the signal is NMR silent, thus assumed bound to the nanodisc). 1D ^1^H CPMG NMR experiments were standardised with 5% D_2_O and 0.02 mM DSS.

As summarised within Fig. 9, the anionic component of SSA 7 was found not to interact with any great significance to any of the phospholipid nanodiscs tested. This SSA also did not exhibit any antimicrobial efficacy (Table 1), again supporting the hypothesis that the SSA mode of antimicrobial activity relates to the ability of the SSA to adhere to the cell membrane. Interestingly, the anionic component of SSA 12 shows the greatest binding affinity for all three nanodiscs of the SSAs tested, correlating with the results of the synthetic phospholipid vesicle membrane binding assays; however, this SSA also demonstrated one of the lowest comparative vesicle lysis and antimicrobial activities (Fig. 5 and Table 1 respectively). This suggests that increased levels of phospholipid interaction alone, which results in this instance from the enhanced acidic (hydrogen bond donor)/lipophilic properties of this SSA, provided by the thiourea moiety, may not result in sufficient membrane disruption/permeation events to elicit a therapeutic effect.

**Fig. 10:**
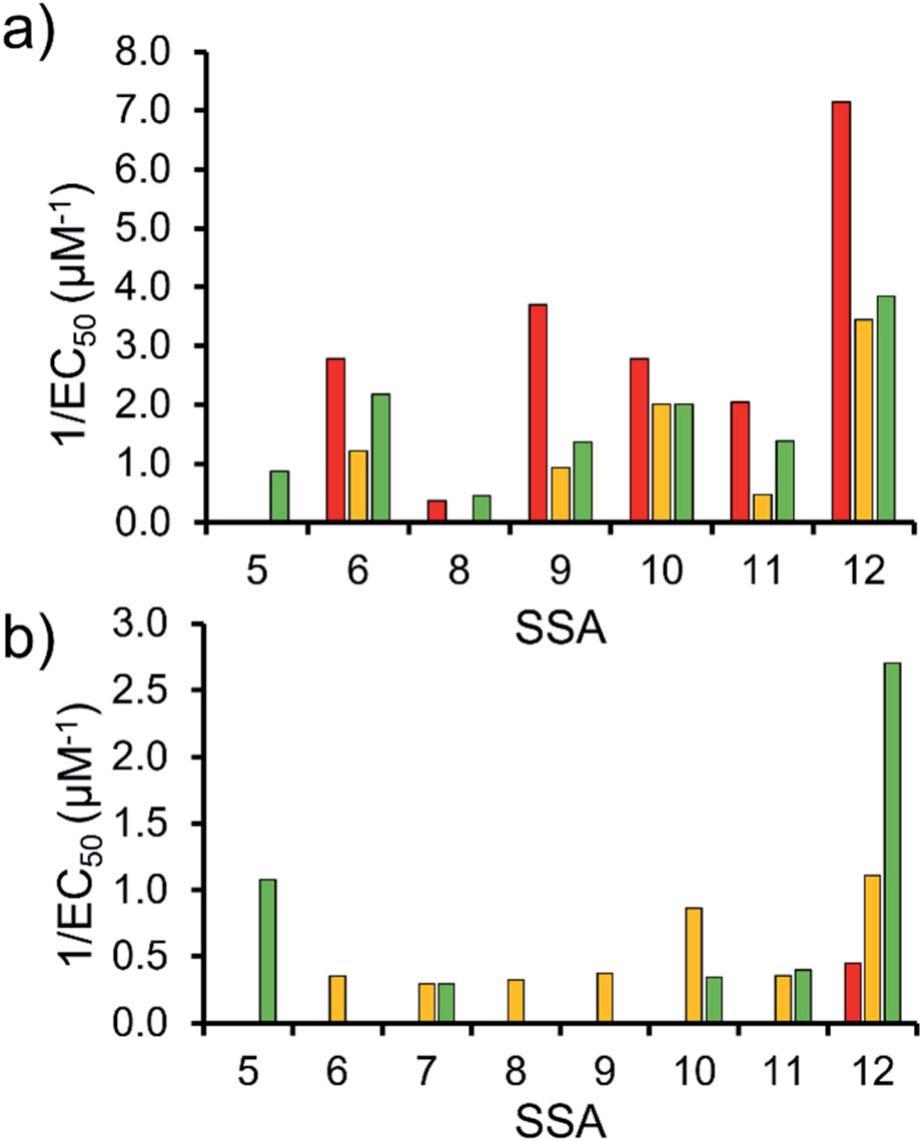
1/EC_50_ (μM^−1^) values obtained from the fitting of the (a) SSA anion or (b) SSA cation nanodisc titration data to Hill Plot kinetics using Origin 2018 software, with *V*_max_ fixed to 100% of the SSA component bound to the nanodisc. Where *R*^2^ < 0.85, the results of data fitting are not reported. Red = results from PC phospholipid nanodisc titration studies; yellow = results from PG phospholipid nanodisc titration studies and; green = results from PE : PG mix phospholipid nanodisc titration studies (Table 2). For clarity, errors associated with these measurements are given in Table S4.†

To enable effective analysis, these data were fitted to the Hill Plot model, enabling calculation of the effective concentration of nanodisc required to coordinate 50% of either the SSA anionic or cationic component independently, the EC_50_ value (see ESI – Section 14).† To enable comparison of these date, the reciprocal EC_50_ (1/EC_50_) was calculated where possible for both the anionic and cationic component of each SSA (Fig. 10). Where a 1/EC_50_ could be calculated (6, 9–12), the SSA anion was shown to preferentially bind to the PC nanodisc, over model bacterial phospholipid nanodiscs (Fig. 10a). However, when considering the 1/EC_50_ for the SSA cationic component (Fig. 10b), the reverse is true. This is perhaps expected, but nevertheless despite the identical experimental conditions, including the same TBA counter cation across SSAs 5–12, there is a marked difference in the 1/EC_50_ obtained, where the only difference is the SSA anion present. These results also do not correlate with CMC values provided in Table 3.^38^ This is most noticeable for the series of SSA titrations involving the PE : PG mix (Table 2) nanodiscs. The only variation within this set of titrations is the structure of the SSA anion; we therefore conclude that the SSA anion is influencing SSA cation nanodisc coordination events. This observation provides evidence for the presence of further complex interactions informing SSA:nanodisc coordination processes.

### Comparison of phospholipid interaction/lysis data and SSA antimicrobial efficacy

Preliminary computational modelling studies support the hypothesis that the molecular structure of the SSAs themselves can be tailored to selectively interact with the phospholipid headgroups of differing chemical composition. Here the anionic component of both SSAs 1 (or SSA 3) and SSA 4 demonstrate selectivity for the bacterial phospholipid model headgroups (PE and PG) over PC, a phospholipid contained on the external surface of normal mammalian cells. In addition, the results obtained from vesicle lysis, membrane fluidity studies, and various membrane coordination assays suggest that each SSA possesses a combination of unique membrane coordination, membrane permeation, and membrane lysis activities. It is therefore likely that the antimicrobial efficacy of each SSA 1–14, exists as the result of a balance of these and other, as yet unidentified, molecular properties.

**Fig. 11:**
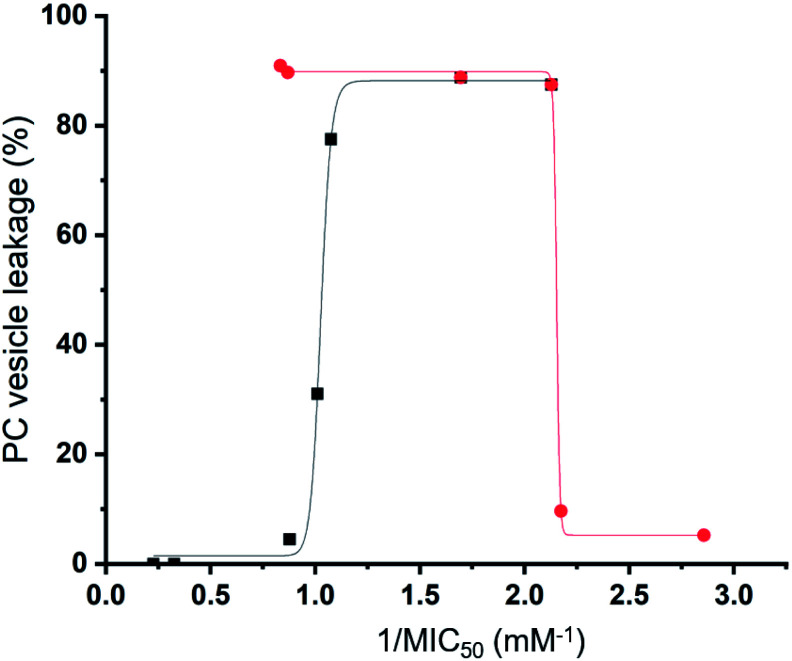
Logistical fit analysis of % PC vesicle lysis data against 1/MIC_50_ (mM^−1^) obtained for SSAs 1–14. Black = 1/MIC_50_ (mM^−1^) increasing with increasing PC vesicle leakage (%). Red = 1/MIC_50_ (mM^−1^) increasing with decreasing PC vesicle leakage (%).

This hypothesis is further supported by analysis of the quantitative values from our synthetic membrane lysis/permeation/interaction studies, through logistical fit analysis (using Origin 2018), with SSA antimicrobial activity. As shown in Fig. 11, when plotting the results of % PC vesicle lysis at 30 seconds after SSA addition, against 1/MIC_50_ values obtained for MRSA, the % vesicle lysis is found to initially increase with increasing antimicrobial efficacy to a point (Fig. 11 – black; *R*^2^ = 0.977). This supports the hypothesis that the increase in antimicrobial efficacy may be due to a simple increase in the general surfactant properties of the SSA. However, with this maximum reached, any additional enhancement of the SSA antimicrobial efficacy was found to correlate with decreasing % PC vesicle lysis (Fig. 11 – red; *R*^2^ = 0.999), meaning that another physicochemical parameter must be responsible for any further enhancement of antimicrobial efficacy.

#### *In vitro* DMPK studies

To determine the translational potential of SSAs into the clinic as ‘druggable’ agents, a range of *in vitro* DMPK studies were performed using SSAs 1–6 and 10 as a representative seven compound subset of the 14 SSAs considered within the scope of the original membrane lysis studies. Here SSA metabolic stability was determined against mouse, rat, and human liver microsomes. Mouse plasma protein binding (PPB) assays were performed to estimate the ability of an SSA to distribute into the tissues of the body. All SSAs tested exhibited high plasma stability and acceptable % recoverability values to validate the % PPB values obtained. To gauge the suitability of SSAs for oral dosing, these seven SSAs were further analysed using a Caco-2 permeability assay. The results of these studies confirmed high levels of compound efflux for all SSAs tested, meaning that these SSAs are likely to exhibit poor oral absorption characteristics, thus favour intravenous (i.v.) administration. A summary of the results obtained for these studies in addition to full discussion of these data can be found in Section 4 of the ESI.†

#### *In vivo* DMPK studies

**Fig. 12:**
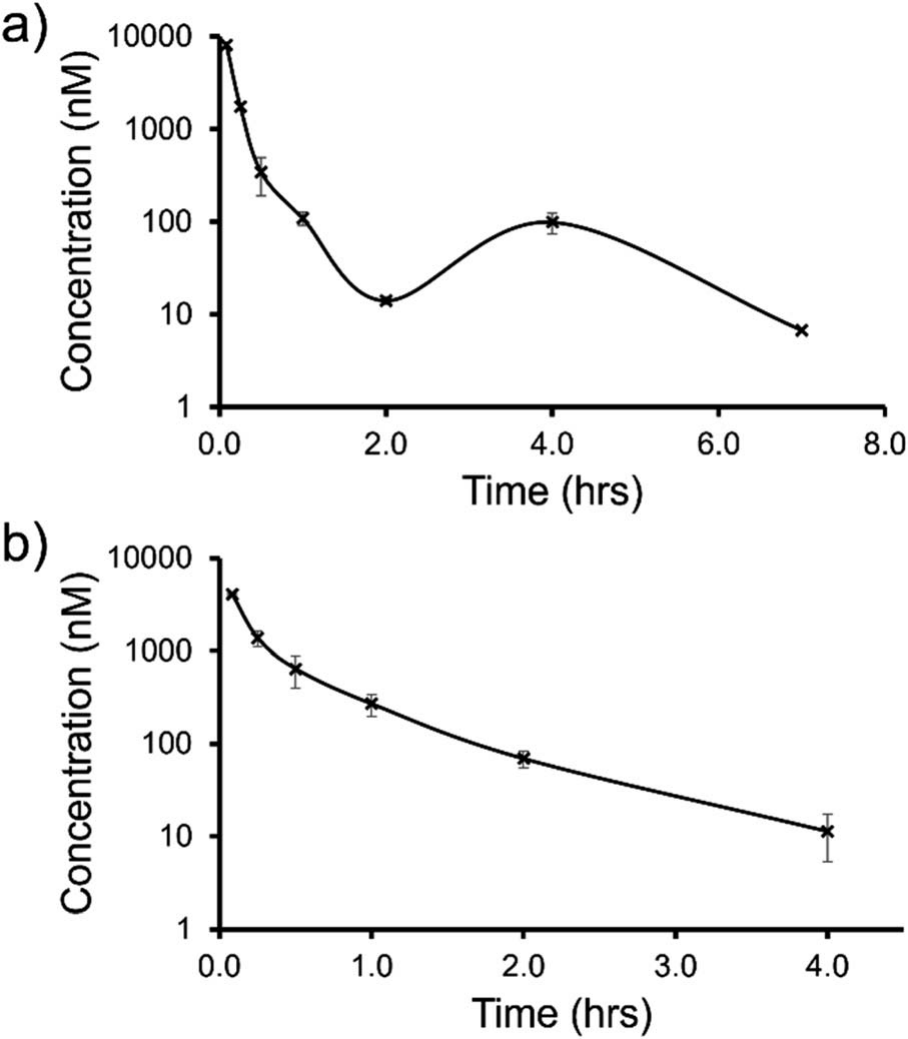
Mean blood concentration–time profile following i.v. administration of (a) SSA 3 and (b) SSA 5 at 1 mg kg^−1^ in female CD-1 mice (*n* = 3 technical repeats per time point).

Based on the results of *in vitro* DMPK studies, two SSAs were chosen to progress to mouse intravenous (i.v.) pharmacokinetic (PK) studies (Section 4, ESI).† SSA 3 was selected due to the combination of moderate Caco-2 permeability from the basolateral to the apical surface of the cell monolayer (P_app_ (B–A)), combined with low PPB values, while SSA 5 was selected as this SSA exhibited the highest P_app_ (B–A) value of those SSAs tested. Additionally, the anionic component of these two SSAs differs significantly; SSA 3 contains a trifluoromethyl substituent while SSA 5 contains a benzothiazole moiety, giving us the opportunity to observe initial structure activity relationships. Within these studies SSAs 3 and 5 were dosed at 1 mg kg^−1^, to female CD-1 mice (*n* = 9 per SSA) *via* an intravenous tail vein bolus dose formulation as solutions in 2% DMSO/98% water. Blood taken from the mouse tail vein was extracted by protein precipitation and SSA concentration calculated. The results of these studies are shown in Fig. 12 and Table 4.

**Table 4:** Summary of mouse (*n* = 3) i.v. blood PK studies. The formulation describes the solution in which the SSA was introduced to the mouse. *C*_max_ = Maximum concentration of compound observed within the blood after dosing. *T*_max_ = Time taken to reach the *C*_max_ (here *T* = 0 is the point at which a compound is introduced by i.v. route into the mouse). AUC_last_ = Area under the plasma concentration–time curve from time zero to time of last measurable concentration. AUC_INF_ = Area under the plasma concentration–time curve from time zero to infinity. Cl blood = Apparent total clearance of the drug from the blood. LBF = liver blood flow. *t*_1/2_ = elimination half-life, the time taken for the plasma concentration to fall by half its original value (calculation not possible where enterohepatic recirculation is observed). *V*_d_ = apparent volume of distribution

Study detail/result	SSA 3	SSA 5
Route	i.v.	i.v.
Dose (mg kg^−1^)	1	1
Formulation	2% DMSO/98% H_2_O	2% DMSO/98% H_2_O
*C*_max_ (nM)	8088	4046
*T*_max_ (h)	0.083	0.083
AUC_last_ (h × nM)	2277	1450
AUC_INF_ (h × nM)	NR	1460
CI blood (mL min^−1^ kg^−1^)	25^a^ (28% LBF)	31 (34% LBF)
*t*_1/2_ (h)	ND	0.7
*V*_d_ (L kg^−1^)	0.8^a^	0.9

a = Clearance and volume of distribution was derived based on AUC_0–7h_.

The trifluoromethyl substituted SSA 3 showed low blood clearance, 25 mL min^−1^ kg^−1^, 28% liver blood flow (LBF), with a moderate volume of distribution of 0.8 L kg^−1^ under test conditions.^49^ As shown in Fig. 12a, there are two *C*_max_ values for this compound, one at 0.083 h post dose and another at 4 h post dose. This is indicative of the SSA undergoing enterohepatic recirculation, a process which involves the reabsorption of the compound post initial biliary excretion thus preventing it being removed from the body, resulting in re-exposure, hence the presence of a second *C*_max_ value. An elimination half-life (*t*_1/2_) and AUC_INF_ could not be determined due to a poorly defined terminal phase resulting from SSA blood concentration levels falling to below the limit of quantification after 7 h. Blood clearance and volume of distribution were therefore derived based on area under the curve (Fig. 12a) 0–7 h (AUC_0–7h_).

However, the benzothiazole substituted SSA 5 was shown to be a low to moderate blood clearance compound (*ca.* 31 mLmin^−1^kg, 34% LBF) with a moderate volume of distribution of 0.9 L kg^−1^ and a short elimination half-life of 0.7 h. Interestingly this benzothiazole based compound did not show any evidence of enterohepatic recirculation, unlike the trifluoromethyl substituted SSA 3. At present we are unsure why enterohepatic recirculation occurs with this SSA, but possible hypotheses include direct glucuronidation or transporter interaction related events.

Finally, to gain an understanding of the propensity for SSAs 3 and 5 to diffuse from the mouse circulatory system into the mouse tissues, satellite terminal tissue sampling was also performed. The results from these studies are summarised in Table 5 and show that after 0.5 h greater concentrations of SSA 5 had disseminated into the tissues of the mice than SSA 3, however this trend was reversed after 4 h. At this time point higher concentrations of SSA 3 remained in the lung and liver tissues of the mice than were observed for SSA 5, as a result of the enterohepatic recirculation processes.

**Table 5:** Concentration (nM) of SSA found in tissue obtained from *n* = 3 mice after 0.5 h and 4 h post i.v. dosing. BLQ = below the limit of quantification. Blood concentration of SSA 3 (Fig. 12): 0.5 h = 344 nM; 4.0 h = 99 nM. Blood concentration of SSA 5 (Fig. 12): 0.5 h = 643 nM; 4.0 h = 11 nM

Tissue	SSA 3		SSA 5	
	0.5 h	4.0 h	0.5 h	4.0 h
Lung	86	18	153	BLQ
Muscle	86	BLQ	172	BLQ
Liver	1234	206	2201	429

In summary, both SSA 3 and 5 have a similar blood clearance (Cl blood = 25 *vs.* 31 mL min^−1^ kg) and volume of distribution (*V*_ss_ = 0.8 *vs.* 0.9 L kg^−1^), respectively, when administered i.v. to *n* = 9 mice at 1 mg kg^−1^. However, the absence of enterohepatic recycling observed with SSA 5 (Fig. 12b) makes this the lead candidate for further therapeutic drug development.

## Conclusions

We have determined the antimicrobial efficacy for a group of 14 structurally related SSAs and shown that changes in molecular structure does result in changes in antimicrobial activity, against both MRSA and *E. coli*. The results of HF/3-21G computational modelling showed that complexes formed between the anionic component of SSA 1 (or SSA 3) or SSA 4 exhibited a lower binding energy towards the model bacterial phospholipid headgroups, m-PE and m-PG over the model mammalian phospholipid headgroup m-PC. This supports the hypothesis that the SSA anion can selectively coordinate to phospholipids derived from bacteria (PE and PG) over those present in mammalian cells (PC), a result further supported by data collected from phospholipid membrane fluidity assays.

SSA vesicle lysis experiments have shown that in some instances SSA vesicle lysis effects are time dependent. Furthermore, the ability of an SSA to lyse a vesicle of a specific phospholipid composition is dependent on the structure of the SSA anion and probably that of the cation present. In addition, the data collected, further supported by the results of phospholipid membrane fluidity assays, suggests that when mimicking the phospholipid composition of bacterial (*E. coli*) cell membranes within synthetic vesicle studies, 10% CA should be added to the traditional PE-PG phospholipid mix.

When comparing results obtained from SSA nanodisc coordination assays with MIC_50_ values obtained from antimicrobial efficacy determination studies, and the results of vesicle assays, the contrast between these data sets leads us to suggest that enhanced levels of phospholipid SSA interaction alone may not result in sufficient membrane disruption/permeation events to elicit a therapeutic effect. Interestingly, the results of SSA nanodisc studies also suggest that the structure of the SSA anion may also drive the coordination of the SSA cation to the surface of the phospholipid bilayer.

Finally, a 7-compound subset of the 14 SSAs included within the initial vesicle leakage assays were taken forward for *in vivo* and *in vitro* DMPK analysis. The results of these studies showed that two lead SSAs 3 and 5 demonstrated a druggable profile, with SSA 5 identified as the lead SSA from the analysis of these data due to the lack of enterohepatic recirculation events.

## Data availability

We believe that most of our data is included within the ESI† associated with this article however, please contact the corresponding authors should you wish to access any additional data.

## Author contributions

JEB: investigation; validation; writing – original draft, review & editing. CB and JB: investigation; validation; writing – review & editing. KLFH: investigation; validation; writing – original draft, review & editing. HAK: investigation; validation; writing – original draft, review & editing. ERC: validation; writing – original draft, review & editing. YL: investigation; validation. LJW: investigation; writing – review & editing. HYL: investigation; validation. CKH: investigation; validation; writing – review & editing. JMS: validation; writing – review & editing. MDG: validation; writing – review & editing. AC: supervision; validation; writing – review & editing. JLOR: supervision; validation; writing – review & editing. MC: validation; writing – review & editing. CJEH: investigation; validation; writing – review & editing. JRH: conceptualization; funding acquisition; project administration; supervision; writing – original draft, review & editing.

## Conflicts of interest

There are no conflicts to declare.

## Supplementary Material

SC-013-D2SC02630A-s001
