# Supplementary material for: Establishing the selective phospholipid membrane coordination, permeation and lysis properties for a series of ‘druggable’ supramolecular self-associating antimicrobial amphiphiles
Source: Chem Sci. 2022 Aug 10;13(33):9761–73. doi: 10.1039/d2sc02630a (PMC9400670; doi:10.1039/d2sc02630a)
Supplement: SC-013-D2SC02630A-s001 [file SC-013-D2SC02630A-s001.pdf]

Electronic Supplementary Material (ESI) for Chemical Science.
This journal is © The Royal Society of Chemistry 2022

Establishing the selective phospholipid membrane coordination, permeation and lysis properties for a series of 'druggable' supramolecular self-associating antimicrobial amphiphiles†

Jessica E. Boles, Charlotte Bennett,‡ Jennifer Baker,‡ Kira L. F. Hilton, Hiral Kotak, Ewan Clark, Yifan Long, Lisa J. White, Hin Yuk Lai, Charlotte K. Hind, J. Mark Sutton, Michelle D. Garrett, Anne Cheasty, Jose L. Ortega-Roldan, Mark Charles,* Cally J. E. Haynes* and Jennifer R. Hiscock*

‡ Denotes joint authorship

Contents

Section 1: Chemical Structures 3

Section 2: General Methods 4

Section 3: Microbial Methods 5

Section 4: DMPK Methods and Further Discussion 7

Summary of DMPK in vitro results and discussion: 8

Section 5: Chemical Synthesis and Characterisation 11

Section 6: Antimicrobial Screening Data 24

USA 300 Methicillin-resistant *Staphylococcus aureus* (MRSA) 24

***Escherichia coli* (*E. coli*) DH10B** 24

Section 7: Full Growth Curve Data 26

USA 300 Methicillin-resistant *Staphylococcus aureus* (MRSA) 26

***Escherichia coli* (*E. coli*)** 28

Section 8: MIC_{50} Determination 30

USA 300 Methicillin-resistant *Staphylococcus aureus* (MRSA) 30

***Escherichia coli* (*E. coli*)** 33

Section 9: Surface Tension and Critical Micelle Concentration (CMC) Determination 36

Section 10: Vesicle Leakage Assay Data 39

Initial comparative datasets 39

PC lipid titrations 42

PG lipid titrations 45

PE:PG 3:1 lipid titrations 48

***E. coli* total lipid titrations** 50

***E. coli* polar lipid titrations** 52

Section 11: Membrane Fluidity Assay Data 57

Section 12: Fluorescence Polarisation Adhesion Assay Data 60

Section 13: Nanodisc Coordination Data 64

Nanodisc characterisation 64

Nanodisc titrations 67
Section 14: Determination of Nanodisc Coordination EC_{50} Values 79
Mechaelis Menten fits 80
Hill Plot fits 104
Section 15: Computational data 129
Binding energy calculations using ab initio and DFT modelling 129
Discussion relating to the choice of supporting molecular modelling studies 129
Solvent model comparison 135
Cartesian coordinates calculated at HF/3-21G level. 136
Cartesian coordinates calculated at M06-2X/6-31G level 165
3D visualisations – HF/3-21G level 180
3D visualisations – M06-2X/6-31G level 193
Section 16: References 201

Section 1: Chemical Structures

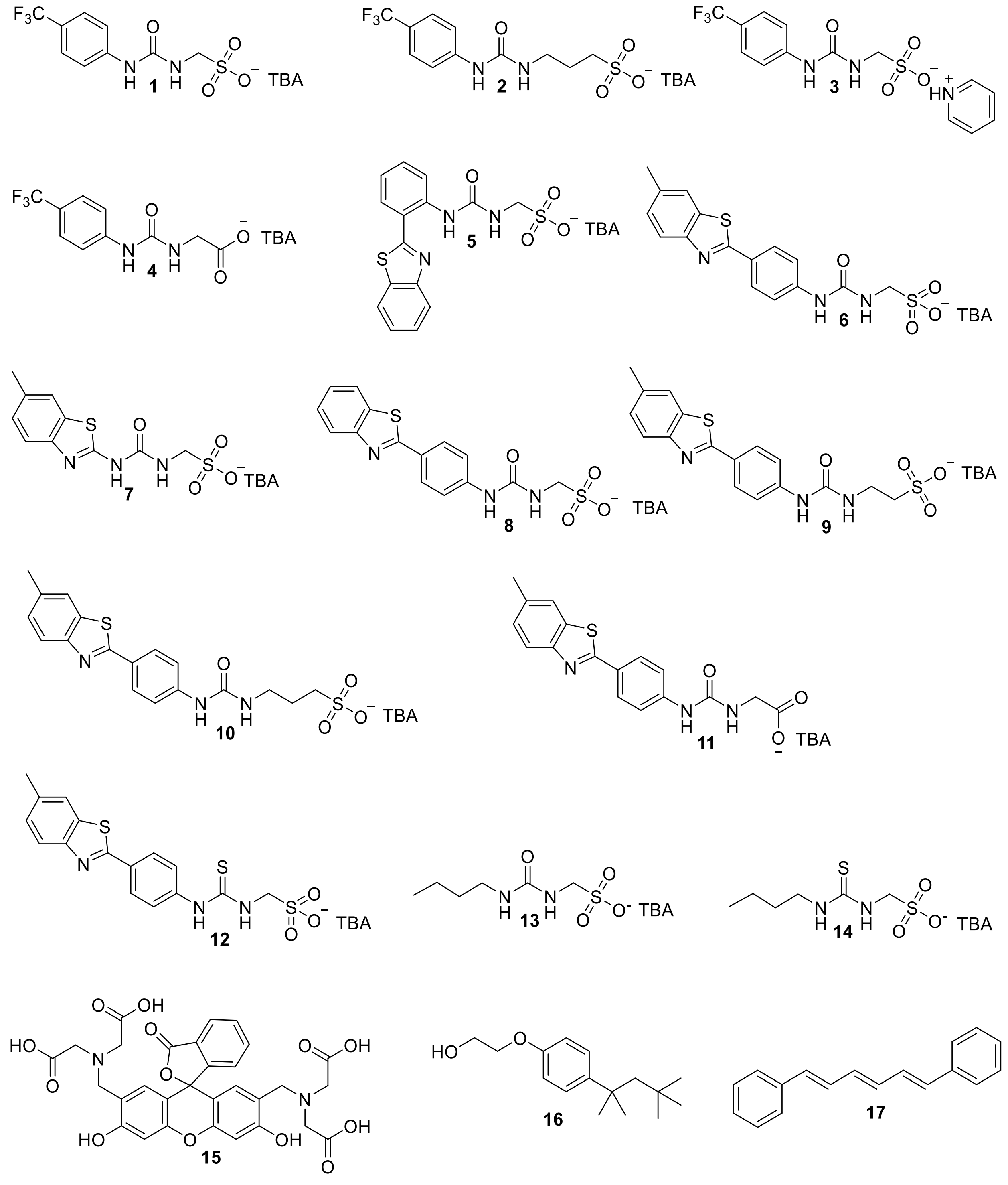

Figure S1 – Chemical structures of SSAs **1-14**, calcein, **15,** Triton X-100, **16** and 1,6-diphenyl-hexa-1,3,5-triene (DPH), **17**.

Section 2: General Methods

General remarks: A positive pressure of nitrogen and oven dried glassware were used for all reactions. All solvents and starting materials were purchased from known chemical suppliers or available stores and used without any further purification unless specifically stipulated. The NMR spectra were obtained using a Burker AV2 400 MHz, AVNEO 400 MHz or Avance III 600 Hz spectrometer. The data was processed using ACD Labs or MestreNova software. NMR Chemical shift values are reported in parts per million (ppm) and calibrated to the centre of the residual solvent peak set (s = singlet, br = broad, d = doublet, t = triplet, q = quartet, m = multiplet). Fluorescence and fluorescence polarisation measurements were conducted using a Clariostar plate reader and analysed using MARS data analysis software.

Tensiometry Studies: All the samples were prepared in an EtOH:H_2O (1:19) solution. All samples underwent an annealing process in which the various solutions were heated to approximately 40 °C before being allowed to cool to room temperature, allowing each sample to reach a thermodynamic minimum. All samples were prepared through serial dilution of the most concentrated sample. Three surface tension measurements were obtained for each sample at a given concentration, using the pendant drop method. The average values were then used to calculate the critical micelle concentration (CMC).

Preparation of vesicles: All lipids were purchased as powder stocks from Avanti Polar Lipids. Lipid vesicles were prepared according to standard procedures.[1] Each phospholipid was dissolved in chloroform (~ 10 mL) and the solvent was removed under reduced pressure. The thin film of dried lipid was further dried in vacuo for at least 2 hours. Lipid films were resuspended in the desired buffer and subjected to 9 freeze-thaw cycles in liquid nitrogen and extruded 20 times through a 200 nm polycarbonate membrane.

Preparation of calcein loaded vesicles: Lipid films were resuspended in buffer A (70 mM calcein, 10 mM TRIS pH 8.5, 0.5 mM EDTA, 110 mM NaCl, pH adjusted to 6-7) and subjected to 9 freeze-thaw cycles in liquid nitrogen and extruded 20 times through a 200 nm polycarbonate membrane. The suspension was run down a size exclusion column packed with sephadex G-50 using buffer B (10 mM TRIS pH 8.5, 0.5 mM EDTA, 110 mM NaCl). The hydrodynamic dimeter of the lipids was monitored using an Anton Paar Litesizer^{TM} 500 to ensure the separation of free and entrapped calcein. Phospholipid concentration was adjusted to 30 µM for vesicle leakage experiments.

Vesicle leakage assay: Black bottom 96-well plates were prepared with the appropriate lipid solution (100 µL, 30 µM) and desired SSA solution (100 µL) prepared in H_2O/EtOH 95: 5. A solution of 1 % Triton X-100 (**16**) was used for a 100 % lysis control and a H_2O/EtOH 95: 5 solution was used as a 0 % lysis control. Fluorescent measurements were taken at 25 °C using an excitation value of 495 nm and an optimised gain of 983. Data were acquired in endpoint mode. All experiments were repeated in triplicate to ensure experimental reproducibility.

Preparation of DPH fluorescent labelled vesicles: Lipid films were resuspended in buffer (150 mM KCl, 10 mM HEPES, pH 7.4, 2 mM EGTA) and subjected to 9 freeze-thaw cycles in liquid nitrogen and extruded 20 times through a 200 nm polycarbonate membrane. For fluorescent labelling, the desired vesicles were pre-incubated with 1,6-diphenyl-hexa-1,3,5-triene (DPH) (10 µM) at 60 °C for 1 hour.

Membrane fluidity validity assay: DPH labelled DSPG (1,2-Distearoyl-sn-glycero-3-phosphoglycerol) vesicles were prepared with a defined gel-to-liquid crystalline phase transition temperature of 55 °C. Fluorescence polarisation (FP) measurements were taken using a 355 nm filter for excitation and a 430 m for emission. The DPH labelled vesicles were set to a FP value of 100 mP. Steady state measurements were taken at 25 °C – 60 °C, the temperature on the microplate was increased in 10 °C increments until 45 °C, temperatures above this were reached externally using a heating plate. Data

were acquired in endpoint mode. All experiments were repeated in triplicate to ensure experimental reproducibility.

Membrane fluidity assay: Black bottom 96-well plates were prepared by serially diluting desired SSA in a H_2O/EtOH 95: 5 solution across the plate, the appropriate DPH labelled vesicles (100 µL, 30 µM) were added to each well to give a total well volume of 200 µL. FP measurements were taken at 25 °C using a 355 nm filter for excitation and a 430 m for emission. The DPH labelled vesicles were set to a FP value of 100 mP. Data were acquired in endpoint mode. All experiments were repeated in triplicate to ensure experimental reproducibility.

Fluorescence polarisation: Lipid films were resuspended in Buffer (150 mM KCl, 10 mM HEPES, pH 7.4, 2 mM EGTA) and subjected to 9 freeze-thaw cycles in liquid nitrogen and extruded 20 times through a 200 nm polycarbonate membrane. Black bottom 96-well plates were prepared by serially diluting desired vesicle solution across the plate, the appropriate SSA solution (100 µL, 0.15 mM) was added to each well to give a total well volume of 200 µL. Fluorescent SSA solutions were set to a FP value of 100 mP. Data were acquired in endpoint mode. All experiments were repeated in triplicate to ensure experimental reproducibility.

Nanodisc preparation: Lipid films were resuspended in Buffer (20 mM NaCl, 20 mM NaH_2PO_4, pH 7.4) and sonicated for 1 hour, at a 5:1 ratio, SMA was added and incubated at 37 °C for 1 hour. Dialysed overnight in 5 L of the same buffer in 10 KDa cut-off dialysis tubing, concentrated, and underwent gel filtration size exclusion chromatography on a Superdex 200 10/300 GL column (GE Healthcare) in the same buffer, whilst monitoring absorbance at 260 nm. Nanodisc quantification carried out via monitoring of absorbance at 260 nm using a calibration curve.[2]

^{1}H NMR CPMG: ^{1}H NMR CPMG[3,4] spectra were obtained with a Bruker Avance III 600 Hz spectrometer equipped with a TCIP cryoprobe at 298 K. Samples were supplemented with 5 % D_2O and 0.02 mM 4,4-dimethyl4-silapentane-1-sulfonic acid (DSS). The standard zgprcpmg pulse sequence from the Bruker library was modified with a watergate element to allow suppression of the water signal and was used for all experiments. Although this sequence is readily obtainable, we are happy to provide it on request. The P1 pulses were 9 µs at a power of 7.9 W. Presaturation was applied between acquisitions for 100 ms at a power level of 280 mW and acquisition was carried out of 852 µs. Water suppression was achieved with a 3-9-19 watergate sequence using 1 ms pulses with a smothered chirp shape with a peak strength of 21.2 G. The CPMG element had a length of 300 ms with delays between 180 ° pulses of 1 ms. Repetition times were chosen to achieve suitable water suppression with the presat and watergate sequences used in 95 % H_2O solution. The length of the CPMG element was chosen to achieve good differentiation between unbound and strongly bound ligands. Data was collected with 16,384 points and a spectral width of 16.0242 ppm, receiver gain was set to 128, with 512 scans, 8 dummy scans and an acquisition time of 0.85 s. Data was processed using MestreNova software. All spectra were automatically phased, baselined corrected using a polynomial function and calibrated to the centre of the DSS peak.

Fitting of ^{1}H NMR CPMG data: The data was fit to Michaelis Menten and Hill plot models and compared through R^2 analysis using Origin 2022 software,[5] growth/sigmoidal category, Hill/MichaelisMenten function, Levenberg Marquardt iteration algorithm. V_{max} fixed to 100 % as this was the greatest proportion of SSA that could be coordinated to the nanodiscs. See Tables S4 and 5.

Section 3: Microbial Methods

These methods were taken directly from UK Health and Security Office (UKHSA) standard operating procedures.

Preparation of Luria Broth media (LB): Yeast extract (5 g), tryptone (10 g) and sodium chloride (10 g) were dissolved in dH_2O (1 L) then divided into bottles and autoclaved.

Preparation of Luria Broth (LB) agar plates: Agar (6 g) was added to LB (400 mL) and autoclaved. Once cool, the LB agar was poured into sterile petri dishes under sterile conditions and allowed to set. LB plates were stored at 4 °C until use.

Preparation of bacterial plates: Sterile LB agar plates were streaked using *Escherichia coli* DH10B or USA300 Methicillin-Resistant *Staphylococcus aureus* then incubated at 37 °C overnight.

Preparation of Inoculum: An initial culture was made up by inoculating LB media (5 mL) with a single colony of bacteria under sterile conditions and incubated at 37 °C with shaking overnight. The following day, overnight bacterial cultures were subcultured into fresh LB medium to a starting OD_{600} of 0.01.

Preparation of 96 well microplate for screening: 20 mM solutions of each compound to be tested were made up using 5 % ethanol. The 1:100 cell suspension (150 µL) was pipetted into the wells and SSA compound solutions (30 µL) were added into each well. The final screening concentration for each compound was 3.3 mM in the well. These were incubated for 20 hours in a plate reader, with optical density readings being taken at 600 nm every 15 minutes. Optical density readings were plotted against time to produce growth curves.

Preparation of 96 well microplate for MIC_{50}: The 1:100 cell suspension (150 µL) was dispensed into individual wells under sterile conditions. Compounds (30 µL) were added to the wells to equal a total volume of 180 µL in the wells. Six repeats of each concentration for each compound were created each plate. The plates were sealed using parafilm, then incubated at 37°C in a microplate reader for 18-25 hours. Optical density readings were taken at 600 nm every 15 min. Data was used to generate growth curves.

Calculation of MIC_{50}: Growth curves were plotted using OD_{600} optical density readings in Microsoft ® Excel ® 2013. OD_{600} optical density readings 900 minutes from the start of each of these growth cultures for each concentration of compound were plotted in Origin ® 2015. The resultant curve was normalized and fitted using the Boltzmann fit to define the MIC_{50} values for each compound.

Section 4: DMPK Methods and Further Discussion

Materials used for DMPK studies: Sprague Dawley rat liver microsomes (male, 771 donors, Lot 1910100), CD-1 mouse liver microsomes (male, 6424 donors, Lot 2010017) and human liver microsomes (mixed gender,50 donors, Lot 1810003) were purchased from Sekisui Xenotech (Kansas City, USA).

Microsomal incubations: SSA **1**-**6** and **10** (final concentration 1 µM) were pre-incubated for 10 min in mouse, rat, or human liver microsomes (final concentration 0.5 mg/mL prepared in 100 mM potassium phosphate buffered saline (PBS)) at 37°C. The reaction was initiated with the addition of the cofactor, NADPH-generating system. The NADPH-generating system consisted of NADP (1.3 mM, pH 7.4), glucose-6-phosphate (3.3 mM, pH 7.4), glucose-6-phosphate dehydrogenase (0.4 unit/mL) and $MgCl_2$ (3.3 mM). The reaction was allowed to proceed for 45 min. At 0, 5, 10, 15, 30, and 45 min aliquots were sampled and quenched in ice cold acetonitrile with internal standard. Incubations were conducted in singlicate; control incubations (substituting PBS for NADPH) were conducted simultaneously. All control incubations were within acceptance criteria in mouse, rat, and human microsomes confirming stability was acceptable over 45 minutes in the absence of co-factor.

Protein Binding studies: The fraction unbound in mouse plasma (BioIVT, Frankfurt Am Main, Germany) of SSA **1**-**6** and **10** were measured using a Rapid Equilibrium Dialysis device (Thermo Fisher Scientific, Loughborough, UK). Mouse plasma containing 2 µM of SSA **1**-**6** and **10** were dialysed against 100 mM PBS for 4h at 37°C at 5% CO_2. Samples were removed, matrix matched and quenched with acetonitrile containing internal standard; further processing is described in: **Sample preparation and liquid chromatography-mass spectrometry analysis**.

Caco-2 Permeability studies: The permeability assay for SSA **1**-**6** and **10** was performed at Cyprotex Discovery Limited (Macclesfield, UK) and analysed at Cancer Research Horizons. SSA **1**-**6** and **10** were incubated at a final concentration of 10 uM with human intestinal Caco-2 cells at 37°C, 5% CO2 with a relative humidity of 95%. After 120 minutes the apical compartment inserts and the companion plates were separated, and the apical and basolateral samples were transferred. Incubations were conducted in duplicate and the fluorescent membrane integrity marker; Lucifer Yellow was co-incubated with the SSA compounds at the start of the assay. Compounds of known permeability were used as controls and were within acceptance criteria. Further processing is described in: **Sample preparation and liquid chromatography-mass spectrometry analysis**.

Sample preparation and liquid chromatography-mass spectrometry analysis: All samples were centrifuged at 3500 rpm at 4°C for 10 min to precipitate protein and enable sampling of supernatant prior to appropriate dilution for liquid chromatography-mass spectrometry (LC-MS/MS analysis).

For the purpose of CL_{INT} determination, samples were analysed using a Waters I-Class ultra-high-performance liquid chromatography (UHPLC) system coupled to a Waters Xevo TQ-XS mass spectrometer (Waters, Elstree, UK). Separation of analytes was achieved using an Accucore Vanquish C18+ column (1.5 µm, 50 x 2.1 mm) (Thermo Fisher Scientific, Hemel Hempstead) at a temperature of 40°C and a binary mobile phase gradient at a flow rate of 0.4 mL/min. Initial LC conditions comprised 95% solvent A (0.1% formic acid + 5 mM ammonium acetate in water), 5% solvent B (0.1% formic acid + 5 mM ammonium acetate in acetonitrile); this was ramped to 90% B at 5 min, and then immediately returned to initial conditions and held for the remaining 0.5 min of the method. Sample analysis was by electrospray ionisation combined with multiple reaction monitoring in negative ion mode. The capillary voltage was 0.8 kV, desolvation gas was 600°C.

For the purpose of fu and SSA-**3** PK determination, samples were analysed using a Waters I-Class ultra-high-performance liquid chromatography (UHPLC) system coupled to a Waters Xevo TQ-XS mass spectrometer. Separation of analytes was achieved using an Accucore Vanquish C18+ column (1.5 µm, 50 x 2.1 mm) at a temperature of 40°C and a binary mobile phase gradient at a flow rate of 0.4 mL/min. Initial LC conditions comprised 66% solvent A (0.1% formic acid + 5 mM ammonium acetate in water), 33% solvent B (0.1% formic acid + 5 mM ammonium acetate in methanol); this was ramped to 63% B at 1.8 min and 95% B at 1.9 min, held for 0.5 min and then immediately returned to initial conditions and held for the remaining 0.5 min of the method. Sample analysis was by electrospray ionisation combined with multiple reaction monitoring in negative ion mode. The capillary voltage was 0.8 kV, desolvation gas was 600°C.

For the purpose of apparent permeability determination, samples were analysed using a Waters I-Class ultra-high-performance liquid chromatography (UHPLC) system coupled to a Waters Vion mass spectrometer (Waters, Elstree, UK). Separation of analytes was achieved used an Accucore Vanquish C18+ column (1.5 µm, 50 x 2.1 mm) at a temperature of 40°C and a binary mobile phase gradient at a flow rate of 0.4 mL/min. Initial LC conditions for SSA **1**-**4** comprised 66% solvent A (0.1% formic acid + 5 mM ammonium acetate in water), 33% solvent B (0.1% formic acid + 5 mM ammonium acetate in methanol); this was ramped to 63% B at 1.8 min and 95% B at 1.9 min, held for 0.5 min and then immediately returned to initial conditions and held for the remaining 0.5 min of the method. Initial LC conditions for SSA **5**-**10** comprised 54% solvent A (0.1% formic acid + 5 mM ammonium acetate in water), 46% solvent B (0.1% formic acid + 5 mM ammonium acetate in methanol); this was ramped to 67% B at 1.8 min and 95% B at 1.9 min, held for 0.5 min and then immediately returned to initial conditions and held for the remaining 0.5 min of the method. Sample analysis was by electrospray ionisation in negative ion mode. The capillary voltage was 2.5 kV, desolvation gas was 600°C.

For the purpose of PK determination of SSA **5**, samples were analysed using a Waters I-Class ultra-high-performance liquid chromatography (UHPLC) system coupled to a Waters Xevo TQ-XS mass spectrometer. Separation of analytes was achieved using an Accucore Vanquish C18+ column (1.5 µm, 50 x 2.1 mm) at a temperature of 40°C and a binary mobile phase gradient at a flow rate of 0.4 mL/min. Initial LC conditions comprised 54% solvent A (0.1% formic acid + 5 mM ammonium acetate in water), 46% solvent B (0.1% formic acid + 5 mM ammonium acetate in methanol); this was ramped to 67% B at 1.8 min and 95% B at 1.9 min, held for 0.5 min and then immediately returned to initial conditions and held for the remaining 0.5 min of the method. Sample analysis was by electrospray ionisation combined with multiple reaction monitoring in negative ion mode. The capillary voltage was 0.8 kV, desolvation gas was 600°C.

Mean blood concentration-time profile: Within these studies SSAs **3** and **5** were dosed at 1 mg/kg, to female CD-1 mice (n=9 per SSA) via an intravenous tail vein bolus dose formulation as solutions in 2 % DMSO/98 % water. Blood taken from the mouse tail vein was extracted by protein precipitation. SSA concentrations were calculated through UPLC-MS/MS techniques, with tolbutamide as the internal standard. The assay met the acceptance criteria with >7 calibration standards within ± 20% of nominal concentration. The lower limit of quantitation was 2 ng/mL for both compounds. PK parameters were calculated using Phoenix WinNonLin 8.1.0.3530. The results of these studies are shown in Figure 12 and Table 4 – main manuscript.

Summary of DMPK in vitro results and discussion:

We first determined the in vitro metabolic stability of these SSAs against mouse, rat, and human liver microsomes. These microsomes contain membrane bound enzymes, such as cytochrome p450,

which are responsible for first pass metabolism effects, to which a potential therapeutic agent must exhibit some resilience. Within this assay the stability of the intact parent compound is monitored with respect to time, during incubation at 37 °C in the presence of the co-factor NADPH – required for cytochrome p450 activity. From these data, the intrinsic clearance (Cl_{int}) of the compound can be calculated. The lower this value, the more stable the compound is to the actions of these membrane bound metabolic enzymes. This high throughput assay represents a relatively low-cost route to enable the ranking of many compounds with respect to metabolic stability. The results of this assay showed that all of those SSAs tested demonstrated a Cl_{int} < 0.647 mL/min/g liver across this panel of microsomes, considered low by industrial standards.

Mouse plasma protein binding (PPB) assays were performed to estimate the ability of an SSA to distribute into the tissues of the body, as the extent to which a compound binds to the constituents of blood plasma will influence drug distribution. For example, protein binding will prevent a compound diffusing from the circulatory system into tissues to reach a cellular site of targeted action. In addition, these interactions also affect compound metabolism and elimination rates. The results of the PPB assays are summarised in Table S1. Here the fraction of SSA which remains unbound to protein under these assay conditions, a value known as the fu, is calculated, and used to derive the percentage of SSA which undergoes protein plasma binding (PPB), Equation S1.

$$\mathbf{PPB\ (\%) = (1\text{-}fu) \times 100}$$

Equation S1 - Calculation of percentage of SSA that remains undergoes PPB, fu.

The percentage of SSA recovered during the analysis process should be 100%; any deviation from this value indicates experimental limitations such as unintended potential non-specific binding events to experimental equipment, solubility issues, etc.. As part of the PPB assay, plasma stability was assessed. This reports the % of an SSA, supplied under these experimental conditions, to remain intact over a 4-hour time period. This is also important as those compounds which are found to degrade rapidly within plasma are generally known to exhibit poor in vivo efficacy. In addition, this instability can lead to false in vivo data interpretation.

Table S1 - Summary of data collected from n = 4 technical repeat mouse plasma protein binding assays. PPB = % of SSA bound to the plasma proteins after 4 hours incubation at 37 °C. Low PPB = < 80 %; Moderate PPB = 80-95 %; High PPB = 95-99 %; Very high PPB = > 99 %. Recovery = % SSA recovered from the assay after 4 hours.

SSA	PPB	Recovery	Plasma stability
**1**	75.0	91	94
**2**	83.4	79	93
**3**	73.8	80	89
**4**	67.2	86	96
**5**	> 99.7	109	92
**6**	99.3	101	90
**10**	99.3	94	89

All SSAs tested exhibited high plasma stability and acceptable % recoverability values to validate the % PPB values obtained. SSAs **1**, **3** and **4** were all found to exhibit low PPB values. All of these SSAs contain a trifluoromethyl functionality, a TBA counter cation and a methylene urea-anion linker. In

addition, replacement of the trifluoromethyl group by the benzothiazole substituent (SSAs **5**, **6** and **10**) significantly raises the PPB from low/moderate to very high which, we believe, is due to the presence of the extended aromatic ring systems supporting more favourable SSA:protein π-π stacking/hydrophobic interactions within the mouse plasma.

To gauge the suitability of SSAs for oral dosing, the seven SSAs were further analysed using a Caco-2 permeability assay, the results of which are summarised in Table S2. This in vitro assay is used to both predict human intestinal permeability and investigate drug efflux, predicting in vivo compound absorption. Caco-2 cells have characteristics that cause them to resemble intestinal epithelial cells as they form a polarised monolayer with a well-defined brush border on the outward facing (apical) cell surface and at the intercellular junctions. This assay allows assessment of a compound's ability to undergo transport from the apical to the basolateral (A-B) surface of the cell monolayer and *vice versa*.

None of the SSAs were found to permeate the monolayer in the A-B direction due to efflux, however low to high SSA permeation was observed in the B-A direction, which was broadly found to correlate with calculated clogD values obtained using StarDrop software (Version: 7.1.2.31725). High permeability coefficient (P_{app}) values were obtained for SSAs **5** and **6**, which both contain a benzothiazole functionality. However, moving this functionality from the meta to the para position on the SSA anion benzene ring system was found to decrease the P_{app} by 48 %, possibly due to the loss of intramolecular urea:benzothiazole hydrogen bonding[38] and exposure of the polar urea functionality of the monomeric unit to the wider biological environment. Increasing the length of the urea-sulfonate alkyl spacer from a methylene group (SSA **6**) to a propylene group (SSA **10**) was found to further decrease SSA permeability. This trend was also observed for the trifluoromethyl substituted SSAs **1** and **2**. Interestingly, further comparison of the P_{app}(B-A) values obtained for SSAs **1**-**4** showed the permeation properties of SSAs with the same anion, but different cation to be similar (SSAs **1** and **3**), while replacing the sulfonate group for the carboxylate anion was also found to decrease SSA permeability (SSAs **1** and **4**). This is perhaps expected due to the increased charge density, and presumably decrease in lipophobicity, observed for the carboxylate over the sulfonate moiety.

As mentioned, compound recovery was also determined to enable assay validation; here the % recovery of SSAs **6** and **10** from the Caco-2 assay was low (P_{app} (A-B)), potentially due to non-specific SSA binding to the apical layer. However, as the P_{app} calculated for these assays was found to be 0.0 x 10^{-6} cm/s, this low percentage recovery is not problematic. In addition, for all SSAs tested, efflux ratios were not quantified due to the low P_{app} (A-B). However, a high rate of active efflux can be considered due to the way in which the efflux ratio is calculated (efflux ratio = P_{app}(B-A)/P_{app}(A-B)). As the results of these studies show these SSAs to exhibit a high proportion of compound efflux, meaning that at present these compounds are likely to suffer issues moving from the intestinal lumen to circulation (apical to basolateral layer), this suggests that these compounds would favour intra venous (i.v.) administration.

Table S2 - Summary of results from Caco-2 permeability assays (n = 2 technical repeats) for SSAs **1-6** and **10**. Here the P_{app} is given as $x10^{-6}$ cm/s. Low P_{app} = < 5 $x10^{-6}$ cm/s; Moderate P_{app} = 5-10 $x10^{-6}$ cm/s; High P_{app} = > 10 $x10^{-6}$ cm/s. A = apical; B = basolateral; N/A = not applicable. Recovery = % SSA recovered from the assay after 2 hours. clogD values were calculated using StarDrop at pH 7.4, here a value between 1 and 3 is considered good for permeability and hence oral bioavailability.

SSA	P_{app} (A-B)	Recovery	P_{app} (B-A)	Recovery	Efflux ratio	clogD
**1**	0.0	93	5.4	101	N/A	*a*
**2**	0.0	97	1.7	102	N/A	0.1652
**3**	0.0	99	5.6	98	N/A	*a*
**4**	0.0	93	2.3	96	N/A	*a*
**5**	0.0	79	21.0	107	N/A	0.4737
**6**	0.0	59	11.0	86	N/A	0.5840
**10**	0.0	54	6.7	95	N/A	0.9826

a - negative cLogD value calculated, therefore not reported.

Section 5: Chemical Synthesis and Characterisation

Compound 1: This compound was synthesised in line with our previously published methods. Proton NMR were found to match our previously published. [6]

Compound 2: This compound was synthesised in line with our previously published methods. Proton NMR were found to match our previously published. [7]

Compound 3: This compound was synthesised in line with our previously published methods. Proton NMR were found to match our previously published. [8]

Compound 4: This compound was synthesised in line with our previously published methods. Proton NMR were found to match our previously published. [7]

Compound 5: This compound was synthesised in line with our previously published methods. Proton NMR were found to match our previously published. [9]

Compound 6: This compound was synthesised in line with our previously published methods. Proton NMR were found to match our previously published. [9]

Compound 7: Aminomethansulfonic acid (0.22 g**,** 2.00 mmol) was dissolved in tetrabutylammonium in methanol (2.00 mL, 2 mmol) with excess methanol (15 mL) to aid dissolving before being taken to complete dryness. A mixture of 2-Aminobenzothiazole (0.33 g, 2.00 mmol) and 1,1′-Carbonyldiimidazole (0.33 g, 2.00 mmol) were heated at reflux at 80 °C for 4 hours in chloroform (20 mL). Tetrabutylammonium aminomethanesulfonate (2.00 mmol) was dissolved in chloroform (2.00 mL) and added to the original reaction mixture and heated at reflux at 80 °C overnight. The solvent was removed and the resulting oil dissolved in ethyl acetate (15 mL), producing the pure product as a white solid with a yield of 91 % (0.99 g, 1.82 mmol); Melting point: > 200 °C; ^{1}H NMR (400 MHz, 298 K, DMSO-d_6): δ: 0.93 (t, *J* = 7.24 Hz, 12H), 1.25 – 1.35 (m, 8H), 1.52 – 1.59 (m, 8H), 2.37 (s, 3H), 3.13 – 3.17 (m, 8H), 3.91 (d, *J* = 5.88 Hz, 2H), 7.11 – 7.17 (m, 2H), 7.50 (d, *J* = 8.16 Hz, H), 7.67 (s, 1H), 10.52 (s, 1H); ^{13}C{^{1}H} NMR (100 MHz, 298K, DMSO-d_6): δ: 13.6, (CH_3), 19.3 (CH_2), 21.0 (CH_3), 23.1 (CH_2), 55.9

(CH_2), 57.5 (CH_2), 119.5 (ArCH), 121.1 (ArCH), 127.0 (ArCH), 131.7 (ArC), 132.0 (ArC), 147.1 (ArC), 153.3 (ArC), 158.7 (ArC); IR (film): ν (cm^{-1}) = 3345 (NH stretch), 1697 (C=O stretch), 1038 (CN stretch); HRMS for the sulfonate-urea ion ($C_{10}H_{10}N_3O_4S_2^-$) (ESI^-): m/z: act: 300.0133 [M] cal: 300.0118 [M].

Compound 8: Aminomethansulfonic acid (0.11 g**,** 2 mmol) was dissolved in tetrabutylammonium in methanol (2.00 mL, 2.00 mmol) with excess methanol to aid dissolving before being taken to complete dryness. A mixture of 4-(benzothiazol-2-yl)aniline (0.45 g, 2.00 mM) and triphosgene (0.30 g, 1 mmol) was heated at reflux for 4 hours in ethyl acetate (20 mL). Tetrabutylammonium aminomethanesulfonate (2.00 mmol) was dissolved in ethyl acetate (2.00 mL) and added to the original reaction mixture and heated at reflux overnight. The crude precipitate was the clean product with a yield of 67 % (0.81 g, 1.34 mmol); melting point: 195 °C; ^1H NMR (400MHz, 298K, DMSO-d_6): δ: 0.93 (t, *J* = 7.32, 12H), 1.26 – 1.35 (m, 8H), 1.52 – 1.60 (m, 8H), 3.13 – 3.17 (m, 8H), 3.90 (d, *J* = 5.68 Hz, 2H), 6.65 (s, 1H), 7.41 (t, *J* = 7.76 Hz, 1H), 7.51 (t, *J* = 7.84 Hz, 1H), 7.57 (d, *J* = 8.44 Hz, 2H), 7.95 – 8.00 (m, 3H), 8.09 (d, *J* = 7.92 Hz, 1H), 9.18 (s, 1H); $^{13}C\{^1H\}$ NMR (100 MHz, 298 K, DMSO-d_6): δ: 13.6 (CH_3), 19.2 (CH_2), 23.1 (CH_2), 56.0 (CH_2), 57.5 (CH_2), 117.6 (ArCH), 122.2 (ArCH), 122.4 (ArCH), 125.0 (ArCH), 125.4 (ArC), 126.5 (ArCH), 128.0 (ArCH), 134.2 (ArC), 143.7 (ArC), 153.7 (ArC), 154.2 (ArC), 167.4 (C=O); IR (film): ν (cm^{-1}) = 3267 (NH stretch), 1697 (C=O stretch), 1327 (S=O stretch), 1038 (CN stretch); HRMS for the sulfonate-urea ion ($C_{15}H_{12}N_3O_4S_2^-$) (ESI^-): m/z: act: 390.2425 [M + C_2H_4] cal: 362.0275 [M].

Compound 9: Aminomethansulfonic acid (0.11 g**,** 2.00 mmol) was dissolved in tetrabutylammonium in methanol (2.00 mL, 2 mmol) with excess methanol (15 mL) to aid dissolving before being taken to complete dryness. A mixture of 4-(benzothiazol-2-yl)aniline (0.48 g, 2 mmol) and triphosgene (0.30 g, 1 mmol) was heated at reflux for 4 hours in ethyl acetate (20 mL). Tetrabutylammonium aminomethanesulfonate (2 mM) was dissolved in ethyl acetate (2.00 mL) and added to the original reaction mixture and heated at reflux overnight. The crude precipitate was the clean product with a yield of 64 % (0.81 g, 1.28 mmol); melting point: 195 °C; ^1H NMR (400MHz, 298K, DMSO-d_6): δ: 0.93 (t, J = 7.36, 12H), 1.26 – 1.36 (m, 8H), 1.53 – 1.60 (m, 8H), 2.45 (s, 3H), 2.57 (t, *J* = 6.04 Hz, 2H), 3.14 – 3.18 (m, 8H), 3.39 (q, *J* = 6.32 Hz, 2H), 6.51 (t, *J* = 5.28 Hz, 1H), 7.32 (d, *J* = 8.04 Hz, 1H), 7.59 (d, *J* = 8.72 Hz, 2H), 7.85 – 7.92 (m, 4H), 9.30 (s, 1H); $^{13}C\{^1H\}$ NMR (100 MHz, 298 K, DMSO-d_6): δ: 13.4 (CH_3), 19.1 (CH_2), 21.0 (CH_3), 23.0 (CH_2), 36.1 (CH_2), 50.9 (CH_2), 57.5 (CH_2), 117.5 (ArCH), 121.6 (ArCH), 121.9 (ArCH), 125.3 (ArC), 127.7 (ArCH), 127.8 (ArCH), 134.2 (ArC), 134.6 (ArC), 143.8 (ArC), 151.8 (ArC), 154.7 (ArC), 166.2 (C=O); IR (film): ν (cm^{-1}) = 3267 (NH stretch), 1697 (C=O stretch), 1327 (S=O stretch), 1038 (CN stretch); HRMS for the sulfonate-urea ion ($C_{15}H_{12}N_3O_4S_2^-$) (ESI^-): m/z: act: 390.0575 [M] cal: 390.0588 [M].

Compound 10: This compound was synthesised in line with our previously published methods. Proton NMR were found to match our previously published. [10]

Compound 11: A mixture of 4-(6-methylbenzothiazole-2-yl)aniline (0.48 g, 2.00 mmol) and triphosgene (0.30 g, 1.00 mmol) were heated at reflux for 4 hours in ethyl acetate (10.00 mL), then tert-butyl 2-amino-acetate (0.26 g, 2.00 mmol) was added to the original reaction mixture and then heated at reflux overnight. The crude precipitate was removed. The precipitate was dissolved in chloroform (5.00 mL), triflouroacetic acid (1.50 mL) added and the resulting solution left to stir at RT for 30 minutes. NaOH (6 M) was then added dropwise, testing pH until neutral and the deprotected product precipitates out. Dissolve product in methanol (10 mL) and add tetrabutylammonium hydroxide in excess (2.00 mL, 2.00 mmol). Remove precipitate and take the filtrate to dryness and titrate with water. Filter to produce pure product as a white solid with a yield of 27 % (0.23 g, 0.39 mmol), Melting point: 86 °C; ^1H NMR (400MHz, 298K, DMSO-d_6): δ: 0.92 (t, *J* = 7.28, 12H), 1.25 – 1.34 (m, 8H), 1.51 – 1.58 (m, 8H), 2.44 (s, 3H), 3.12 – 3.16 (m, 8H), 3.39 (d, *J* = 3.48 Hz, 2H), 6.92 (s, 1H),

7.31 (d, J = 8.52 Hz, 1H), 7.72 – 7.90 (m, 6H), 10.49 (s, 1H); $^{13}C\{^1H\}$ NMR (100 MHz, 298 K, DMSO-d_6): δ: 13.5 (CH_3), 19.2 (CH_2), 21.0 (CH_3), 23.1 (CH_2), 45.0 (CH_2), 57.5 (CH_2), 117.1 (ArCH), 121.7 (ArCH), 121.9 (ArCH), 124.6 (ArC), 127.7 (ArCH), 127.8 (ArCH), 134.2 (ArC), 134.5 (ArC), 144.8 (ArC), 151.9 (ArC), 154.8 (ArC), 166.5 (C=O), 170.4 (C=O); IR (film): ν (cm^{-1}) 3331 (NH stretch), 2961 (OH stretch), 1699 (C=O stretch), 1331 (S=0 stretch), 1177 (CN stretch); HRMS for the sulfonate-urea ion ($C_{17}H_{14}N_3O_3S^-$) (ESI$^-$): m/z: act 340.2244 [M], cal: 340.0761 [M].

Compound 12: A mixture of 4-(6-methylbenzothiazole-2-yl)aniline (0.48 g, 2.00 mmol) and thiophosgene (0.15 mL, 2 mmol) as a two phase solution of dichloromethane (20 mL) and saturated sodium hydrogen carbonate (20 mL) was stirred for 4 hours at room temp, then transferred to separation funnel, the organic layer was separated and taken to dryness. Then it was re-dissolved in ethyl acetate (30 mL) and tetrabutylammonium aminomethanesulfonate (2.00 mmol) was added and the solution left to reflux overnight. The ethyl acetate was decanted out, the oil taken to dryness, re-dissolved in methanol (5 mL) and ran down an ion exchange column. Ammonia was used to remove the product, with 50 % loss of tetrabutylammonium. The product was then re-dissolved in methanol (10 mL) and excess tetrabutylammonium (2 mL, 2 mmol) was then added and taken to dryness. The oil was re-dissolved in chloroform (30 mL) and water washed (30 mL). The organic layer was taken to dryness leaving a yellow solid as the clean product with a yield of 28 % (0.35 g, 0.55 mmol); Melting point: >200 °C; ^1H NMR (400MHz, 298K, DMSO-d_6): δ: 0.93 (t, J = 7.28, 12H), 1.26 – 1.35 (m, 8H), 1.52 – 1.60 (m, 8H), 2.46 (s, 3H), 3.13 – 3.18 (m, 8H), 4.09 (s, 0.6H), 4.32 (s 1.4H), 7.34 (dd, J = 8.44, 1.48 Hz, 1H), 7.70 – 8.00 (m, 6.7H), 8.71 (s, 0.3H), 10.21 (s, 0.6H), 11.05 (s, 0.2H); $^{13}C\{^1H\}$ NMR (100 MHz, 298 K, DMSO-d_6): δ: 13.5 (CH_3), 19.2 (CH_2), 21.1 (CH_3), 23.1 (CH_2), 57.5 (CH_2), 60.2 (CH_2), 121.7 (ArCH), 121.8 (ArCH), 122.2 (ArCH), 127.3 (ArCH), 127.8 (ArCH), 128.0 (ArC), 134.5 (ArC), 135.0 (ArC), 142.6 (ArC), 151.8 (ArC), 165.9 (ArC), 179.9 (C=O); IR (film): ν (cm^{-1}) 3242 (NH stretch), 1325 (S=O stretch), 1223 (CN stretch), 1036 (S=O stretch); HRMS for the sulfonate-urea ion ($C_{16}H_{14}N_3O_3S_3^-$) (ESI$^-$): m/z: act: 391.1053 [M$^-$], cal: 392.0203 [M].

Compound 13: This compound was synthesised in line with our previously published methods. Proton NMR were found to match our previously published. [11]

Compound 14: This compound was synthesised in line with our previously published methods. Proton NMR were found to match our previously published.[11]

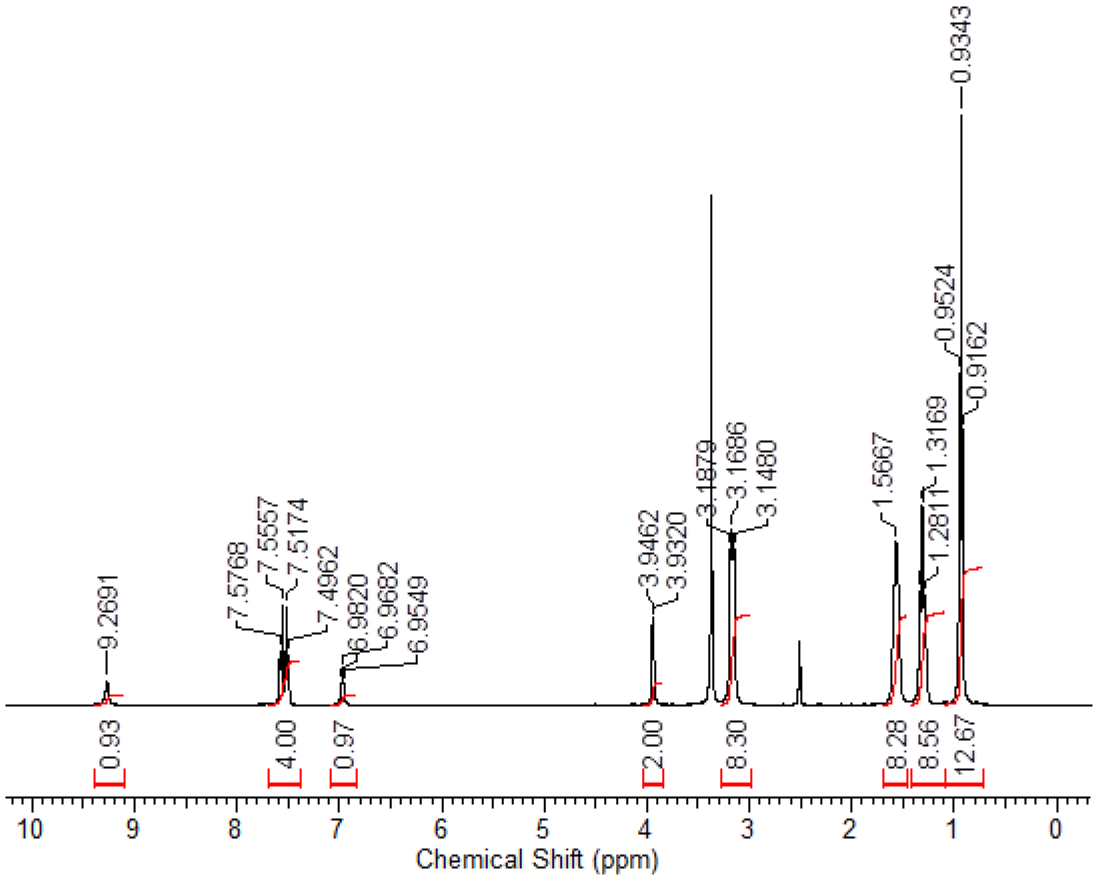

Figure S2 - ^{1}H NMR of compound **1** in DMSO-d_6 conducted at 298.15 K.

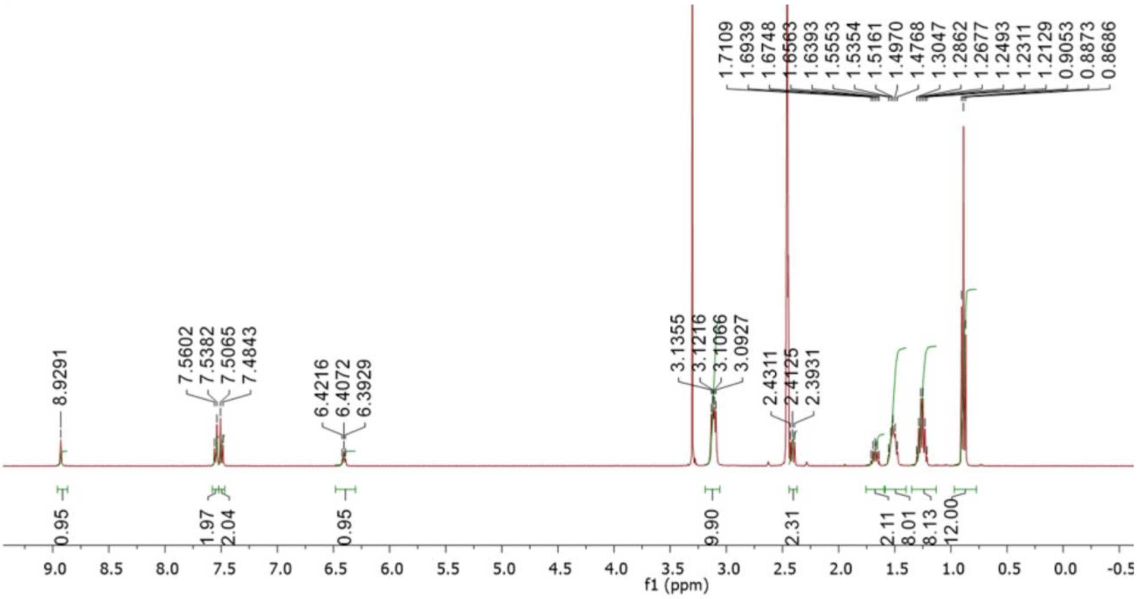

Figure S3 - ^{1}H NMR of compound **2** in DMSO-d_6 conducted at 298.15 K.

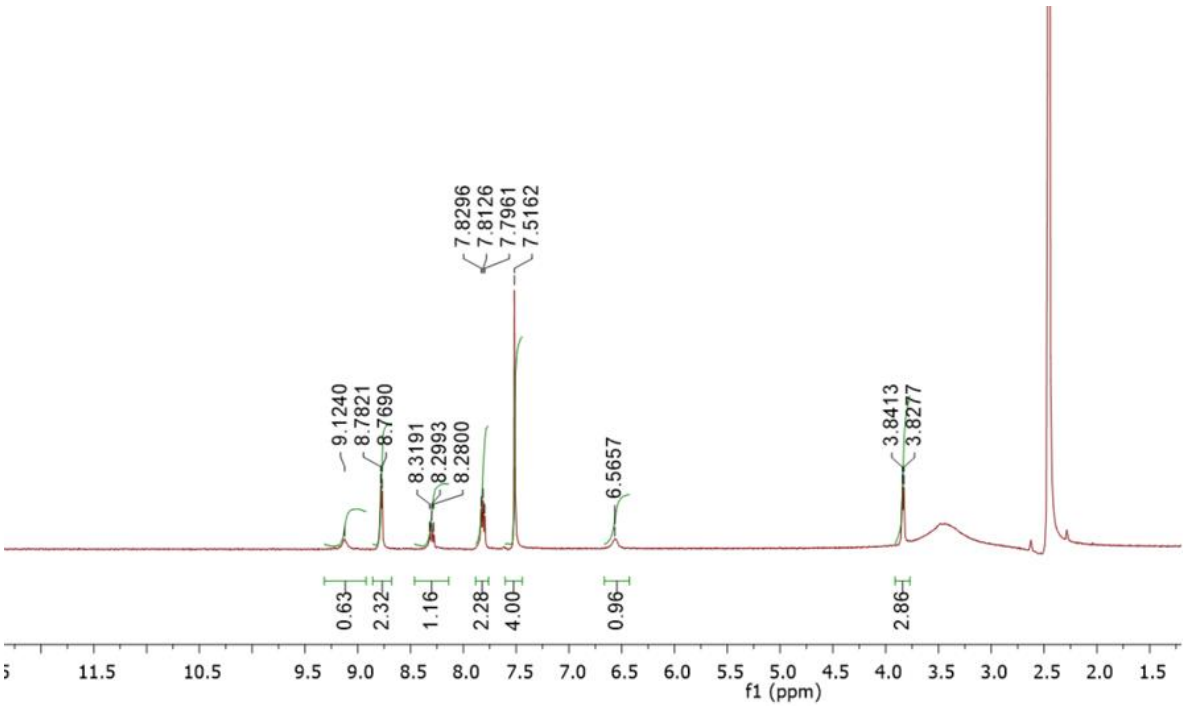

Figure S4 - ^{1}H NMR of compound **3** in DMSO-d_6 conducted at 298.15 K.

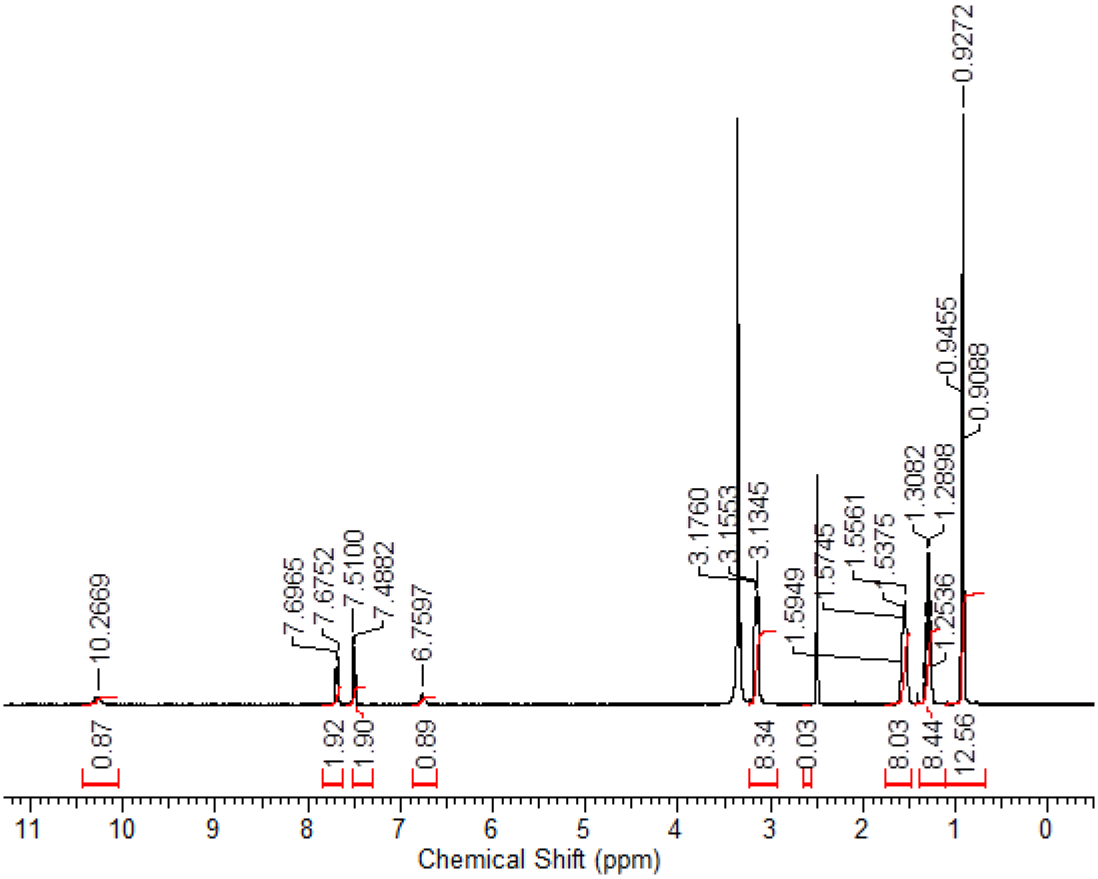

Figure S5 - ^{1}H NMR of compound **4** in DMSO-d_6 conducted at 298.15 K.

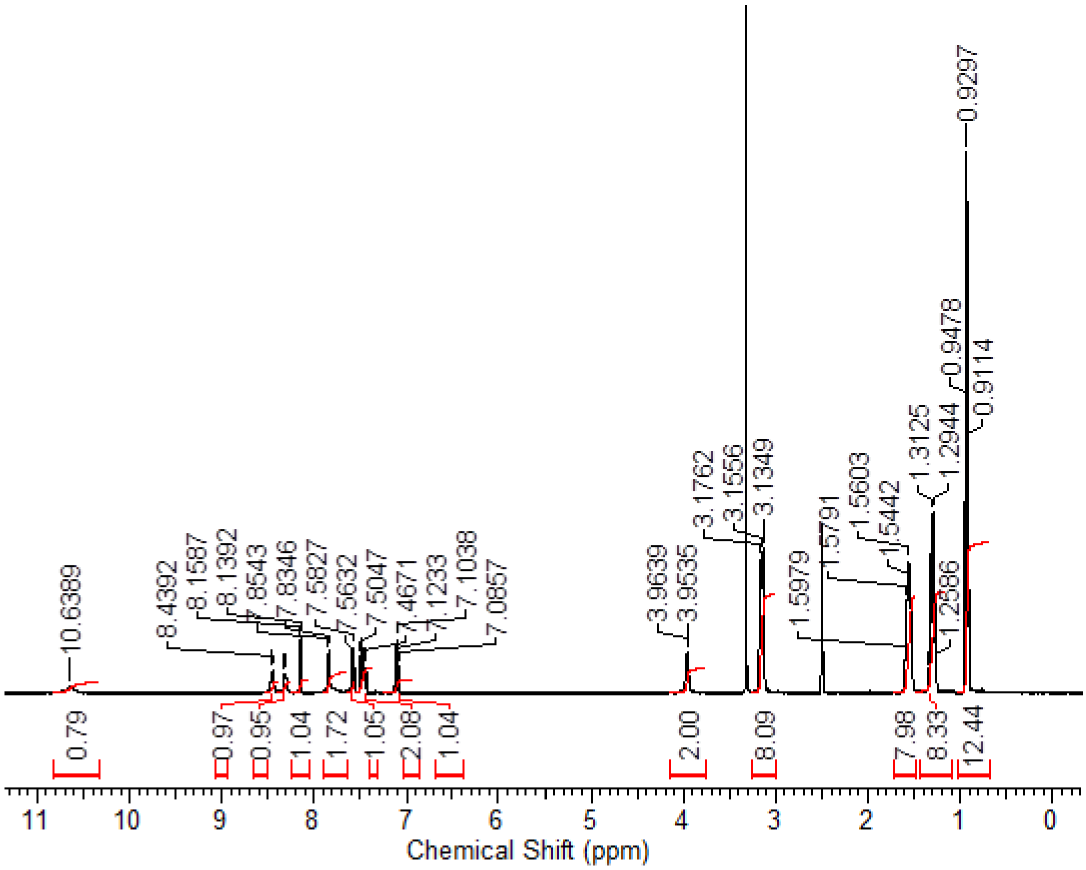

Figure S6 - ^{1}H NMR of compound **5** in DMSO-d$_6$ conducted at 298.15 K.

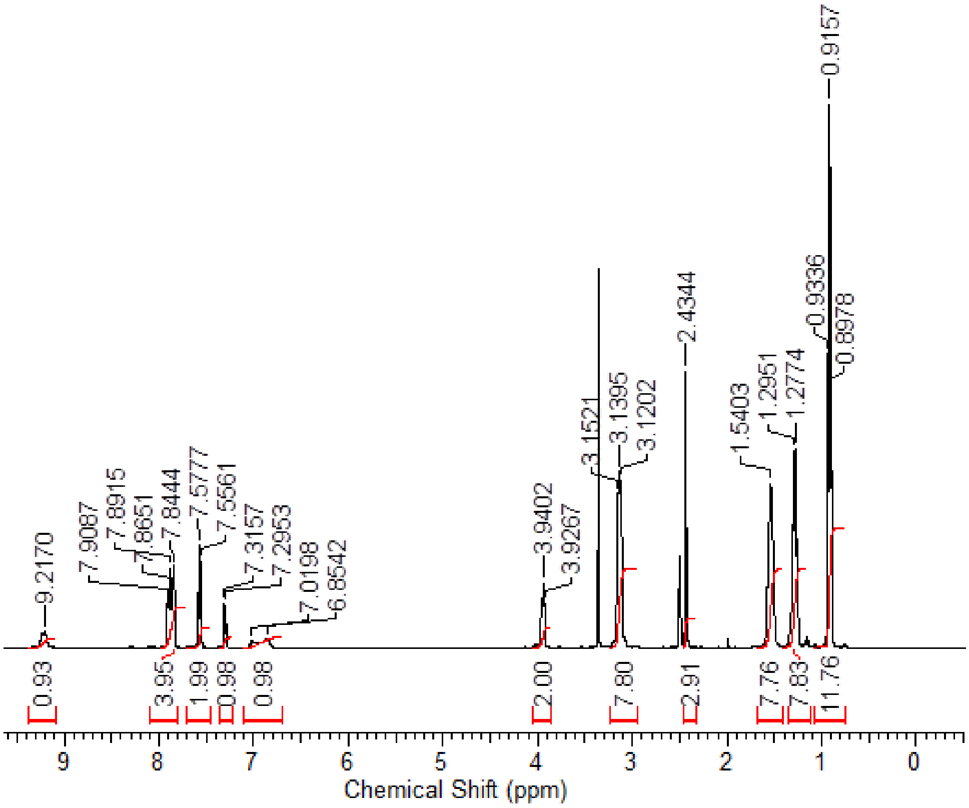

Figure S7 - ^{1}H NMR of compound **6** in DMSO-d$_6$ conducted at 298.15 K.

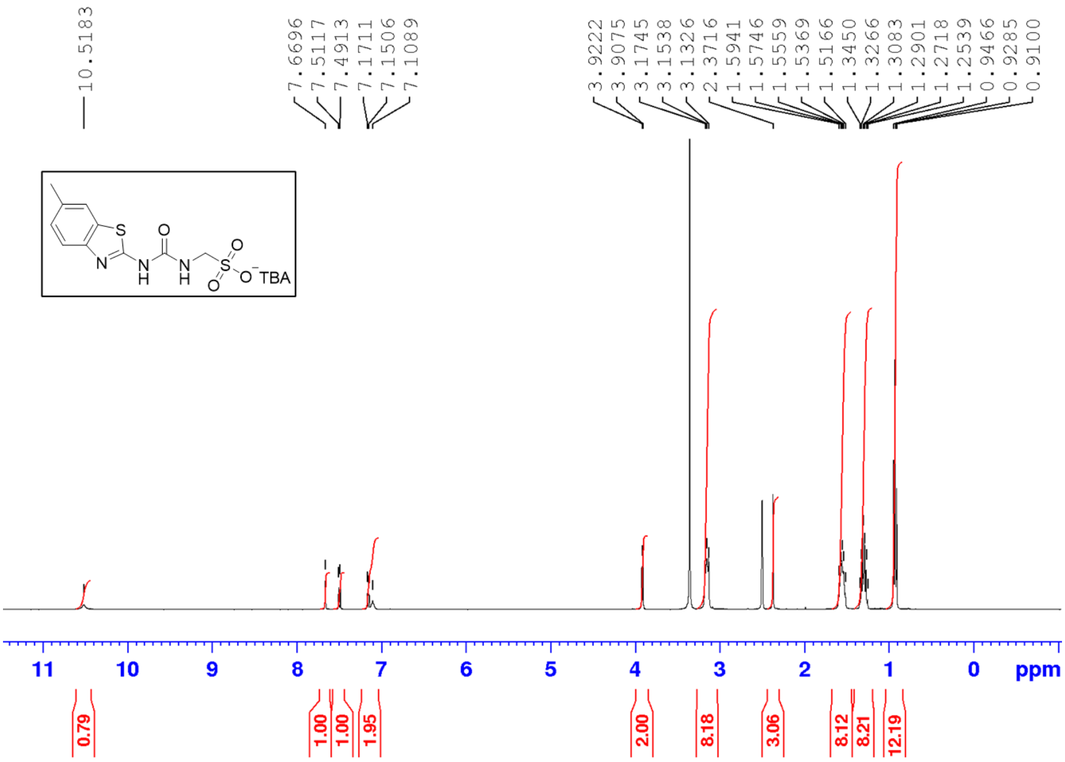

Figure S8 - ^{1}H NMR of compound **7** in DMSO-d$_6$ conducted at 298.15 K.

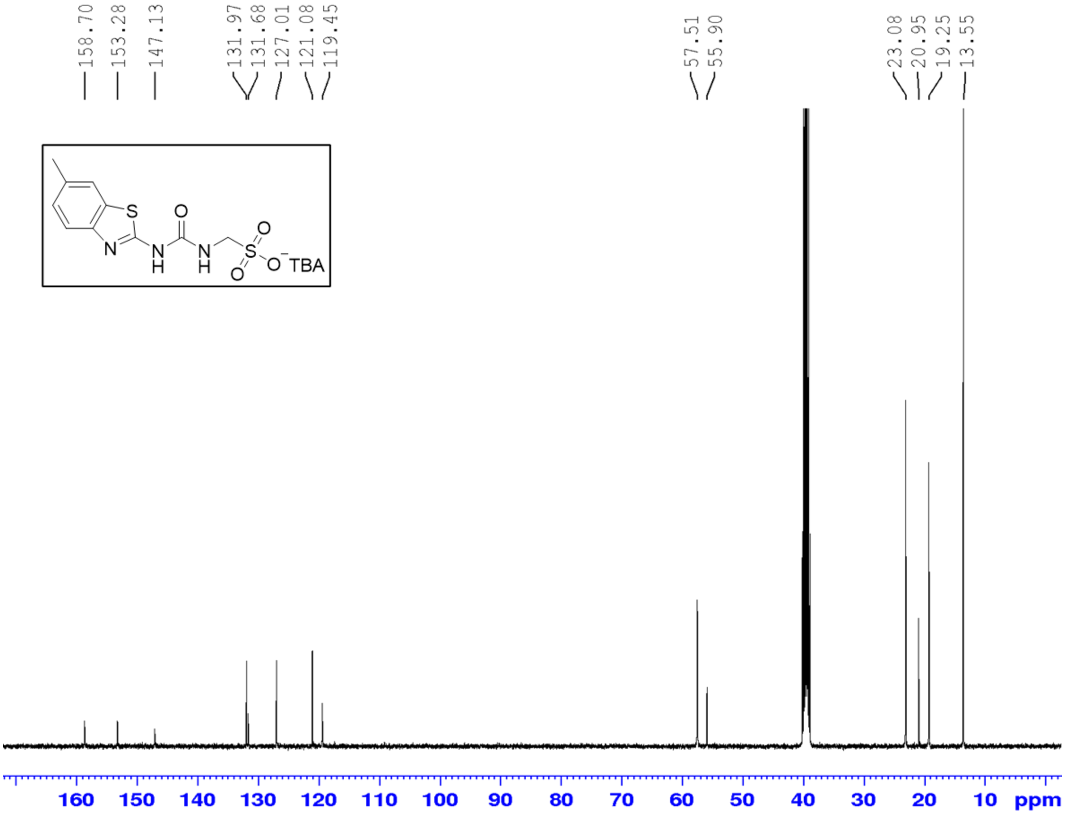

Figure S9 - ^{13}C NMR of compound **7** in DMSO-d$_6$ conducted at 298.15 K.

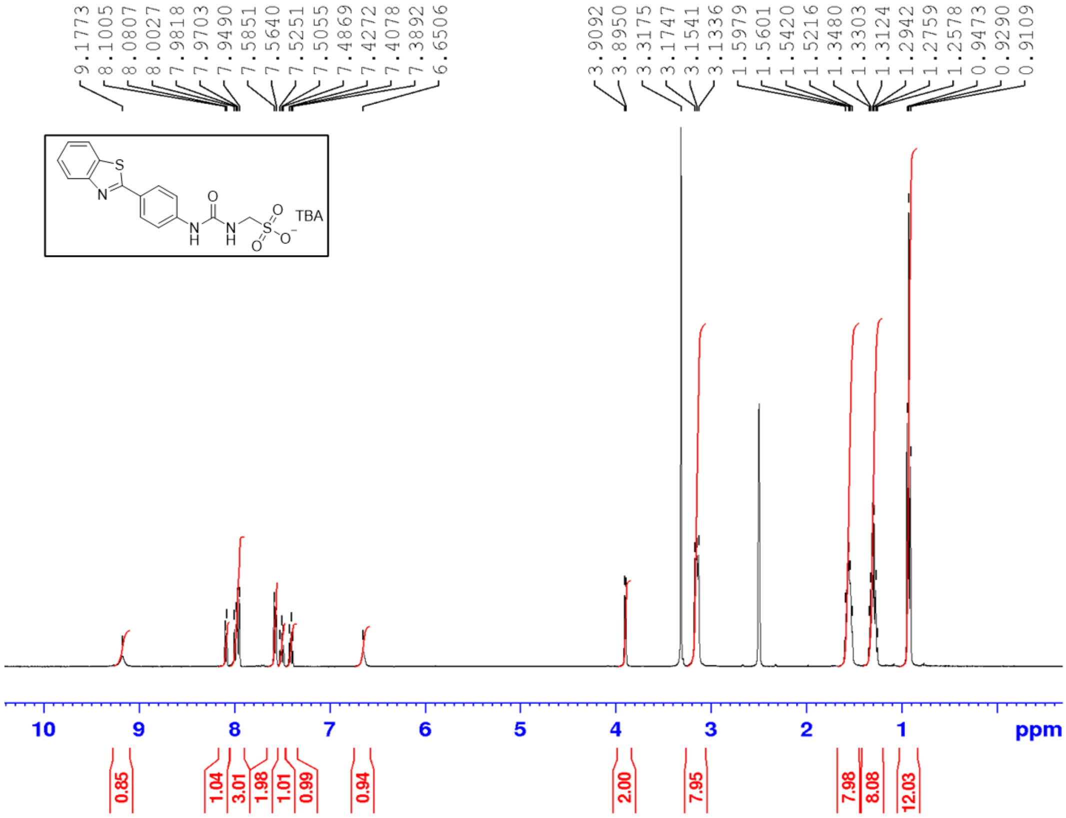

Figure S10 - ^{1}H NMR of compound **8** in DMSO-d_6 conducted at 298.15 K.

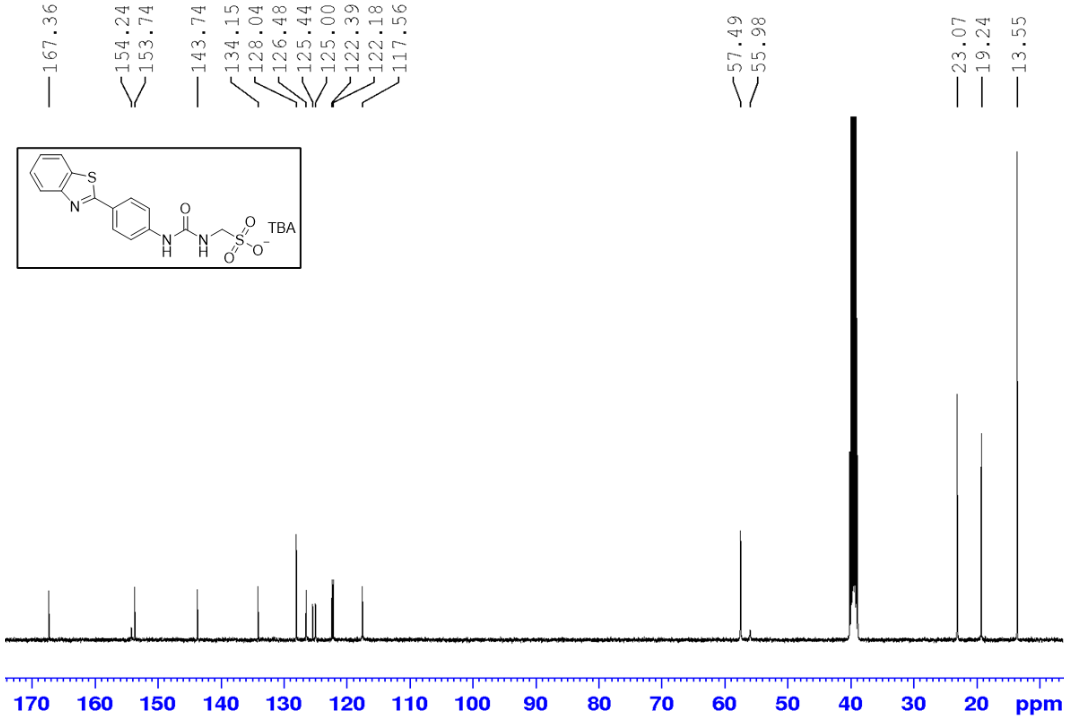

Figure S11 - ^{13}C NMR of compound **8** in DMSO-d_6 conducted at 298.15 K.

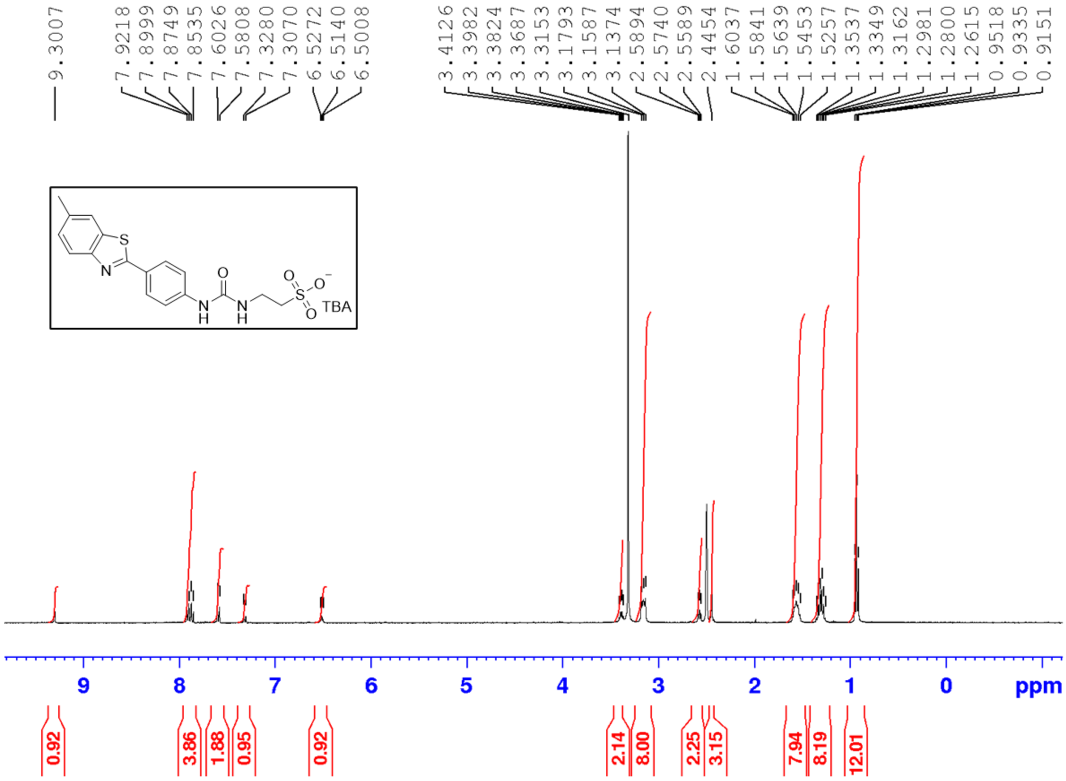

Figure S12 - ^{1}H NMR of compound **9** in DMSO-d$_6$ conducted at 298.15 K.

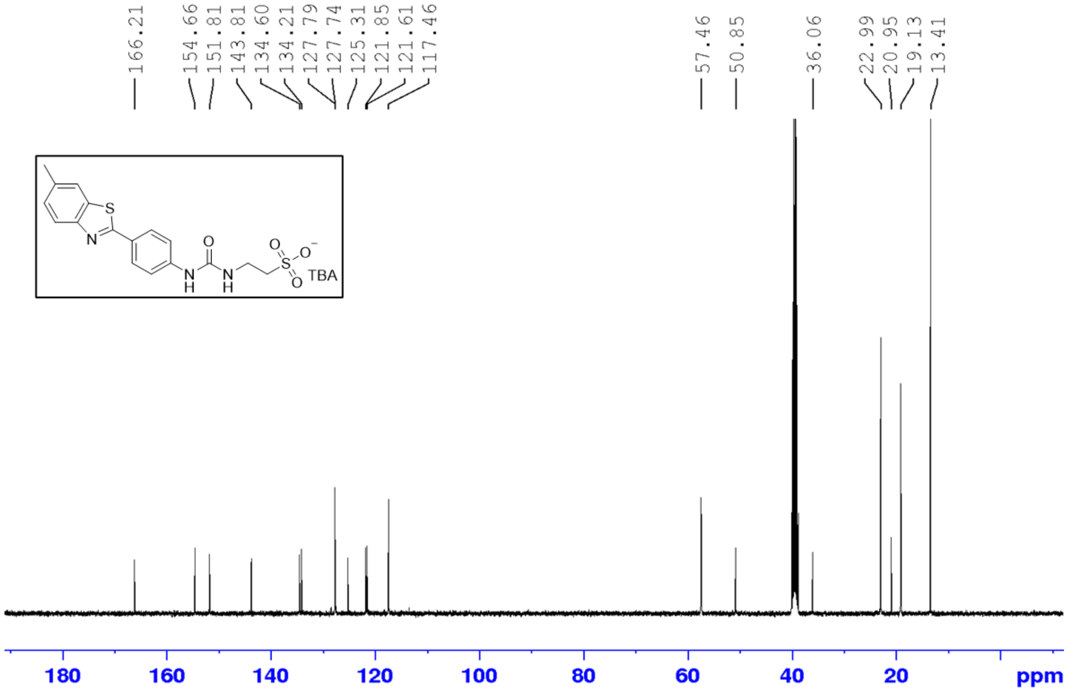

Figure S13 - ^{13}C NMR of compound **9** in DMSO-d$_6$ conducted at 298.15 K.

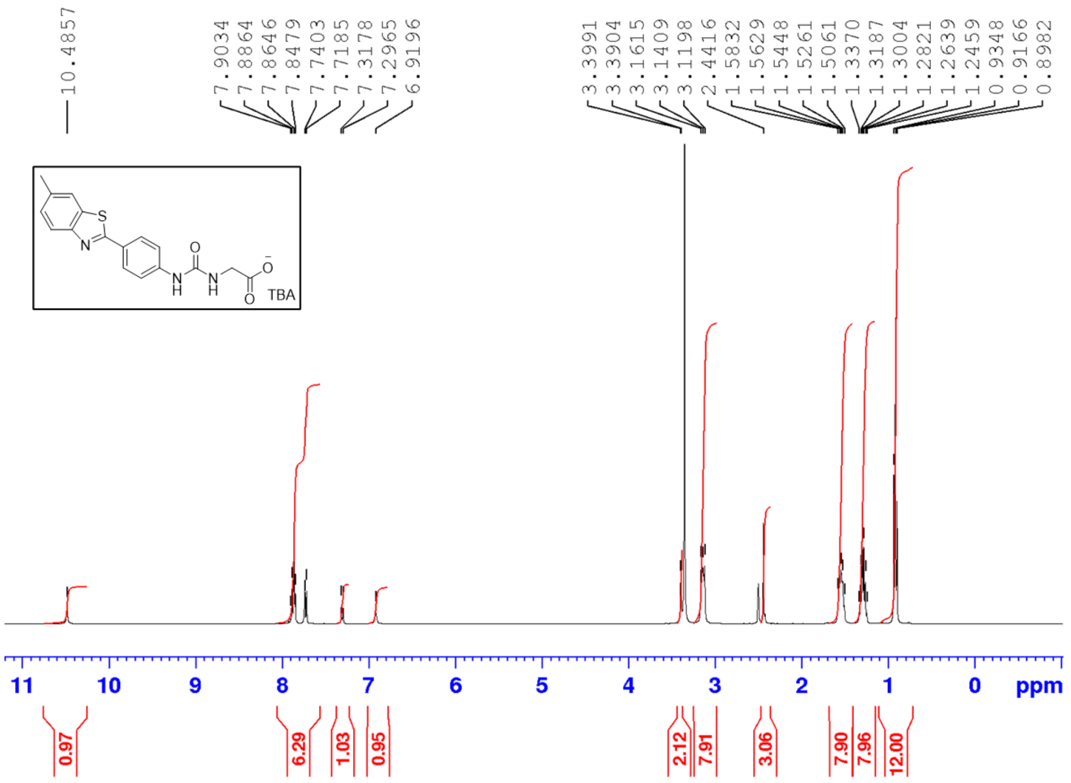

Figure S14 - ^{1}H NMR of compound **11** in DMSO-d$_6$ conducted at 298.15 K.

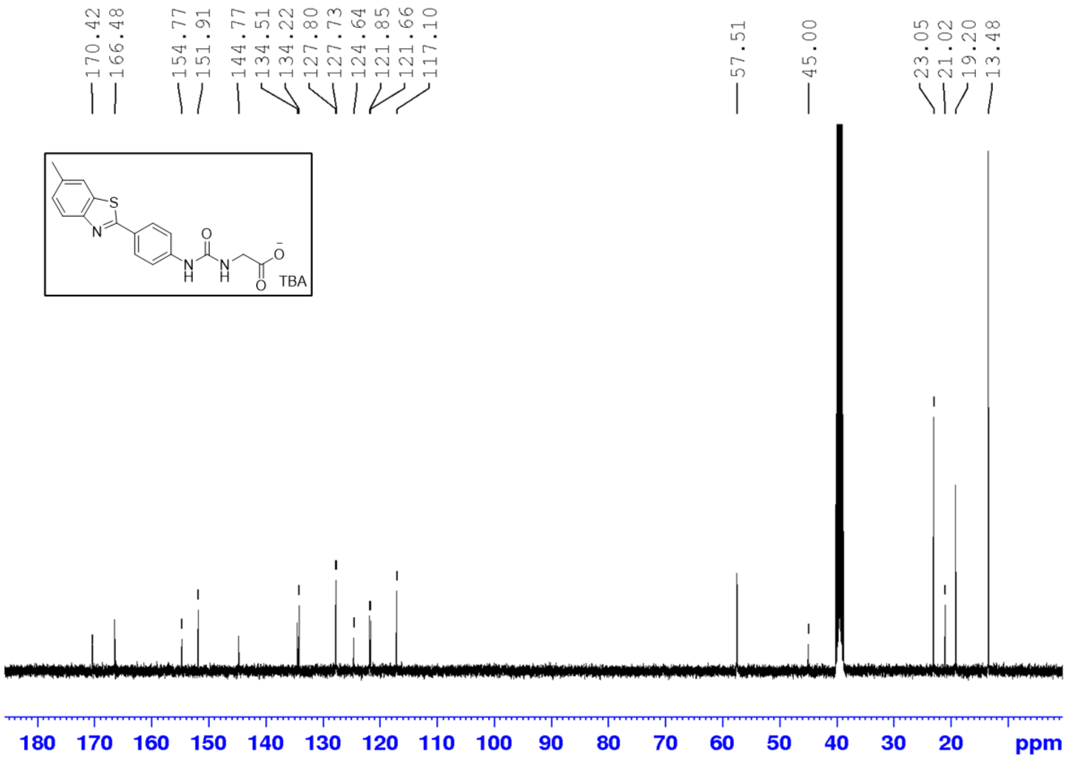

Figure S15 - ^{13}C NMR of compound **11** in DMSO-d$_6$ conducted at 298.15 K.

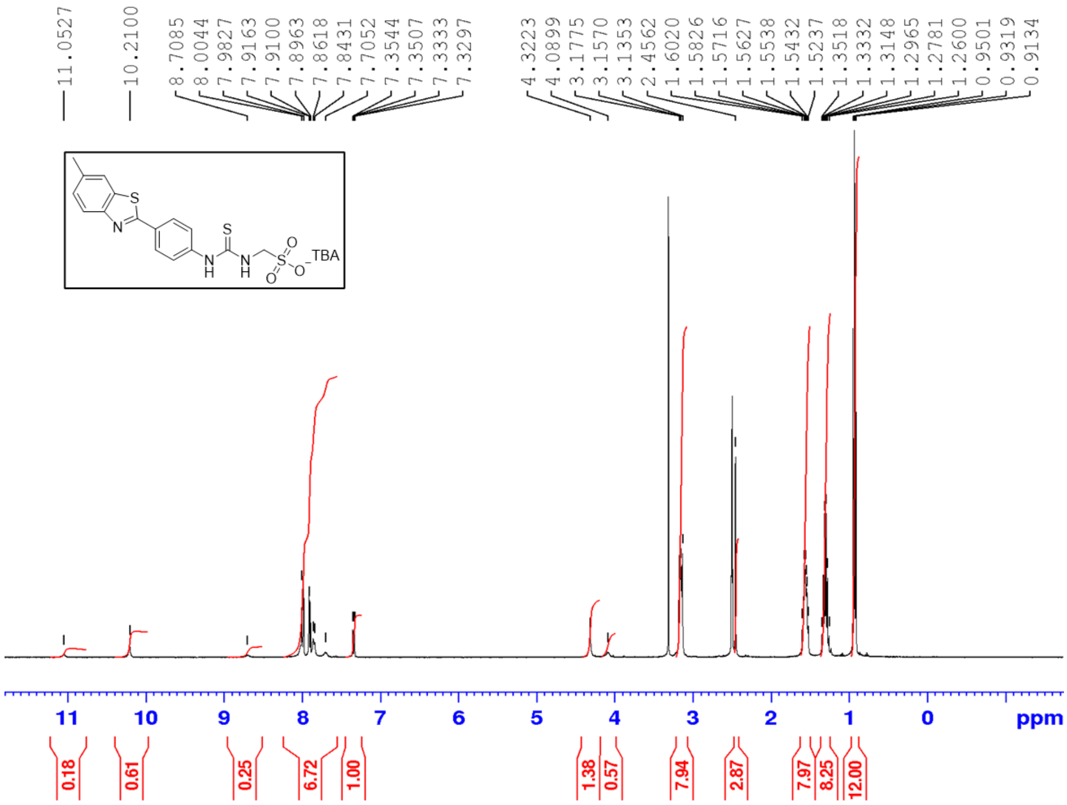

Figure S16 – ^{1}H NMR of compound **12** in DMSO-d$_6$ conducted at 298.15 K.

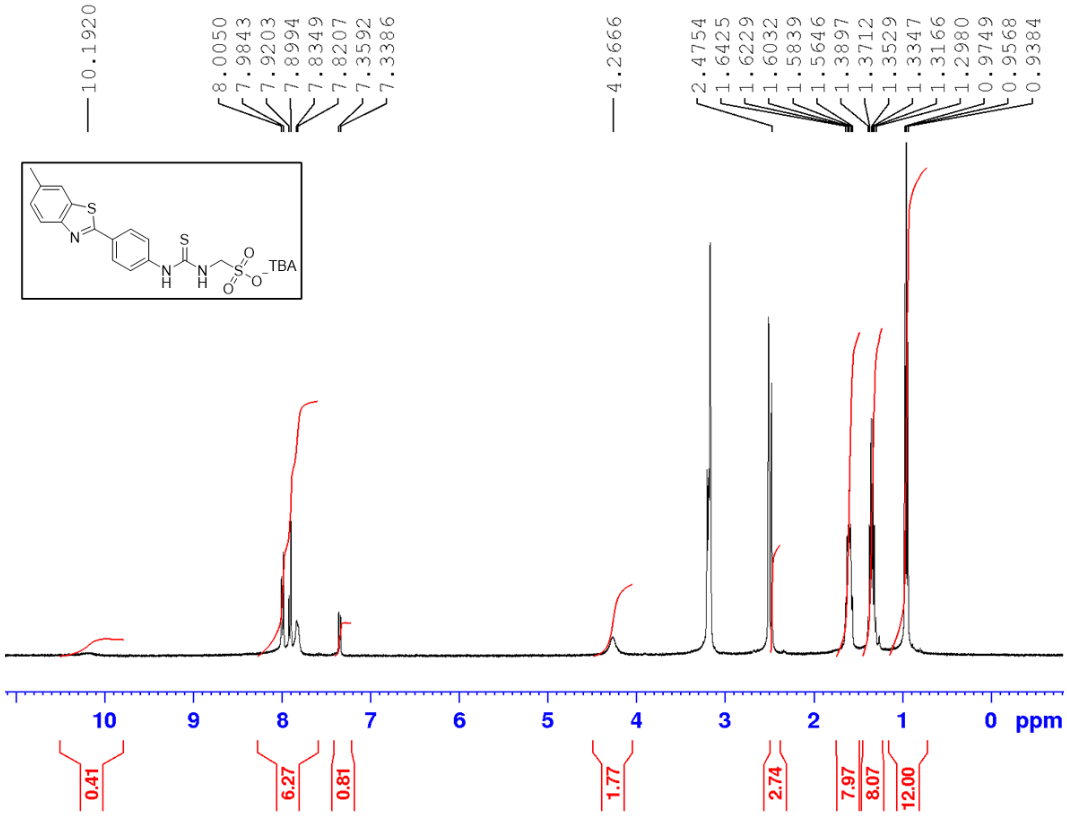

Figure S17 – ^{1}H NMR of compound **12** in DMSO-d$_6$ conducted at 333 K.

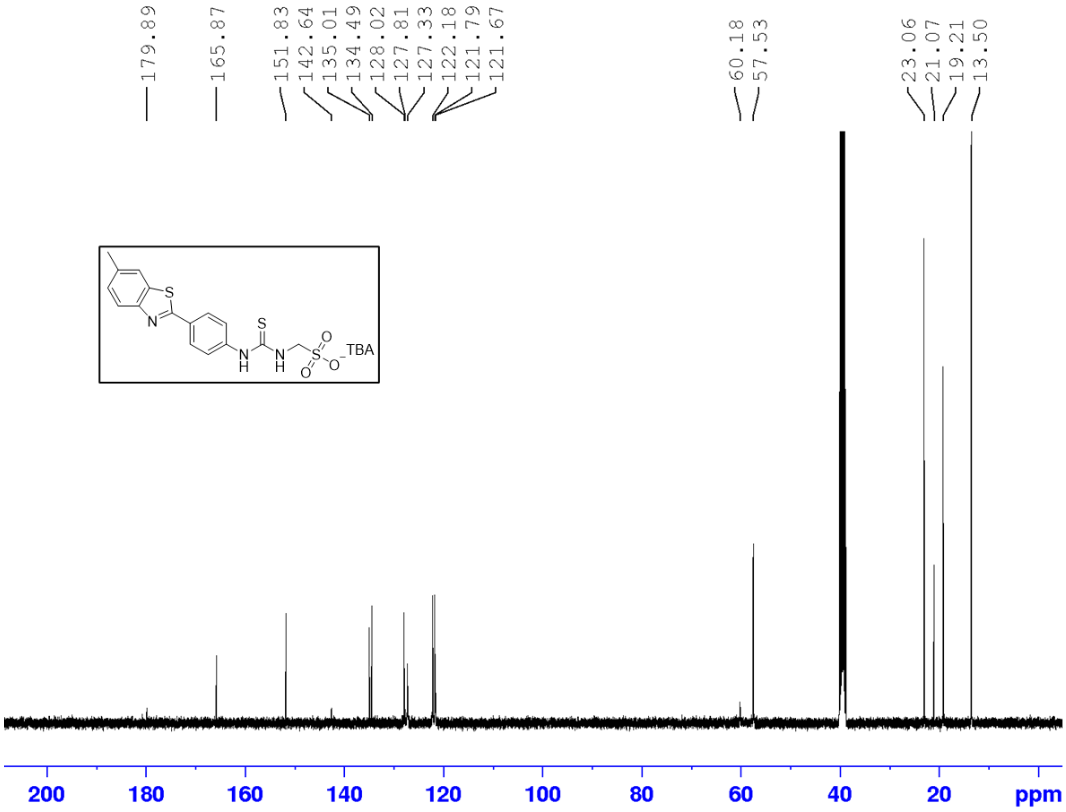

Figure S18 – ^{13}C NMR of compound **12** in DMSO-d$_6$ conducted at 298.15 K.

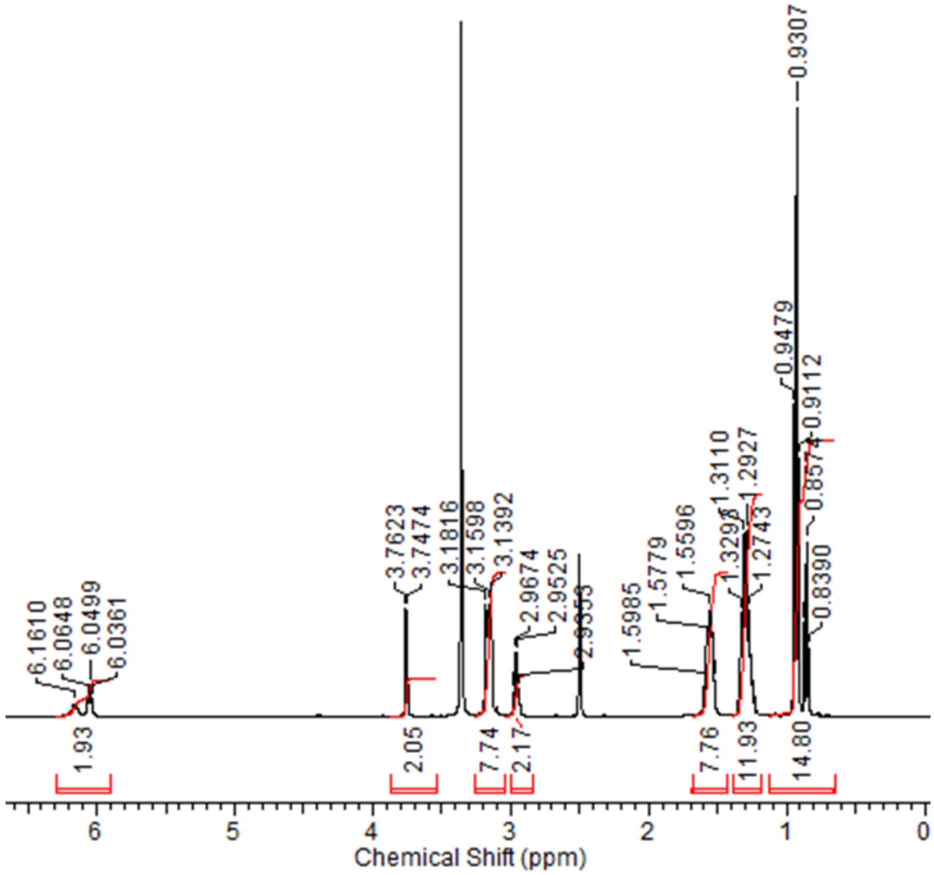

Figure S19 – ^{1}H NMR of compound **13** in DMSO-d$_6$ conducted at 298.15 K.

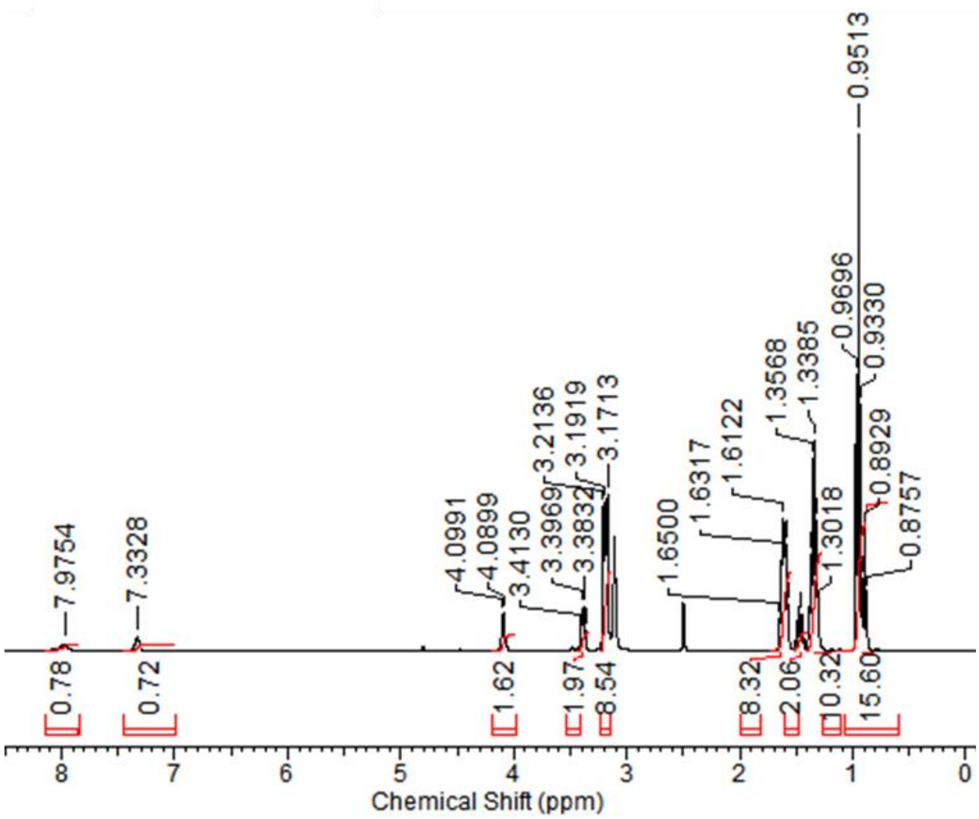

Figure S20 – ^{1}H NMR of compound **14** in DMSO-d_6 conducted at 333.15 K.

Section 6: Antimicrobial Screening Data

USA 300 Methicillin-resistant *Staphylococcus aureus* (MRSA)

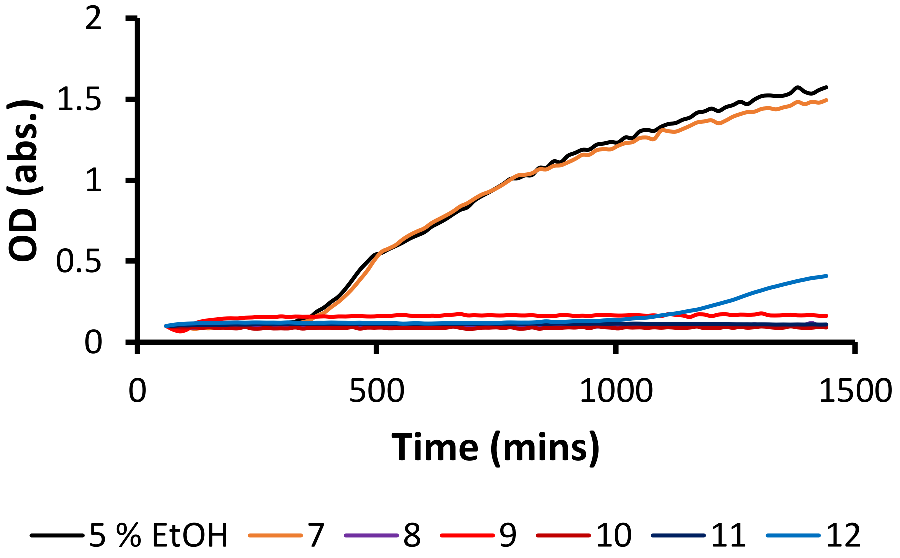

Figure S21 - MRSA growth curves created from an average of 6 optical density readings in the presence of compounds **7**, **8**, **9**, **10**, **11** and **12**.

Escherichia coli (*E. coli*) DH10B

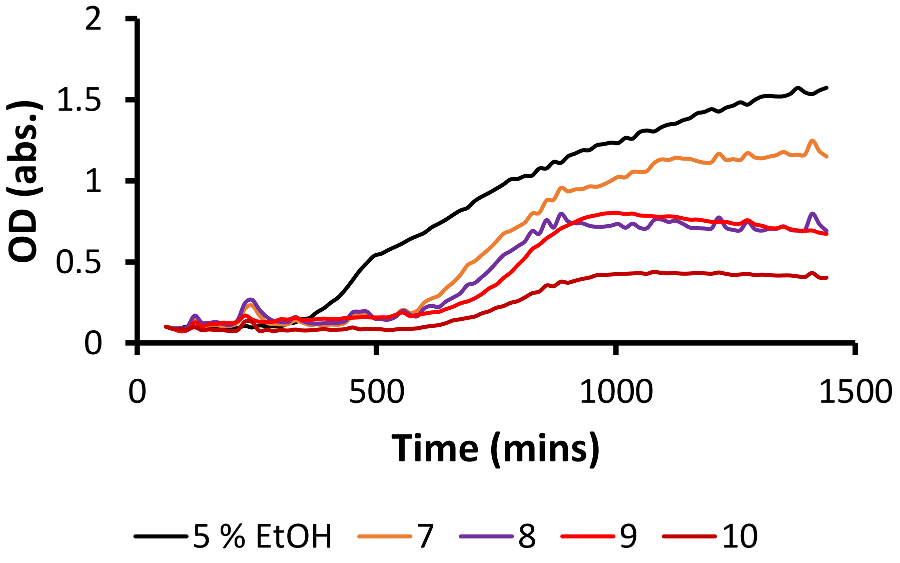

Figure S22 - E. coli growth curves created from an average of 6 optical density readings in the presence of compounds **7**, **8**, **9** and **10**.

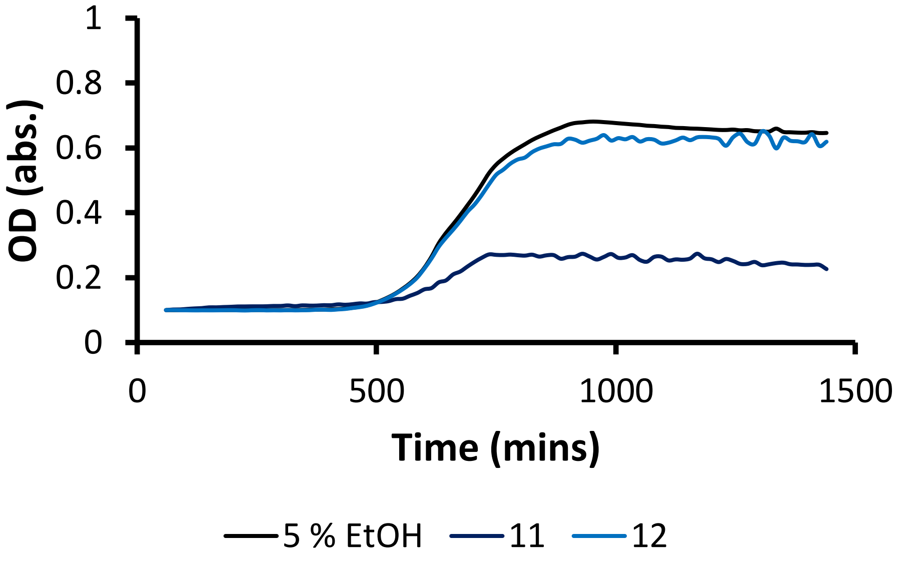

Figure S23 - E. coli growth curves created from an average of 6 optical density readings in the presence of compounds **11** and **12**.

Section 7: Full Growth Curve Data

USA 300 Methicillin-resistant *Staphylococcus aureus* (MRSA)

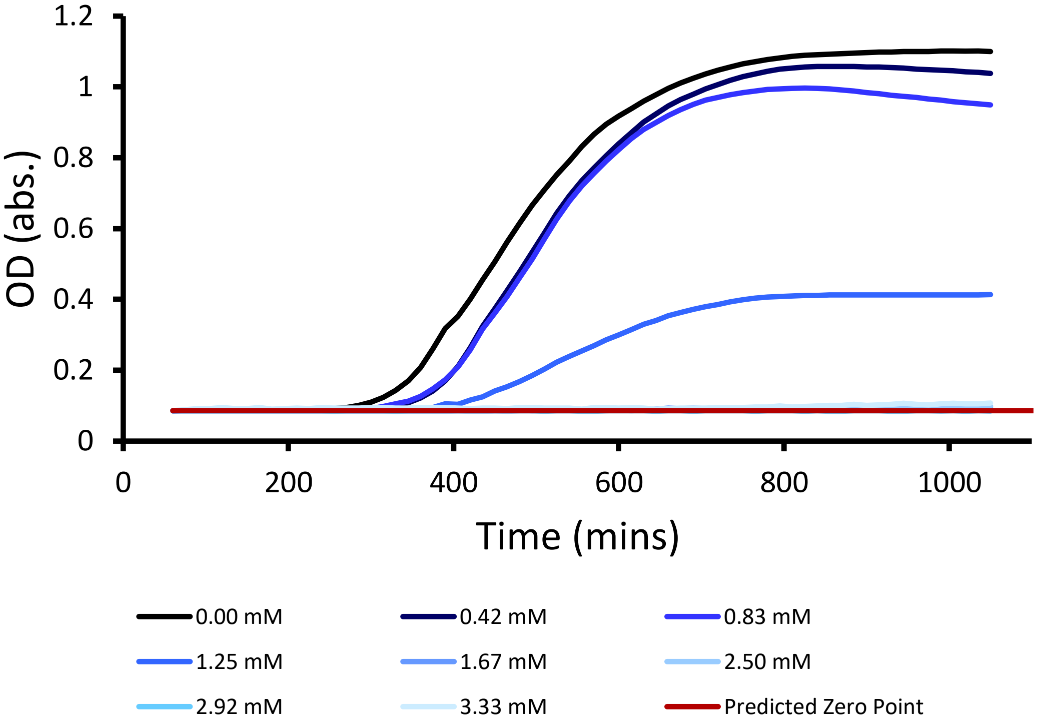

Figure S24 - MRSA USA 300 growth curves created from an average of 6 optical density readings for each concentration in the presence of compound **8** at varying concentrations.

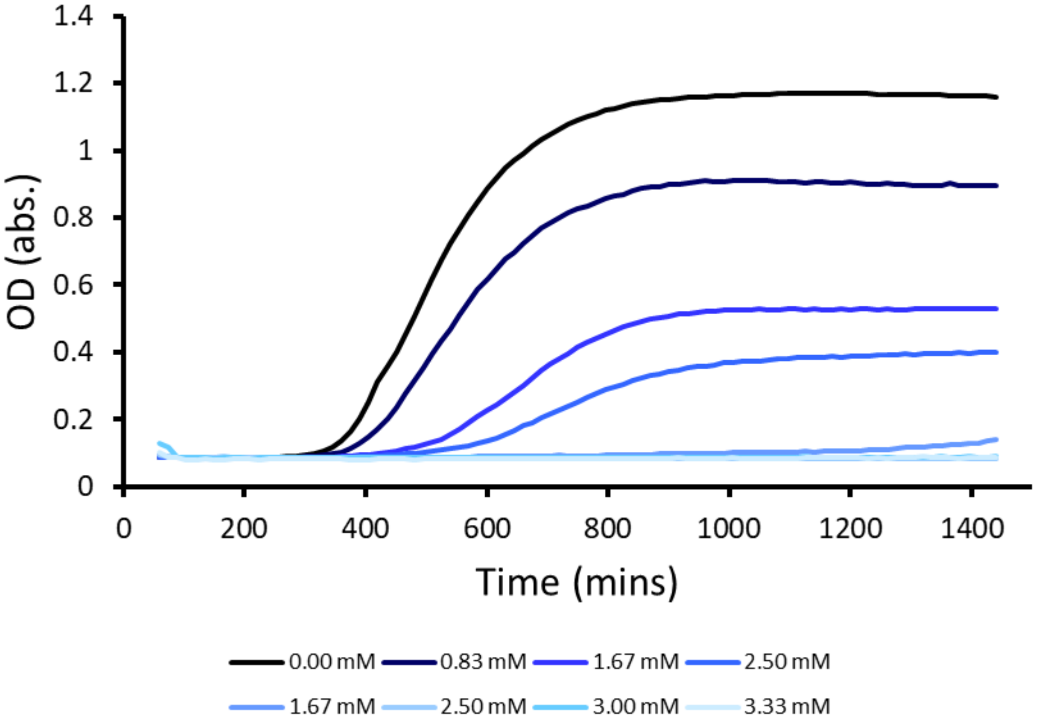

Figure S25 - MRSA USA 300 growth curves created from an average of 6 optical density readings for each concentration in the presence of compound **9** at varying concentrations.

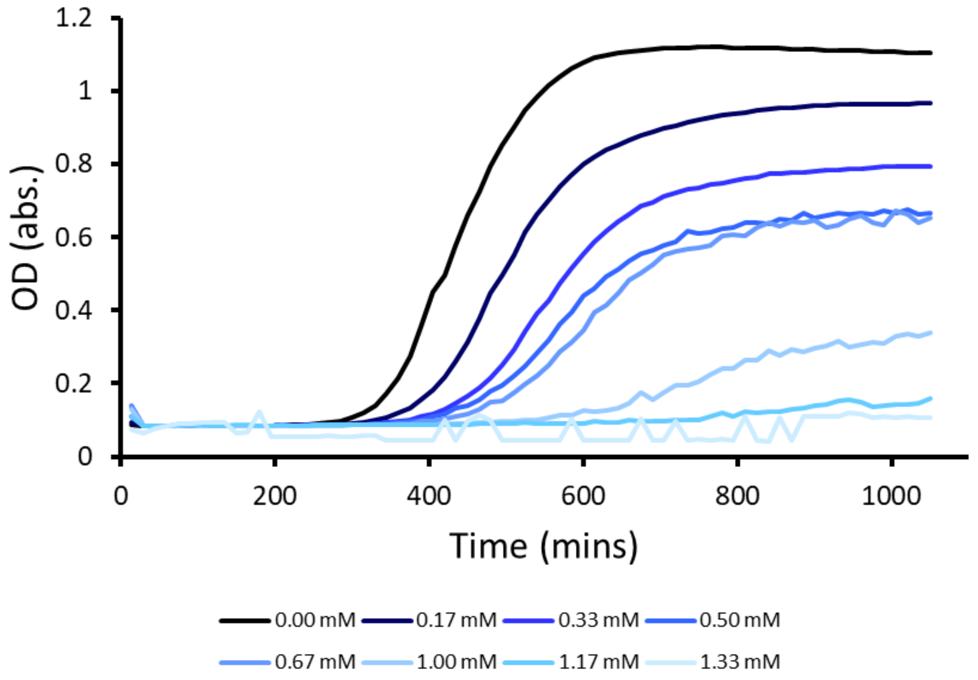

Figure S26 - MRSA USA 300 growth curves created from an average of 6 optical density readings for each concentration in the presence of compound **10** at varying concentrations.

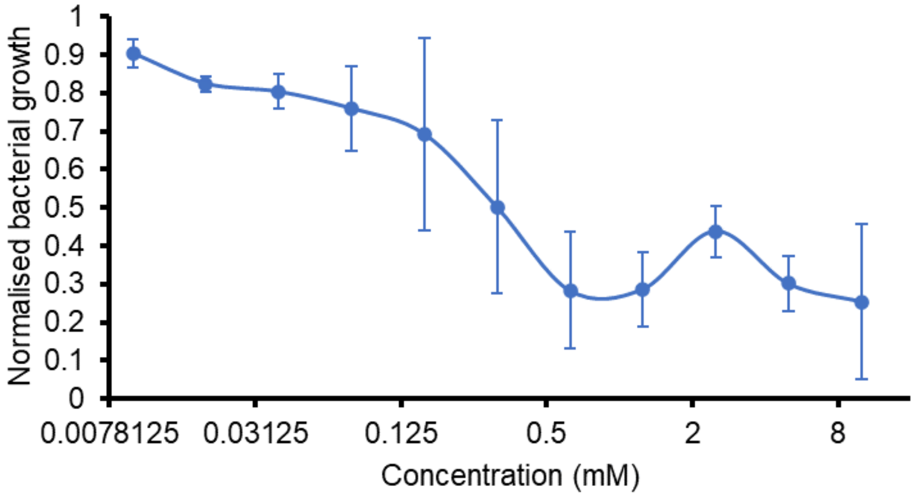

Figure S27 – MRSA USA 300 growth curves created from an average of 6 optical density readings for each concentration in the presence of compound **11** at varying concentrations.

Escherichia coli (*E. coli*)

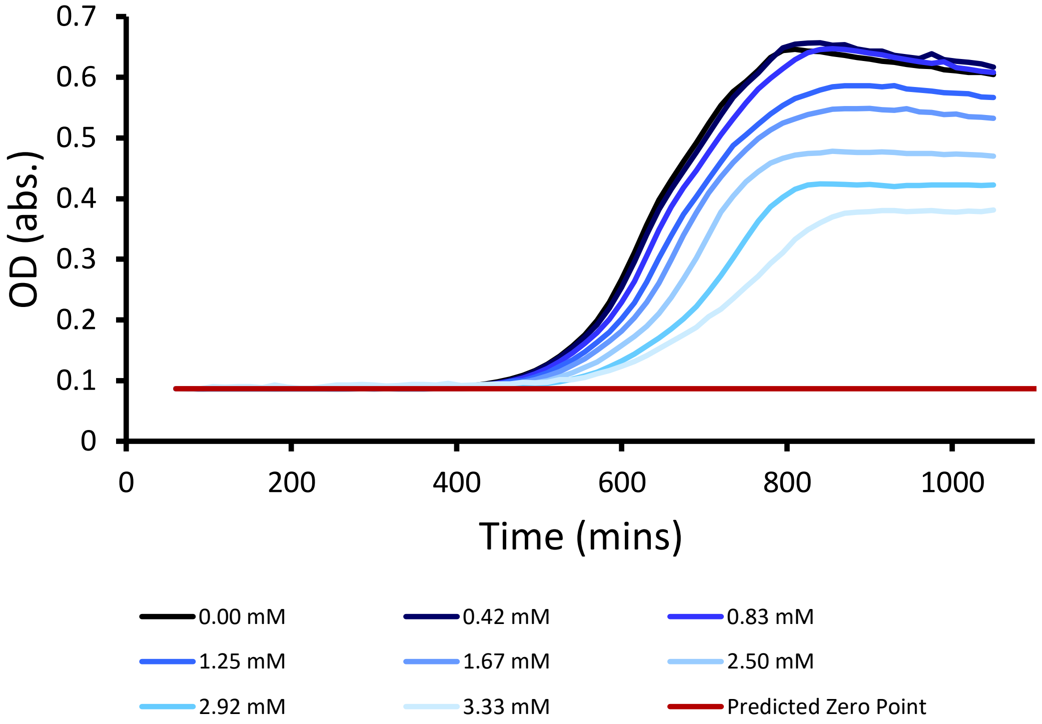

Figure S28 - *E. coli* DH10B growth curves created from an average of 6 optical density readings for each concentration in the presence of compound **8** at varying concentrations.

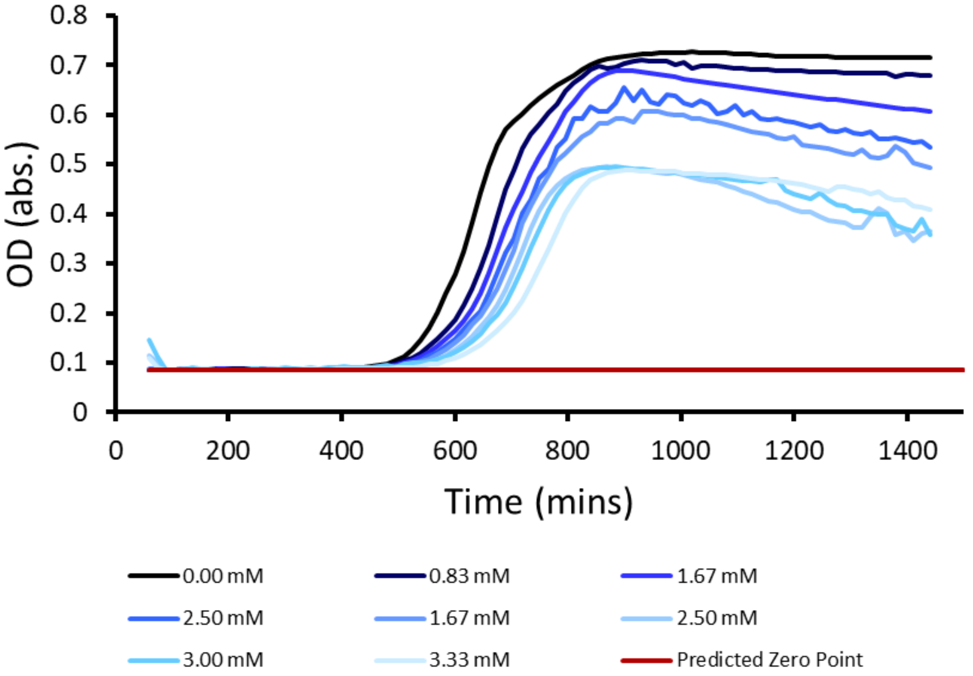

Figure S29 - *E. coli* DH10B growth curves created from an average of 6 optical density readings for each concentration in the presence of compound **9** at varying concentrations.

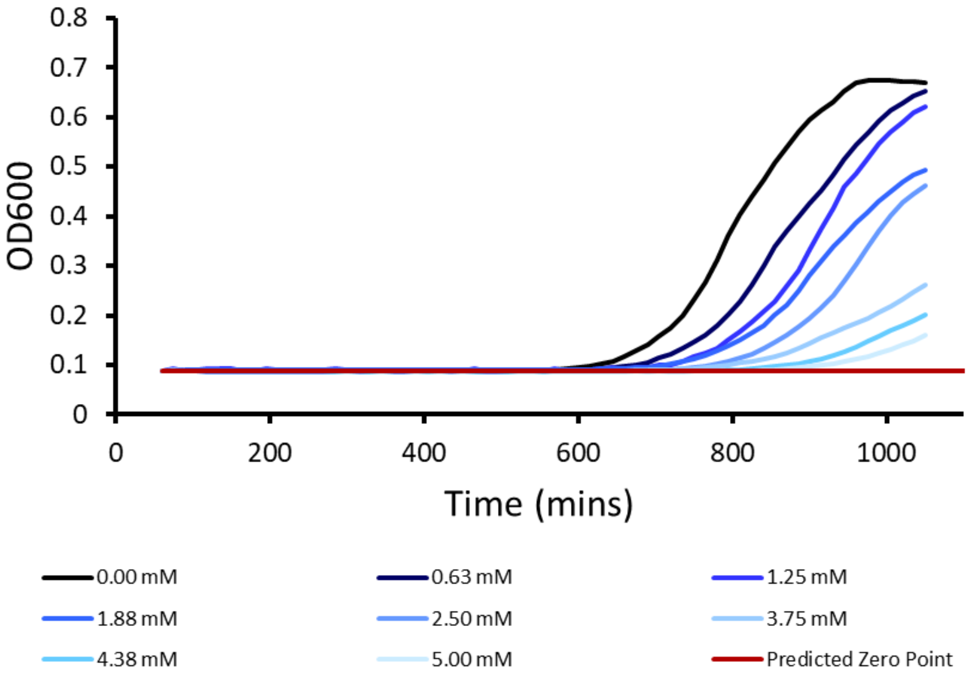

Figure S30 - *E. coli* DH10B growth curves created from an average of 6 optical density readings for each concentration in the presence of compound **10** at varying concentrations.

Section 8: MIC_{50} Determination

USA 300 Methicillin-resistant *Staphylococcus aureus* (MRSA)

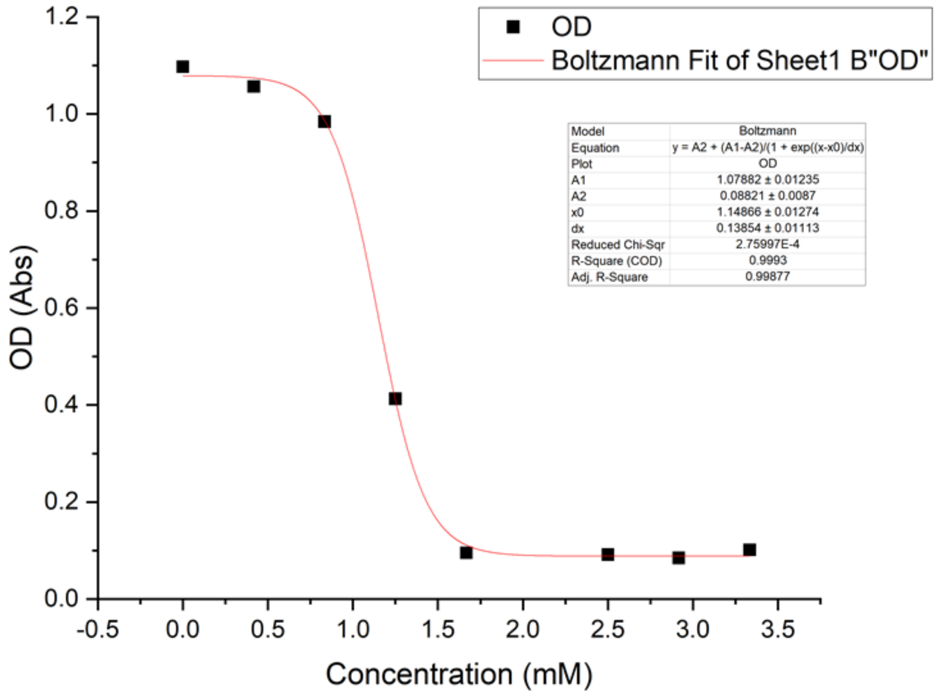

Figure S31 - Boltzmann fit used to calculate MIC_{50} using optical density values at 900 minutes for compound **8** at varying concentrations.

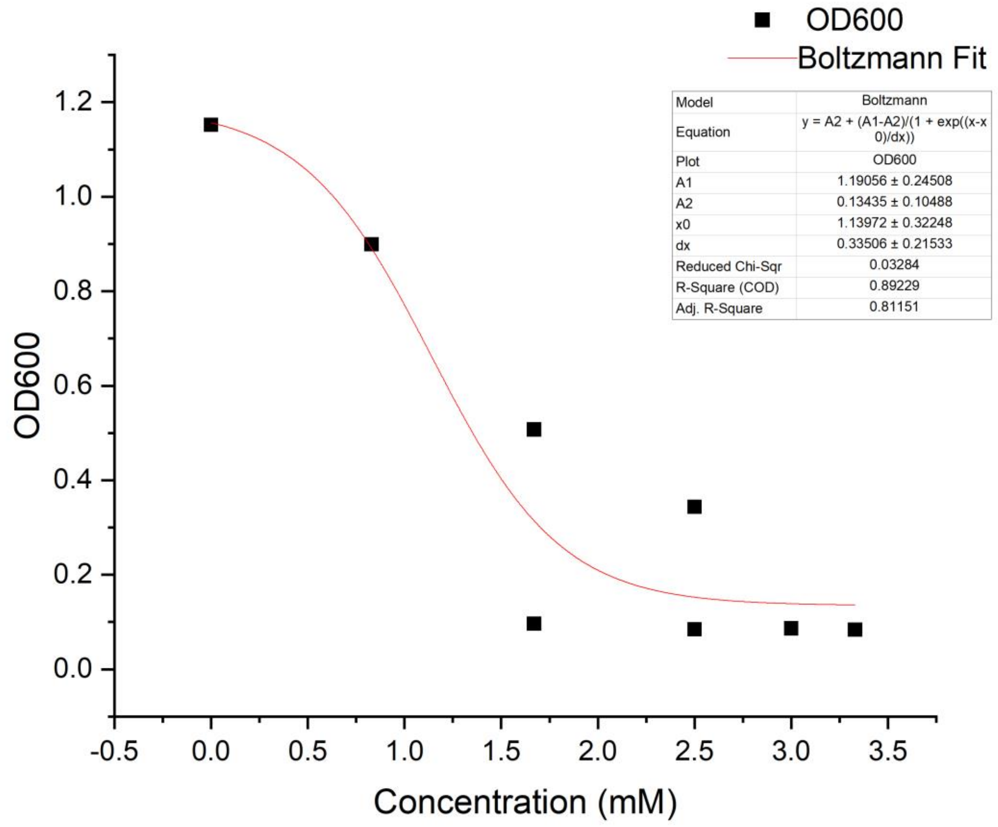

Figure S32 - Boltzmann fit used to calculate MIC_{50} using optical density values at 900 minutes for compound **9** at varying concentrations.

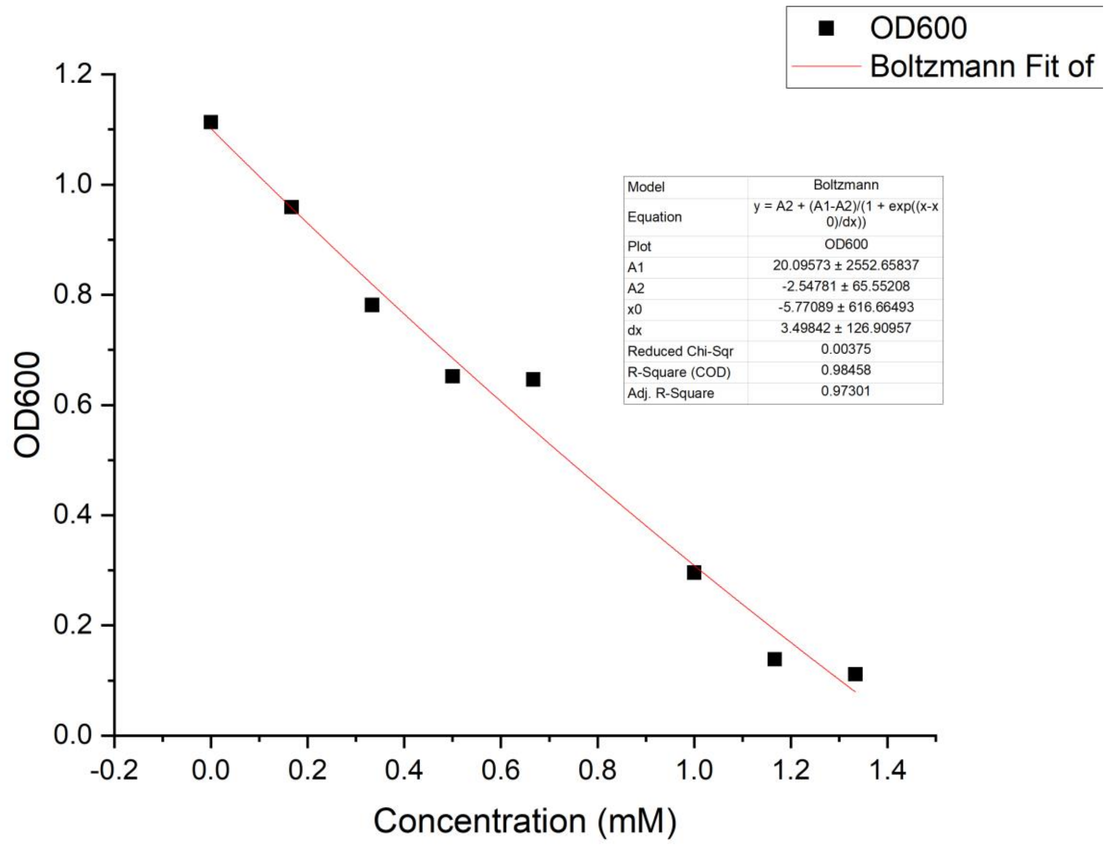

Figure S33 - Boltzmann fit used to calculate MIC_{50} using optical density values at 900 minutes for compound **10** at varying concentrations.

Escherichia coli (*E. coli*)

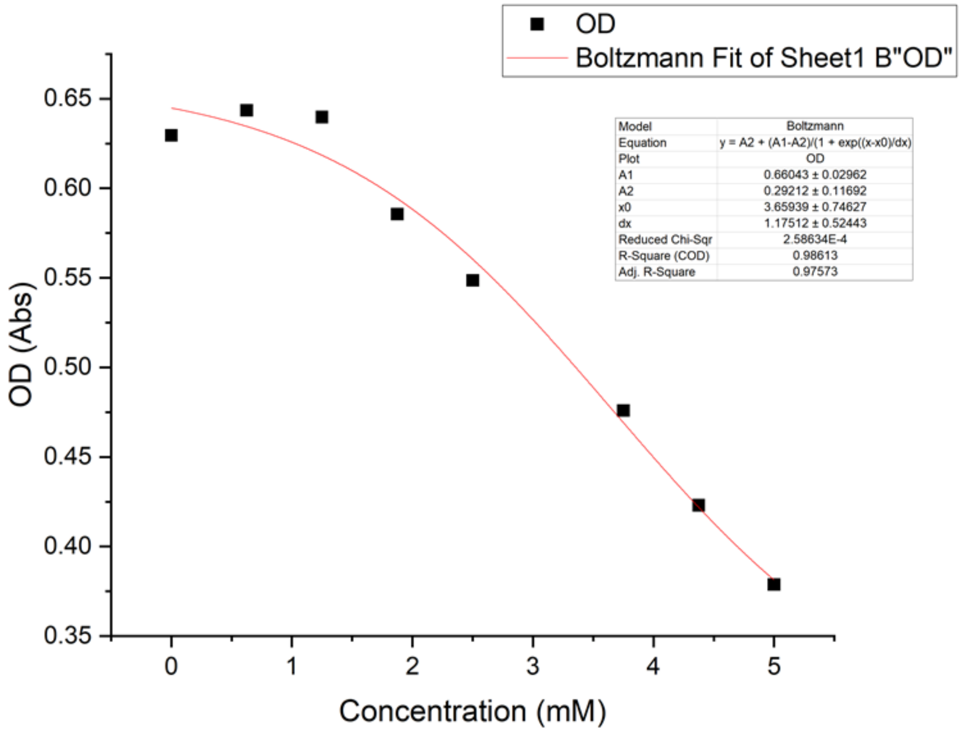

Figure S34 - Boltzmann fit used to calculate MIC_{50} using optical density values at 900 minutes for compound **8** at varying concentrations.

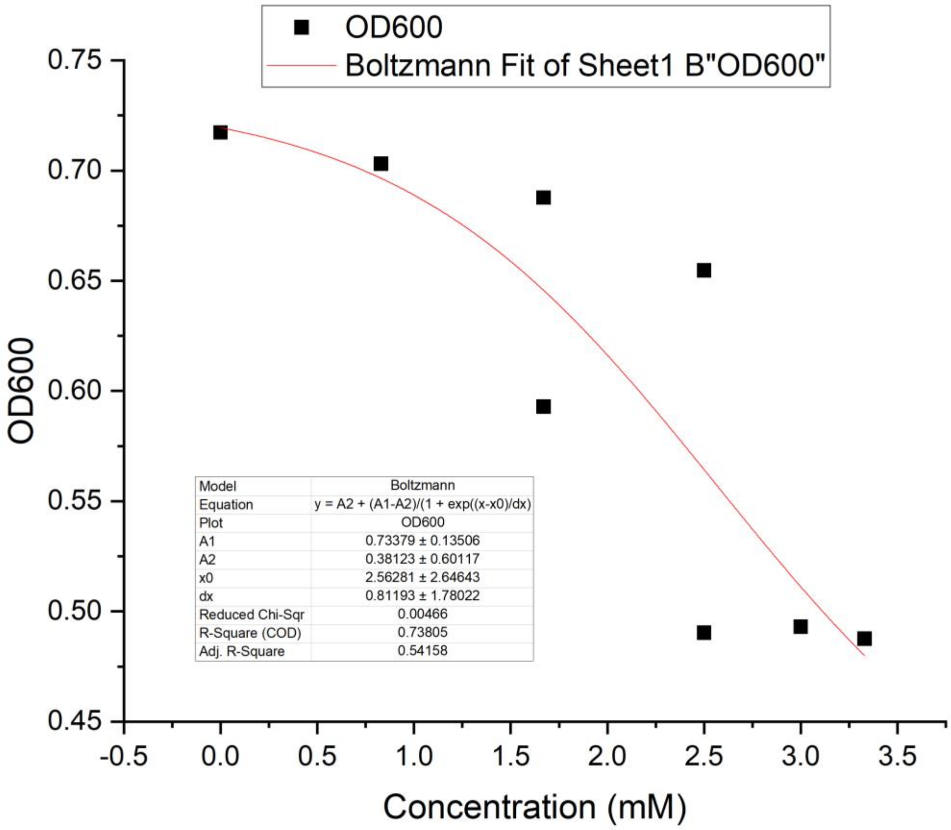

Figure S35 - Boltzmann fit used to calculate MIC_{50} using optical density values at 900 minutes for compound **9** at varying concentrations.

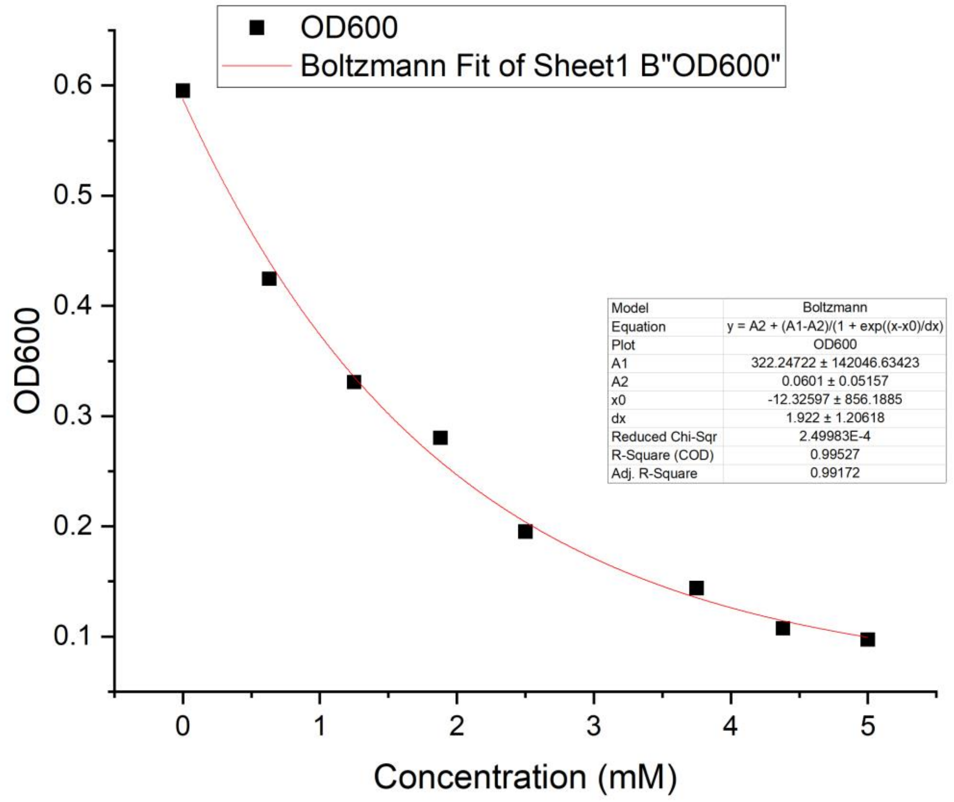

Figure S36 - Boltzmann fit used to calculate MIC_{50} using optical density values at 900 minutes for compound **10** at varying concentrations.

Table S3 - MIC_{50} values (µg/mL) determined for **1** – **14** against clinically relevant Gram-positive MRSA USA300 and model Gram-negative *E. coli* DH10B bacteria (n = 3) at an initial calibrated cell concentration equal to the 0.5 McFarland standard, after 900 mins. Here the number included within the bracket represents the ranking of the SSAs antimicrobial efficacy, with 1 = most active antimicrobial agent. Please see ESI Section 3 for further experimental details.

SSA	MRSA	*E. coli*	SSA	MRSA	*E. coli*
**1**	248	2078	**8**	696	2214
**2**	*a*	840	**9**	759.50	1367
**3**	132	Fail	**10**	382	750
**4**	574	630	**11**	181-367^{b}	*a*
**5**	599	2159	**12**	*a*	*c*
**6**	576	3107	**13**	1992	*c*
**7**	Fail	Fail	**14**	1436	2821

a = MIC_{50} value could not be determined due to compound solubility. *b* = MIC_{50} values are estimated due to data quality. *c* = SSA did not pass initial antimicrobial screening, exhibited < 10 % inhibition of growth at 3.3 mM after 900 mins and therefore excluded from further study.

Section 9: Surface Tension and Critical Micelle Concentration (CMC) Determination

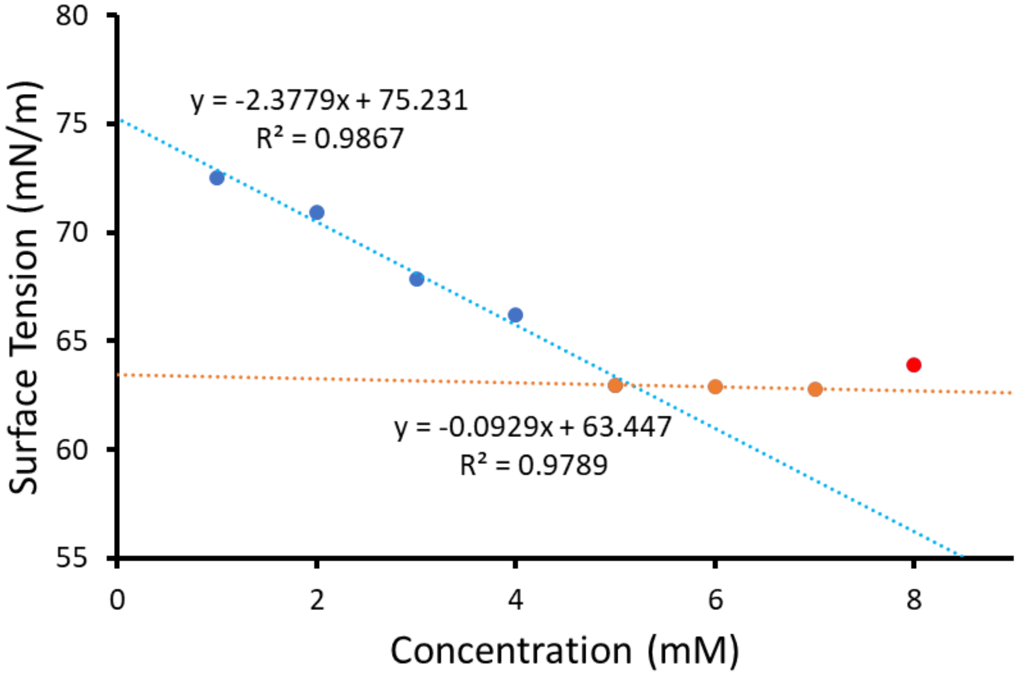

Figure S37 - Calculation of CMC (5.16 mM) for compound **8** in an EtOH:H_2O 1:19 mixture using surface tension measurements.

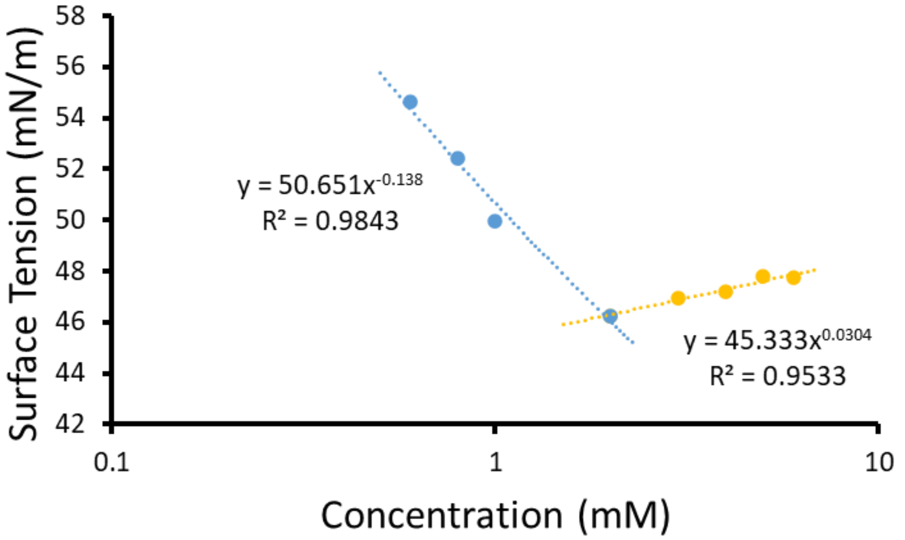

Figure S38 - Calculation of CMC (1.92 mM) for compound **9** in an EtOH:H_2O 1:19 mixture using surface tension measurements.

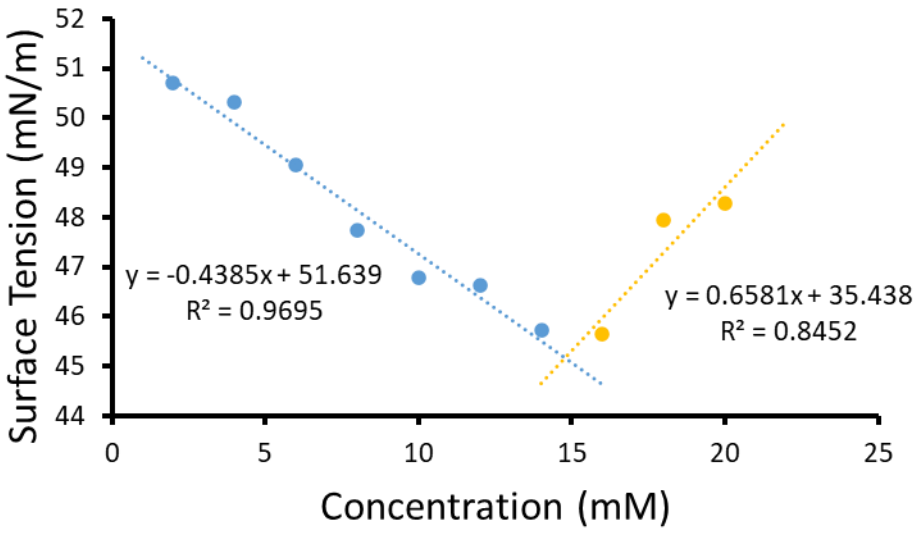

Figure S39 - Calculation of CMC (14.77 mM) for compound **10** in an EtOH:H_2O 1:19 mixture using surface tension measurements.

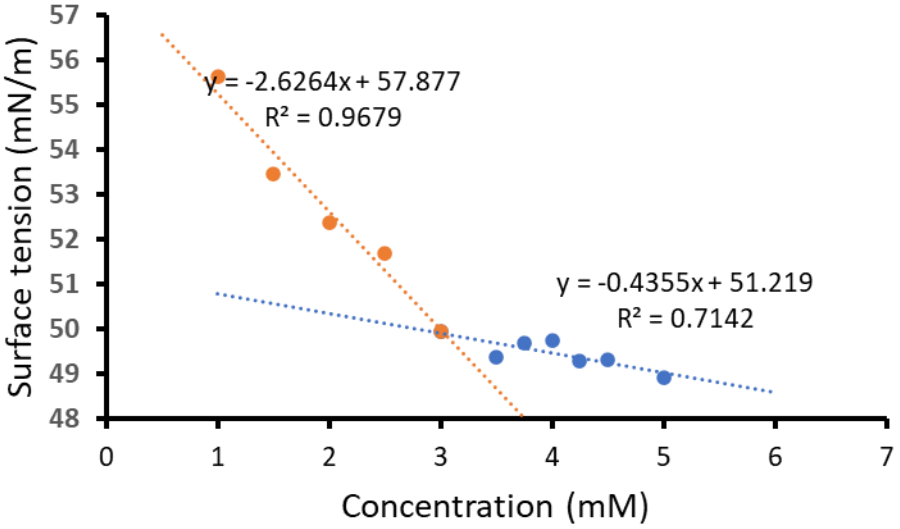

Figure S40 - Calculation of CMC (3.03 mM) for compound **11** in an EtOH:H_2O 1:19 mixture using surface tension measurements.

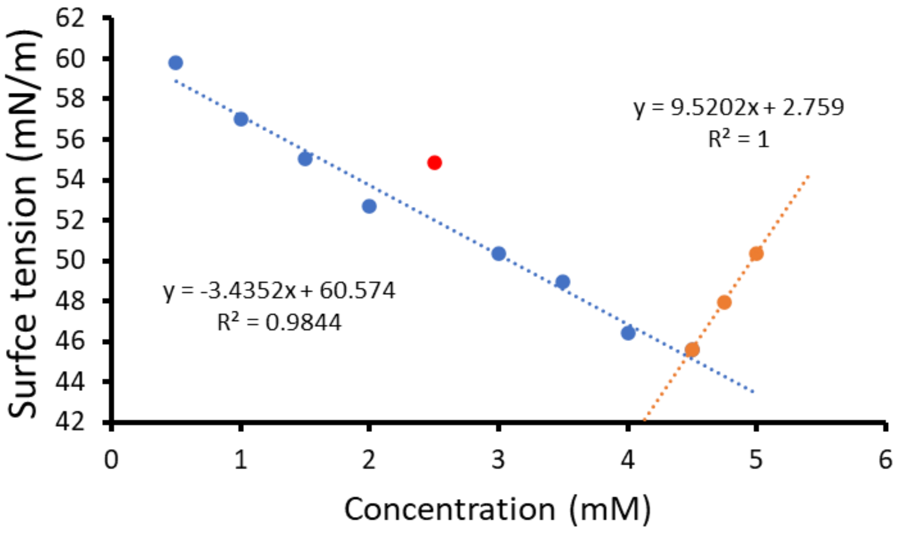

Figure S41 - Calculation of CMC (4.56 mM) for compound **12** in an EtOH:H_2O 1:19 mixture using surface tension measurements.

Section 10: Vesicle Leakage Assay Data

Initial comparative datasets

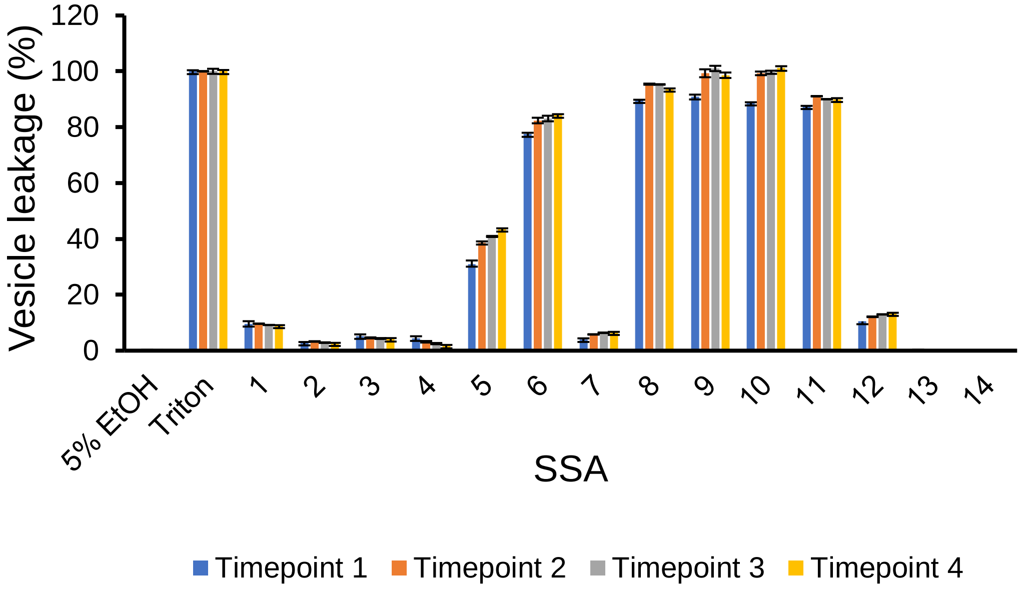

Figure S42 – The percentage lysis of PC lipid membranes (λ_{em} = 515 nm) following the addition of a series of SSAs (1.5 mM). Time points 1-4 refer to 30 secs, 5 mins, 10 mins and 15 mins respectively. Triton X-100 (1%) was used as a positive control for 100 % cell lysis.

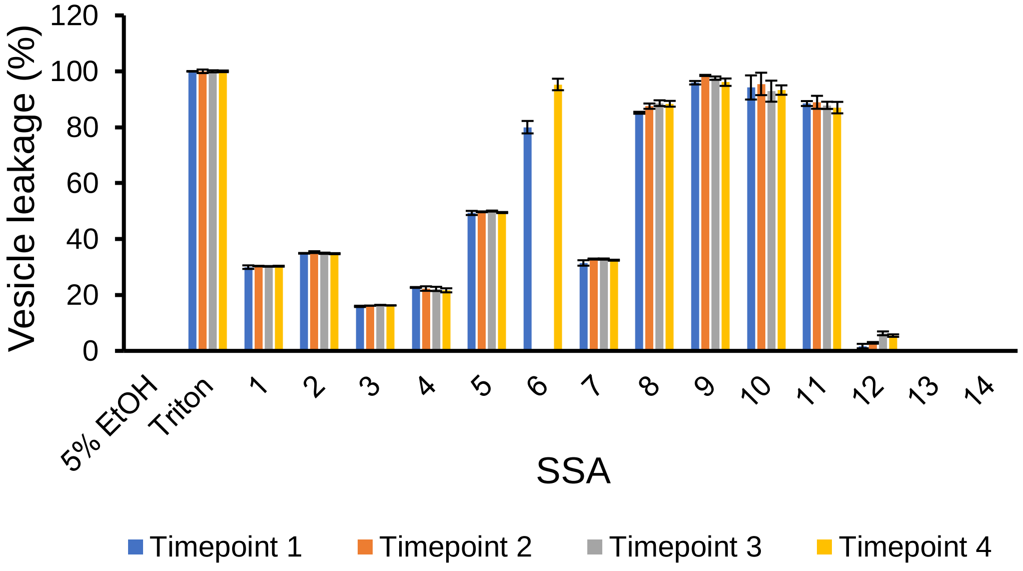

Figure S43 – The percentage lysis of PG lipid membranes (λ_{em} = 515 nm) following the addition of a series of SSAs (1.5 mM). Time points 1-4 refer to 30 secs, 5 mins, 10 mins and 15 mins respectively. Triton X-100 (1%) was used as a positive control for 100 % cell lysis.

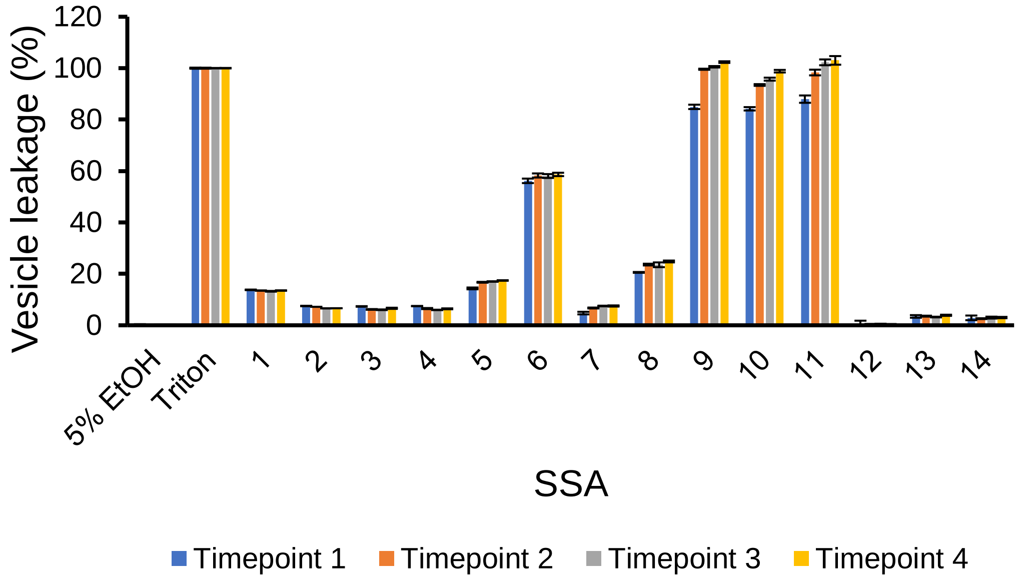

Figure S44 – The percentage lysis of PE:PG 3:1 lipid membranes (λ_{em} = 515 nm) following the addition of a series of SSAs (1.5 mM). Time points 1-4 refer to 30 secs, 5 mins, 10 mins and 15 mins respectively. Triton X-100 (1%) was used as a positive control for 100 % cell lysis.

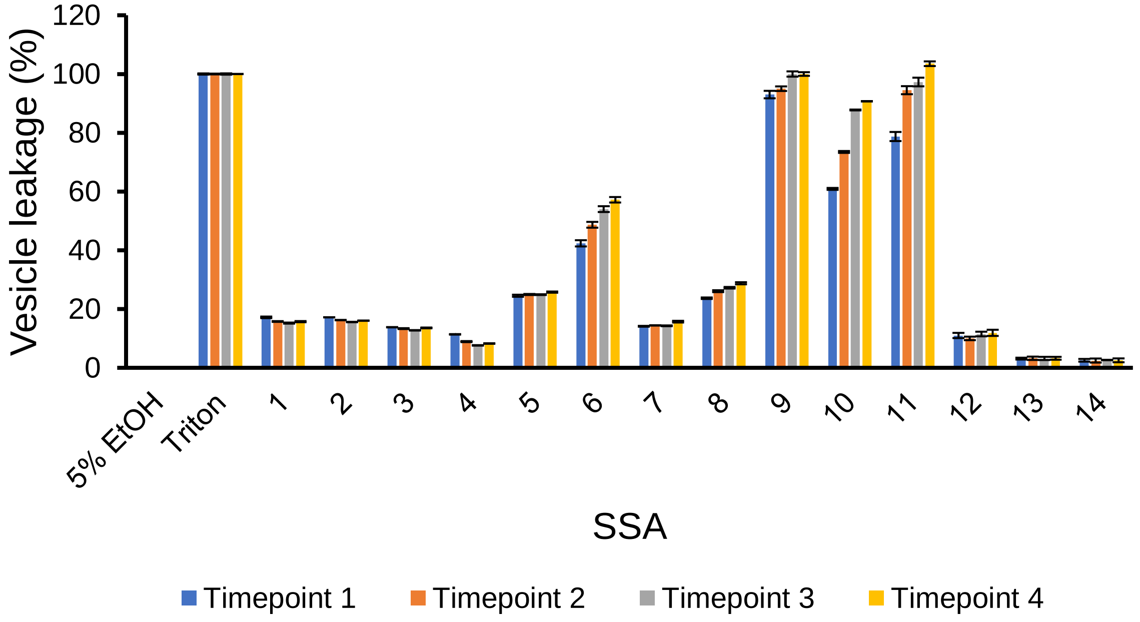

Figure S45 – The percentage lysis of *E. coli* total lipid membranes (λ_{em} = 515 nm) following the addition of a series of SSAs (1.5 mM). Time points 1-4 refer to 30 secs, 5 mins, 10 mins and 15 mins respectively. Triton X-100 (1%) was used as a positive control for 100 % cell lysis.

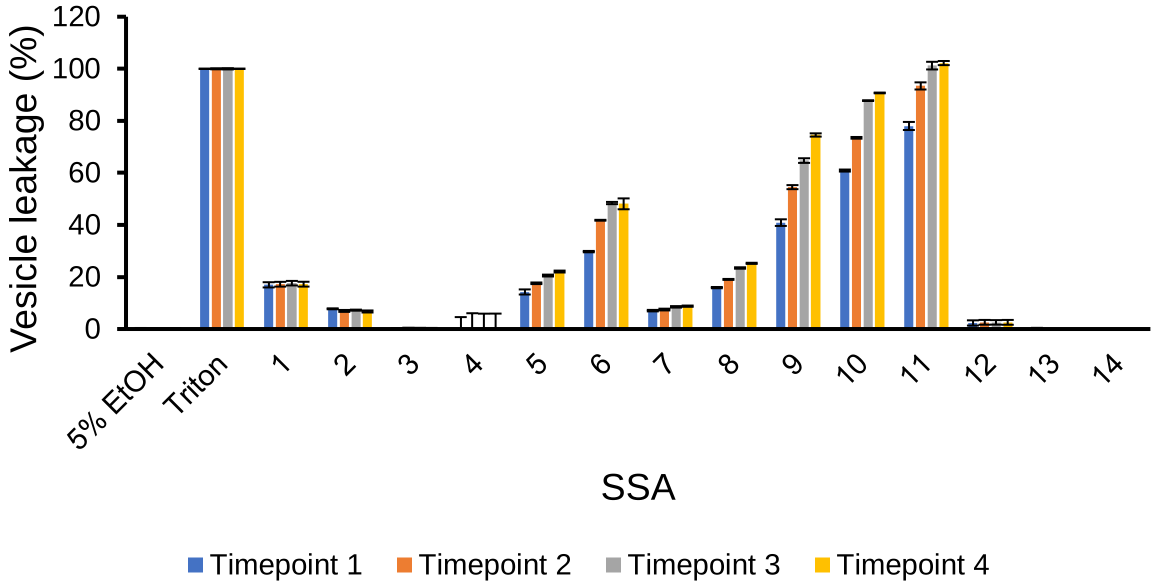

Figure S46 – The percentage lysis of *E. coli* polar lipid membranes (λ_{em} = 515 nm) following the addition of a series of SSAs (1.5 mM). Time points 1-4 refer to 30 secs, 5 mins, 10 mins and 15 mins respectively. Triton X-100 (1%) was used as a positive control for 100 % cell lysis.

PC lipid titrations

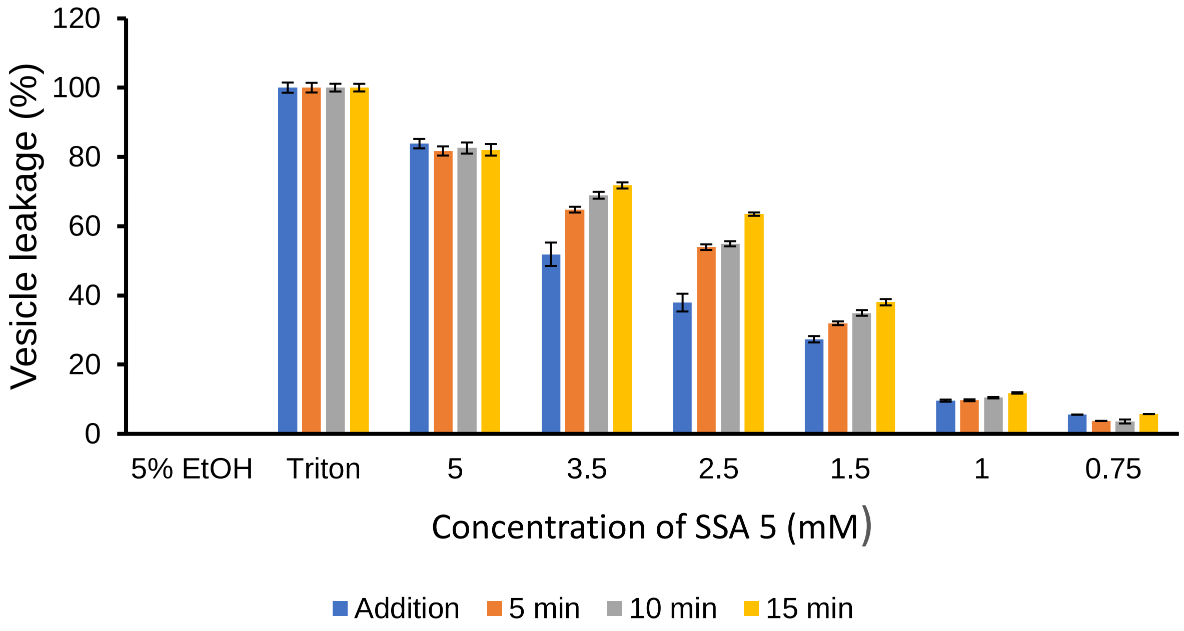

Figure S47 – The percentage lysis of PC lipid membranes (λ_{em} = 515 nm) following the addition of SSA **5** at varying concentrations. Triton X-100 (1%) was used as a positive control for 100 % cell lysis.

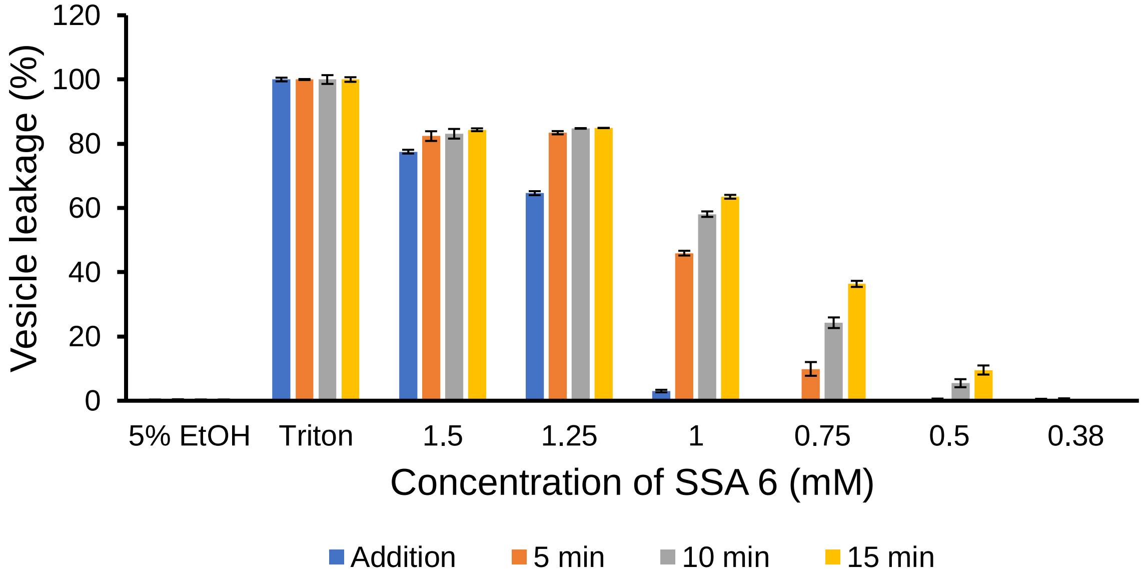

Figure S48 – The percentage lysis of PC lipid membranes (λ_{em} = 515 nm) following the addition of SSA **6** at varying concentrations. Triton X-100 (1%) was used as a positive control for 100 % cell lysis.

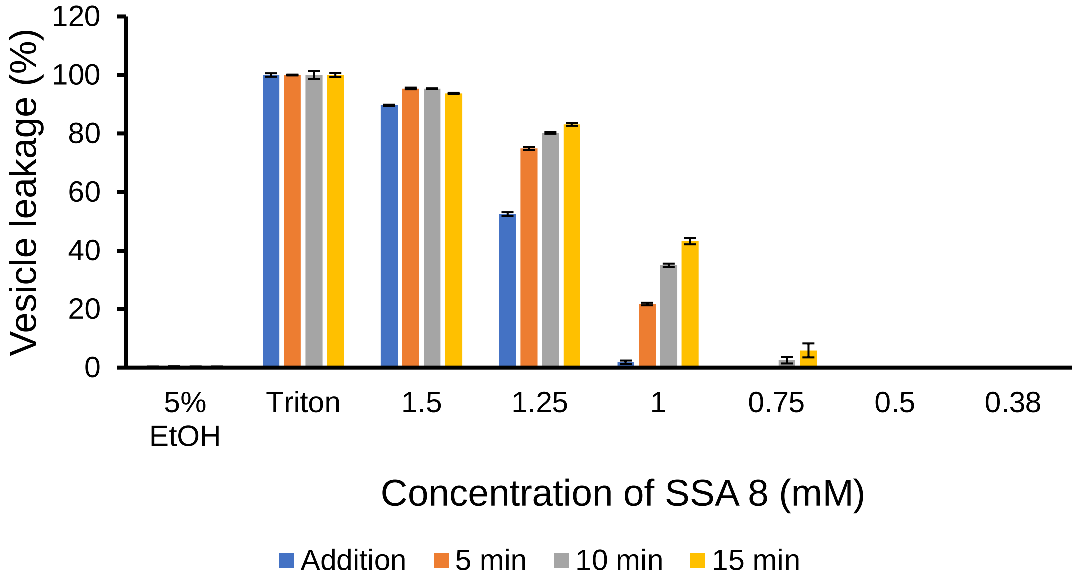

Figure S49 – The percentage lysis of PC lipid membranes (λ_{em} = 515 nm) following the addition of SSA **8** at varying concentrations. Triton X-100 (1%) was used as a positive control for 100 % cell lysis.

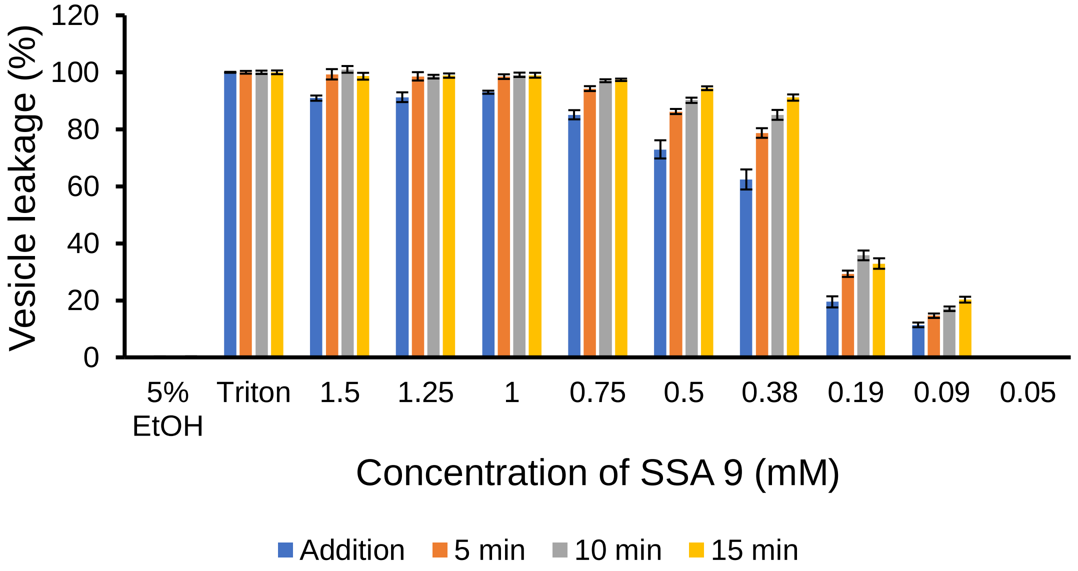

Figure S50 – The percentage lysis of PC lipid membranes (λ_{em} = 515 nm) following the addition of SSA **9** at varying concentrations. Triton X-100 (1%) was used as a positive control for 100 % cell lysis.

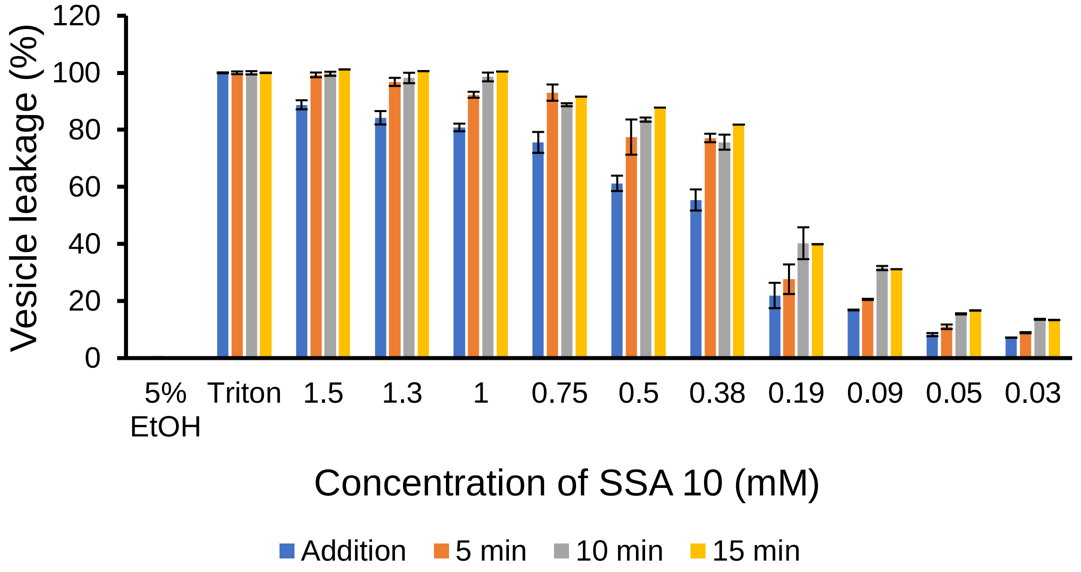

Figure S51 – The percentage lysis of PC lipid membranes (λ_{em} = 515 nm) following the addition of SSA **10** at varying concentrations. Triton X-100 (1%) was used as a positive control for 100 % cell lysis.

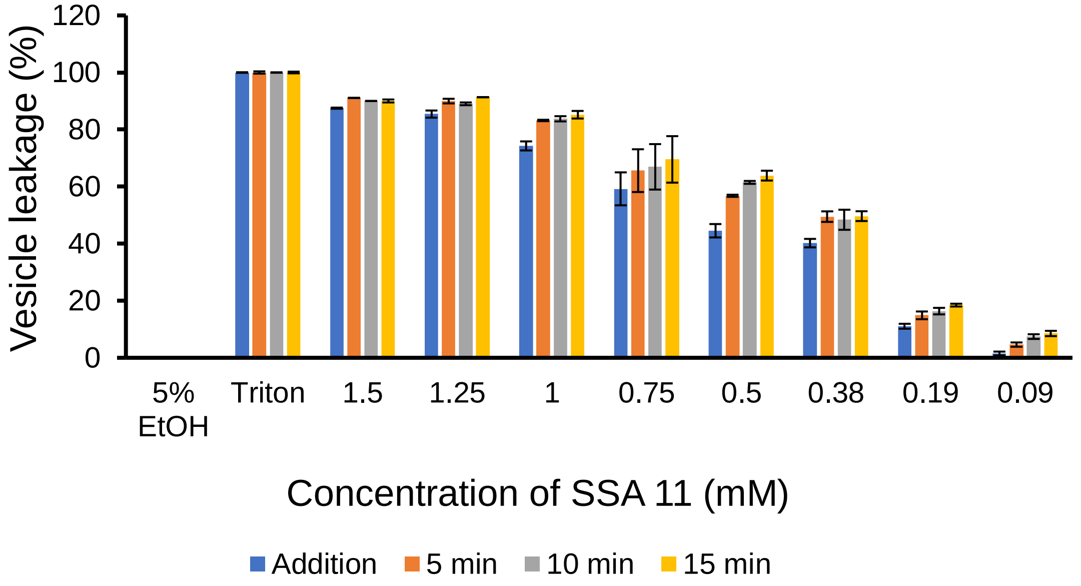

Figure S52 – The percentage lysis of PC lipid membranes (λ_{em} = 515 nm) following the addition of SSA **11** at varying concentrations. Triton X-100 (1%) was used as a positive control for 100 % cell lysis.

PG lipid titrations

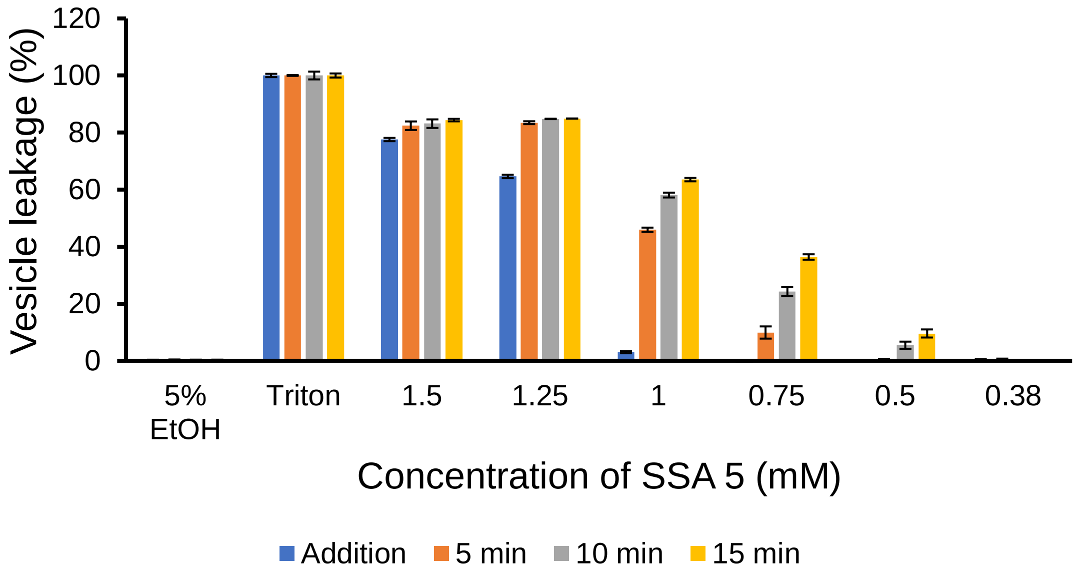

Figure S53 – The percentage lysis of PG lipid membranes (λ_{em} = 515 nm) following the addition of SSA **5** at varying concentrations. Triton X-100 (1%) was used as a positive control for 100 % cell lysis.

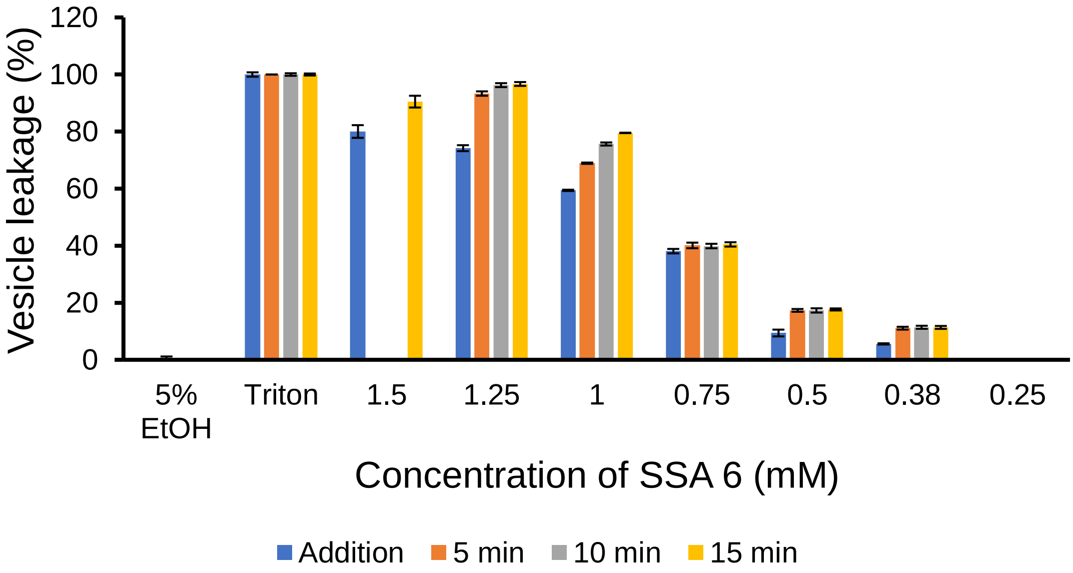

Figure S54 – The percentage lysis of PG lipid membranes (λ_{em} = 515 nm) following the addition of SSA **6** at varying concentrations. Triton X-100 (1%) was used as a positive control for 100 % cell lysis.

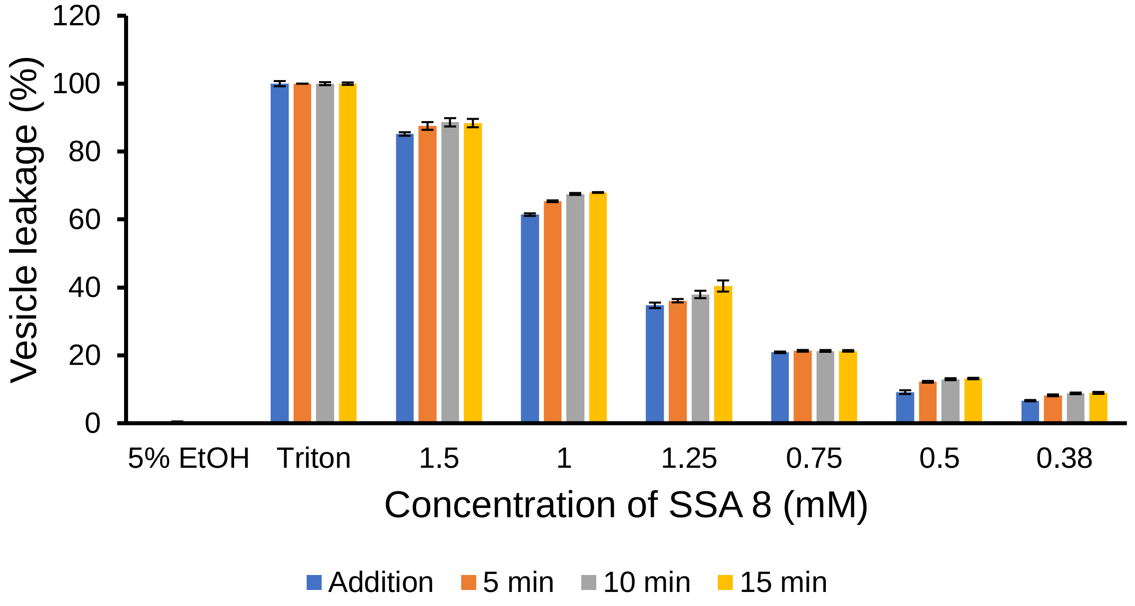

Figure S55 – The percentage lysis of PG lipid membranes (λ_{em} = 515 nm) following the addition of SSA **8** at varying concentrations. Triton X-100 (1%) was used as a positive control for 100 % cell lysis.

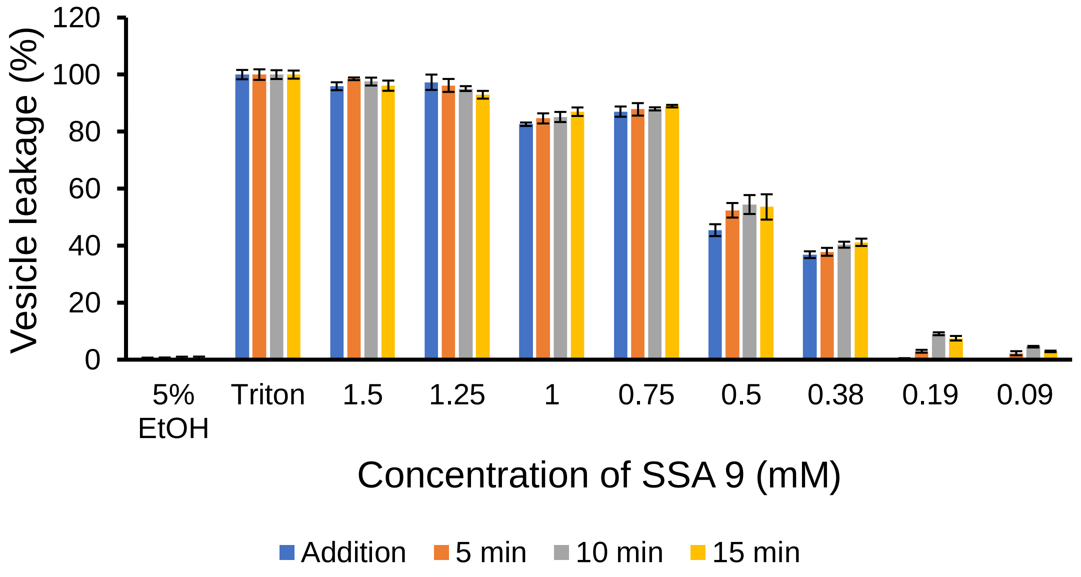

Figure S56 – The percentage lysis of PG lipid membranes (λ_{em} = 515 nm) following the addition of SSA **9** at varying concentrations. Triton X-100 (1%) was used as a positive control for 100 % cell lysis.

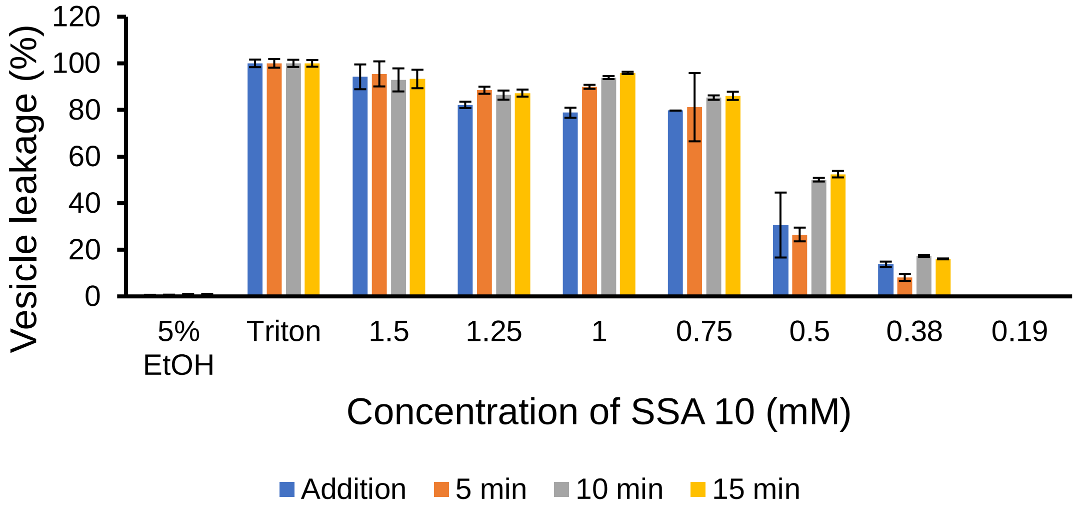

Figure S57 – The percentage lysis of PG lipid membranes (λ_{em} = 515 nm) following the addition of SSA **10** at varying concentrations. Triton X-100 (1%) was used as a positive control for 100 % cell lysis.

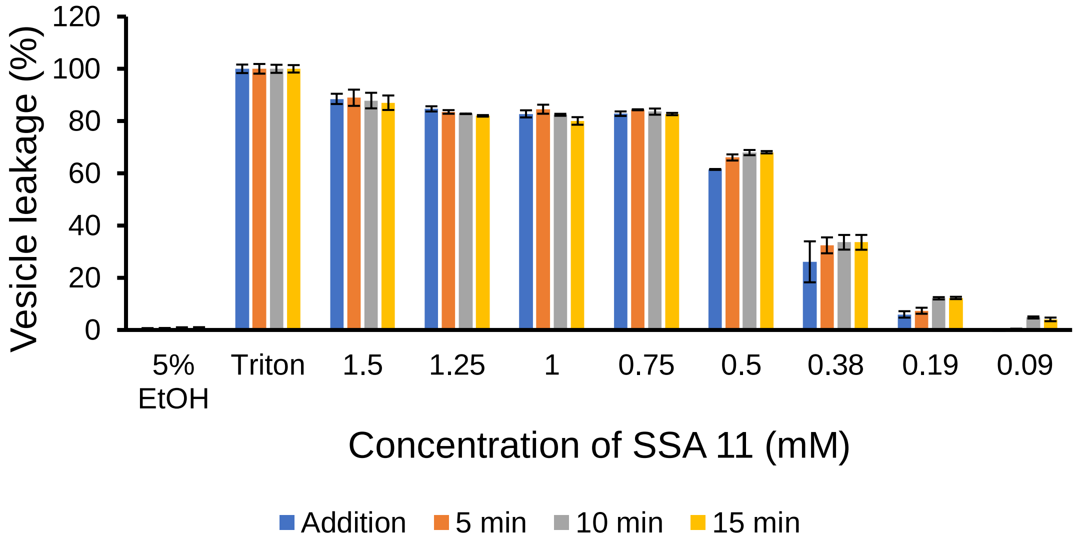

Figure S58 – The percentage lysis of PG lipid membranes (λ_{em} = 515 nm) following the addition of SSA **11** at varying concentrations. Triton X-100 (1%) was used as a positive control for 100 % cell lysis.

PE:PG 3:1 lipid titrations

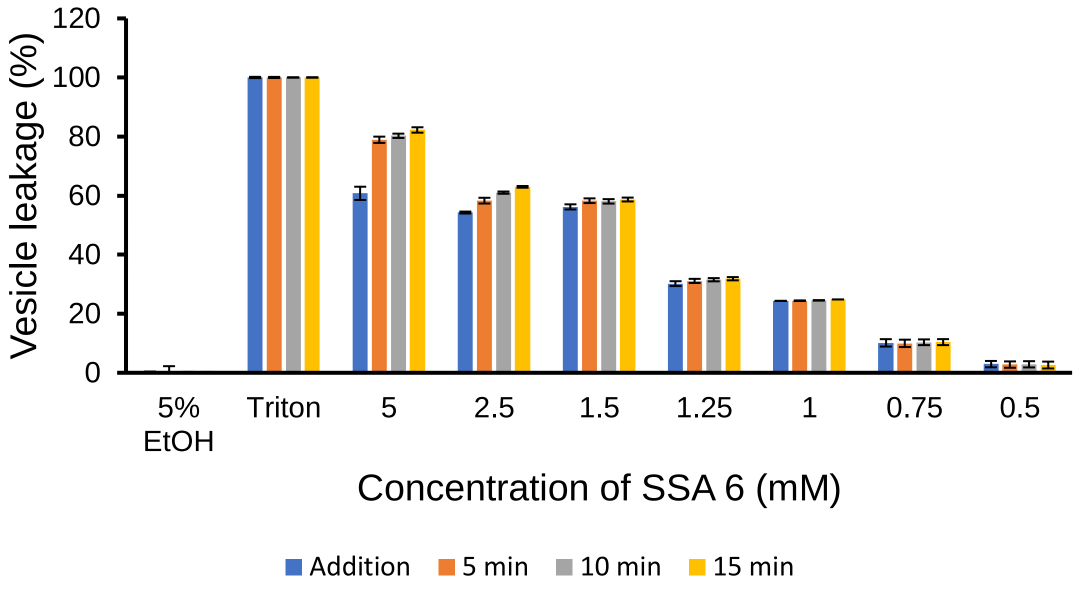

Figure S59 – The percentage lysis of PE:PG 3:1 lipid membranes (λ_{em} = 515 nm) following the addition of SSA **6** at varying concentrations. Triton X-100 (1%) was used as a positive control for 100 % cell lysis.

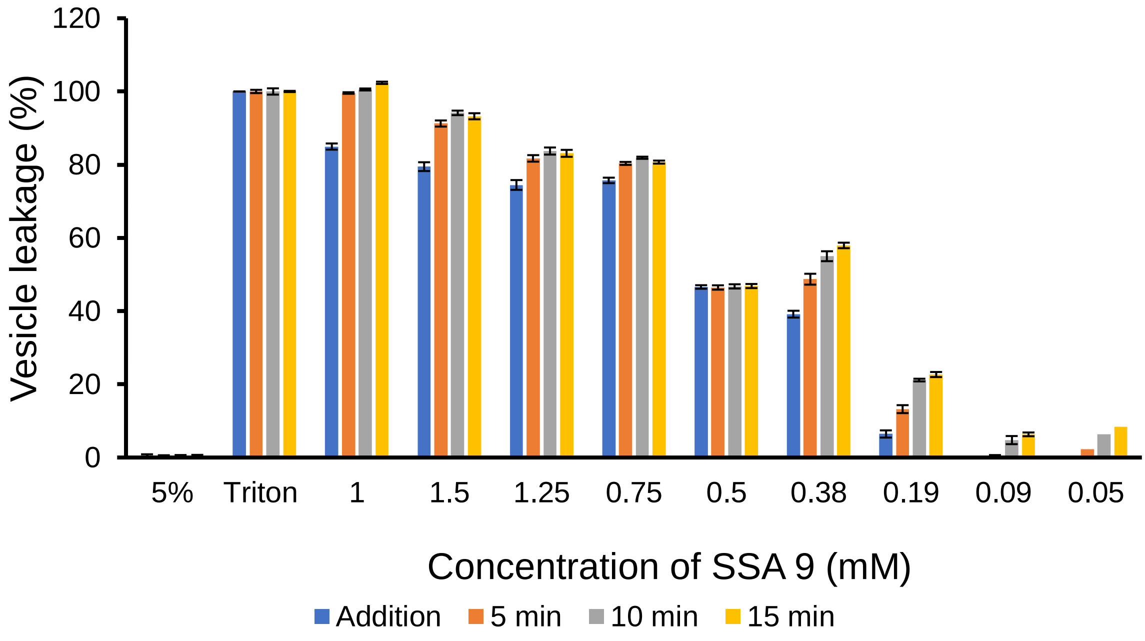

Figure S60 – The percentage lysis of PE:PG 3:1 lipid membranes (λ_{em} = 515 nm) following the addition of SSA **9** at varying concentrations. Triton X-100 (1%) was used as a positive control for 100 % cell lysis.

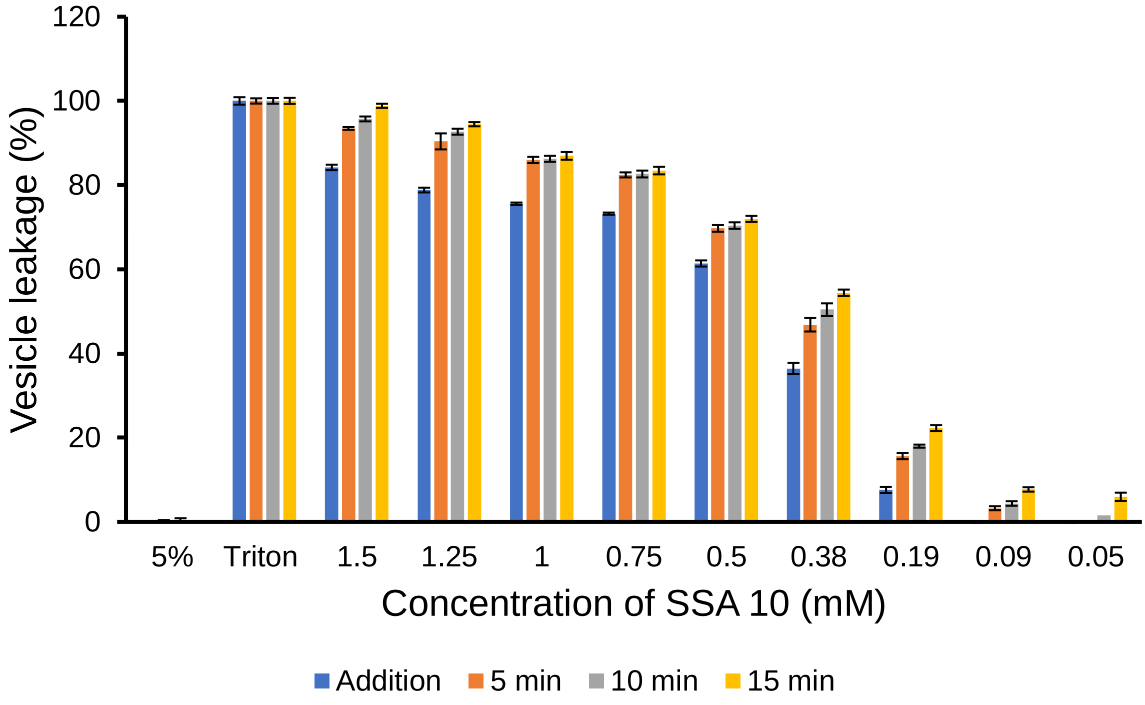

Figure S61 – The percentage lysis of PE:PG 3:1 lipid membranes (λ_{em} = 515 nm) following the addition of SSA **10** at varying concentrations. Triton X-100 (1%) was used as a positive control for 100 % cell lysis.

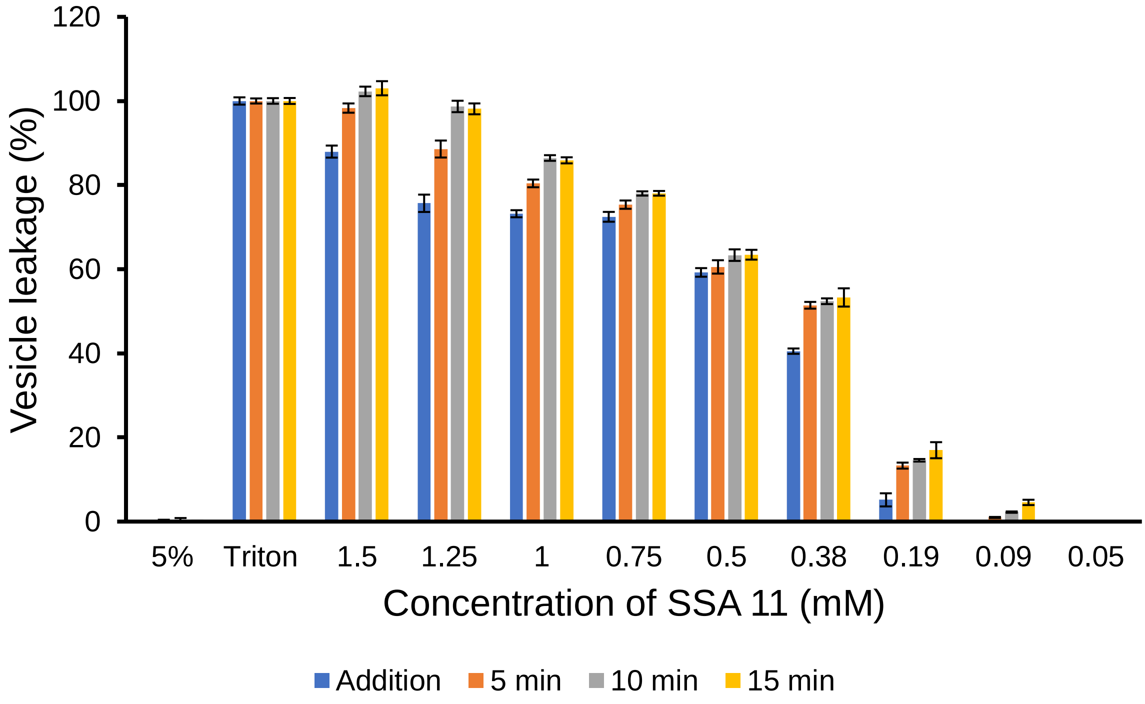

Figure S62 – The percentage lysis of PE:PG 3:1 lipid membranes (λ_{em} = 515 nm) following the addition of SSA **11** at varying concentrations. Triton X-100 (1%) was used as a positive control for 100 % cell lysis.

E. coli total lipid titrations

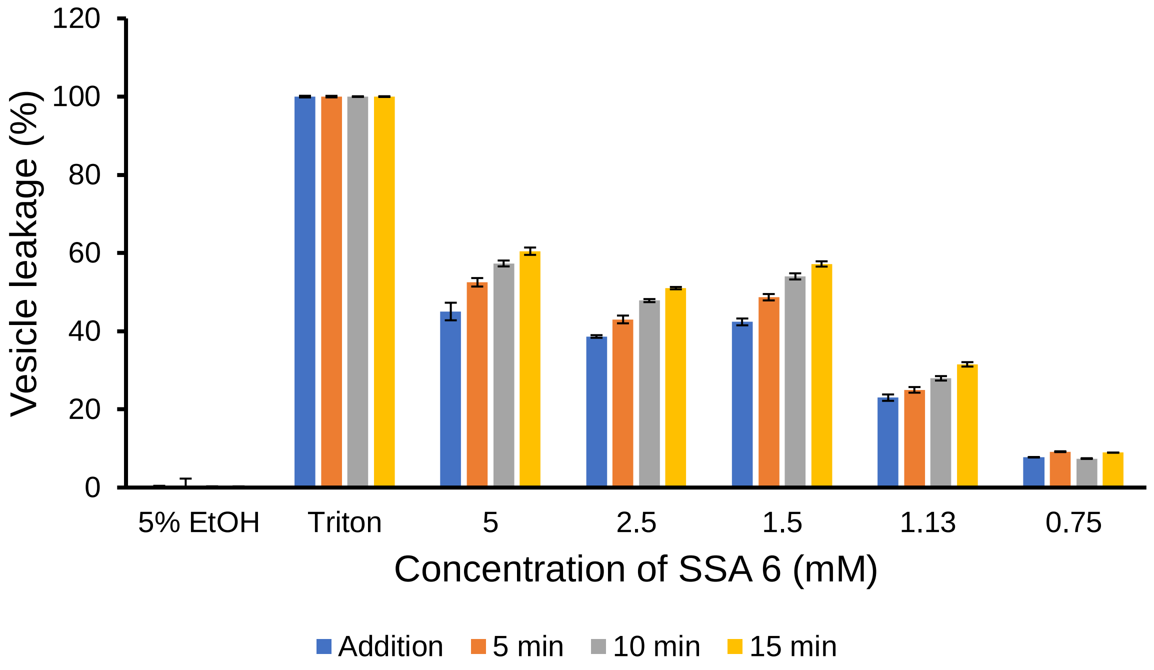

Figure S63 – The percentage lysis of *E. coli* total lipid membranes (λ_{em} = 515 nm) following the addition of SSA **6** at varying concentrations. Triton X-100 (1%) was used as a positive control for 100 % cell lysis.

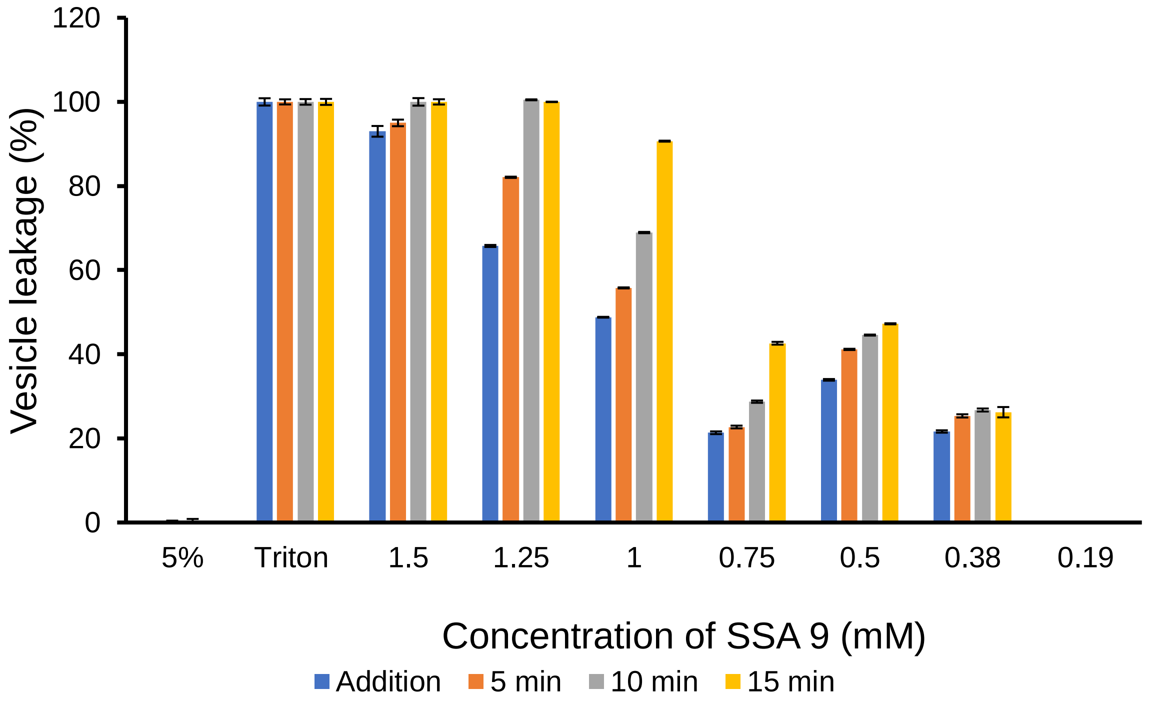

Figure S64 – The percentage lysis of *E. coli* total lipid membranes (λ_{em} = 515 nm) following the addition of SSA **9** at varying concentrations. Triton X-100 (1%) was used as a positive control for 100 % cell lysis.

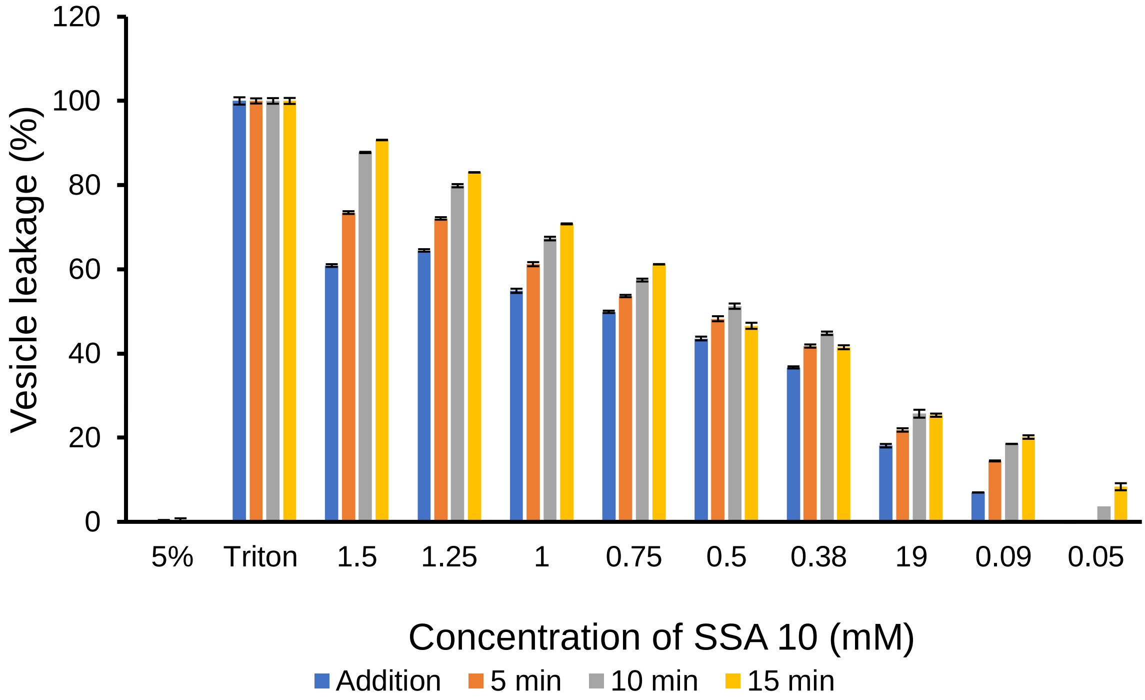

Figure S65 – The percentage lysis of *E. coli* total lipid membranes (λ_{em} = 515 nm) following the addition of SSA **10** at varying concentrations. Triton X-100 (1%) was used as a positive control for 100 % cell lysis.

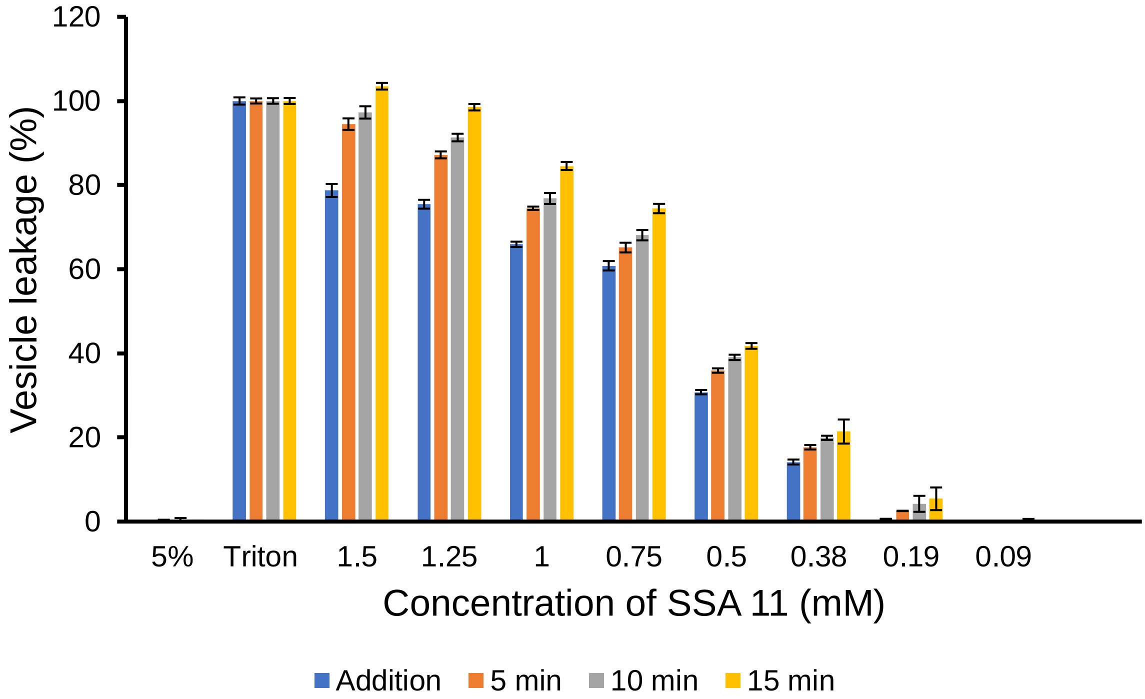

Figure S66 – The percentage lysis of *E. coli* total lipid membranes (λ_{em} = 515 nm) following the addition of SSA **11** at varying concentrations. Triton X-100 (1%) was used as a positive control for 100 % cell lysis.

E. coli polar lipid titrations

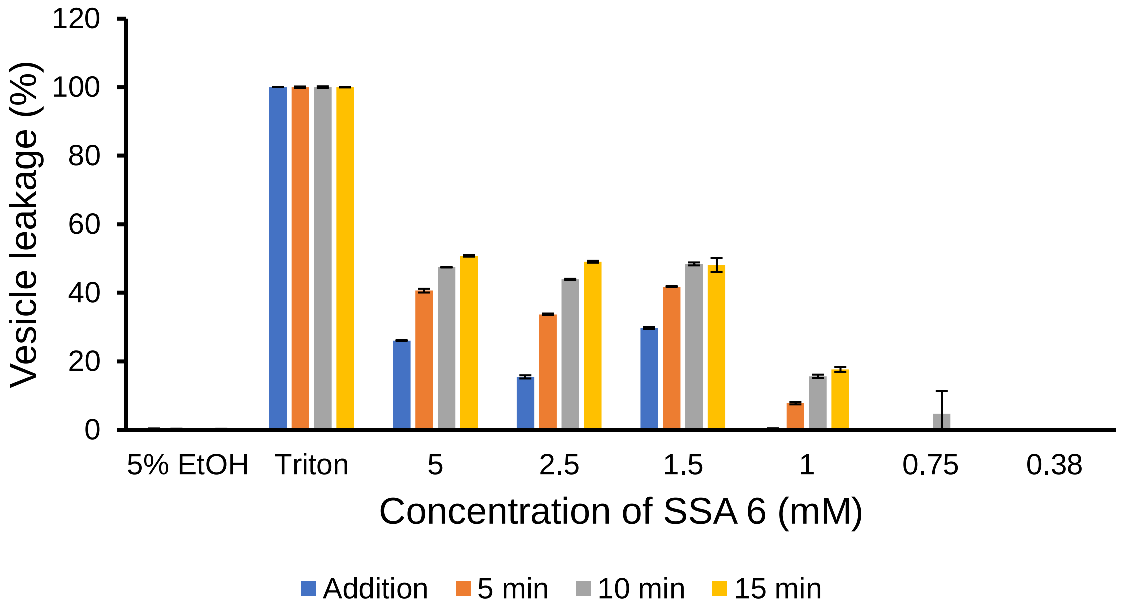

Figure S67 – The percentage lysis of *E. coli* polar lipid membranes (λ_{em} = 515 nm) following the addition of SSA **6** at varying concentrations. Triton X-100 (1%) was used as a positive control for 100 % cell lysis.

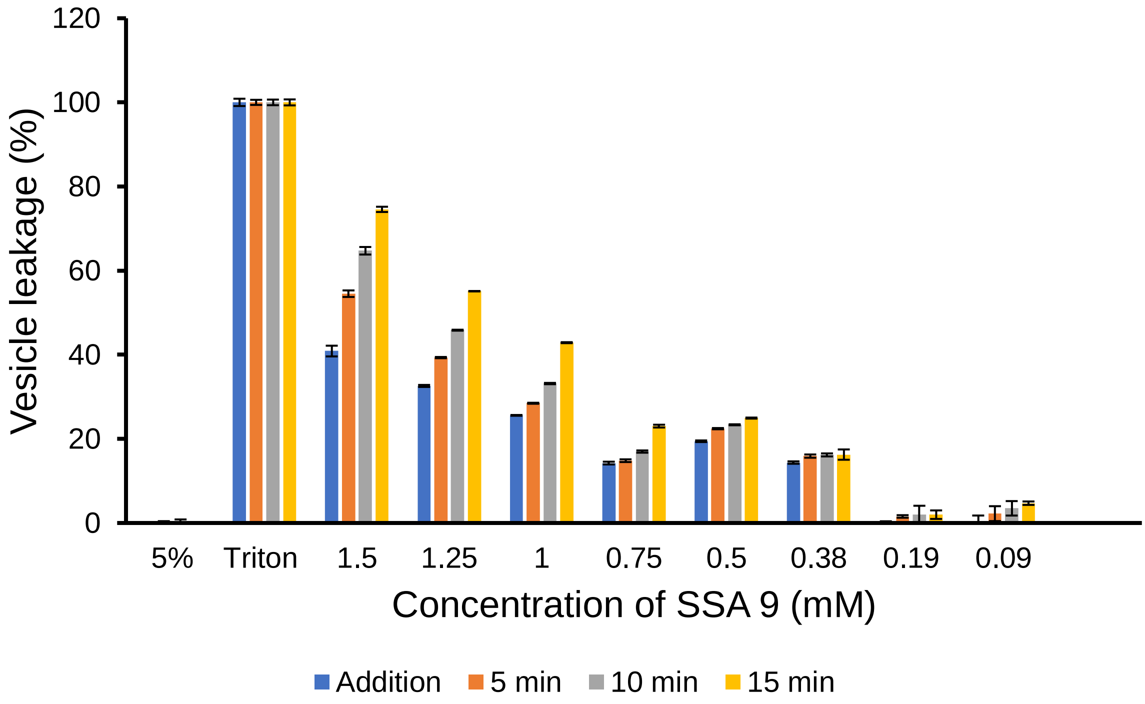

Figure S68 – The percentage lysis of *E. coli* polar lipid membranes (λ_{em} = 515 nm) following the addition of SSA **9** at varying concentrations. Triton X-100 (1 %) was used as a positive control for 100 % cell lysis.

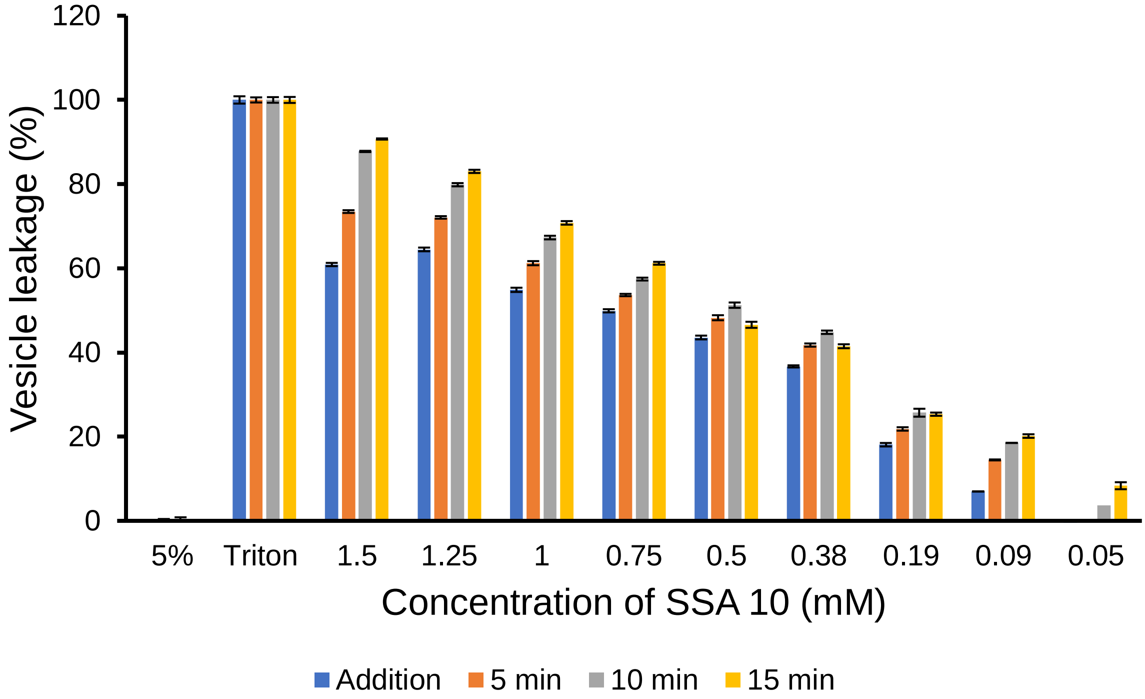

Figure S69 – The percentage lysis of *E. coli* polar lipid membranes (λ_{em} = 515 nm) following the addition of SSA **10** at varying concentrations. Triton X-100 (1 %) was used as a positive control for 100 % cell lysis.

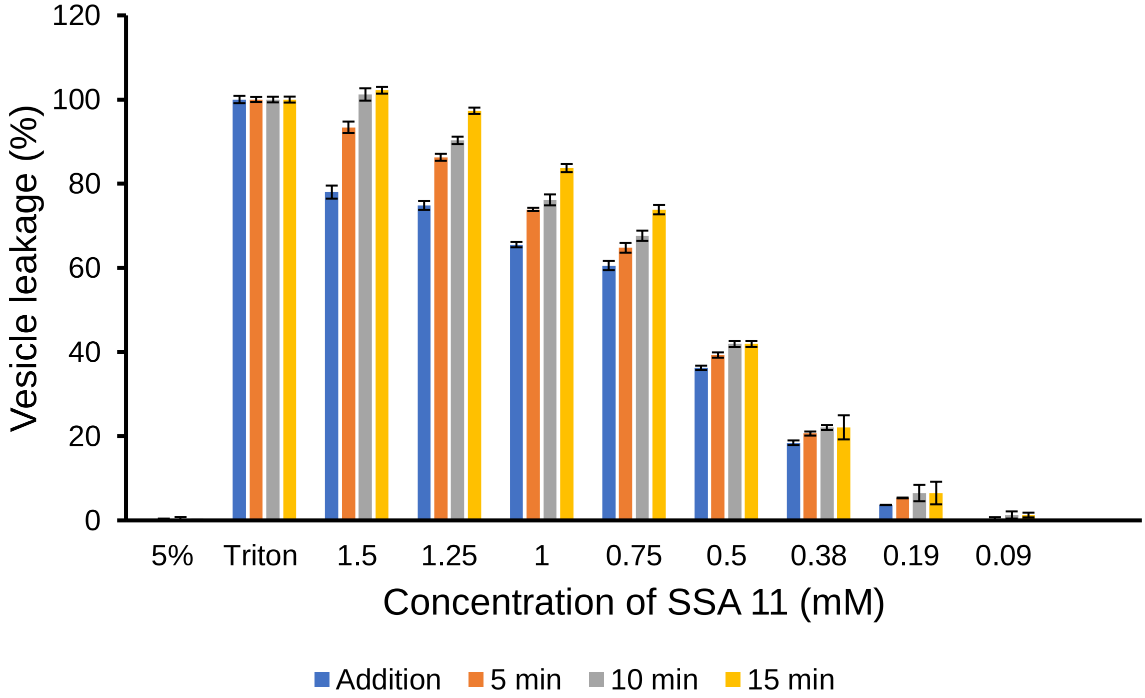

Figure S70 – The percentage lysis of *E. coli* polar lipid membranes (λ_{em} = 515 nm) following the addition of SSA **11** at varying concentrations. Triton X-100 (1 %) was used as a positive control for 100 % cell lysis.

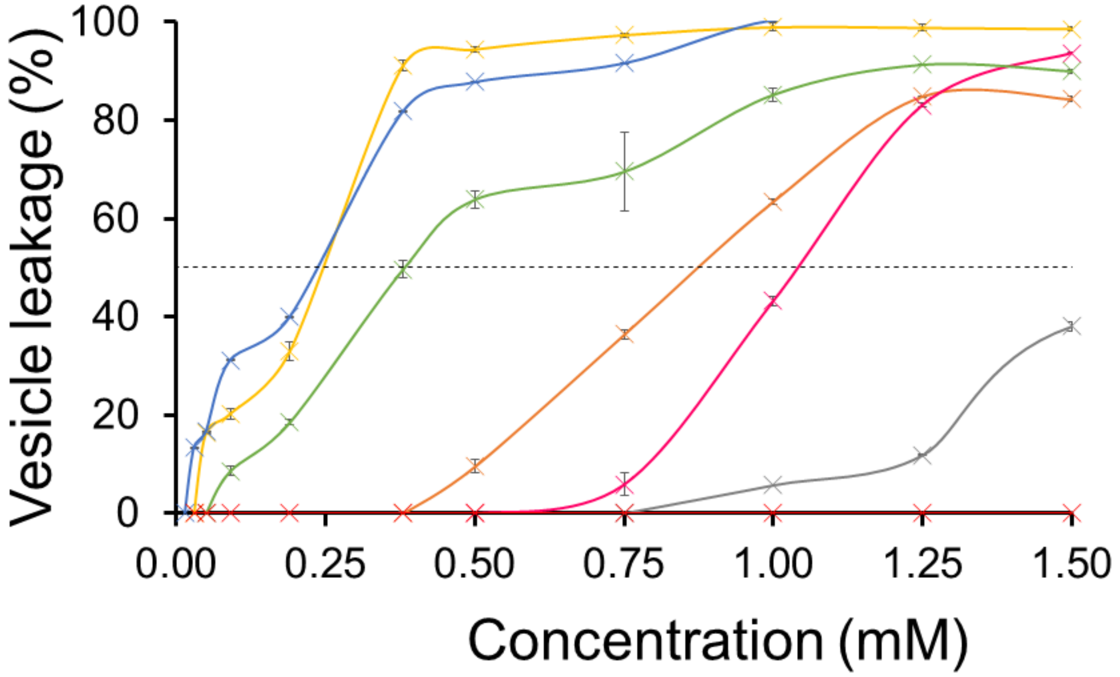

Figure S71 - The percentage lysis of PC lipid membranes (30 µM) following addition of SSA **5** (grey), SSA **6** (orange), SSA **8** (pink), SSA **9** (yellow), SSA **10** (blue), SSA **11** (green) and control solution 5 % EtOH (red) after 15 mins.

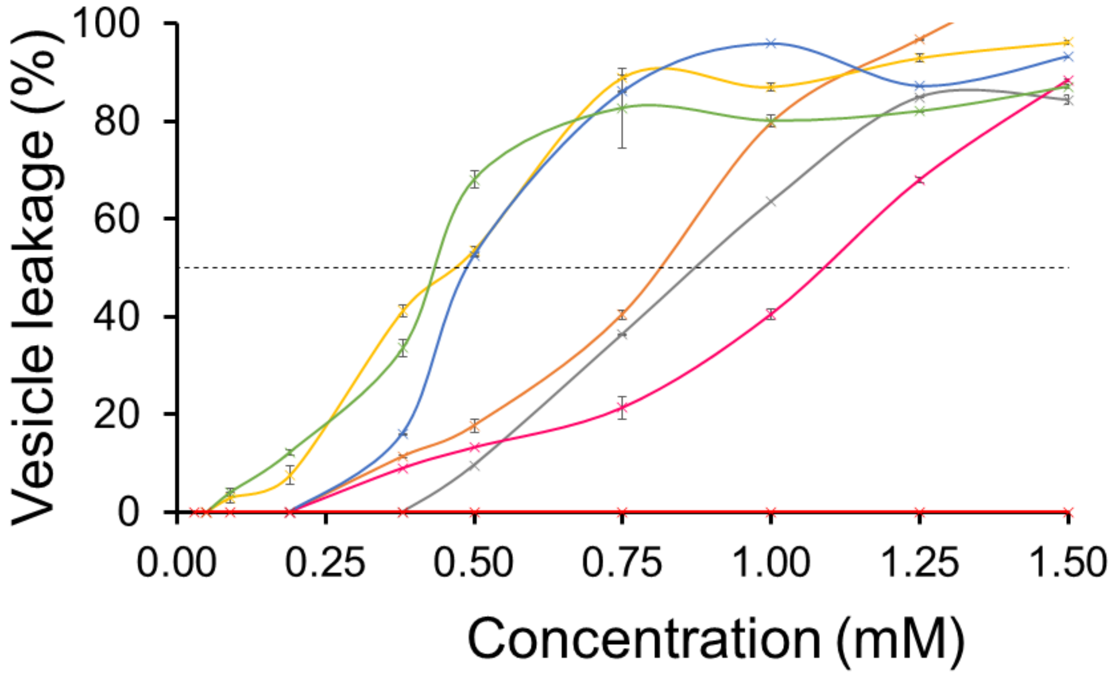

Figure S72 - The percentage lysis of PG lipid membranes (30 µM) following addition of SSA **5** (grey), SSA **6** (orange), SSA **8** (pink), SSA **9** (yellow), SSA **10** (blue), SSA **11** (green) and control solution 5 % EtOH (red) after 15 mins.

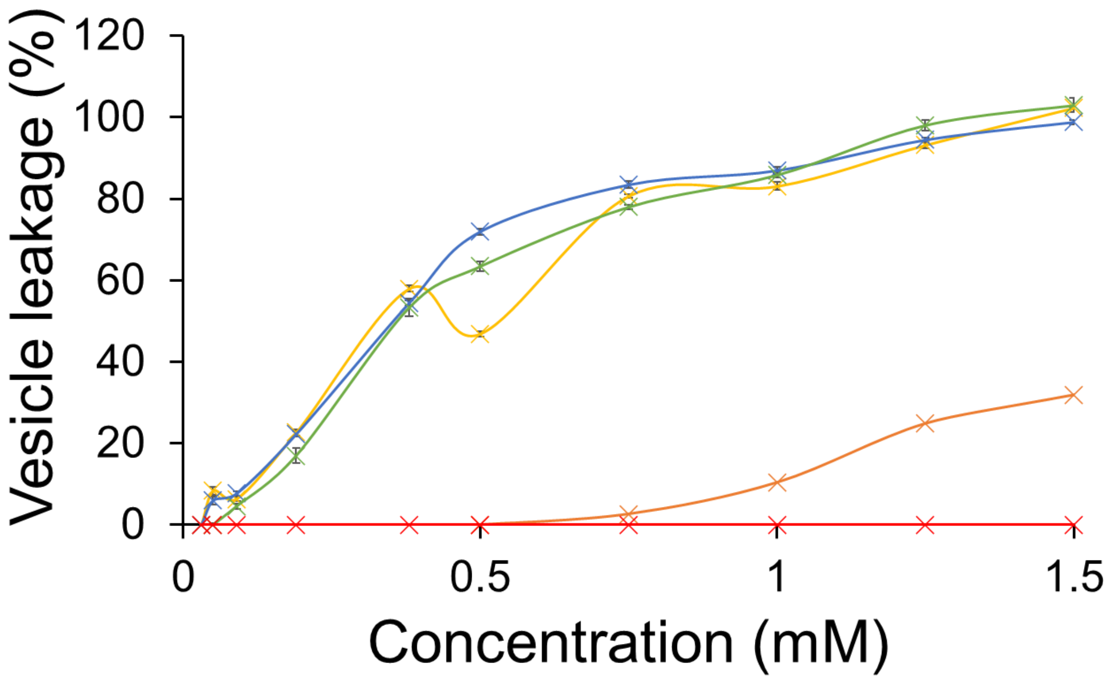

Figure S73 - The percentage lysis of PE-PG mix lipid membranes (30 µM) following addition of SSA **5** (grey), SSA **6** (orange), SSA **8** (pink), SSA **9** (yellow), SSA **10** (blue), SSA **11** (green) and control solution 5 % EtOH (red) after 15 mins.

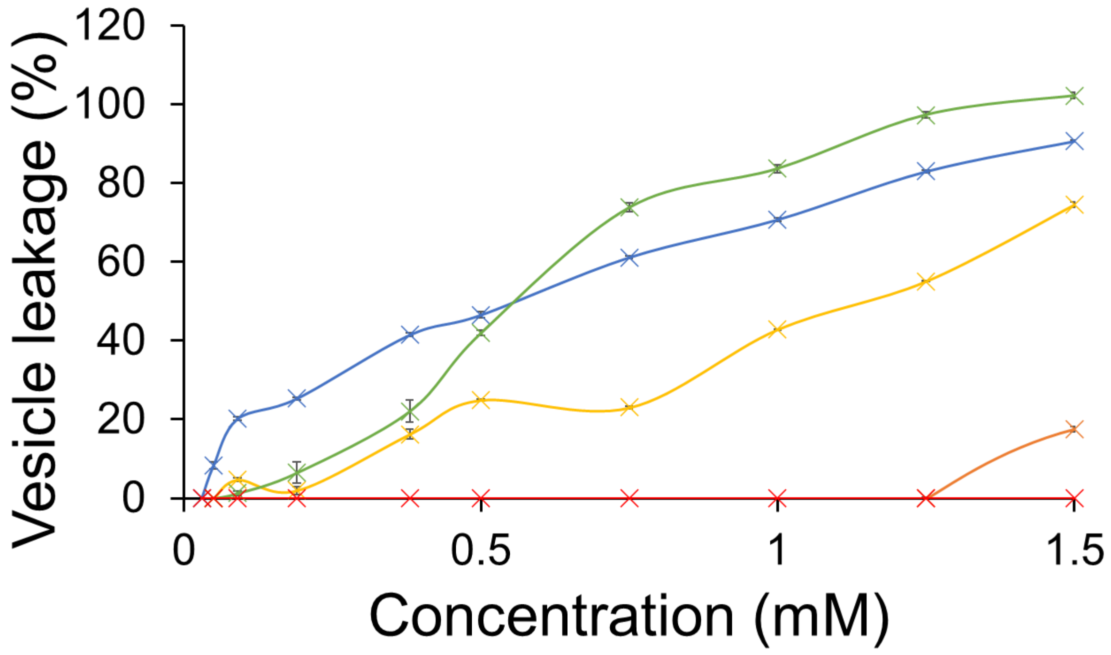

Figure S74 - The percentage lysis of E. coli polar mix lipid membranes (30 µM) following addition of SSA **5** (grey), SSA **6** (orange), SSA **8** (pink), SSA **9** (yellow), SSA **10** (blue), SSA **11** (green) and control solution 5 % EtOH (red) after 15 mins.

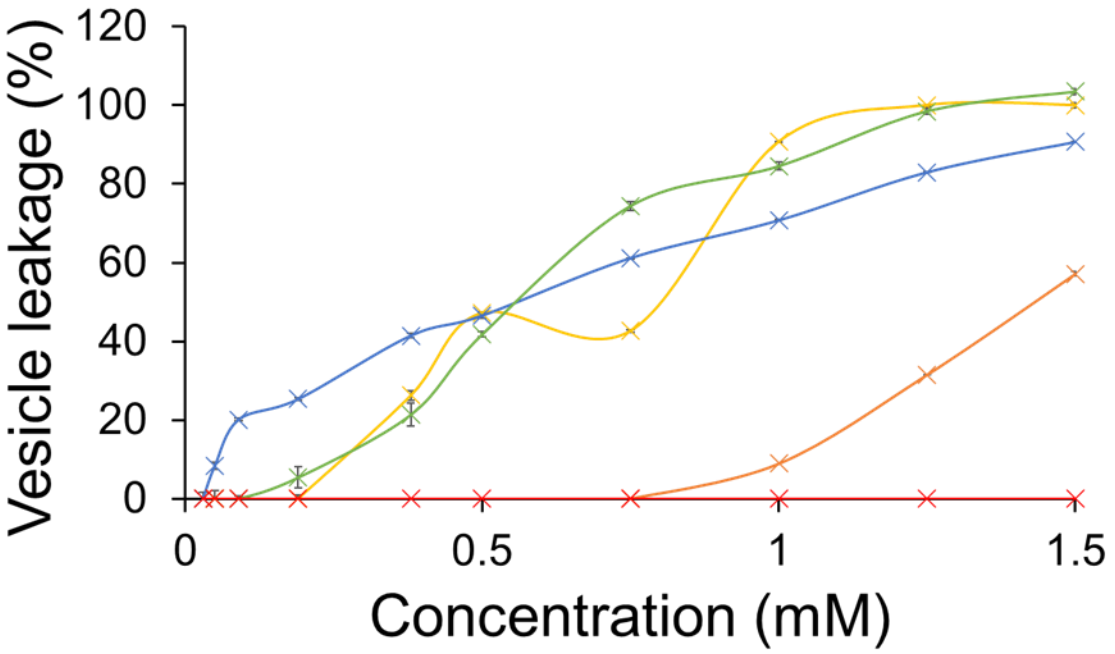

Figure S75 - The percentage lysis of E. coli total mix lipid membranes (30 µM) following addition of SSA **5** (grey), SSA **6** (orange), SSA **8** (pink), SSA **9** (yellow), SSA **10** (blue), SSA **11** (green) and control solution 5 % EtOH (red) after 15 mins.

Section 11: Membrane Fluidity Assay Data

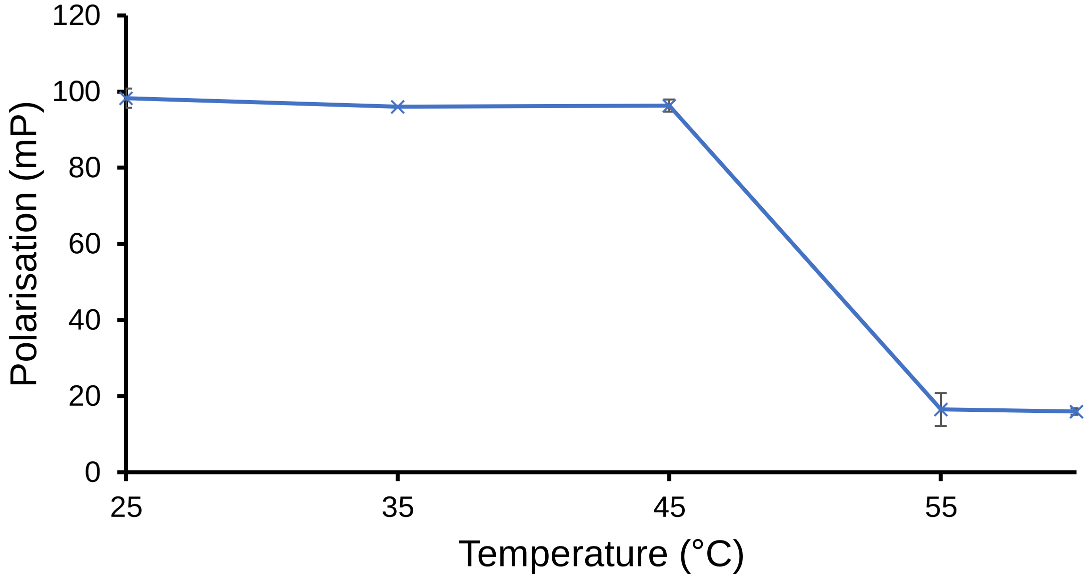

Figure S76 – Effect of temperature on FP measurements in DPH-labelled DSPG vesicles. A target FP value of 100 mP was set to the DPH-labelled vesicles at 25 °C.

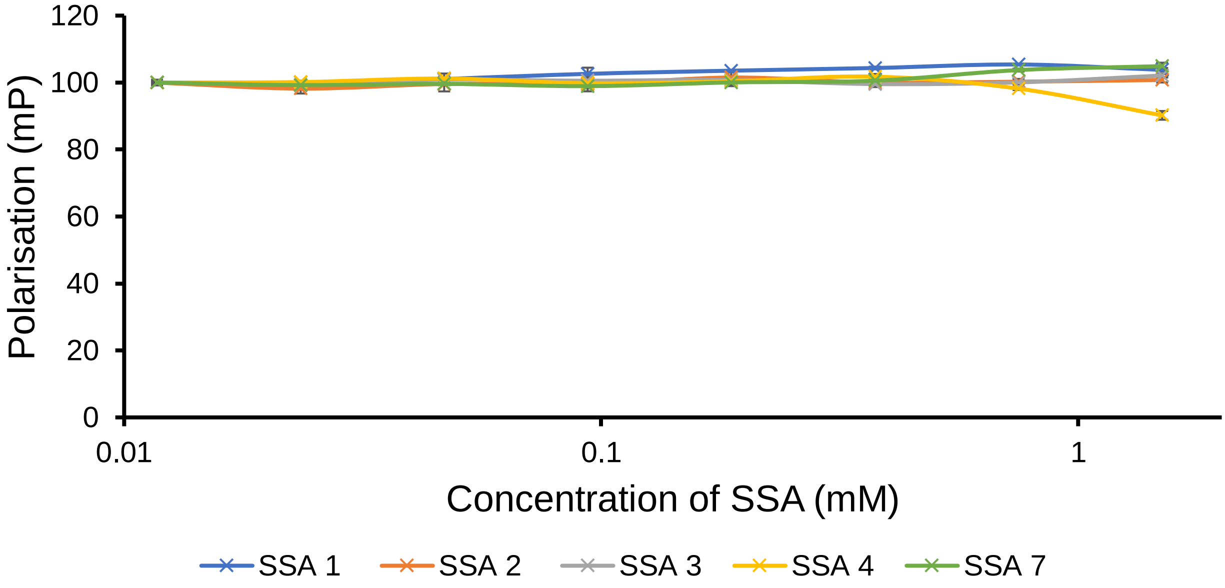

Figure S77 – Effect of SSA on FP measured in DPH-labelled PC vesicles at 25 °C. A target FP value of 100 mP was set to the DPH-labelled vesicles.

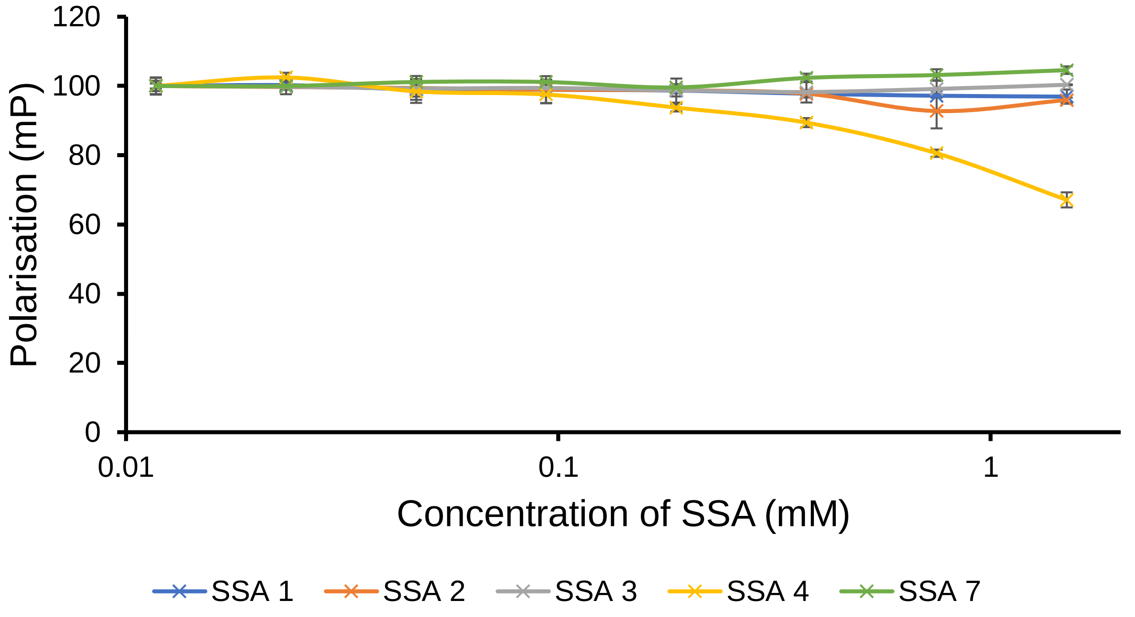

Figure S78 – Effect of SSA on FP measured in DPH-labelled PG vesicles at 25 °C. A target FP value of 100 mP was set to the DPH-labelled vesicles.

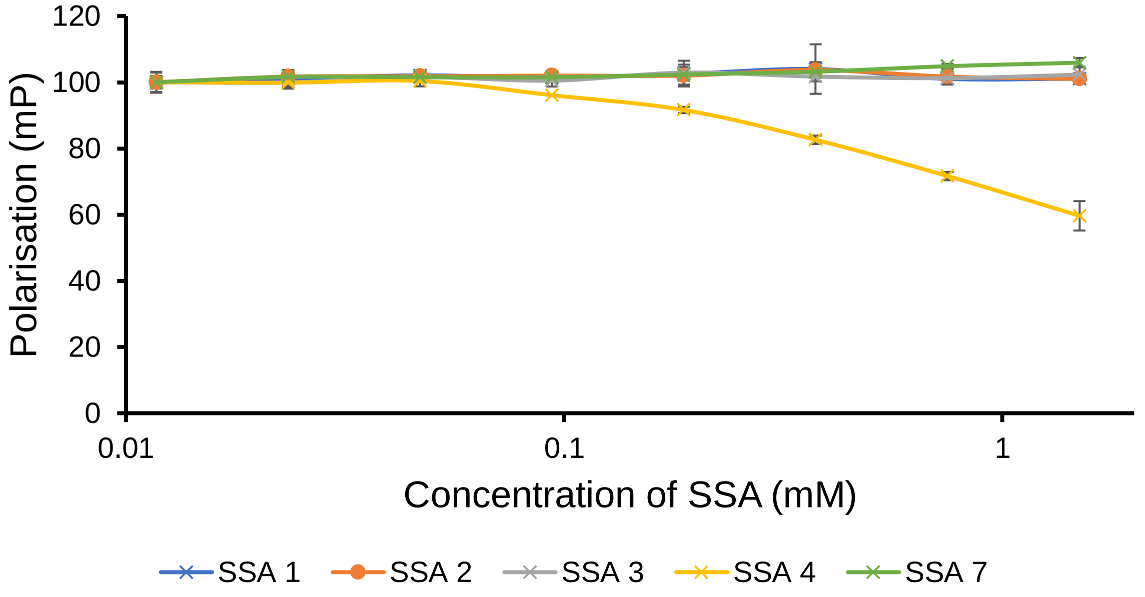

Figure S79 – Effect of SSA on FP measured in DPH-labelled PE:PG 3:1 vesicles at 25 °C. A target FP value of 100 mP was set to the DPH-labelled vesicles.

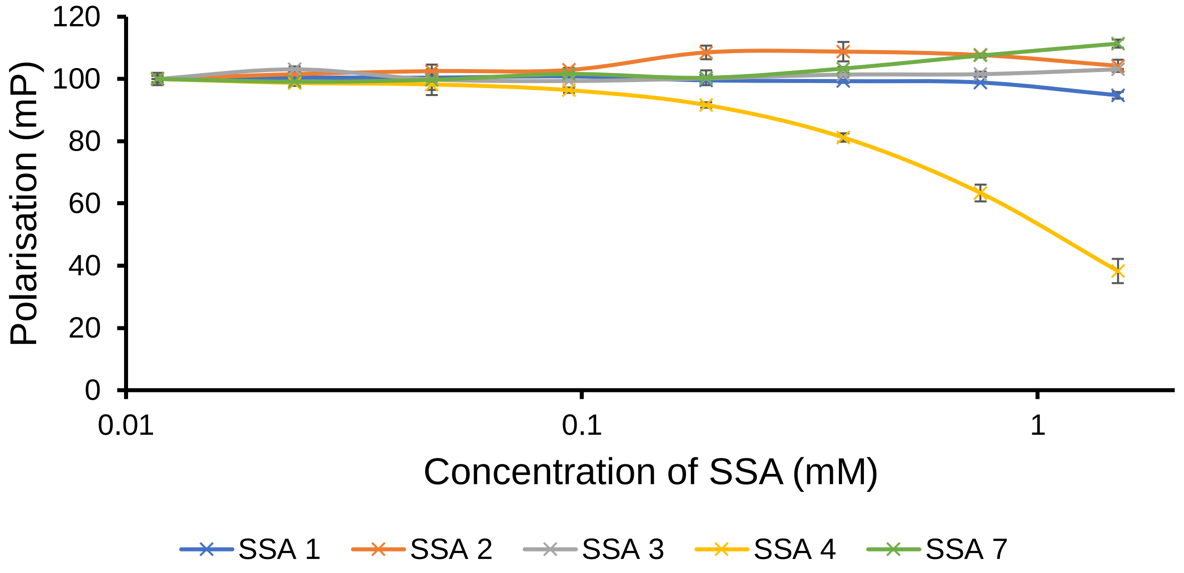

Figure S80 – Effect of SSA on FP measured in DPH-labelled *E. coli* total vesicles at 25 °C. A target FP value of 100 mP was set to the DPH-labelled vesicles.

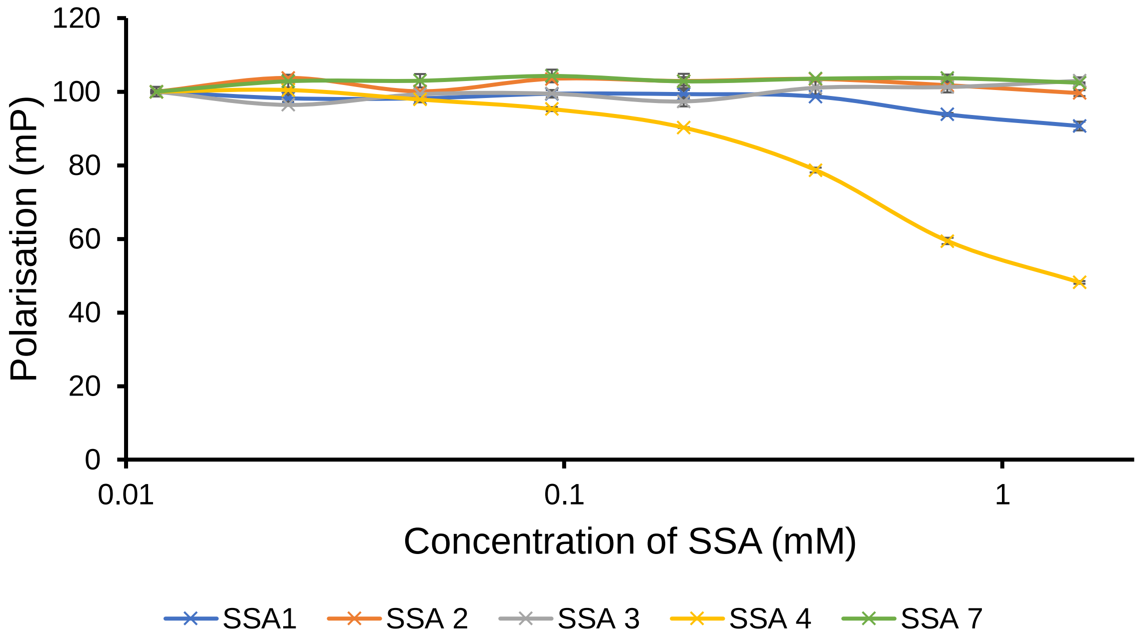

Figure S81 – Effect of SSA on FP measured in DPH-labelled *E. coli* polar vesicles at 25 °C. A target FP value of 100 mP was set to the DPH-labelled vesicles.

Section 12: Fluorescence Polarisation Adhesion Assay Data

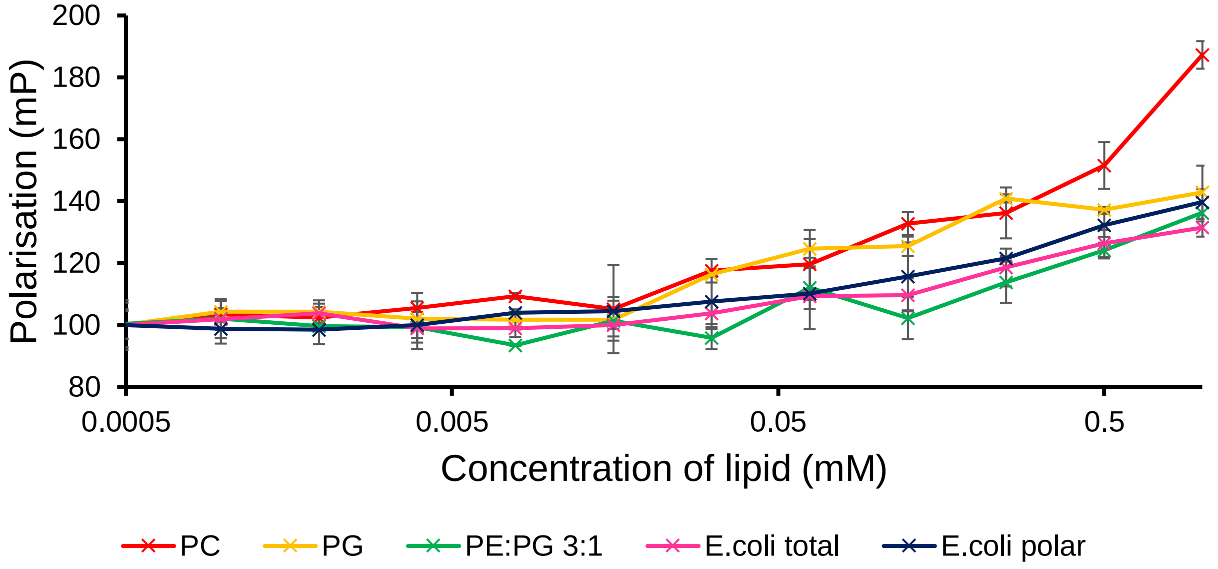

Figure S82 – Effect of fluorescent SSA **5** (0.15 mM) on FP measured in synthetic vesicles at 25 °C. A target FP value of 100 mP was set to fluorescent SSA **5** alone.

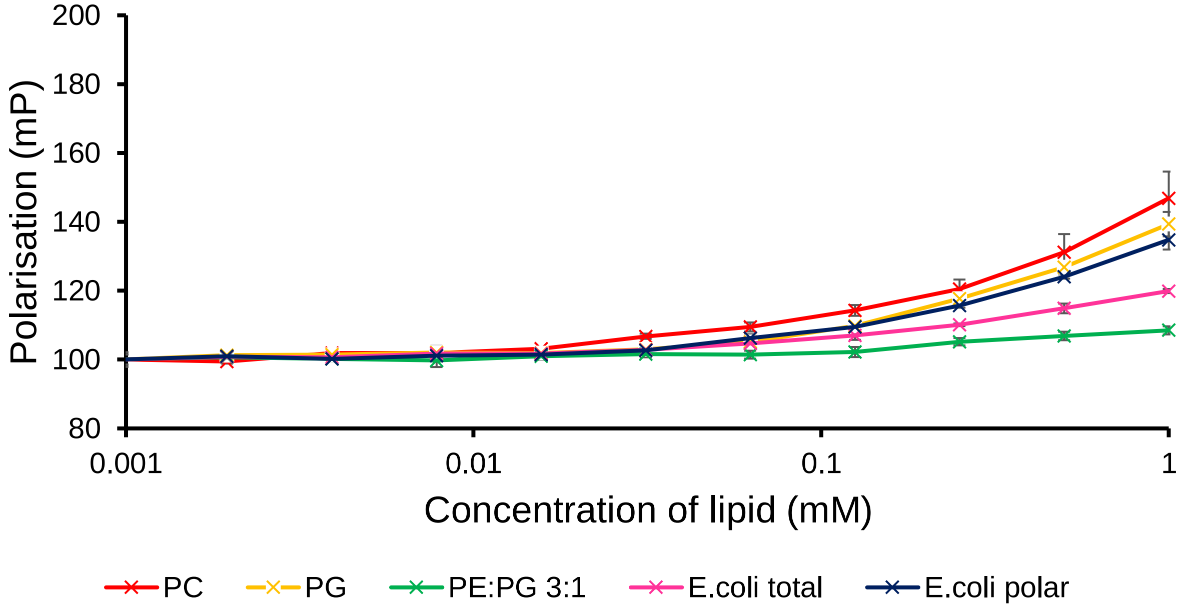

Figure S83 – Effect of fluorescent SSA **6** (0.15 mM) on FP measured in synthetic vesicles at 25 °C. A target FP value of 100 mP was set to fluorescent SSA **6** alone.

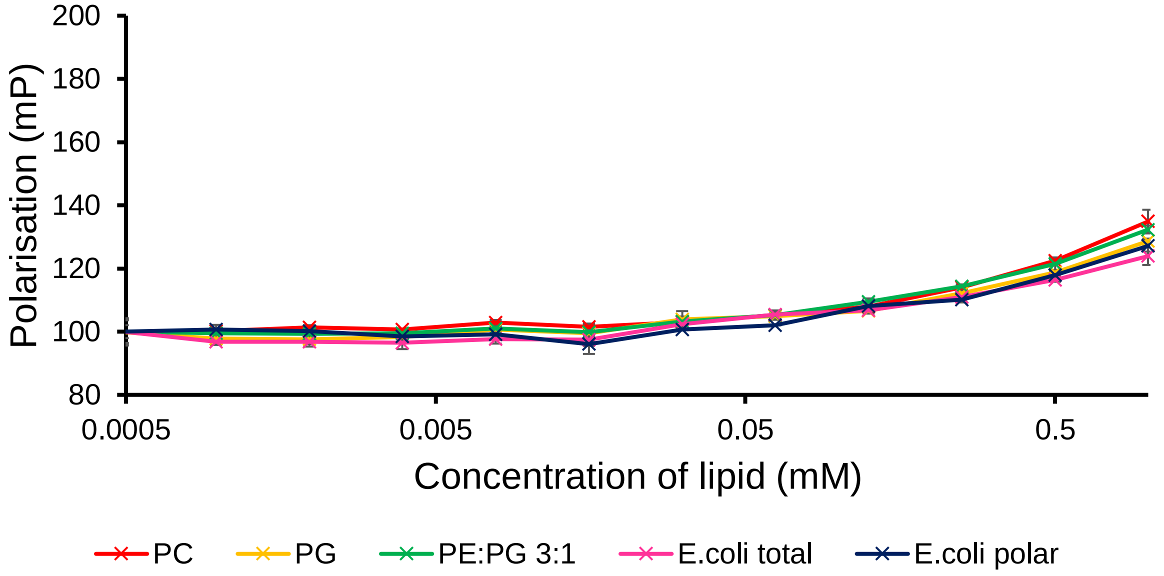

Figure S84 – Effect of fluorescent SSA **8** (0.15 mM) on FP measured in synthetic vesicles at 25 °C. A target FP value of 100 mP was set to fluorescent SSA **8** alone.

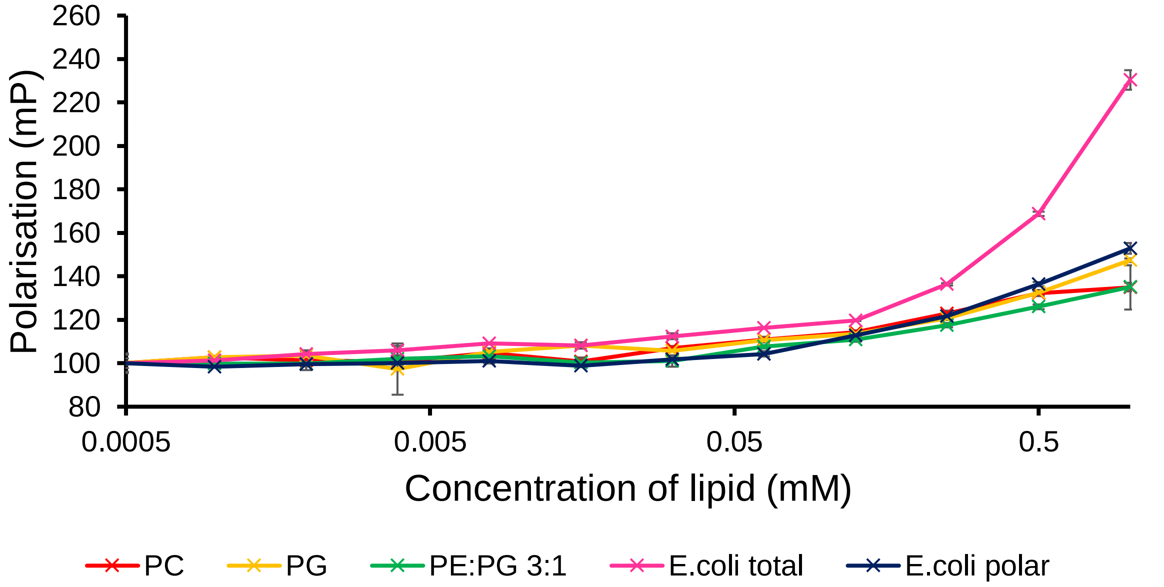

Figure S85 – Effect of fluorescent SSA **9** (0.15 mM) on FP measured in synthetic vesicles at 25 °C. A target FP value of 100 mP was set to fluorescent SSA **9** alone.

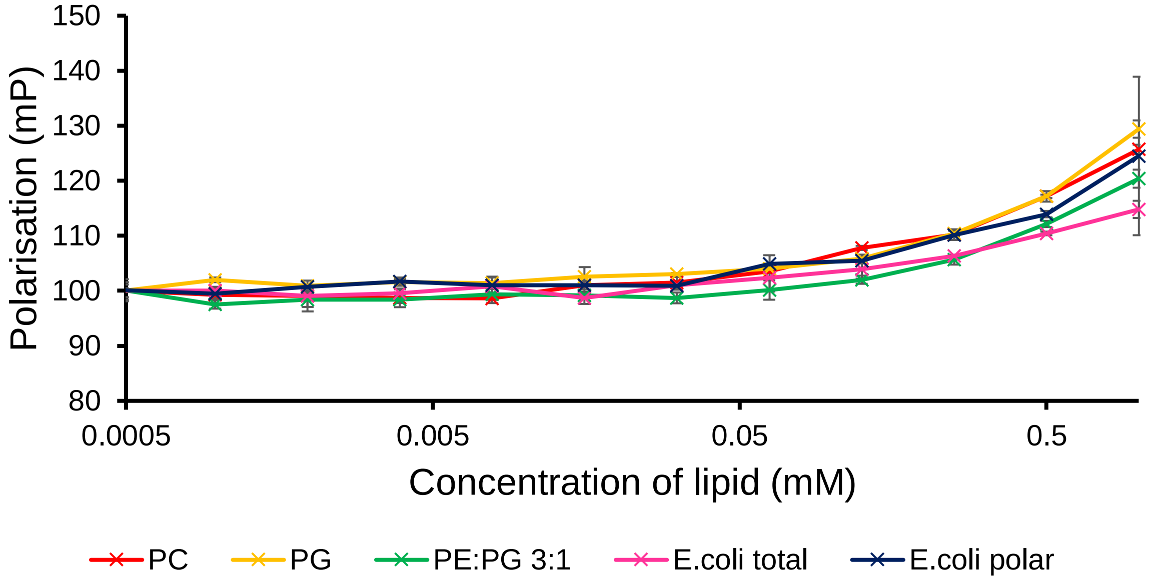

Figure S86 – Effect of fluorescent SSA **10** (0.15 mM) on FP measured in synthetic vesicles at 25 °C. A target FP value of 100 mP was set to fluorescent SSA **10** alone.

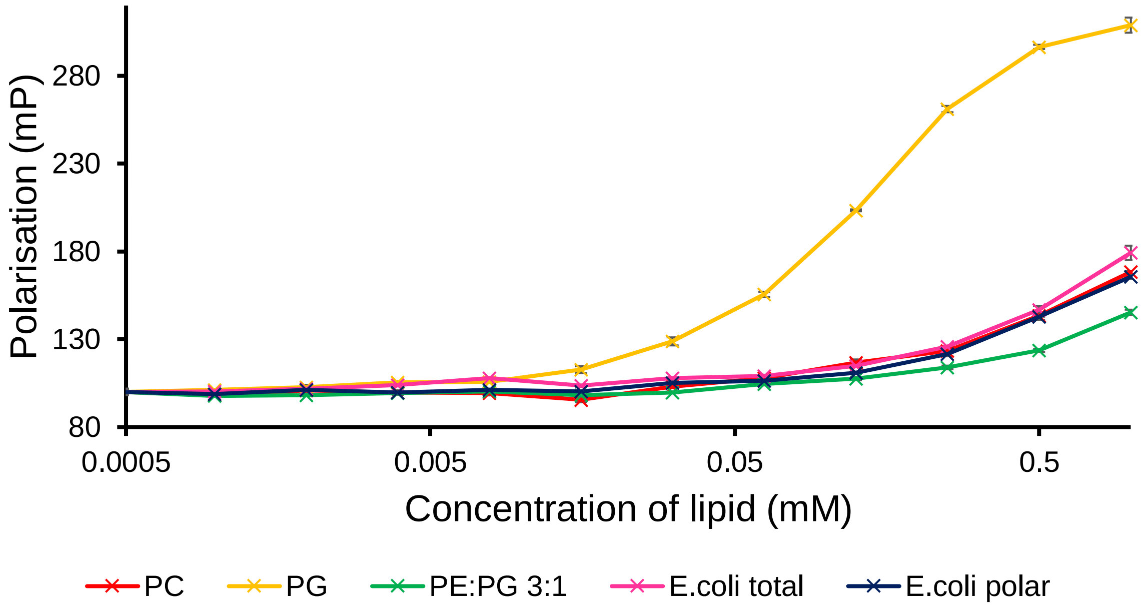

Figure S87 – Effect of fluorescent SSA **11** (0.15 mM) on FP measured in synthetic vesicles at 25 °C. A target FP value of 100 mP was set to fluorescent SSA **11** alone.

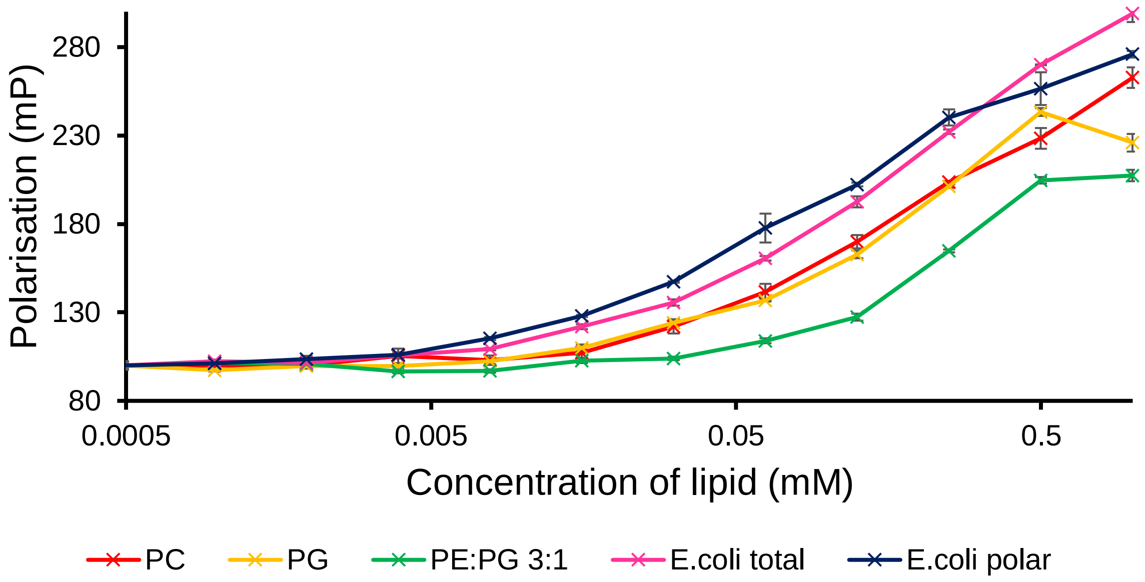

Figure S88 – Effect of fluorescent SSA **12** (0.15 mM) on FP measured in synthetic vesicles at 25 °C. A target FP value of 100 mP was set to fluorescent SSA **12** alone.

Section 13: Nanodisc Coordination Data

Nanodisc characterisation

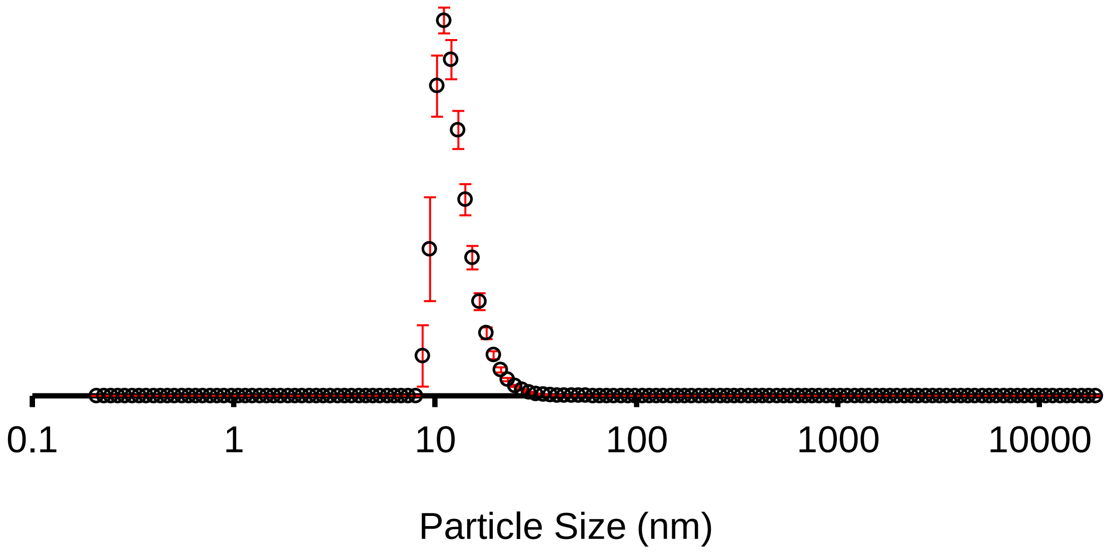

Figure S89 - Average number of weighted particle size distribution of PC nanodiscs (100 µmols) in buffer (NaCl 20 mM, NaH_2PO_4 20 mM, pH 7.4), calculated from 10 DLS runs at 298 K.

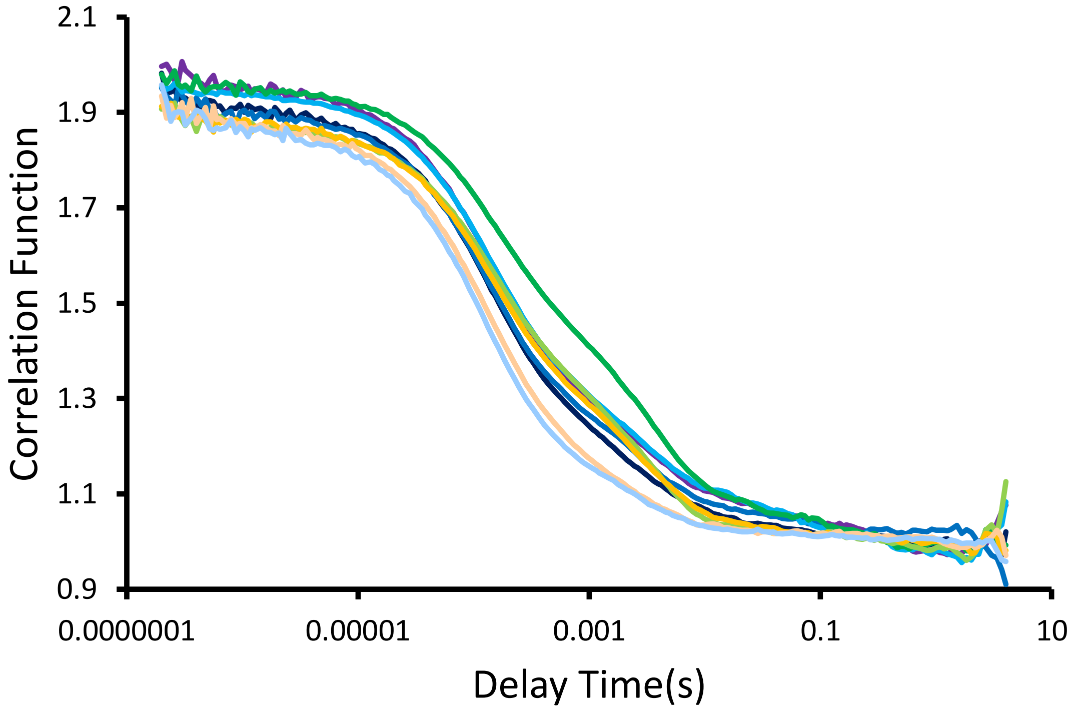

Figure S90 – Correlation function data for 10 DLS runs of PC nanodiscs (100 µmols) in buffer (NaCl 20 mM, NaH_2PO_4 20 mM, pH 7.4) at 298 K.

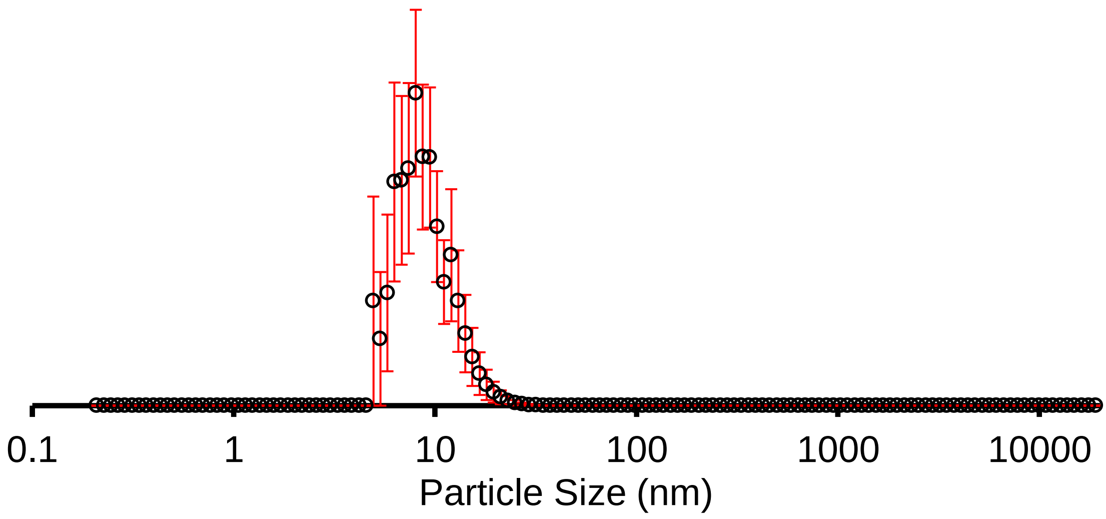

Figure S91 - Average number of weighted particle size distribution of PG nanodiscs (100 μmols) in buffer (NaCl 20 mM, NaH_2PO_4 20 mM, pH 7.4), calculated from 10 DLS runs at 298 K.

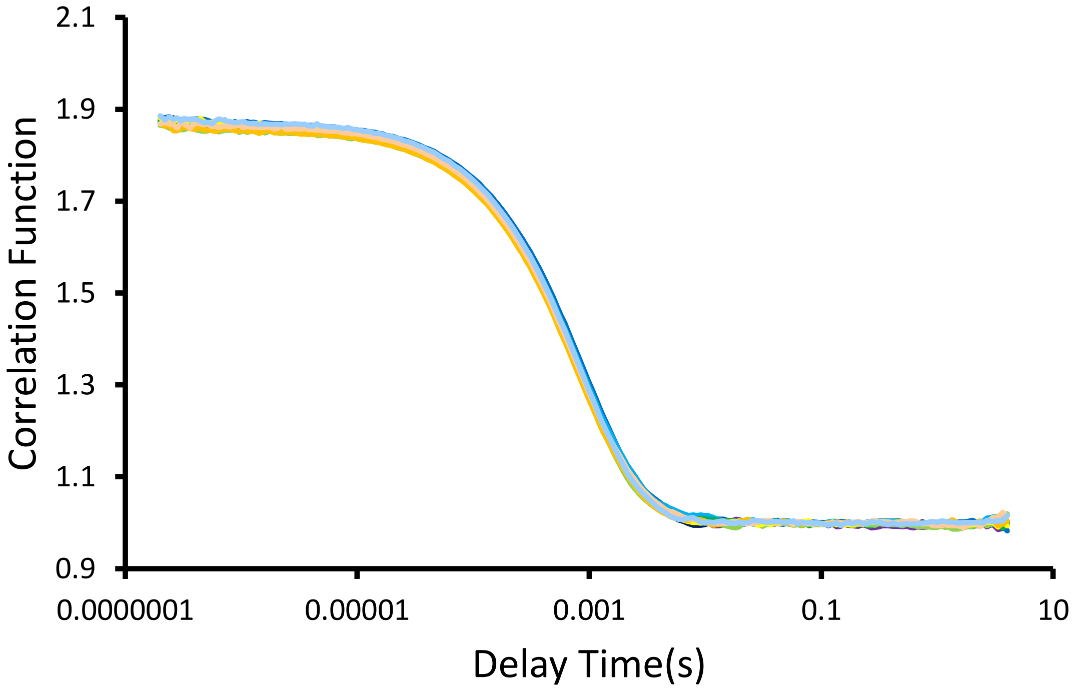

Figure S92 - Correlation function data for 10 DLS runs of PG nanodiscs (100 μmols) in buffer (NaCl 20 mM, NaH_2PO_4 20 mM, pH 7.4) at 298 K.

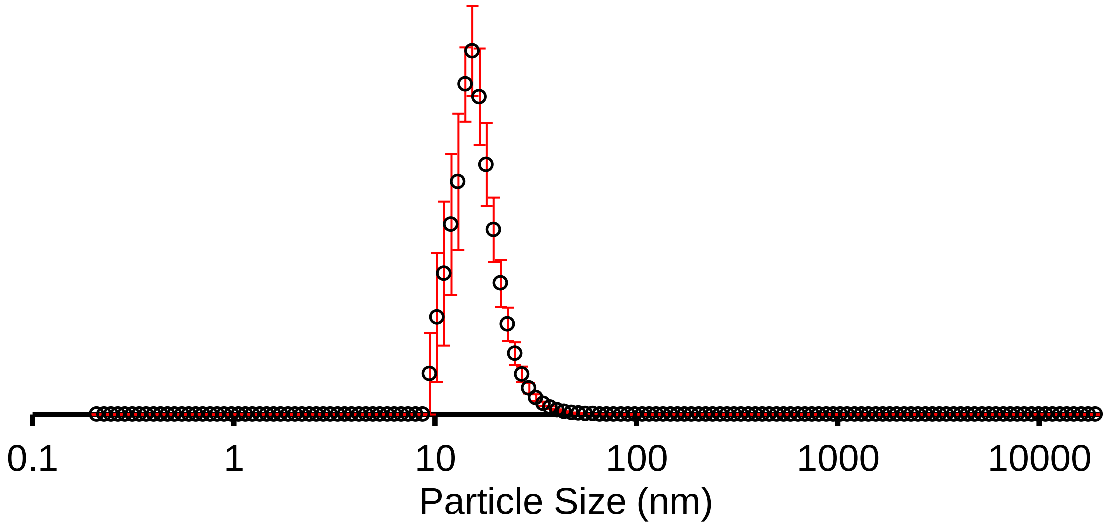

Figure S93 - Average number of weighted particle size distribution of PE:PG 3:1 mix nanodiscs (100 µmols) in buffer (NaCl 20 mM, NaH_2PO_4 20 mM, pH 7.4), calculated from 10 DLS runs at 298 K.

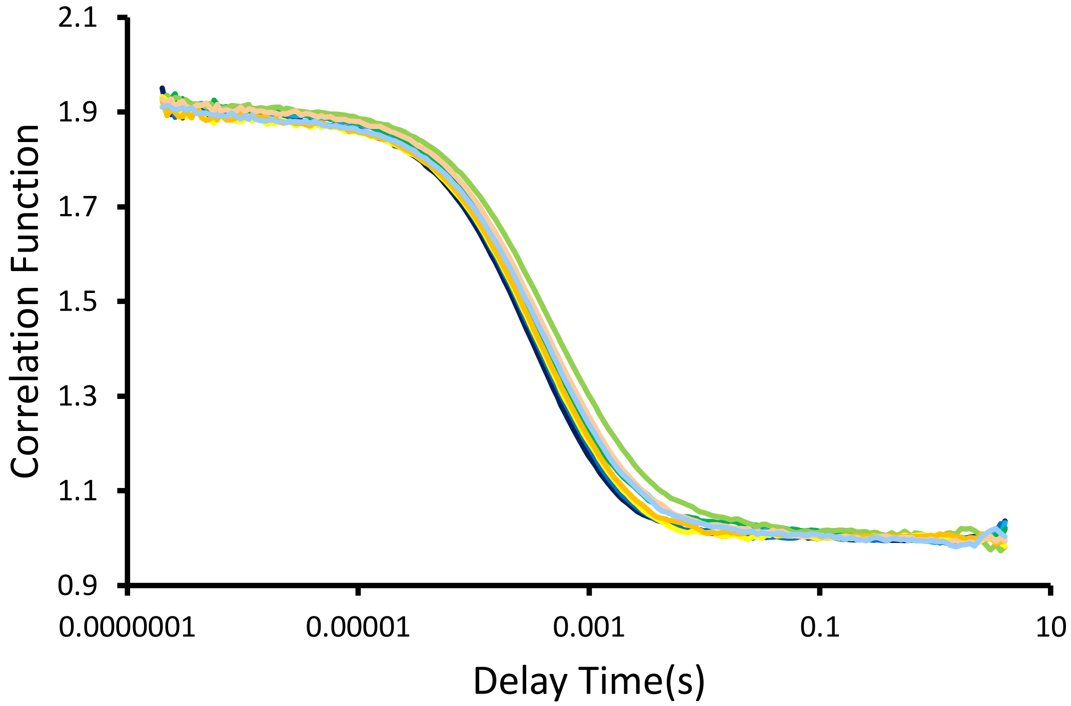

Figure S94 - Correlation function data for 10 DLS runs of of PE:PG 3:1 mix nanodiscs (100 µmols) in buffer (NaCl 20 mM, NaH_2PO_4 20 mM, pH 7.4) at 298 K.

Nanodisc titrations

All titrations carried out in buffer (NaCl 20 mM, NaH_2PO_4, pH 7.4), 5 % D_2O and 0.02 mM DSS. Concentration of SSA was kept at 100 μM, with increasing concentration of nanodisc.

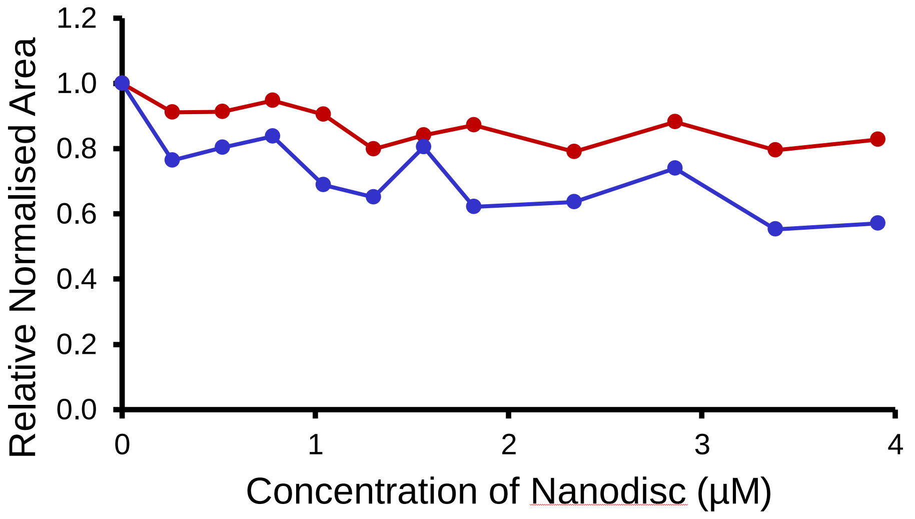

Figure S95 - The relative normalised area of a downfield aromatic resonance of SSA **5** (red) and its associated TBA cation (blue) upon titration with synthetic PC nanodiscs.

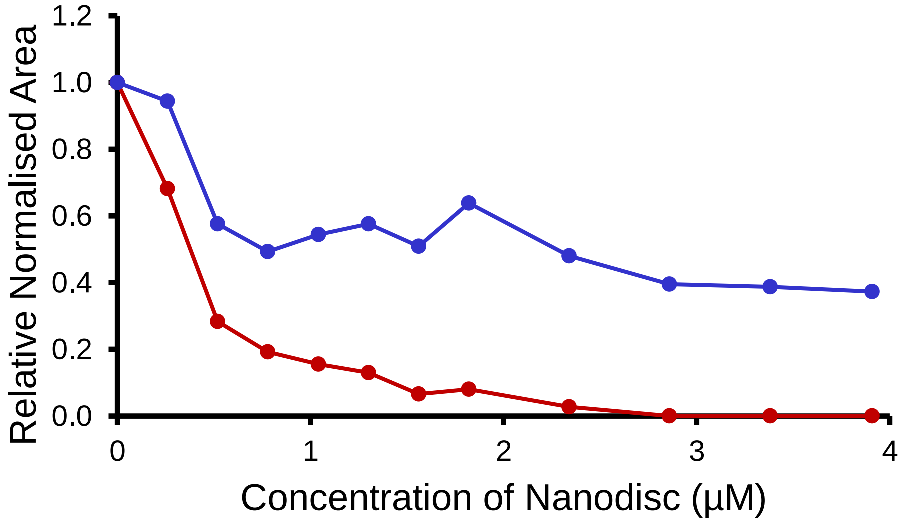

Figure S96 - The relative normalised area of a downfield aromatic resonance of SSA **6** (red) and its associated TBA cation (blue) upon titration with synthetic PC nanodiscs.

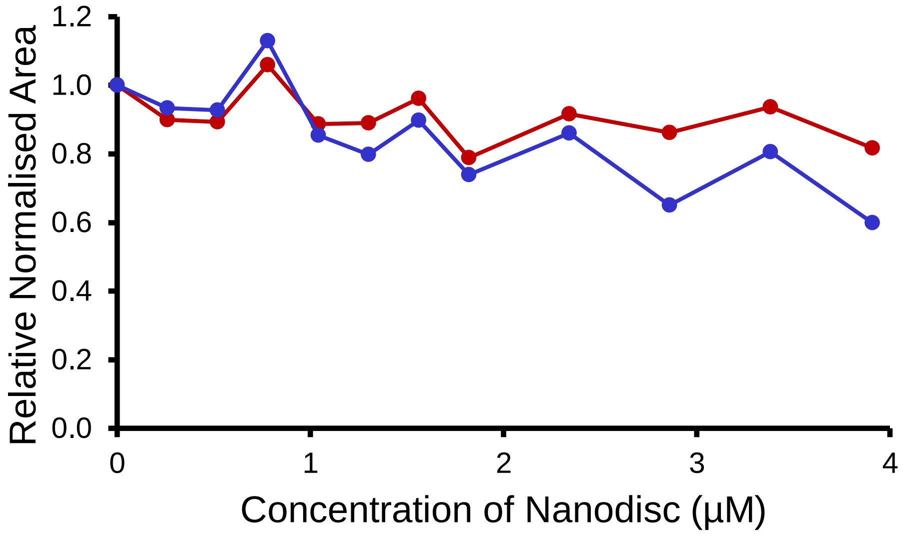

Figure S97 - The relative normalised area of a downfield aromatic resonance of SSA **7** (red) and its associated TBA cation (blue) upon titration with synthetic PC nanodiscs.

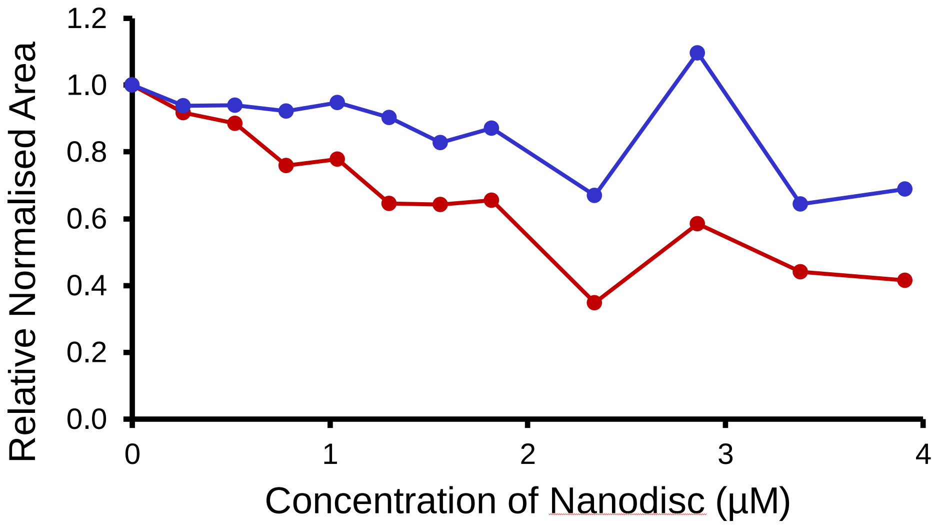

Figure S98 - The relative normalised area of a downfield aromatic resonance of SSA **8** (red) and its associated TBA cation (blue) upon titration with synthetic PC nanodiscs.

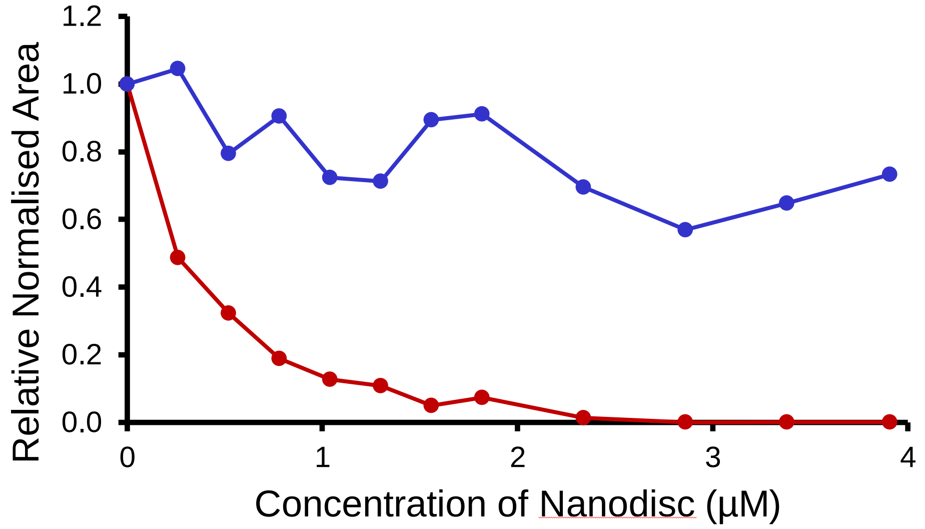

Figure S99 - The relative normalised area of a downfield aromatic resonance of SSA **9** (red) and its associated TBA cation (blue) upon titration with synthetic PC nanodiscs.

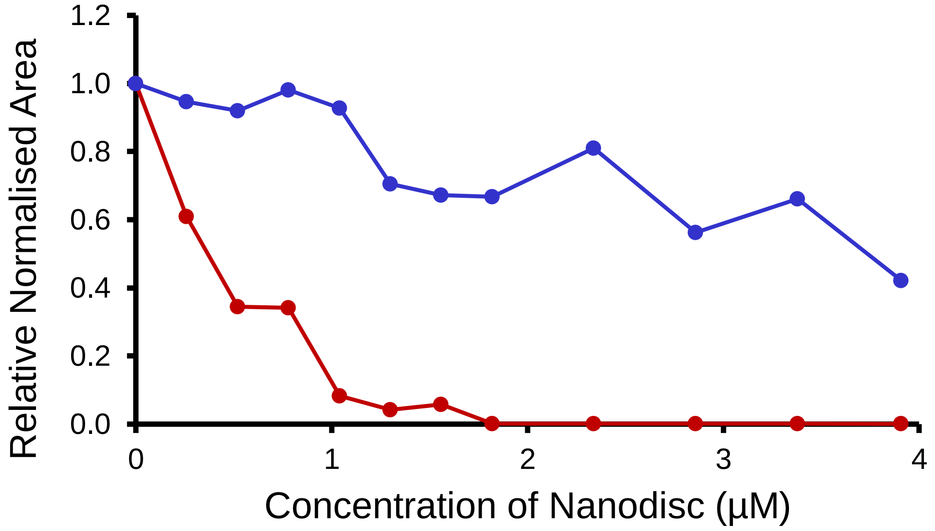

Figure S100 - The relative normalised area of a downfield aromatic resonance of SSA **10** (red) and its associated TBA cation (blue) upon titration with synthetic PC nanodiscs.

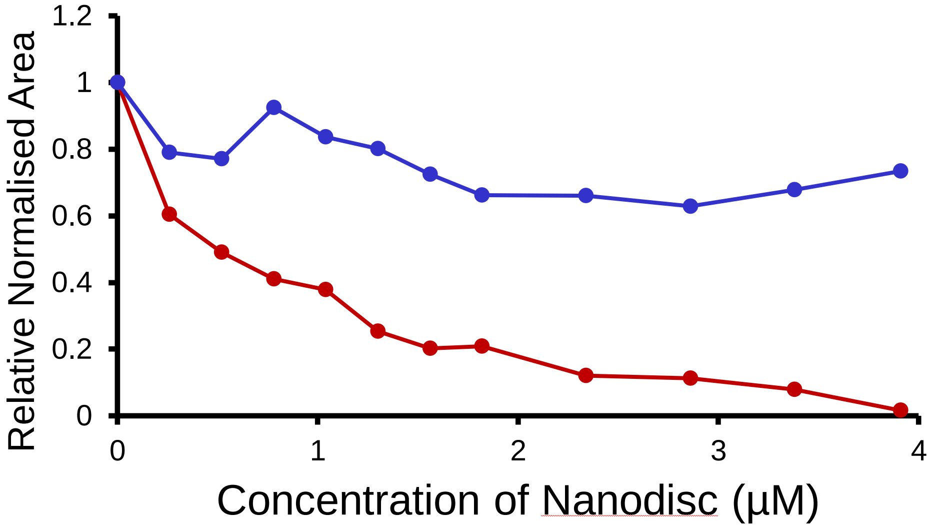

Figure S101 - The relative normalised area of a downfield aromatic resonance of SSA **11** (red) and its associated TBA cation (blue) upon titration with synthetic PC nanodiscs.

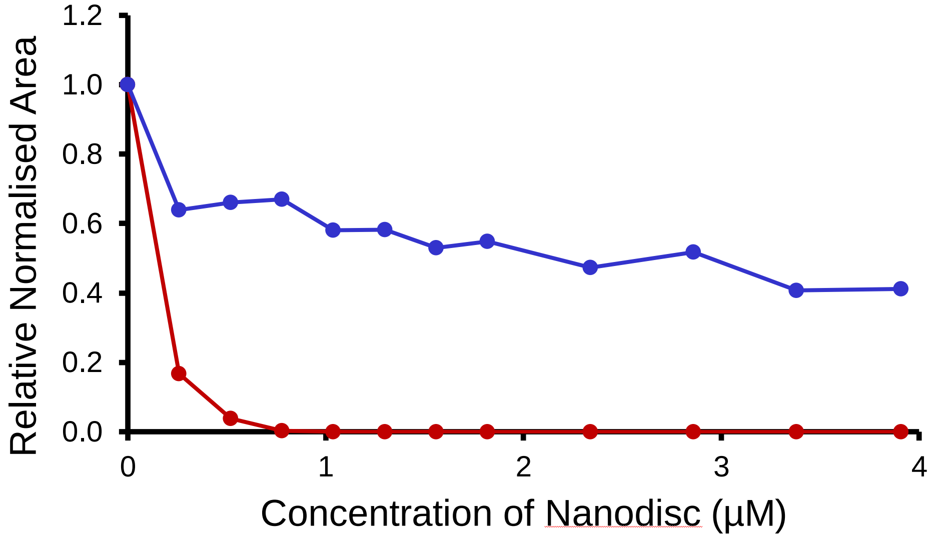

Figure S102 - The relative normalised area of a downfield aromatic resonance of SSA **12** (red) and its associated TBA cation (blue) upon titration with synthetic PC nanodiscs.

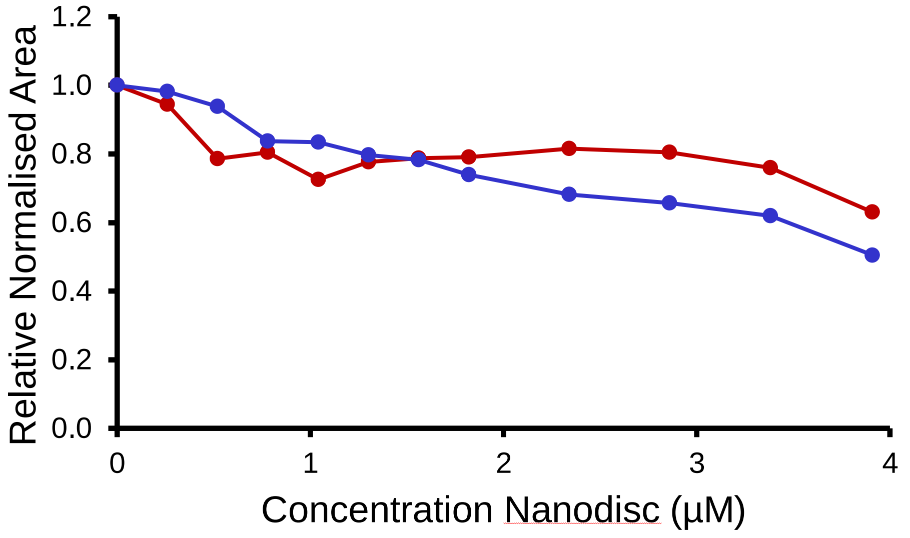

Figure S103 - The relative normalised area of a downfield aromatic resonance of SSA **5** (red) and its associated TBA cation (blue) upon titration with synthetic PG nanodiscs.

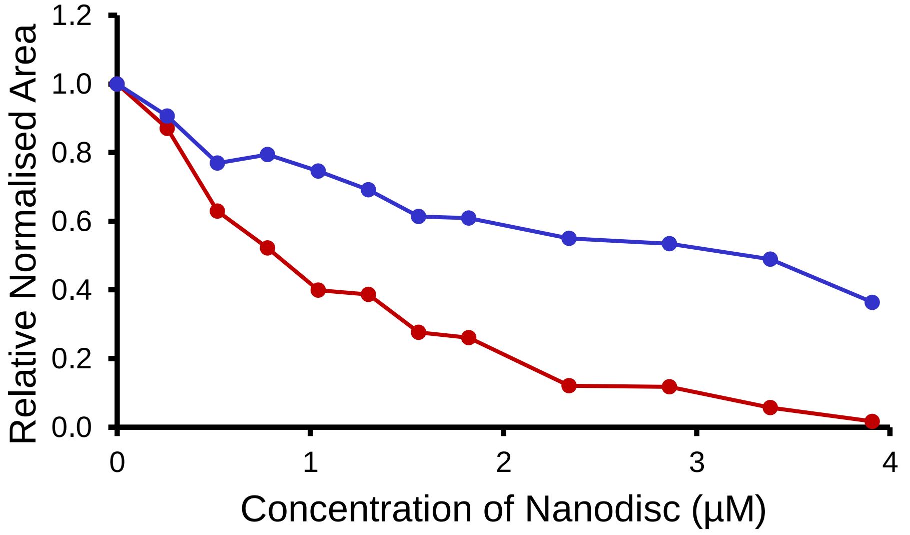

Figure S104 - The relative normalised area of a downfield aromatic resonance of SSA **6** (red) and its associated TBA cation (blue) upon titration with synthetic PG nanodiscs.

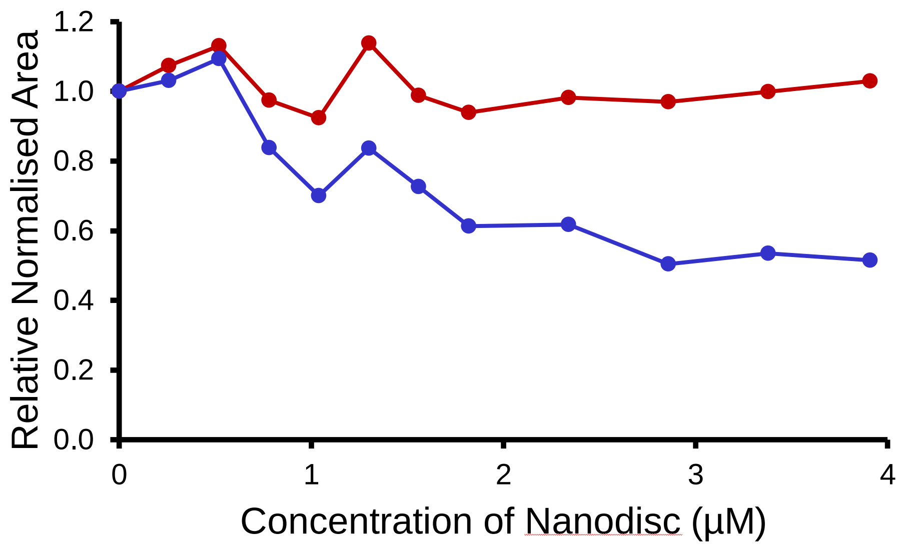

Figure S105 - The relative normalised area of a downfield aromatic resonance of SSA **7** (red) and its associated TBA cation (blue) upon titration with synthetic PG nanodiscs.

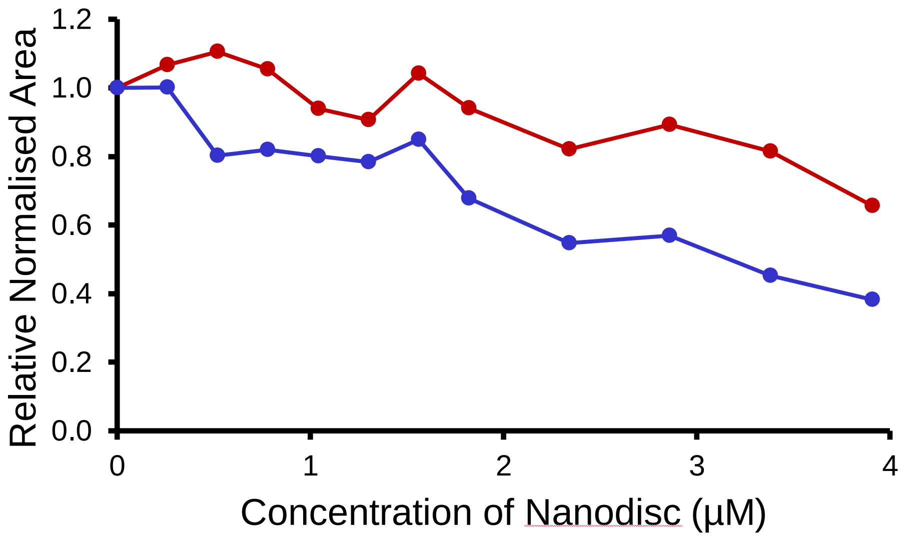

Figure S106 - The relative normalised area of a downfield aromatic resonance of SSA **8** (red) and its associated TBA cation (blue) upon titration with synthetic PG nanodiscs.

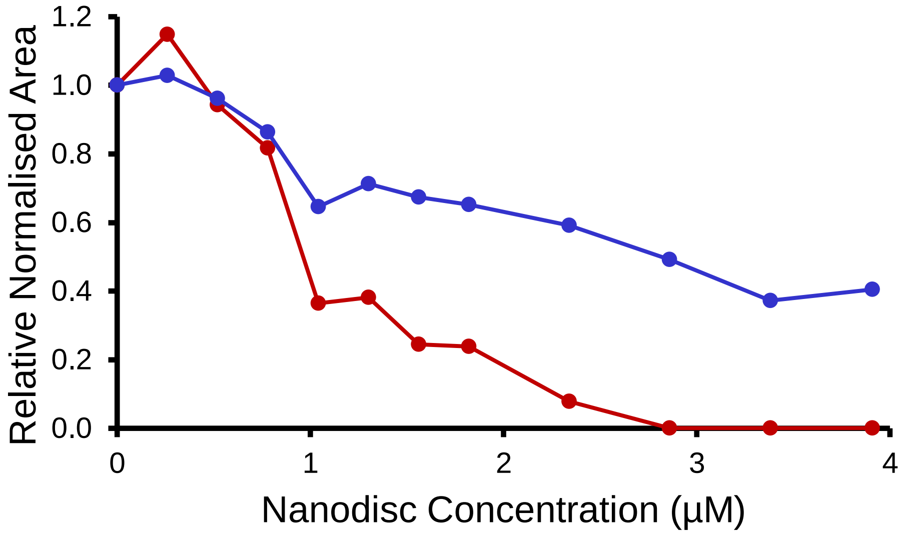

Figure S107 - The relative normalised area of a downfield aromatic resonance of SSA **9** (red) and its associated TBA cation (blue) upon titration with synthetic PG nanodiscs.

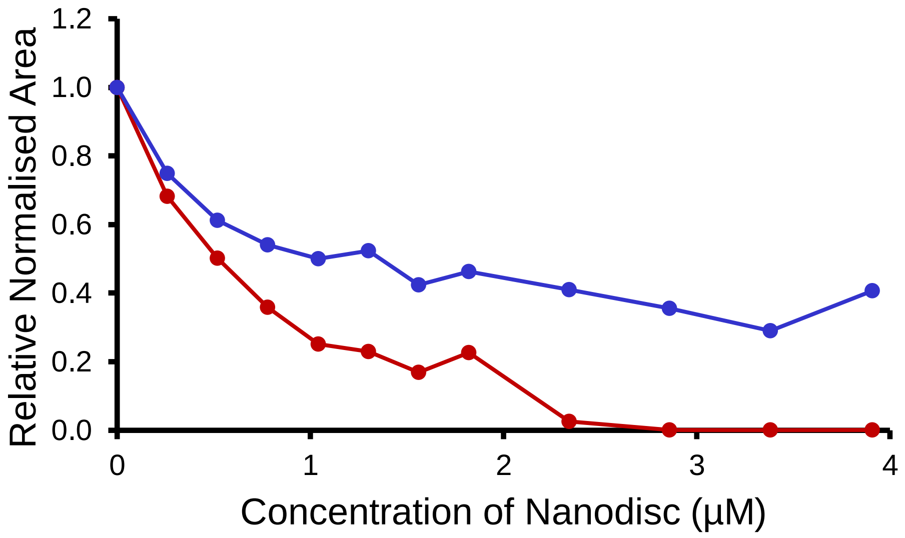

Figure S108 - The relative normalised area of a downfield aromatic resonance of SSA **10** (red) and its associated TBA cation (blue) upon titration with synthetic PG nanodiscs.

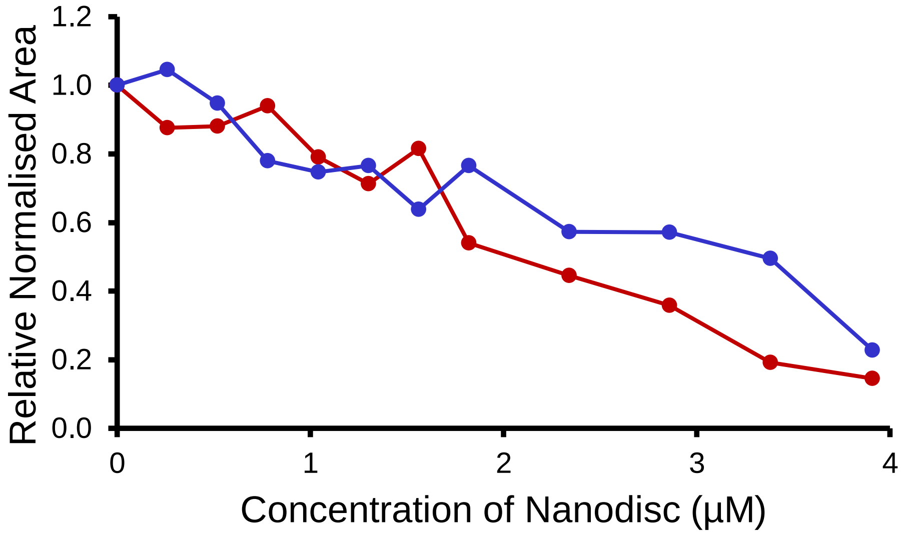

Figure S109 - The relative normalised area of a downfield aromatic resonance of SSA **11** (red) and its associated TBA cation (blue) upon titration with synthetic PG nanodiscs.

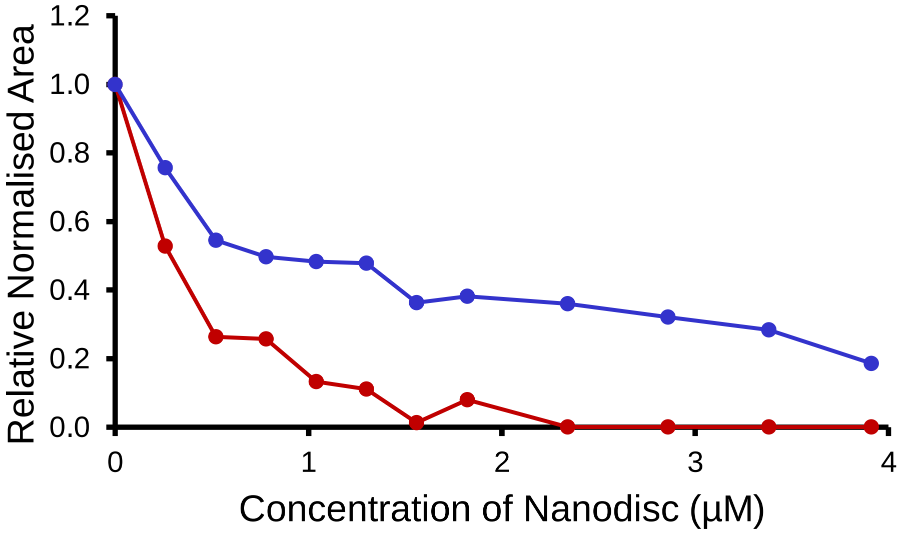

Figure S110 - The relative normalised area of a downfield aromatic resonance of SSA **12** (red) and its associated TBA cation (blue) upon titration with synthetic PG nanodiscs.

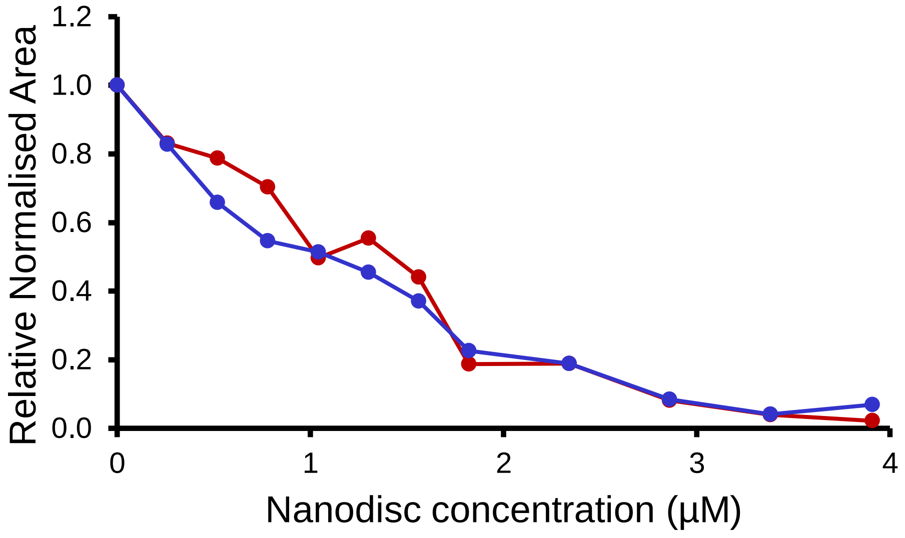

Figure S111 - The relative normalised area of a downfield aromatic resonance of SSA **5** (red) and its associated TBA cation (blue) upon titration with synthetic PE:PG 3:1 mix nanodiscs.

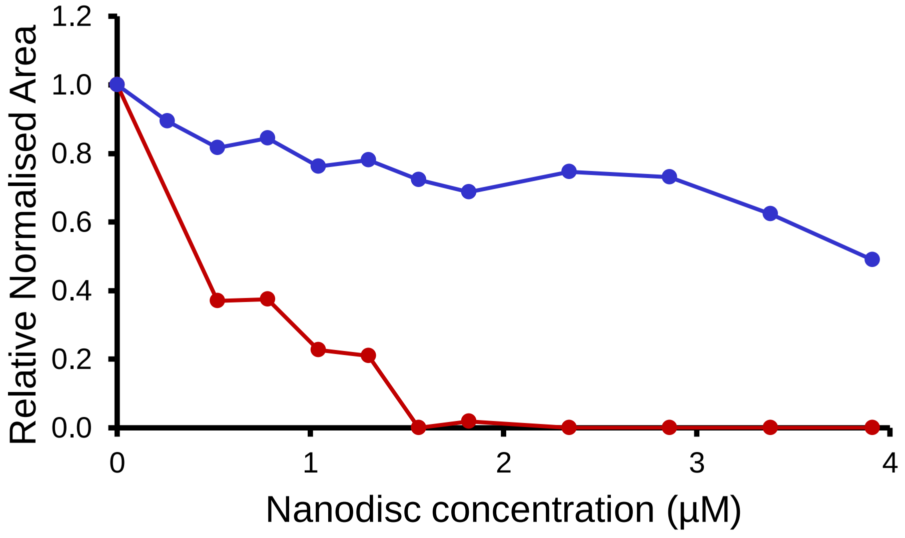

Figure S112 - The relative normalised area of a downfield aromatic resonance of SSA **6** (red) and its associated TBA cation (blue) upon titration with synthetic PE:PG 3:1 mix nanodiscs.

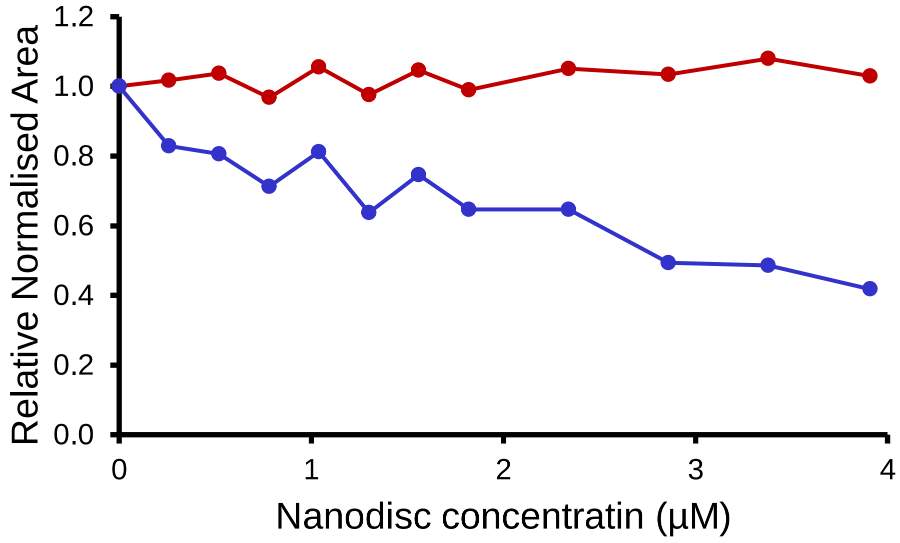

Figure S113 - The relative normalised area of a downfield aromatic resonance of SSA **7** (red) and its associated TBA cation (blue) upon titration with synthetic PE:PG 3:1 mix nanodiscs.

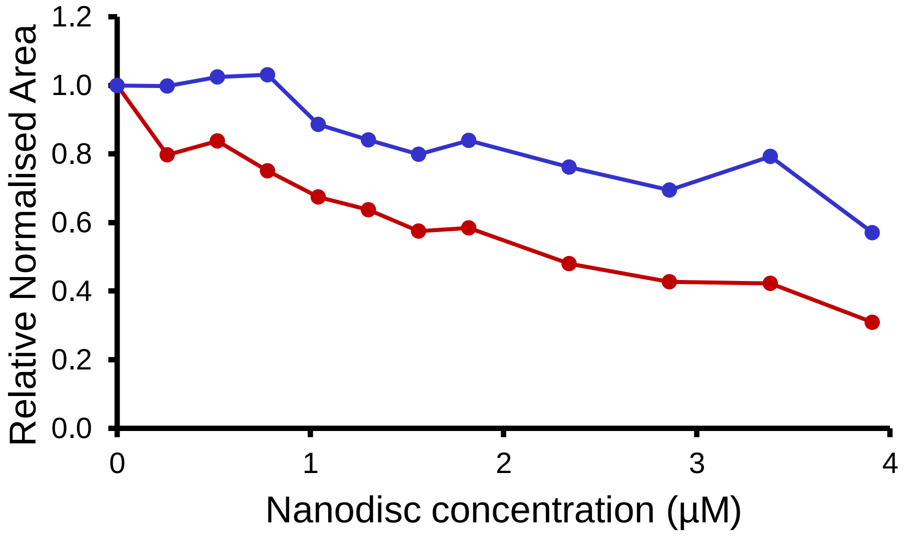

Figure S114 - The relative normalised area of a downfield aromatic resonance of SSA **8** (red) and its associated TBA cation (blue) upon titration with synthetic PE:PG 3:1 mix nanodiscs.

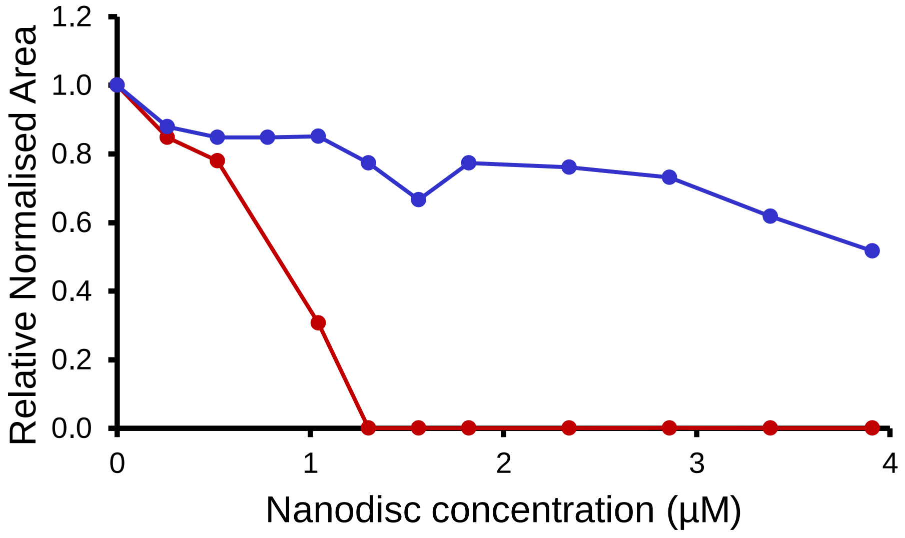

Figure S115 - The relative normalised area of a downfield aromatic resonance of SSA **9** (red) and its associated TBA cation (blue) upon titration with synthetic PE:PG 3:1 mix nanodiscs.

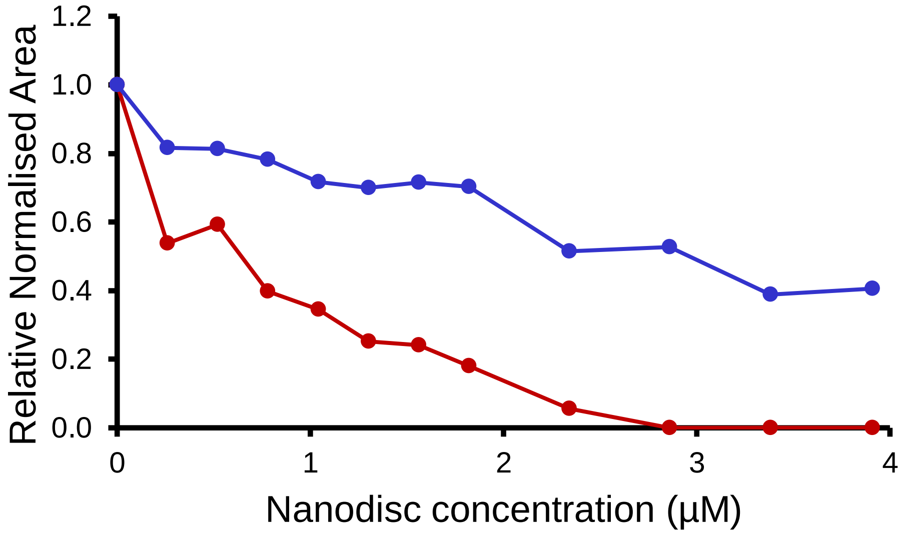

Figure S116 - The relative normalised area of a downfield aromatic resonance of SSA **10** (red) and its associated TBA cation (blue) upon titration with synthetic PE:PG 3:1 mix nanodiscs.

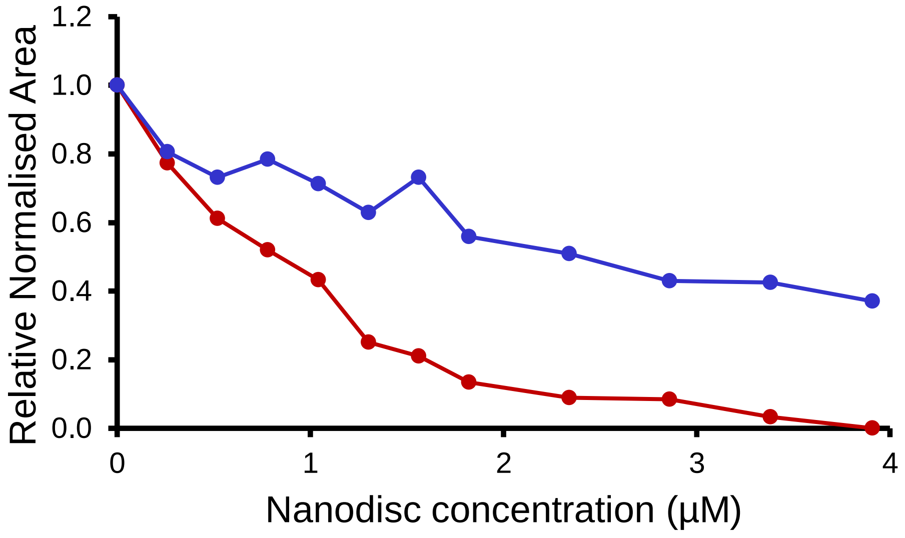

Figure S117 - The relative normalised area of a downfield aromatic resonance of SSA **11** (red) and its associated TBA cation (blue) upon titration with synthetic PE:PG 3:1 mix nanodiscs.

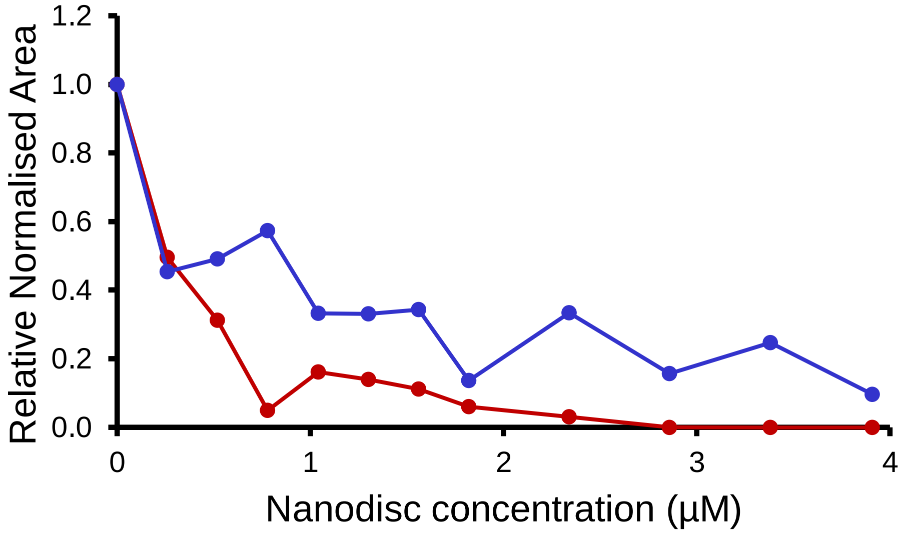

Figure S118 - The relative normalised area of a downfield aromatic resonance of SSA **12** (red) and its associated TBA cation (blue) upon titration with synthetic PE:PG 3:1 mix nanodiscs.

Section 14: Determination of Nanodisc Coordination EC_{50} Values

To enable further comparison of these data, they were fitted to both Michaelis-Menten and Hill Plot models to enable calculation of the concentration of nanodisc required to coordinate 50 % of either the SSA anionic or cationic component independently.

Comparative R^2 analysis of the fits obtained from the SSA anion and cation titration data to either Michaelis-Menten or Hill Plot kinetics using Origin 2018 software, with V_{max} fixed to 100 % of the SSA component to become NMR silent, supported the use of the Hill plot model to fit these data, which are summarised in Table S4.

Table S4 - EC_{50} (µM) and hill coefficient (*n*) values obtained from the fitting of nanodisc titration data to Hill Plot kinetics using Origin 2018 software, with V_{max} fixed as 100 % of the SSA component to become NMR silent, and thus we assume bound to the phospholipid nanodisc. $R^2 > 0.85$ for all data fits reported. A = anionic SSA component. C = cationic SSA component. Nanodisc phospholipid compositions are summarised in Table 2 – main manuscript.

SSA	PC		PG		PE:PG mix	
	EC_{50}	*n*	EC_{50}	*n*	EC_{50}	*n*
**5** A	*a*	*a*	*a*	*a*	1.14 (±0.08)	2.05 (±0.29)
**5** C	*a*	*a*	*b*	1.11 (±0.09)	0.93 (±<0.01)	1.60 (±0.01)
**6** A	0.36 (±0.02)	1.84 (±0.12)	0.82 (±0.04)	1.60 (±0.11)	0.46 (±0.07)	1.85 (±0.40)
**6** C	*a*	*a*	2.86 (±0.18)	0.93 (±0.08)	*b*	0.70 (±0.14)
**7** A	*a*	*a*	*a*	*a*	*a*	*a*
**7** C	*a*	*a*	3.42 (±0.02)	1.20 (±0.01)	3.44 (±0.51)	0.81 (±0.14)
**8** A	2.66 (±0.31)	1.06 (±0.19)	*a*	*a*	2.20 (±0.13)	0.98 (±0.09)
**8** C	*a*	*a*	3.11 (±0.28)	1.35 (±0.21)	*a*	*a*
**9** A	0.27 (±0.02)	1.43 (±0.10)	1.07 (±0.06)	3.29 (±0.57)	0.73 (±0.05)	3.76 (±0.62)
**9** C	*b*	0.71 (±<0.01)	2.69 (±0.20)	1.34 (±0.18)	*a*	*a*
**10** A	0.36 (±0.03)	1.80 (±0.23)	0.50 (±0.04)	1.47 (±0.16)	0.50 (±0.08)	1.25 (±0.21)
**10** C	*b*	1.27 (±<0.01)	1.16 (±0.06)	0.67 (±0.05)	2.92 (±0.30)	0.95 (±0.14)
**11** A	0.49 (±0.04)	1.10 (±0.10)	2.10 (±0.11)	2.41 (±0.33)	0.72 (±0.04)	1.69 (±0.15)
**11** C	*a*	*a*	2.82 (±0.28)	1.45 (±0.26)	2.51 (±0.26)	0.87 (±0.13)
**12** A	0.14 (±0.01)	2.65 (±0.15)	0.29 (±0.02)	1.51 (±0.15)	0.26 (±0.03)	1.44 (±0.20)
**12** C	2.24 (±0.31)	0.42 (±0.07)	0.90 (±0.07)	0.75 (±0.07)	0.37 (±<0.01)	0.62 (±<0.01)

a - $R^2 < 0.85$, the results of data the fitting are not reported. *b* = Less than 50 % SSA becomes NMR silent at experimental endpoint (4.00 µM).

Mechaelis Menten fits

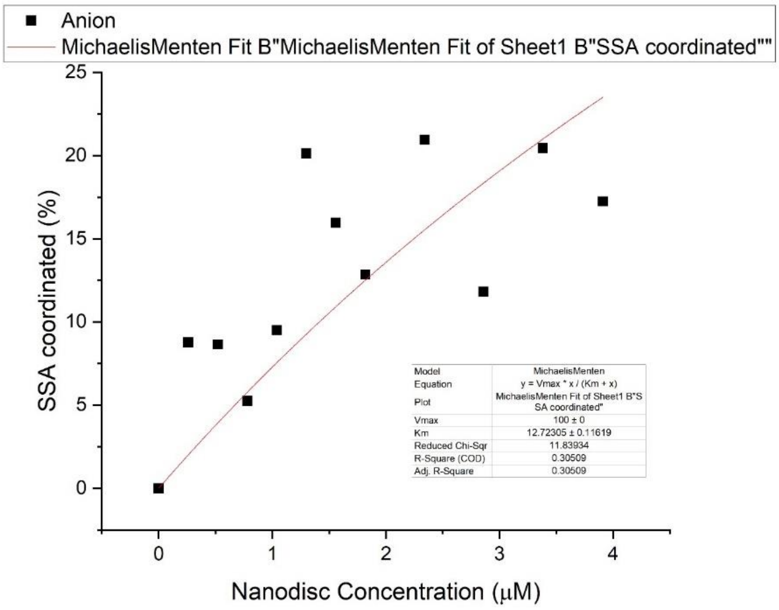

Figure S119 - Graph showing the percentage of SSA **5** anion coordinated to PC nanodiscs, with respect to increasing nanodisc concentration. Data was then fitted to the Michaelis Menten model with V_{max} fixed at 100 %.

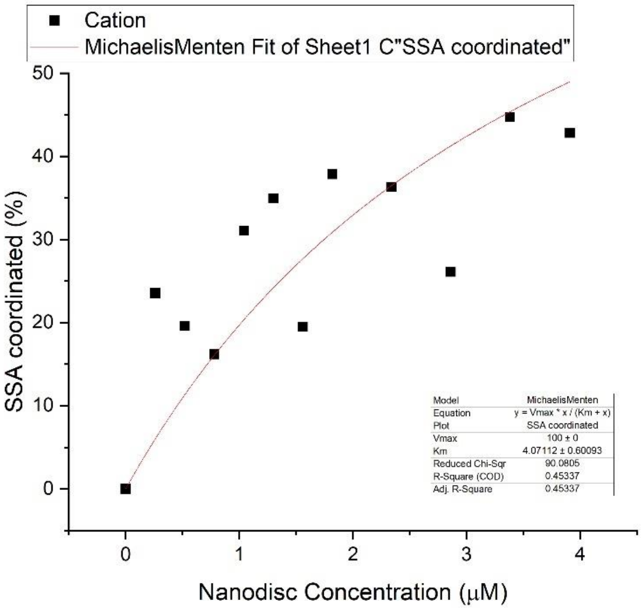

Figure S120 - Graph showing the percentage of SSA **5** cation coordinated to PC nanodiscs, with respect to increasing nanodisc concentration. Data was then fitted to the Michaelis Menten model with V_{max} fixed at 100 %.

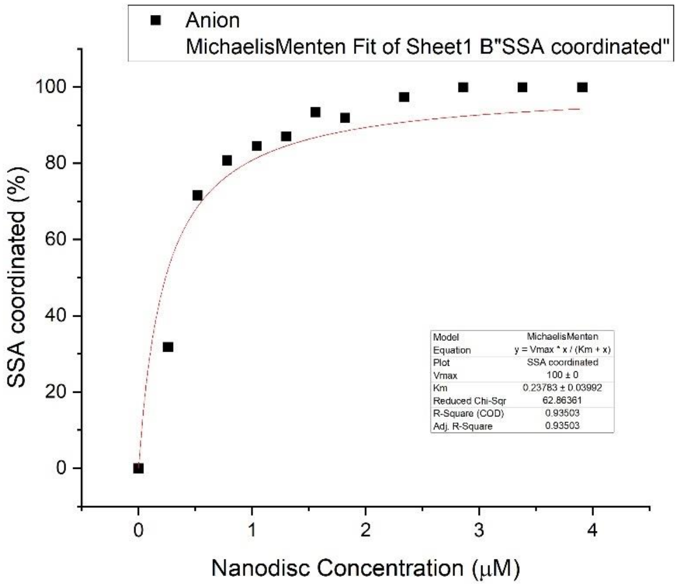

Figure S121 - Graph showing the percentage of SSA **6** anion coordinated to PC nanodiscs, with respect to increasing nanodisc concentration. Data was then fitted to the Michaelis Menten model with V_{max} fixed at 100 %.

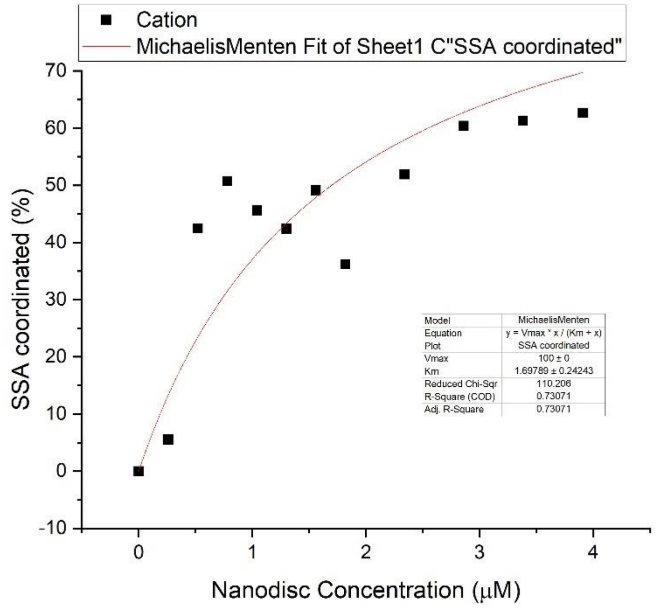

Figure S122 - Graph showing the percentage of SSA **6** cation coordinated to PC nanodiscs, with respect to increasing nanodisc concentration. Data was then fitted to the Michaelis Menten model with V_{max} fixed at 100 %.

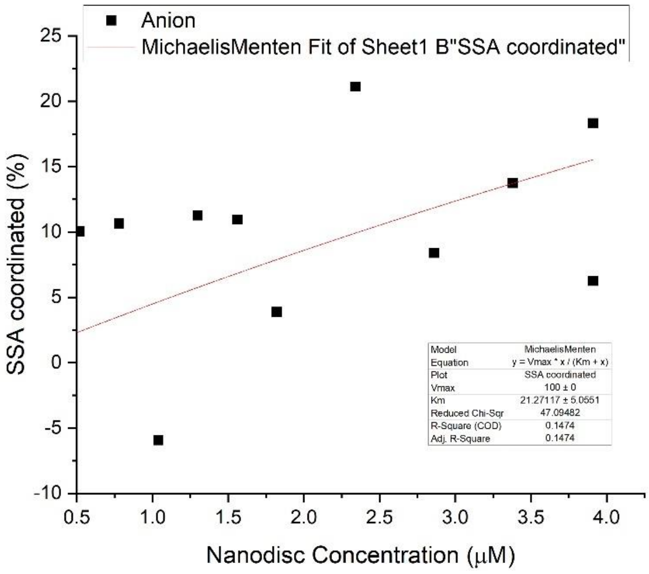

Figure S123 - Graph showing the percentage of SSA **7** anion coordinated to PC nanodiscs, with respect to increasing nanodisc concentration. Data was then fitted to the Michaelis Menten model with V_{max} fixed at 100 %.

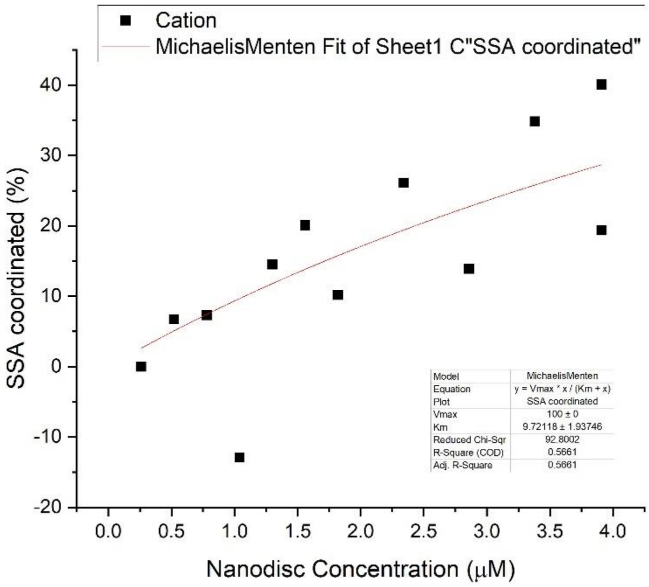

Figure S124 - Graph showing the percentage of SSA **7** cation coordinated to PC nanodiscs, with respect to increasing nanodisc concentration. Data was then fitted to the Michaelis Menten model with V_{max} fixed at 100 %.

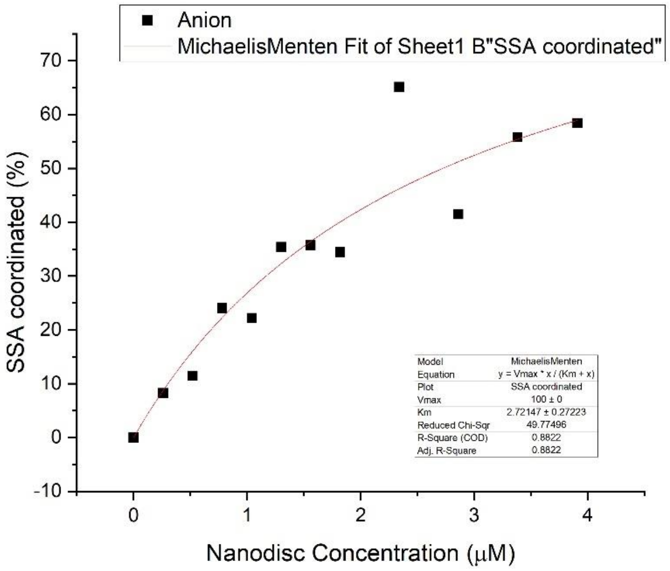

Figure S125 - Graph showing the percentage of SSA **8** anion coordinated to PC nanodiscs, with respect to increasing nanodisc concentration. Data was then fitted to the Michaelis Menten model with V_{max} fixed at 100 %.

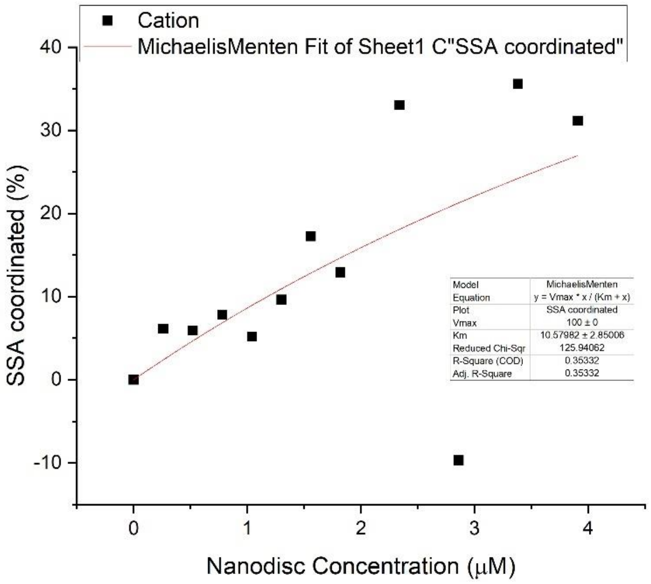

Figure S126 - Graph showing the percentage of SSA **8** cation coordinated to PC nanodiscs, with respect to increasing nanodisc concentration. Data was then fitted to the Michaelis Menten model with V_{max} fixed at 100 %.

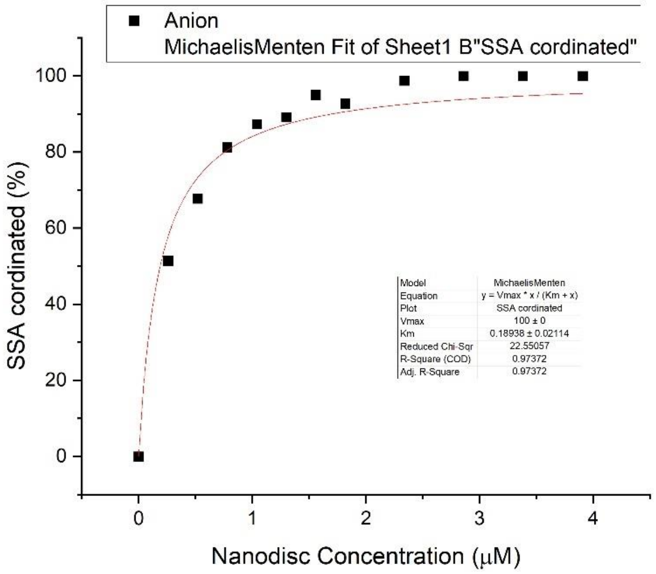

Figure S127 - Graph showing the percentage of SSA **9** anion coordinated to PC nanodiscs, with respect to increasing nanodisc concentration. Data was then fitted to the Michaelis Menten model with V_{max} fixed at 100 %.

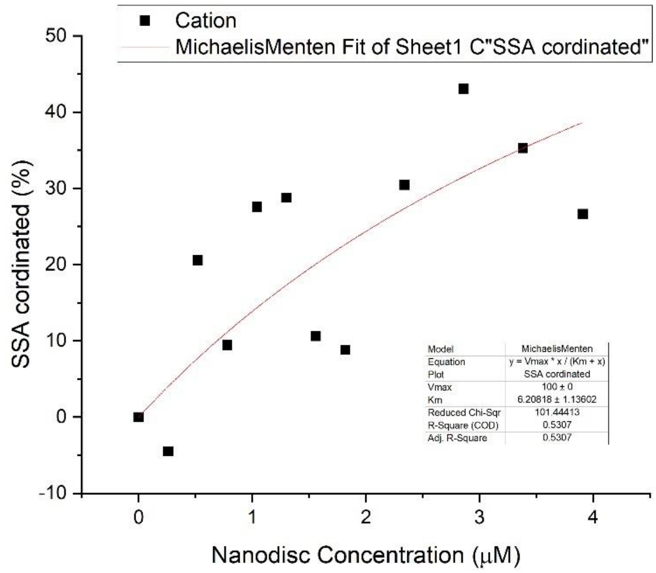

Figure S128 - Graph showing the percentage of SSA **8** cation coordinated to PC nanodiscs, with respect to increasing nanodisc concentration. Data was then fitted to the Michaelis Menten model with V_{max} fixed at 100 %.

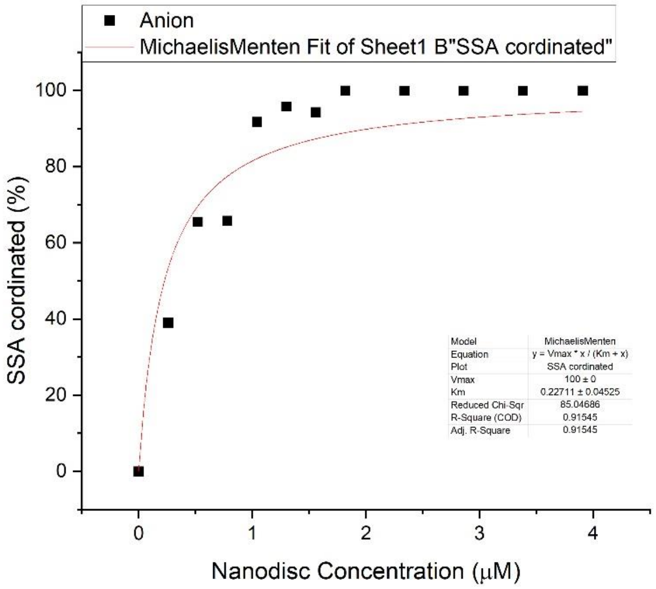

Figure S129 - Graph showing the percentage of SSA **10** anion coordinated to PC nanodiscs, with respect to increasing nanodisc concentration. Data was then fitted to the Michaelis Menten model with V_{max} fixed at 100 %.

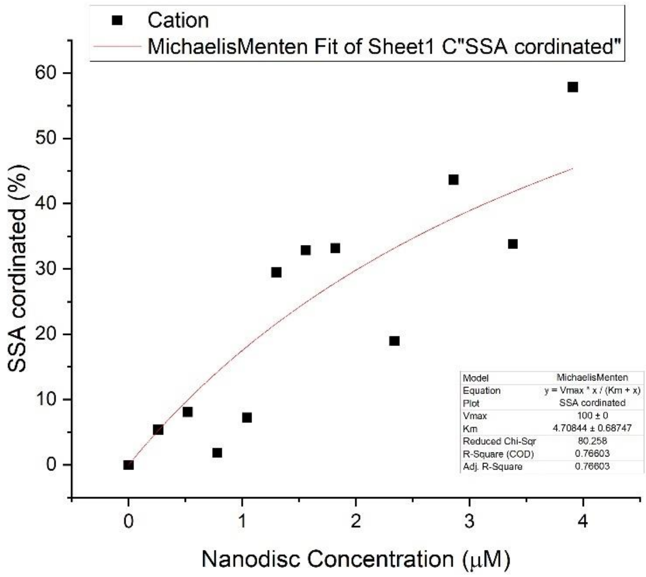

Figure S130 - Graph showing the percentage of SSA **10** cation coordinated to PC nanodiscs, with respect to increasing nanodisc concentration. Data was then fitted to the Michaelis Menten model with V_{max} fixed at 100 %.

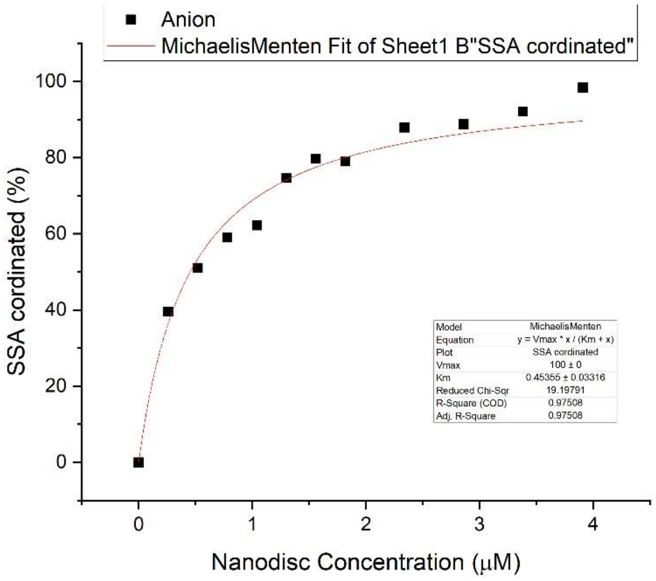

Figure S131 - Graph showing the percentage of SSA **11** anion coordinated to PC nanodiscs, with respect to increasing nanodisc concentration. Data was then fitted to the Michaelis Menten model with V_{max} fixed at 100 %.

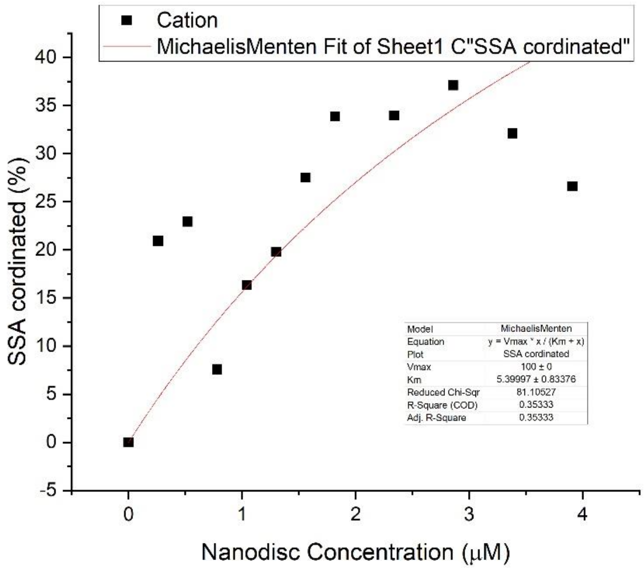

Figure S132 - Graph showing the percentage of SSA **11** cation coordinated to PC nanodiscs, with respect to increasing nanodisc concentration. Data was then fitted to the Michaelis Menten model with V_{max} fixed at 100 %.

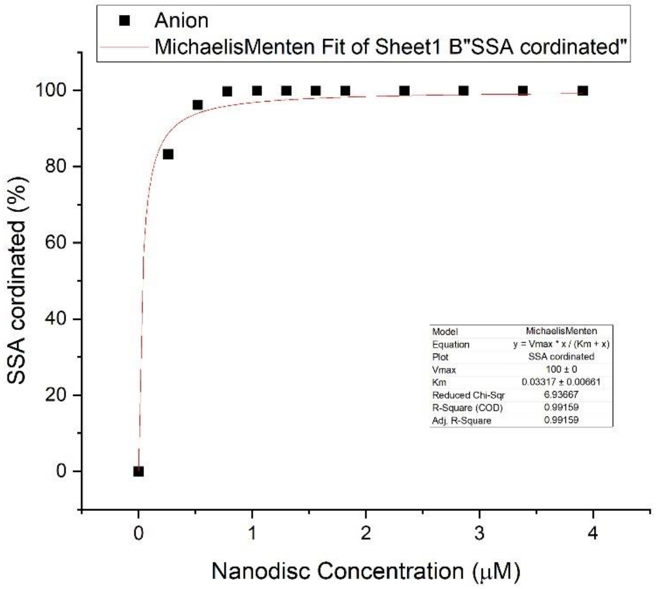

Figure S133 - Graph showing the percentage of SSA **12** anion coordinated to PC nanodiscs, with respect to increasing nanodisc concentration. Data was then fitted to the Michaelis Menten model with V_{max} fixed at 100 %.

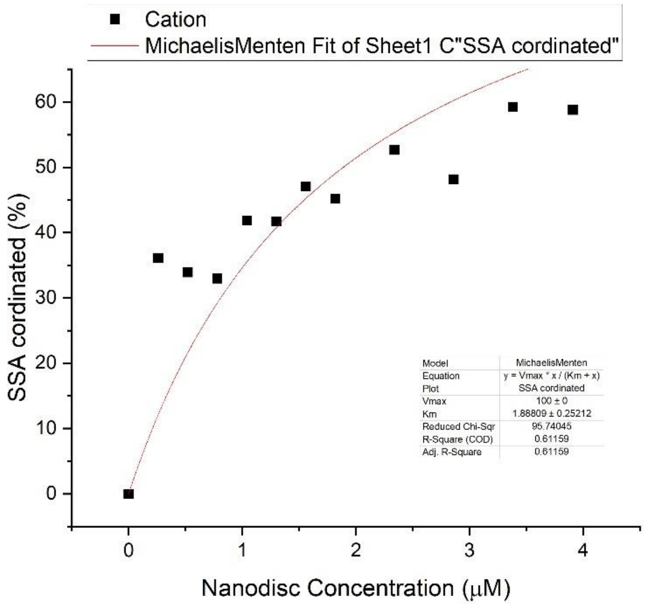

Figure S134 - Graph showing the percentage of SSA **12** cation coordinated to PC nanodiscs, with respect to increasing nanodisc concentration. Data was then fitted to the Michaelis Menten model with V_{max} fixed at 100 %.

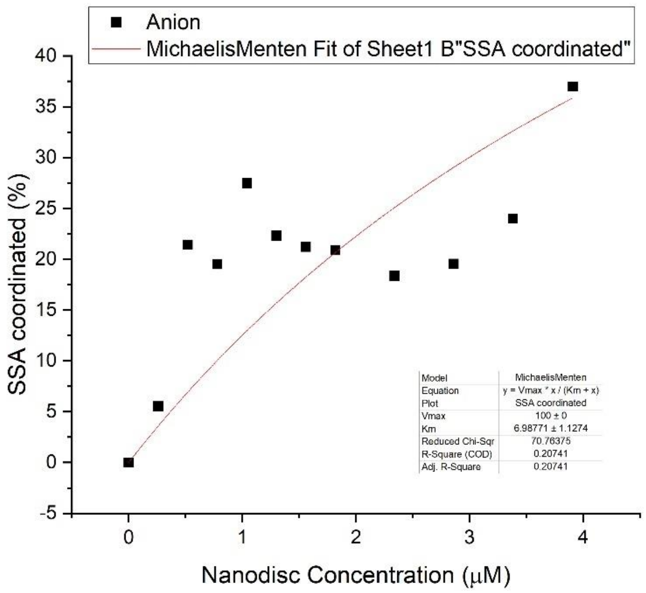

Figure S135 - Graph showing the percentage of SSA **5** anion coordinated to PG nanodiscs, with respect to increasing nanodisc concentration. Data was then fitted to the Michaelis Menten model with V_{max} fixed at 100 %.

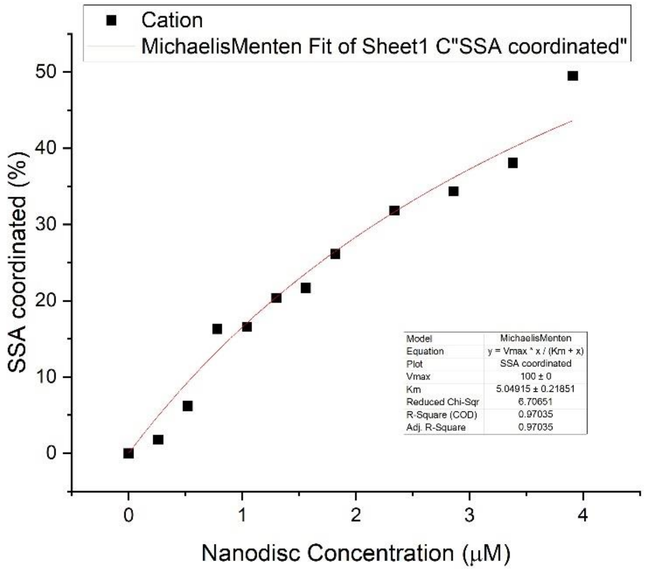

Figure S136 - Graph showing the percentage of SSA **5** cation coordinated to PG nanodiscs, with respect to increasing nanodisc concentration. Data was then fitted to the Michaelis Menten model with V_{max} fixed at 100 %.

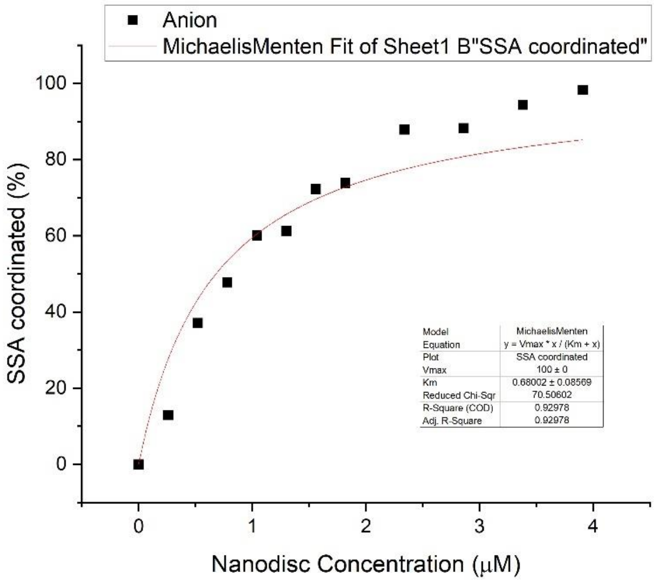

Figure S137 - Graph showing the percentage of SSA **6** anion coordinated to PG nanodiscs, with respect to increasing nanodisc concentration. Data was then fitted to the Michaelis Menten model with V_{max} fixed at 100 %.

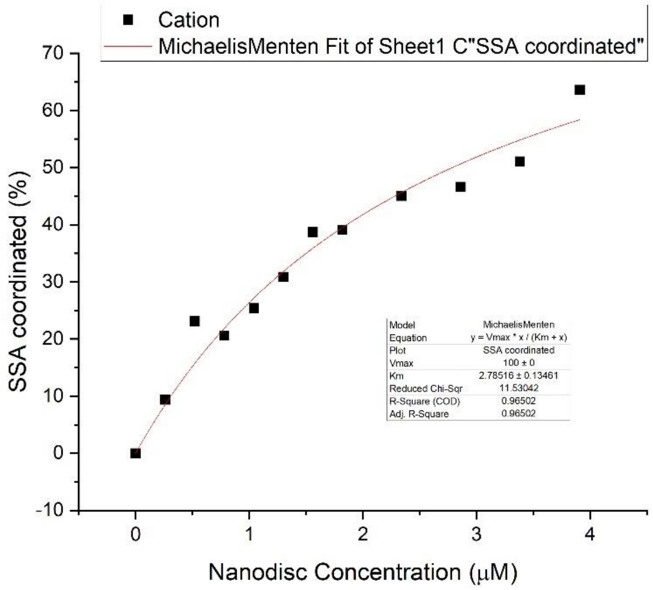

Figure S138 - Graph showing the percentage of SSA **6** cation coordinated to PG nanodiscs, with respect to increasing nanodisc concentration. Data was then fitted to the Michaelis Menten model with V_{max} fixed at 100 %.

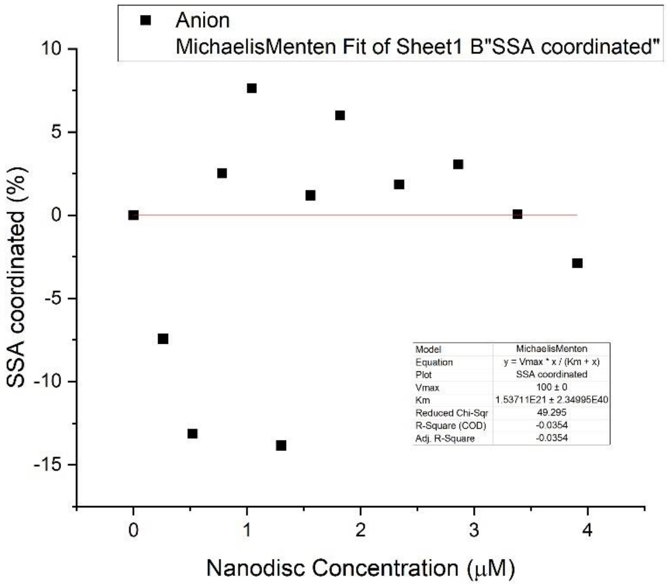

Figure S139 - Graph showing the percentage of SSA **7** anion coordinated to PG nanodiscs, with respect to increasing nanodisc concentration. Data was then fitted to the Michaelis Menten model with V_{max} fixed at 100 %.

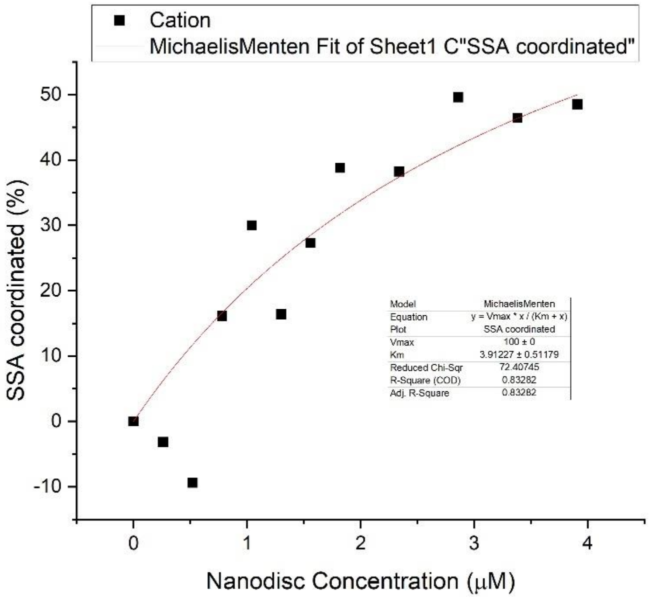

Figure S140 - Graph showing the percentage of SSA **7** cation coordinated to PG nanodiscs, with respect to increasing nanodisc concentration. Data was then fitted to the Michaelis Menten model with V_{max} fixed at 100 %.

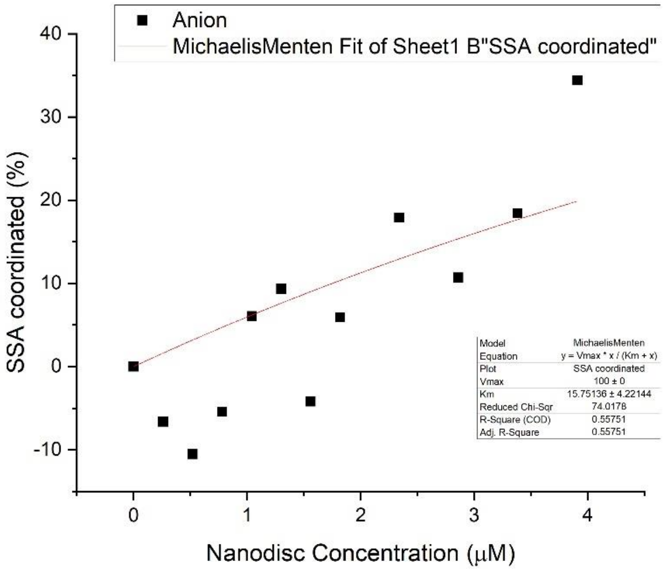

Figure S141 - Graph showing the percentage of SSA **8** anion coordinated to PG nanodiscs, with respect to increasing nanodisc concentration. Data was then fitted to the Michaelis Menten model with V_{max} fixed at 100 %.

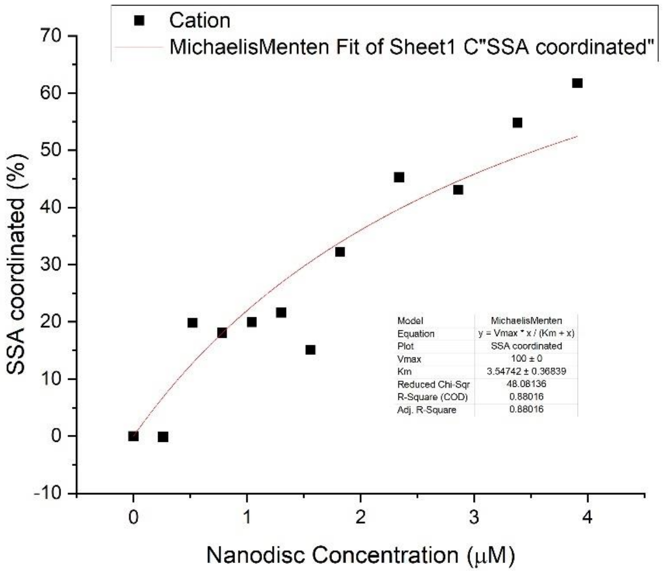

Figure S142 - Graph showing the percentage of SSA **8** cation coordinated to PG nanodiscs, with respect to increasing nanodisc concentration. Data was then fitted to the Michaelis Menten model with V_{max} fixed at 100 %.

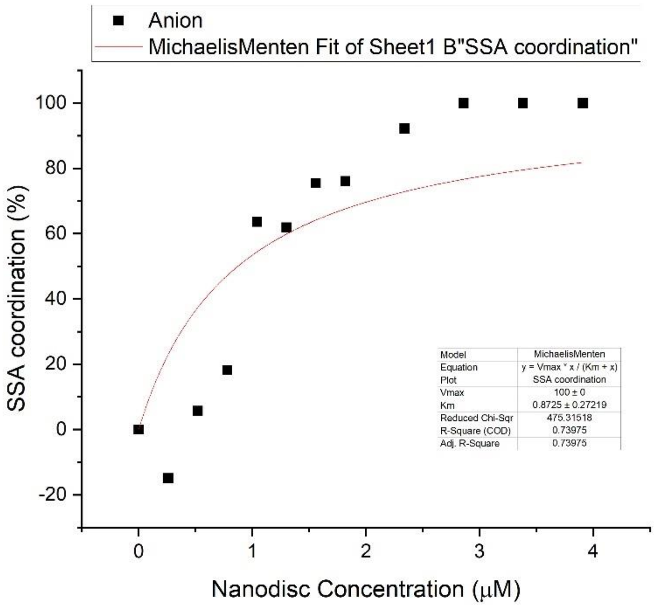

Figure S143 - Graph showing the percentage of SSA **9** anion coordinated to PG nanodiscs, with respect to increasing nanodisc concentration. Data was then fitted to the Michaelis Menten model with V_{max} fixed at 100 %.

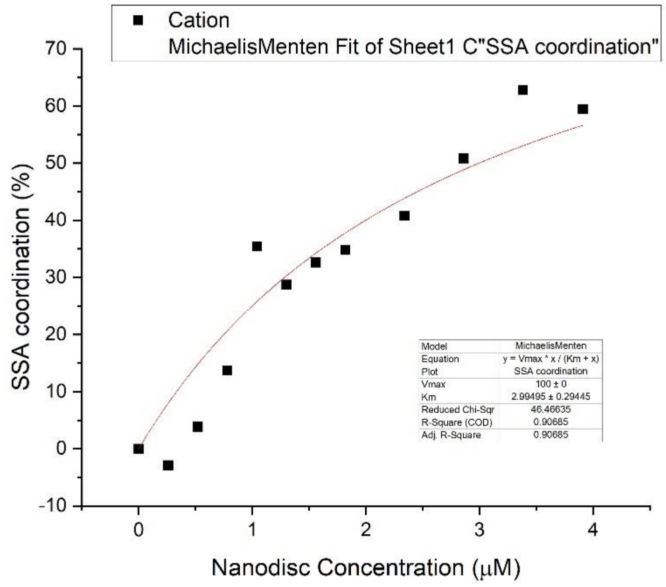

Figure S144 - Graph showing the percentage of SSA **9** cation coordinated to PG nanodiscs, with respect to increasing nanodisc concentration. Data was then fitted to the Michaelis Menten model with V_{max} fixed at 100 %.

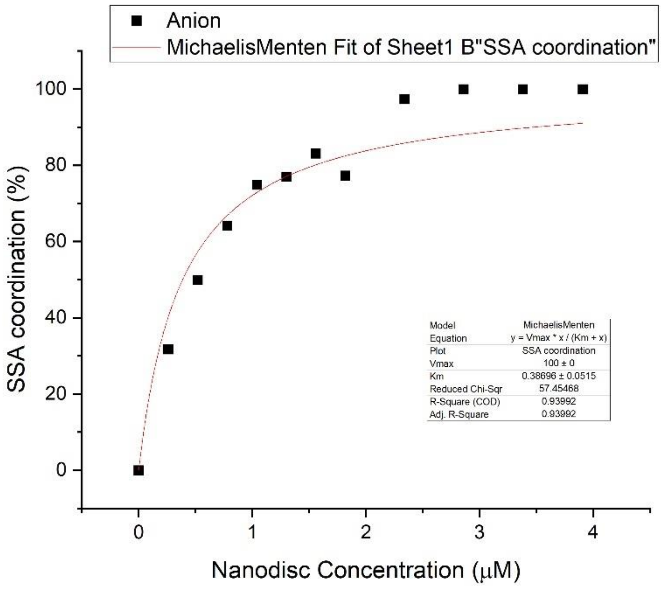

Figure S145 - Graph showing the percentage of SSA **10** anion coordinated to PG nanodiscs, with respect to increasing nanodisc concentration. Data was then fitted to the Michaelis Menten model with V_{max} fixed at 100 %.

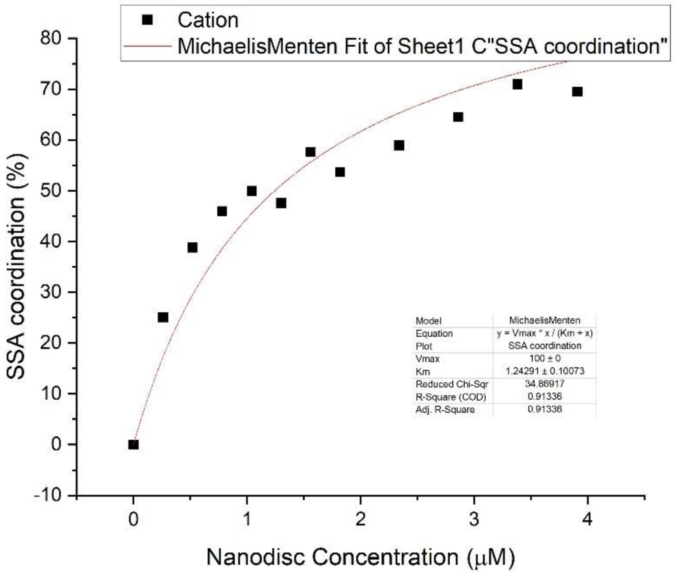

Figure S146 - Graph showing the percentage of SSA **10** cation coordinated to PG nanodiscs, with respect to increasing nanodisc concentration. Data was then fitted to the Michaelis Menten model with V_{max} fixed at 100 %.

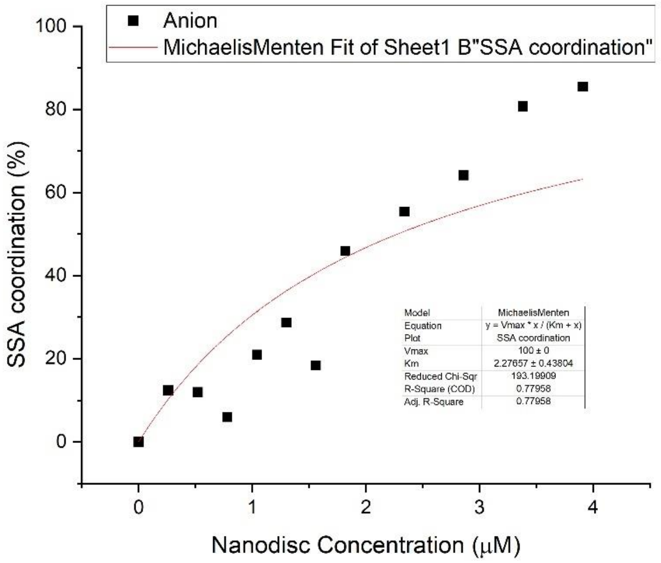

Figure S147 - Graph showing the percentage of SSA **11** anion coordinated to PG nanodiscs, with respect to increasing nanodisc concentration. Data was then fitted to the Michaelis Menten model with V_{max} fixed at 100 %.

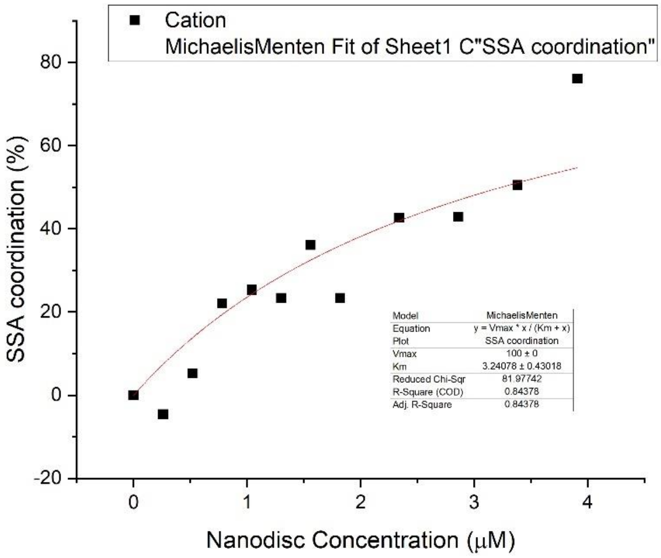

Figure S148 - Graph showing the percentage of SSA **11** cation coordinated to PG nanodiscs, with respect to increasing nanodisc concentration. Data was then fitted to the Michaelis Menten model with V_{max} fixed at 100 %.

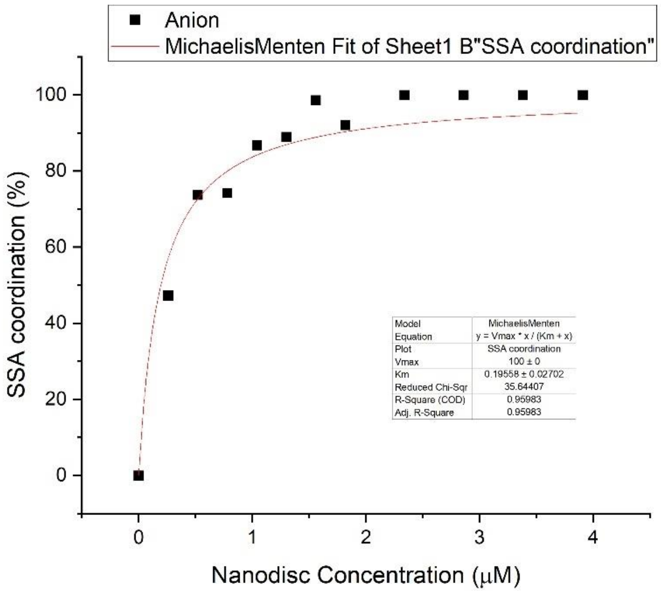

Figure S149 - Graph showing the percentage of SSA **11** anion coordinated to PG nanodiscs, with respect to increasing nanodisc concentration. Data was then fitted to the Michaelis Menten model with V_{max} fixed at 100 %.

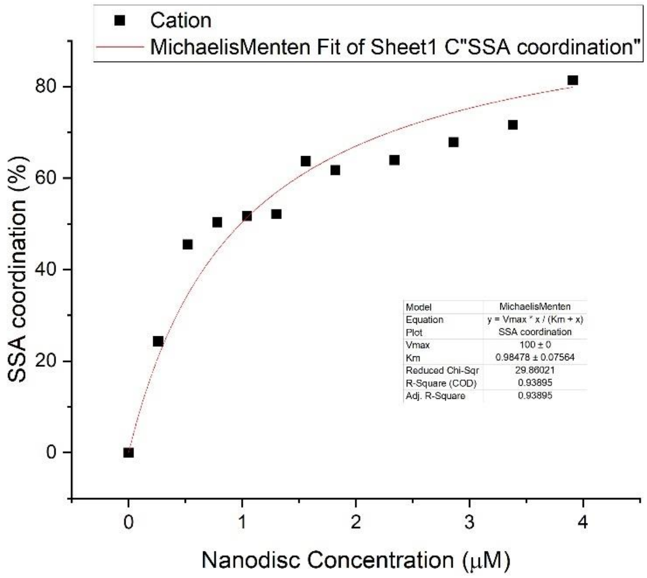

Figure S150 - Graph showing the percentage of SSA **11** cation coordinated to PG nanodiscs, with respect to increasing nanodisc concentration. Data was then fitted to the Michaelis Menten model with V_{max} fixed at 100 %.

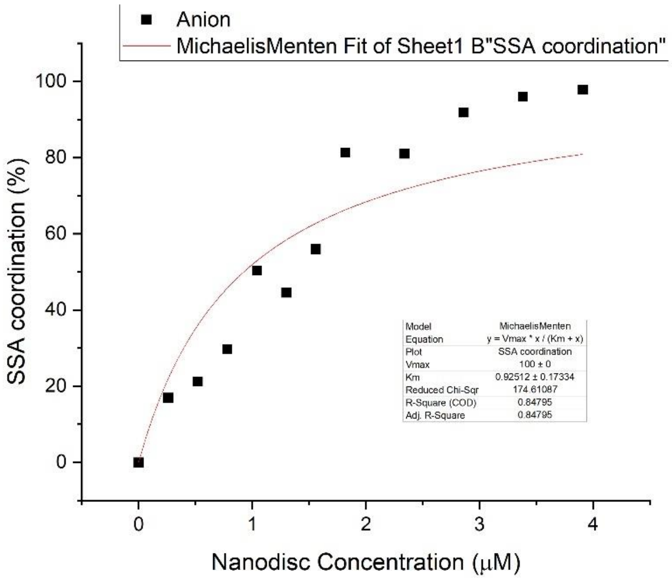

Figure S151 - Graph showing the percentage of SSA **5** anion coordinated to PE:PG 3:1 mix nanodiscs, with respect to increasing nanodisc concentration. Data was then fitted to the Michaelis Menten model with V_{max} fixed at 100 %.

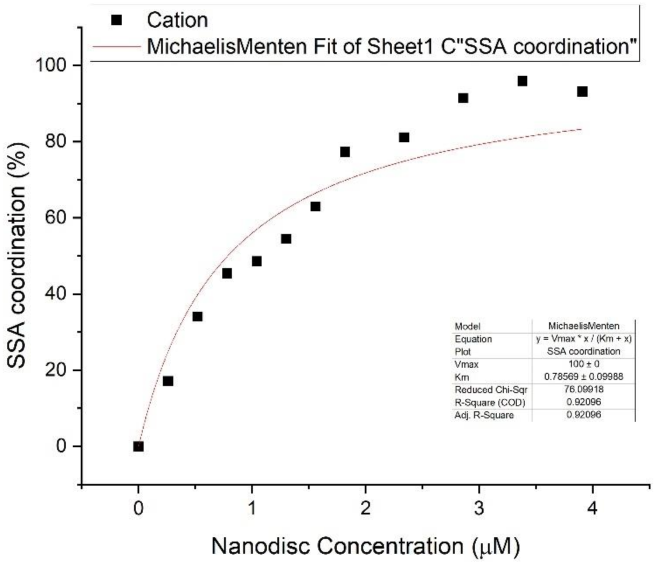

Figure S152 - Graph showing the percentage of SSA **5** cation coordinated to PE:PG 3:1 mix nanodiscs, with respect to increasing nanodisc concentration. Data was then fitted to the Michaelis Menten model with V_{max} fixed at 100 %.

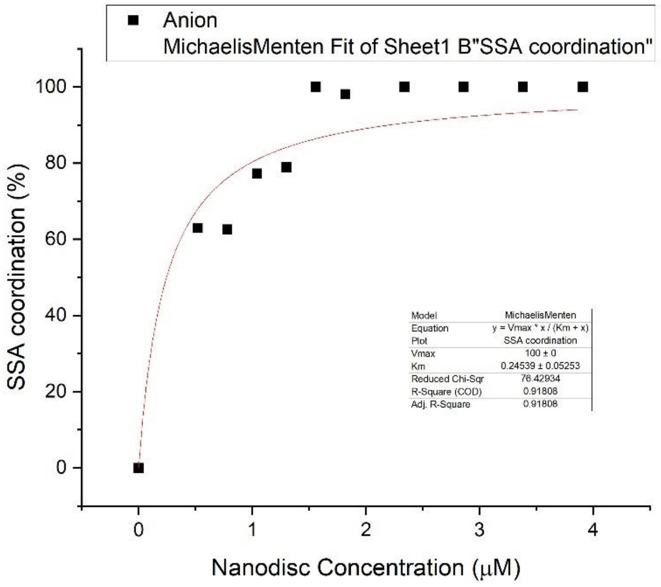

Figure S153 - Graph showing the percentage of SSA **6** anion coordinated to PE:PG 3:1 mix nanodiscs, with respect to increasing nanodisc concentration. Data was then fitted to the Michaelis Menten model with V_{max} fixed at 100 %.

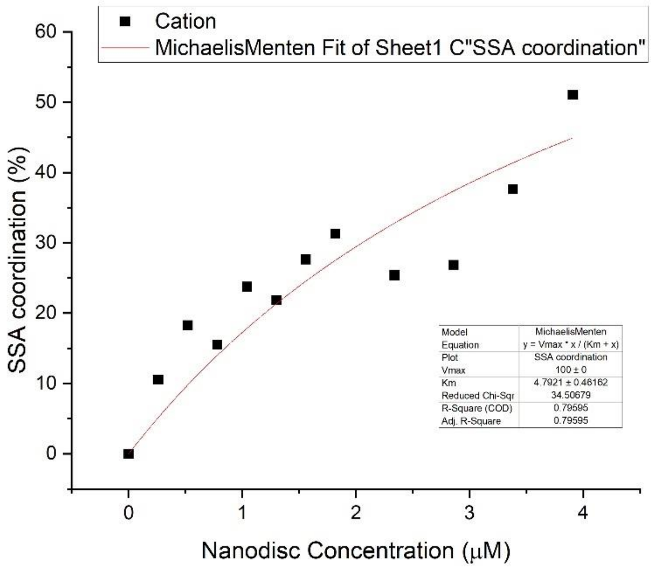

Figure S154 - Graph showing the percentage of SSA **6** cation coordinated to PE:PG 3:1 mix nanodiscs, with respect to increasing nanodisc concentration. Data was then fitted to the Michaelis Menten model with V_{max} fixed at 100 %.

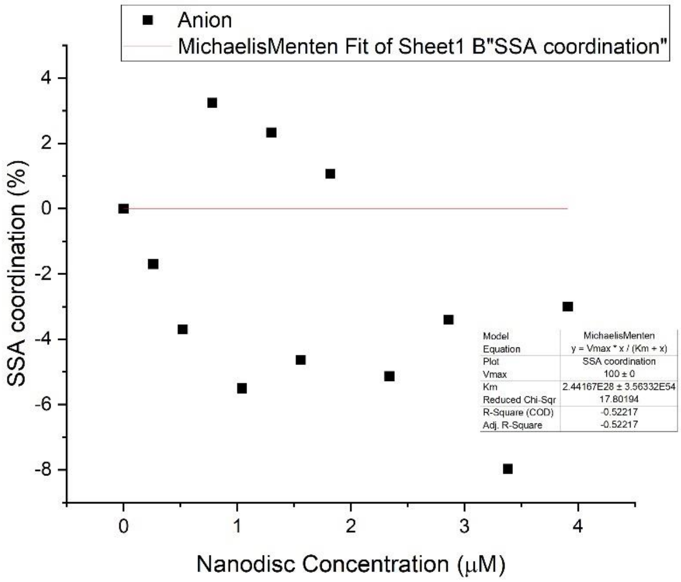

Figure S155 - Graph showing the percentage of SSA **7** anion coordinated to PE:PG 3:1 mix nanodiscs, with respect to increasing nanodisc concentration. Data was then fitted to the Michaelis Menten model with V_{max} fixed at 100 %.

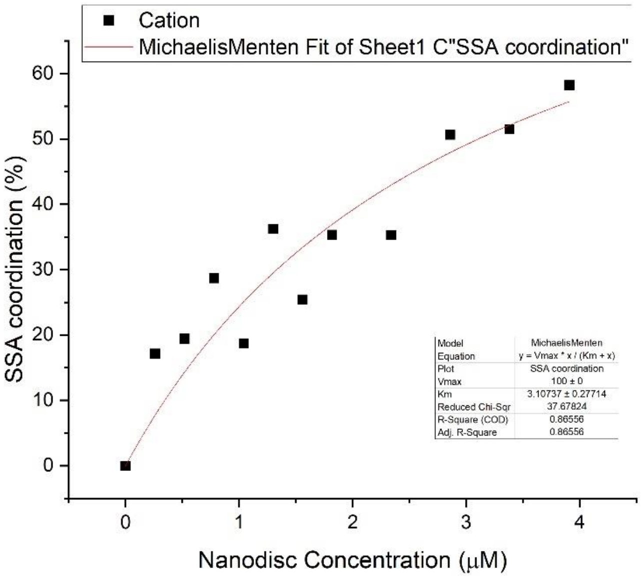

Figure S156 - Graph showing the percentage of SSA **7** cation coordinated to PE:PG 3:1 mix nanodiscs, with respect to increasing nanodisc concentration. Data was then fitted to the Michaelis Menten model with V_{max} fixed at 100 %.

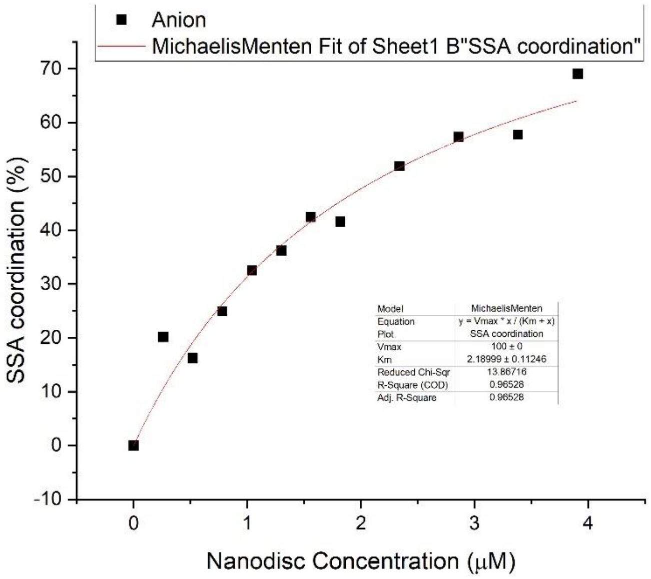

Figure S157 - Graph showing the percentage of SSA **8** anion coordinated to PE:PG 3:1 mix nanodiscs, with respect to increasing nanodisc concentration. Data was then fitted to the Michaelis Menten model with V_{max} fixed at 100 %.

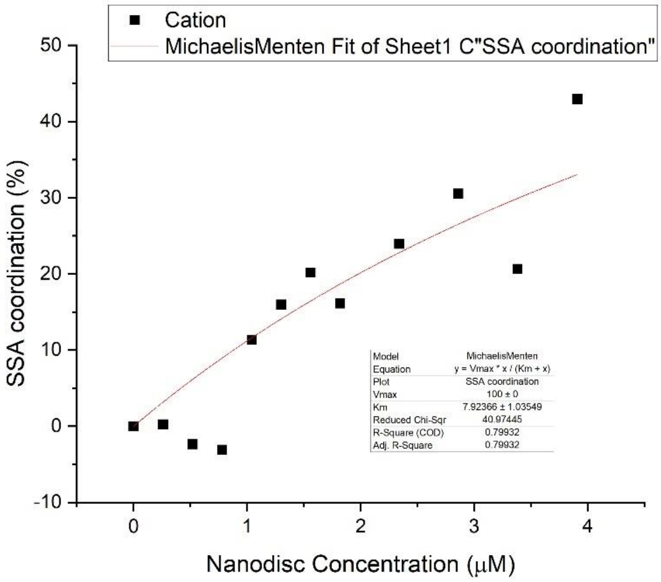

Figure S158 - Graph showing the percentage of SSA **8** cation coordinated to PE:PG 3:1 mix nanodiscs, with respect to increasing nanodisc concentration. Data was then fitted to the Michaelis Menten model with V_{max} fixed at 100 %.

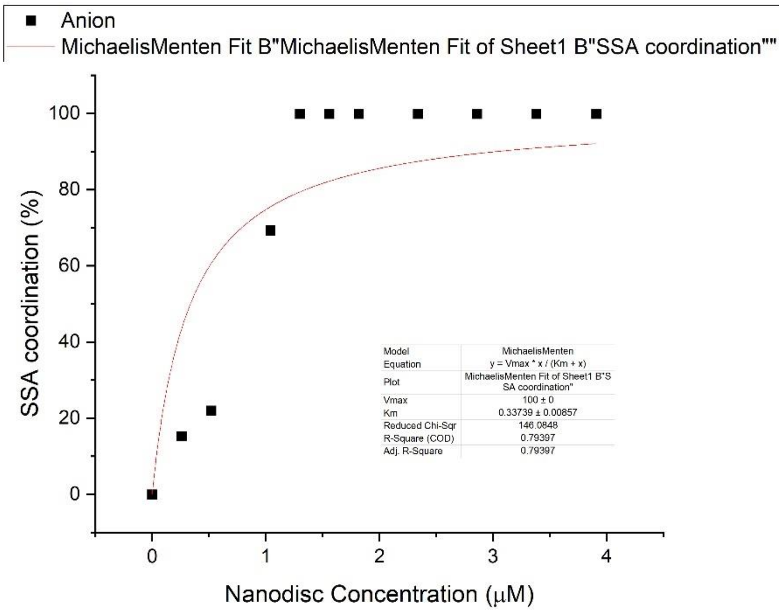

Figure S159 - Graph showing the percentage of SSA **9** anion coordinated to PE:PG 3:1 mix nanodiscs, with respect to increasing nanodisc concentration. Data was then fitted to the Michaelis Menten model with V_{max} fixed at 100 %.

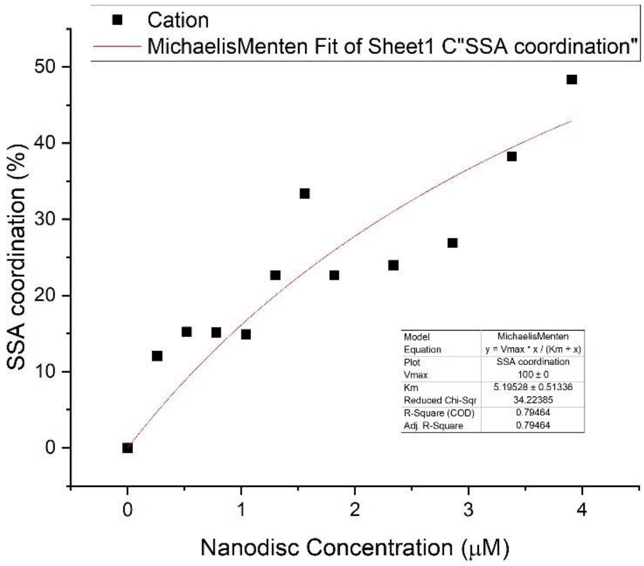

Figure S160 - Graph showing the percentage of SSA **9** cation coordinated to PE:PG 3:1 mix nanodiscs, with respect to increasing nanodisc concentration. Data was then fitted to the Michaelis Menten model with V_{max} fixed at 100 %.

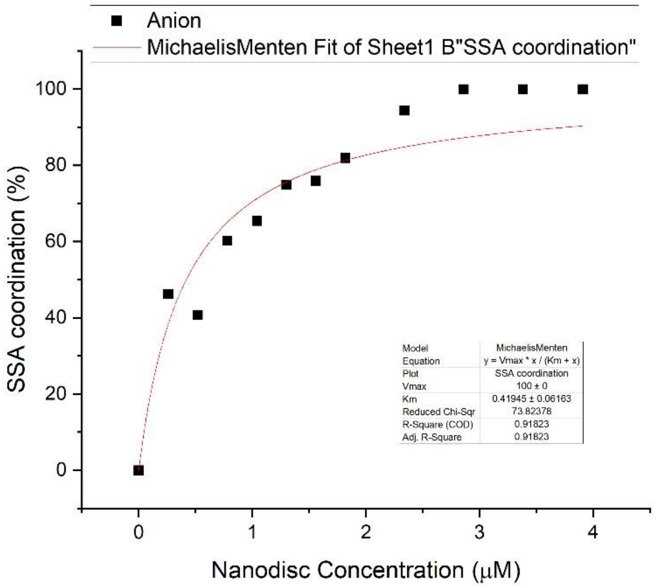

Figure S161 - Graph showing the percentage of SSA **10** anion coordinated to PE:PG 3:1 mix nanodiscs, with respect to increasing nanodisc concentration. Data was then fitted to the Michaelis Menten model with V_{max} fixed at 100 %.

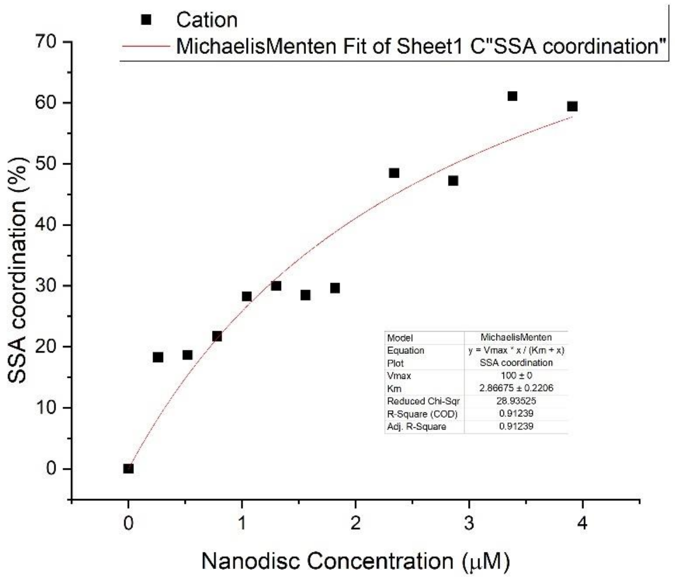

Figure S162 - Graph showing the percentage of SSA **10** cation coordinated to PE:PG 3:1 mix nanodiscs, with respect to increasing nanodisc concentration. Data was then fitted to the Michaelis Menten model with V_{max} fixed at 100 %.

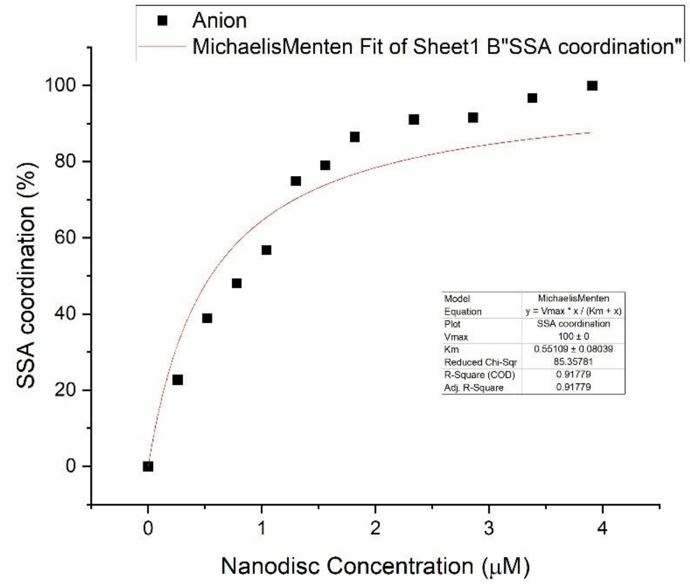

Figure S163 - Graph showing the percentage of SSA **11** anion coordinated to PE:PG 3:1 mix nanodiscs, with respect to increasing nanodisc concentration. Data was then fitted to the Michaelis Menten model with V_{max} fixed at 100 %.

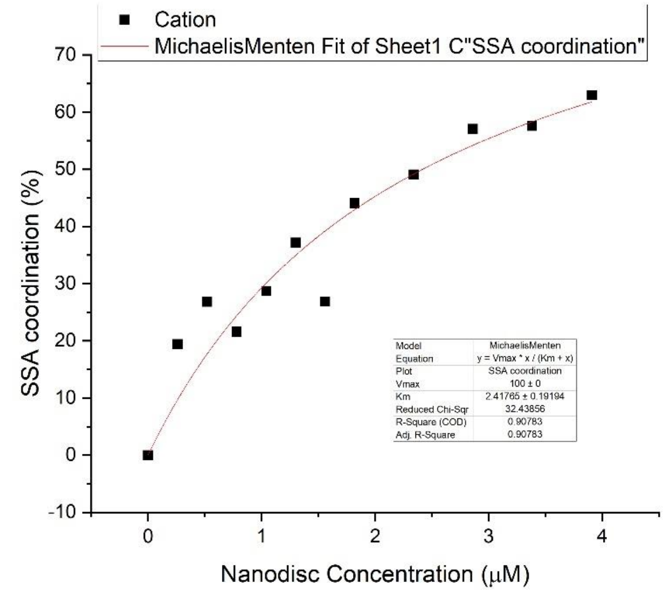

Figure S164 - Graph showing the percentage of SSA **11** cation coordinated to PE:PG 3:1 mix nanodiscs, with respect to increasing nanodisc concentration. Data was then fitted to the Michaelis Menten model with V_{max} fixed at 100 %.

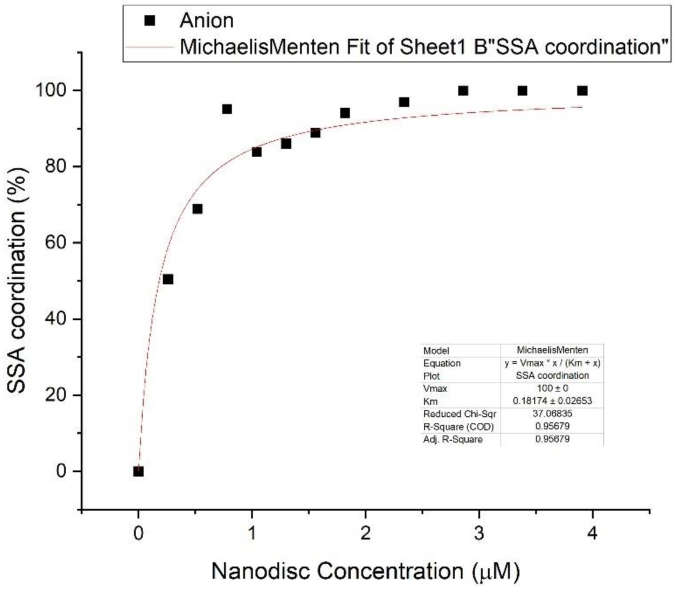

Figure S165 - Graph showing the percentage of SSA **12** anion coordinated to PE:PG 3:1 mix nanodiscs, with respect to increasing nanodisc concentration. Data was then fitted to the Michaelis Menten model with V_{max} fixed at 100 %.

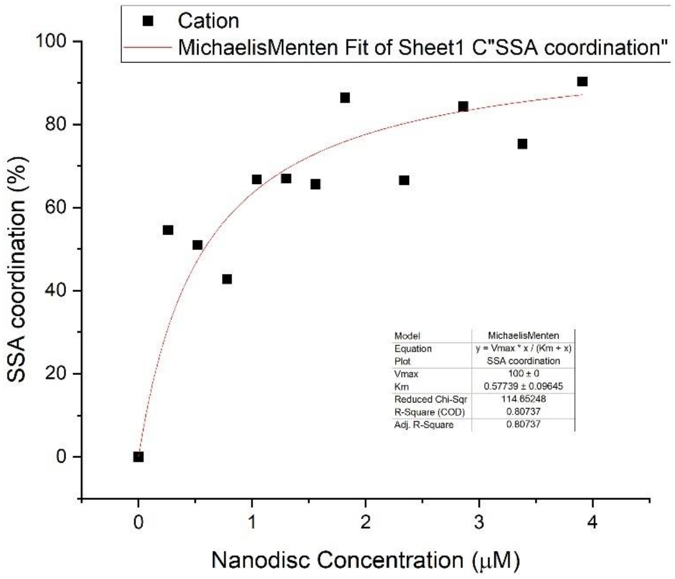

Figure S166 - Graph showing the percentage of SSA **12** cation coordinated to PE:PG 3:1 mix nanodiscs, with respect to increasing nanodisc concentration. Data was then fitted to the Michaelis Menten model with V_{max} fixed at 100 %.

Hill Plot fits

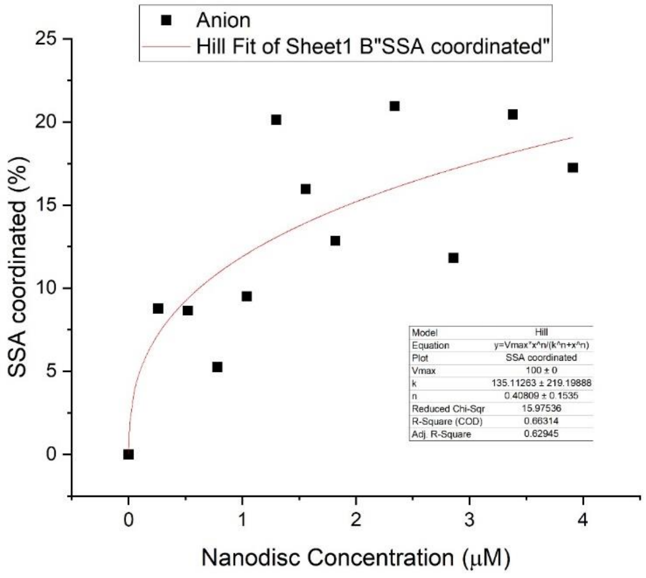

Figure S167 - Graph showing the percentage of SSA **5** anion coordinated to PC nanodiscs, with respect to increasing nanodisc concentration. Data was then fitted to the Hill model with V_{max} fixed at 100 %.

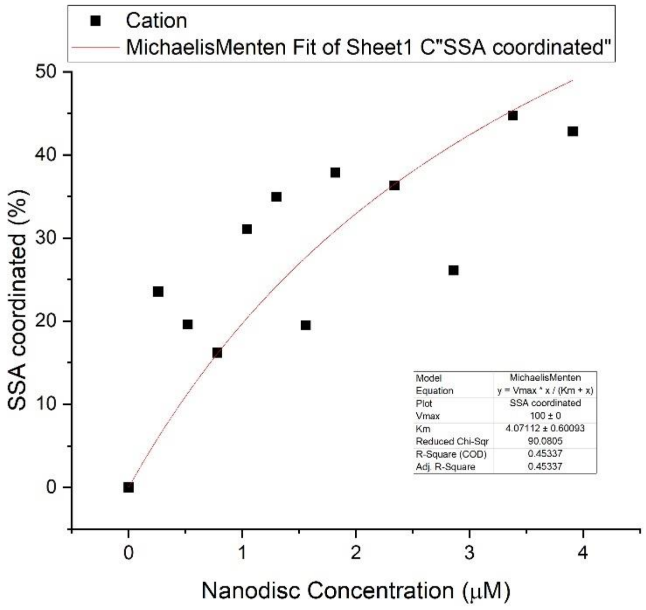

Figure S168 - Graph showing the percentage of SSA **5** cation coordinated to PC nanodiscs, with respect to increasing nanodisc concentration. Data was then fitted to the Hill model with V_{max} fixed at 100 %.

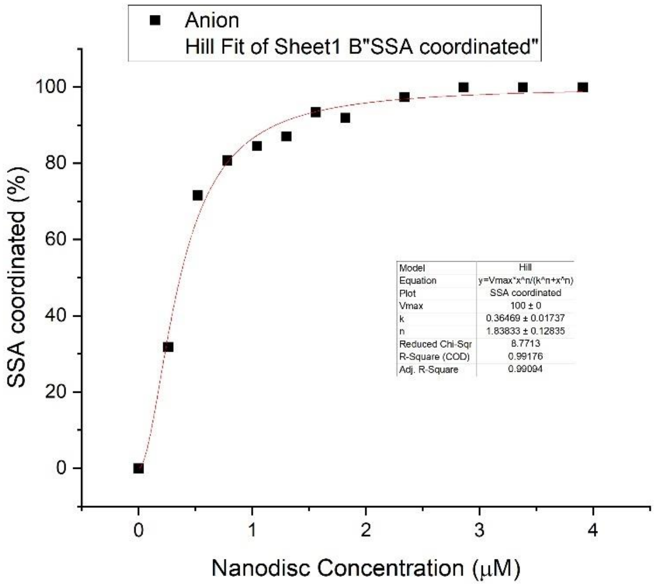

Figure S169 - Graph showing the percentage of SSA **6** anion coordinated to PC nanodiscs, with respect to increasing nanodisc concentration. Data was then fitted to the Hill model with V_{max} fixed at 100 %.

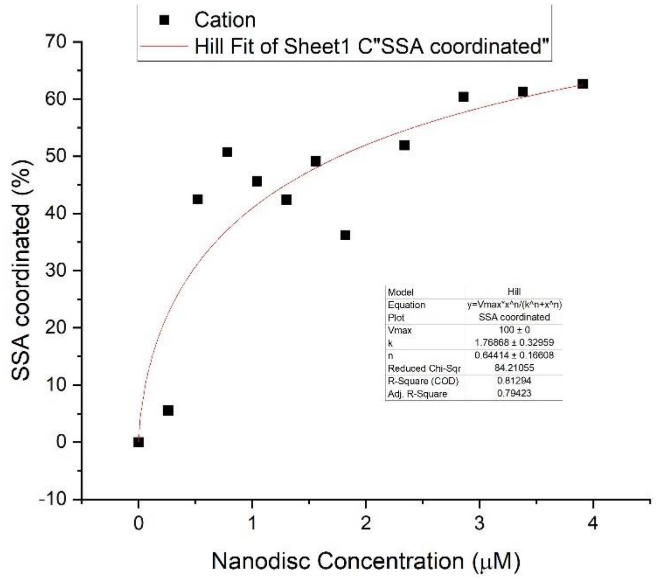

Figure S170 - Graph showing the percentage of SSA **6** cation coordinated to PC nanodiscs, with respect to increasing nanodisc concentration. Data was then fitted to the Hill model with V_{max} fixed at 100 %.

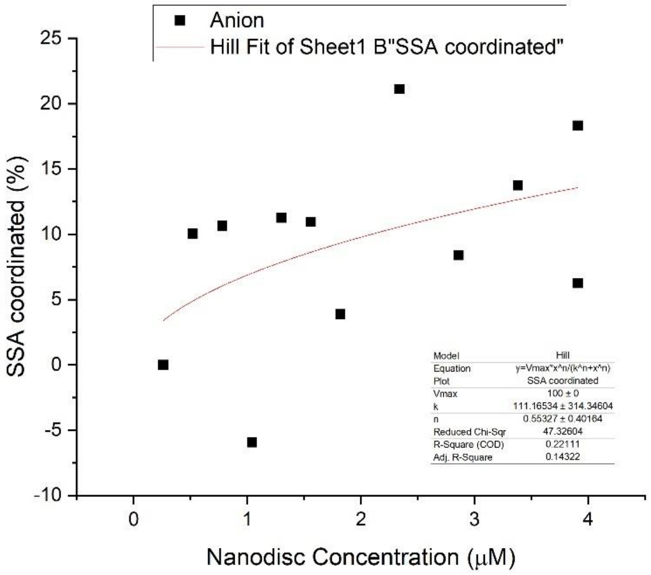

Figure S171 - Graph showing the percentage of SSA **7** anion coordinated to PC nanodiscs, with respect to increasing nanodisc concentration. Data was then fitted to the Hill model with V_{max} fixed at 100 %.

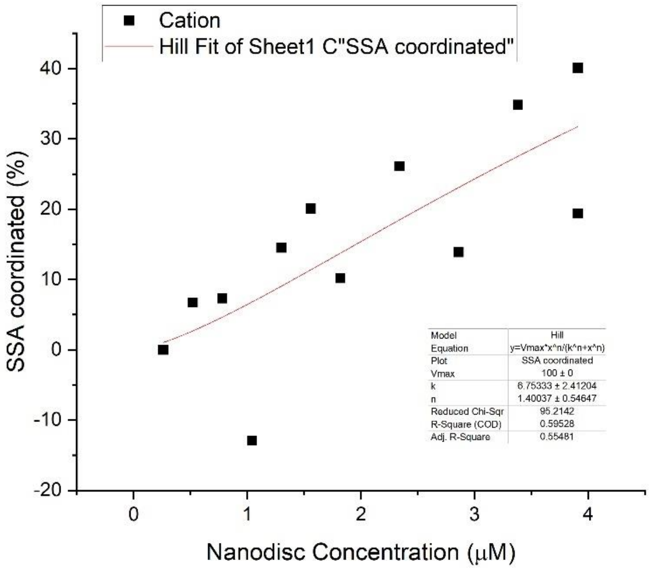

Figure S172 - Graph showing the percentage of SSA **7** cation coordinated to PC nanodiscs, with respect to increasing nanodisc concentration. Data was then fitted to the Hill model with V_{max} fixed at 100 %.

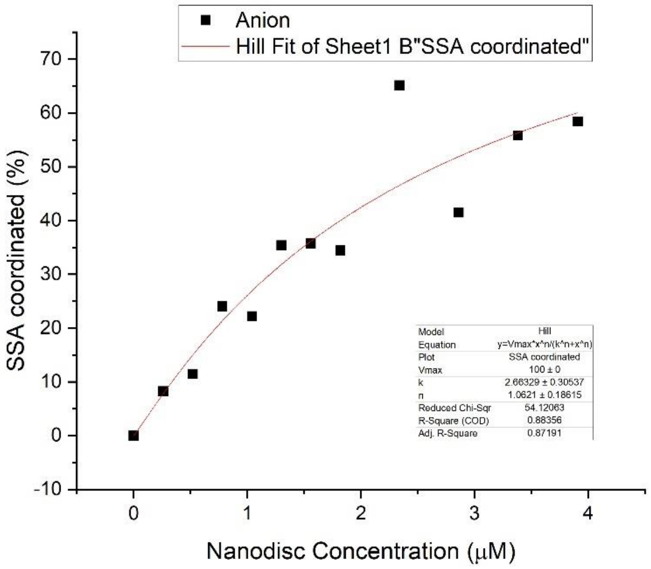

Figure S173 - Graph showing the percentage of SSA **8** anion coordinated to PC nanodiscs, with respect to increasing nanodisc concentration. Data was then fitted to the Hill model with V_{max} fixed at 100 %.

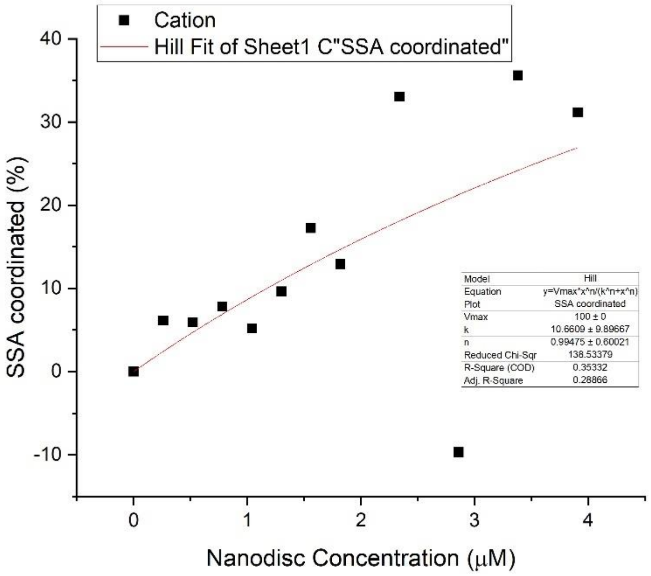

Figure S174 - Graph showing the percentage of SSA **8** cation coordinated to PC nanodiscs, with respect to increasing nanodisc concentration. Data was then fitted to the Hill model with V_{max} fixed at 100 %.

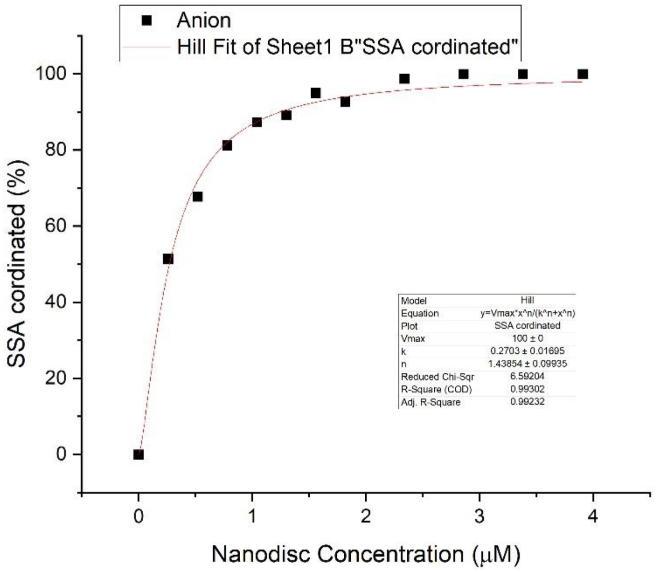

Figure S175 - Graph showing the percentage of SSA **9** anion coordinated to PC nanodiscs, with respect to increasing nanodisc concentration. Data was then fitted to the Hill model with V_{max} fixed at 100 %.

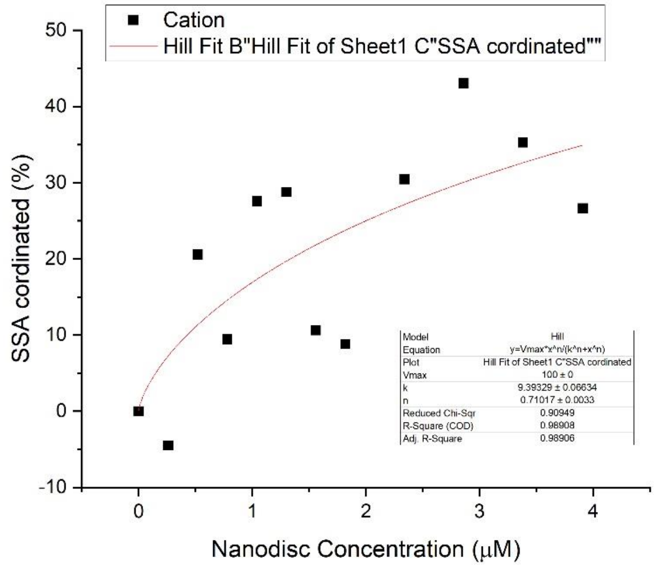

Figure S176 - Graph showing the percentage of SSA **9** cation coordinated to PC nanodiscs, with respect to increasing nanodisc concentration. Data was then fitted to the Hill model with V_{max} fixed at 100 %.

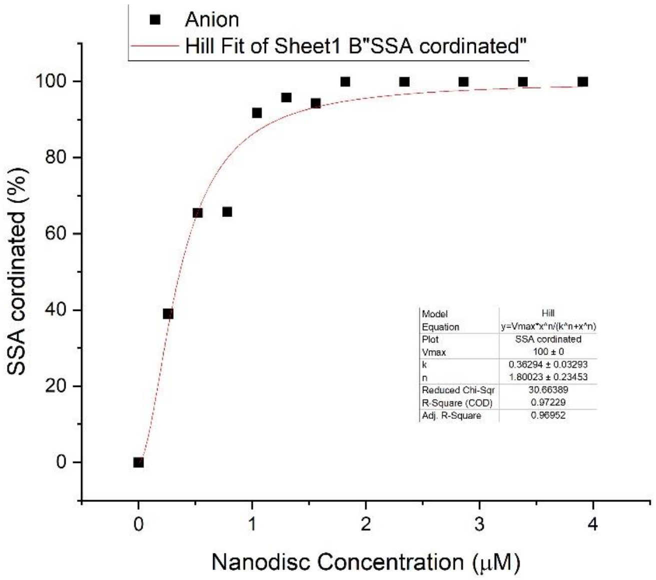

Figure S177 - Graph showing the percentage of SSA **10** anion coordinated to PC nanodiscs, with respect to increasing nanodisc concentration. Data was then fitted to the Hill model with V_{max} fixed at 100 %.

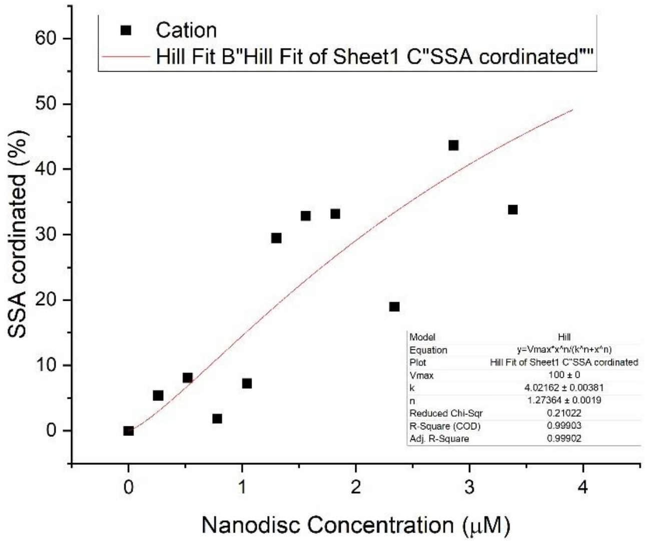

Figure S178 - Graph showing the percentage of SSA **10** cation coordinated to PC nanodiscs, with respect to increasing nanodisc concentration. Data was then fitted to the Hill model with V_{max} fixed at 100 %.

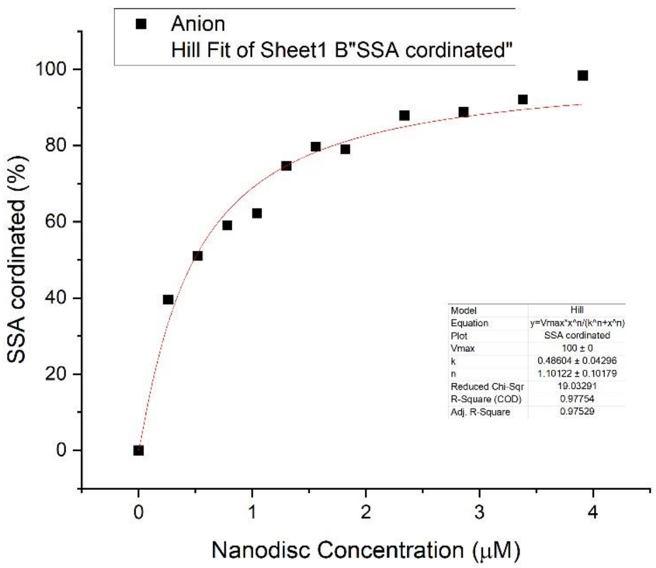

Figure S179 - Graph showing the percentage of SSA **11** anion coordinated to PC nanodiscs, with respect to increasing nanodisc concentration. Data was then fitted to the Hill model with V_{max} fixed at 100 %.

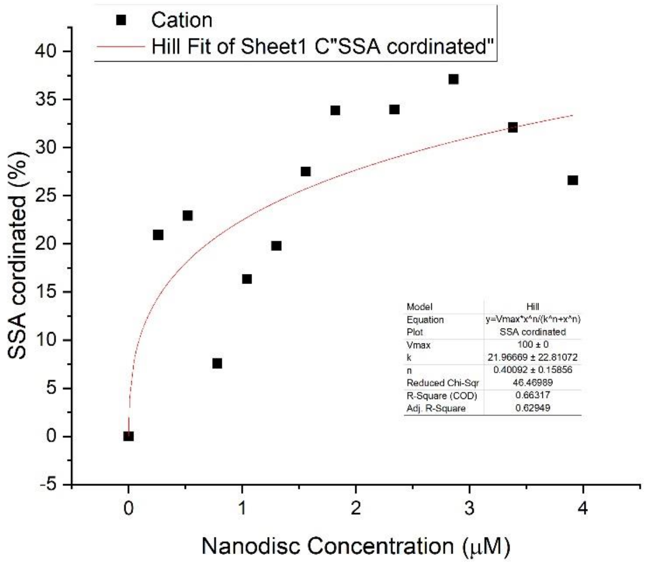

Figure S180 - Graph showing the percentage of SSA **11** cation coordinated to PC nanodiscs, with respect to increasing nanodisc concentration. Data was then fitted to the Hill model with V_{max} fixed at 100 %.

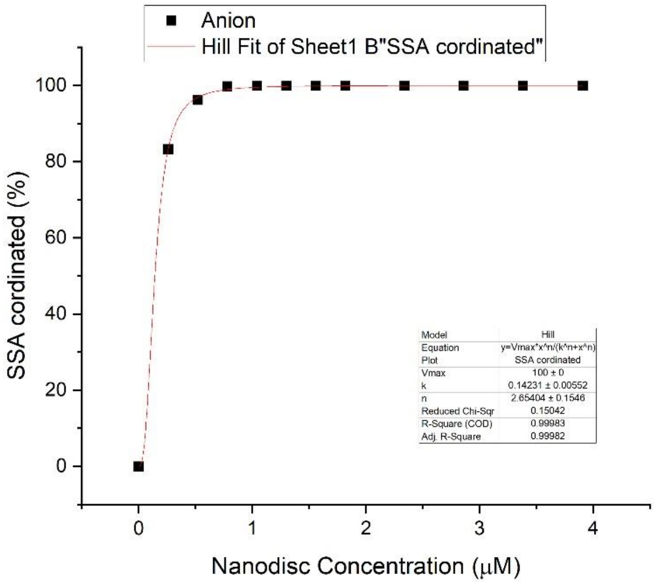

Figure S181 - Graph showing the percentage of SSA **12** anion coordinated to PC nanodiscs, with respect to increasing nanodisc concentration. Data was then fitted to the Hill model with V_{max} fixed at 100 %.

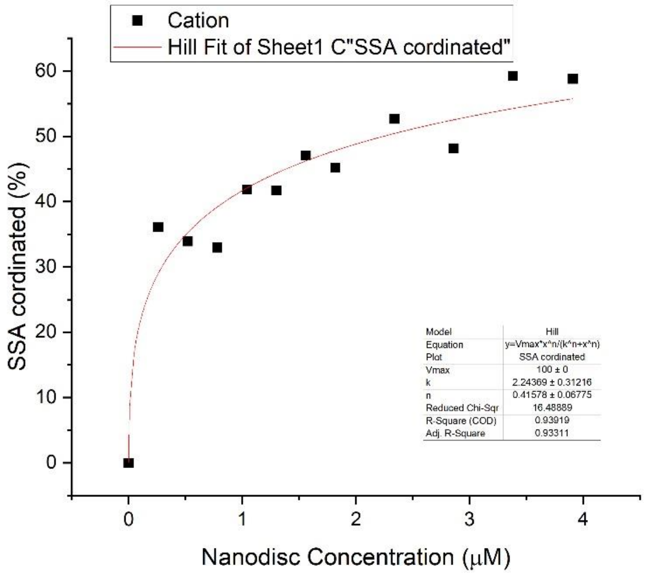

Figure S182 - Graph showing the percentage of SSA **12** cation coordinated to PC nanodiscs, with respect to increasing nanodisc concentration. Data was then fitted to the Hill model with V_{max} fixed at 100 %.

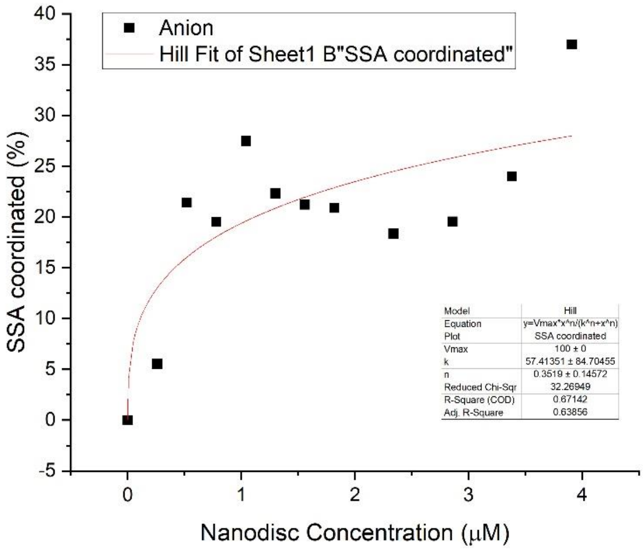

Figure S183 - Graph showing the percentage of SSA **5** anion coordinated to PG nanodiscs, with respect to increasing nanodisc concentration. Data was then fitted to the Hill model with V_{max} fixed at 100 %.

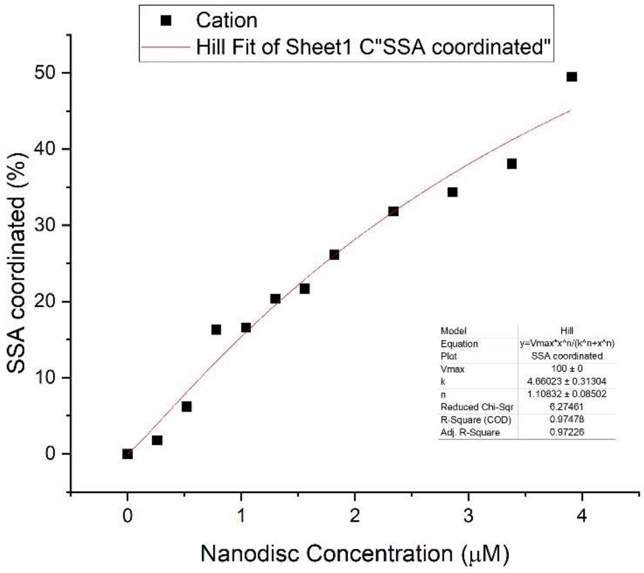

Figure S184 - Graph showing the percentage of SSA **5** cation coordinated to PG nanodiscs, with respect to increasing nanodisc concentration. Data was then fitted to the Hill model with V_{max} fixed at 100 %.

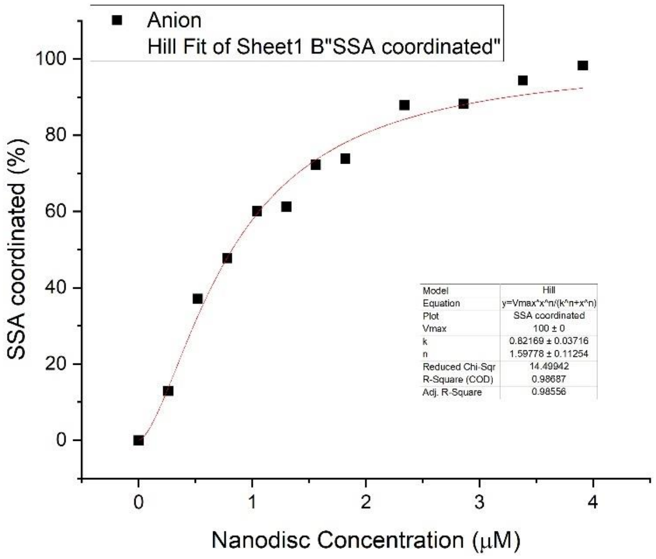

Figure S185 - Graph showing the percentage of SSA **6** anion coordinated to PG nanodiscs, with respect to increasing nanodisc concentration. Data was then fitted to the Hill model with V_{max} fixed at 100 %.

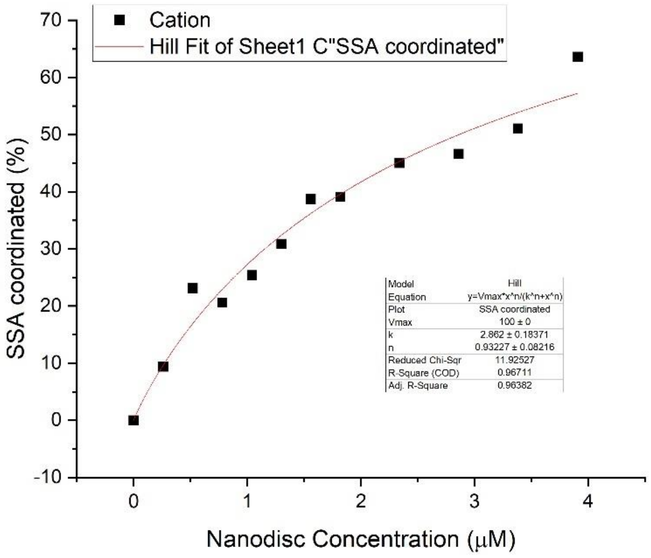

Figure S186 - Graph showing the percentage of SSA **6** cation coordinated to PG nanodiscs, with respect to increasing nanodisc concentration. Data was then fitted to the Hill model with V_{max} fixed at 100 %.

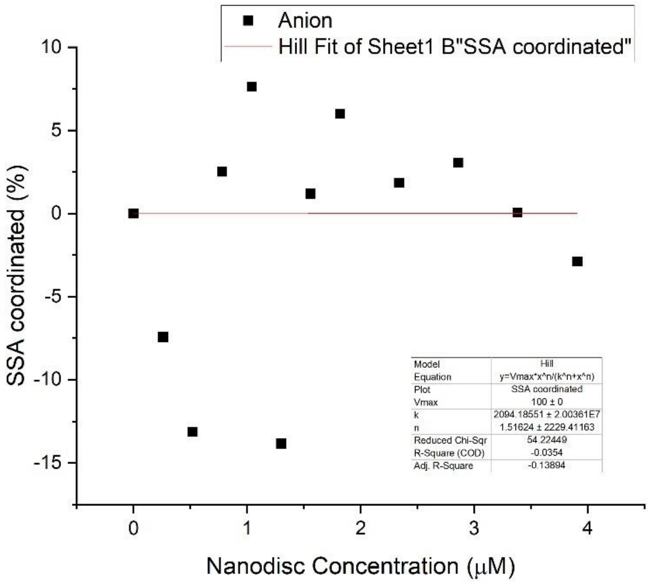

Figure S187 - Graph showing the percentage of SSA **7** anion coordinated to PG nanodiscs, with respect to increasing nanodisc concentration. Data was then fitted to the Hill model with V_{max} fixed at 100 %.

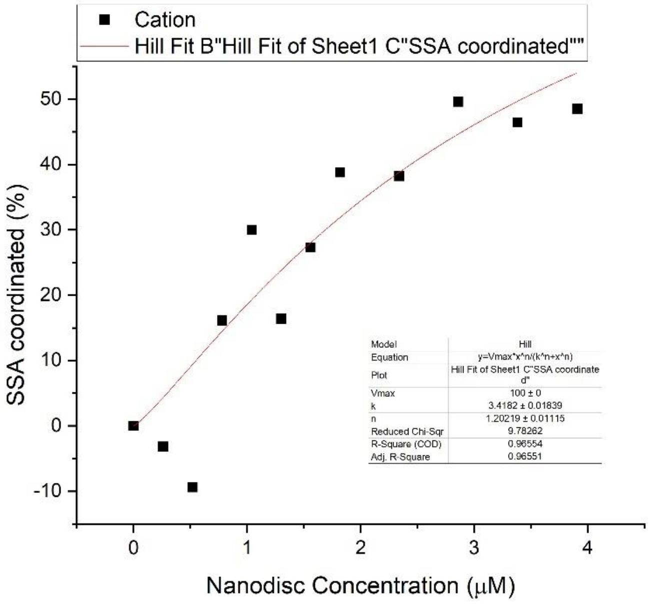

Figure S188 - Graph showing the percentage of SSA **7** cation coordinated to PG nanodiscs, with respect to increasing nanodisc concentration. Data was then fitted to the Hill model with V_{max} fixed at 100 %.

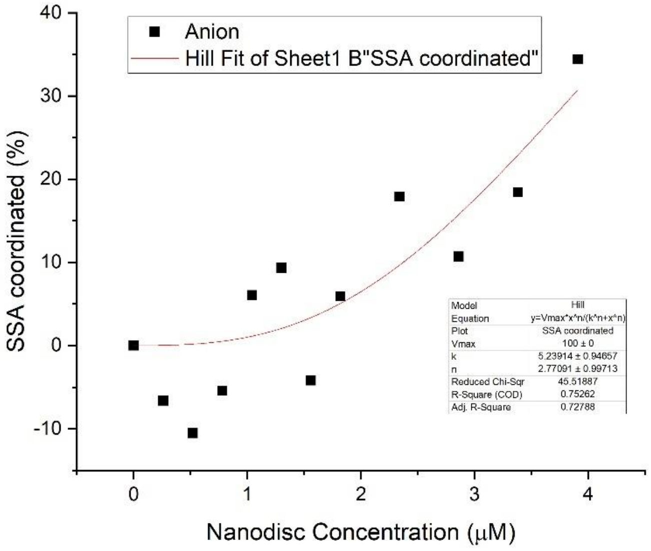

Figure S189 - Graph showing the percentage of SSA **8** anion coordinated to PG nanodiscs, with respect to increasing nanodisc concentration. Data was then fitted to the Hill model with V_{max} fixed at 100 %.

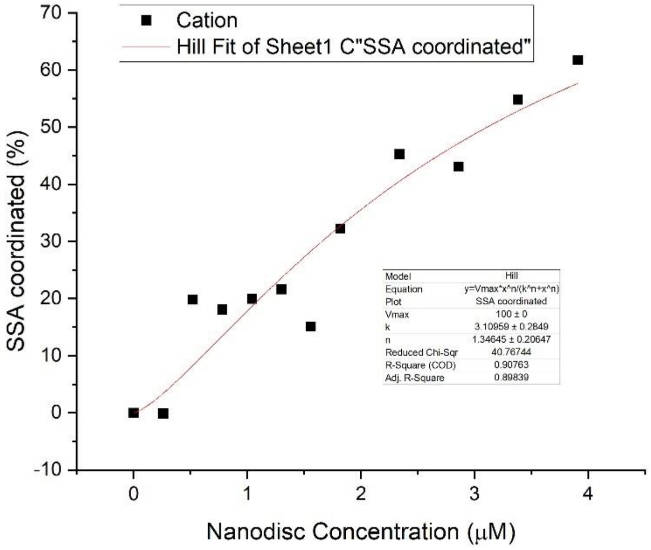

Figure S190 - Graph showing the percentage of SSA **8** cation coordinated to PG nanodiscs, with respect to increasing nanodisc concentration. Data was then fitted to the Hill model with V_{max} fixed at 100 %.

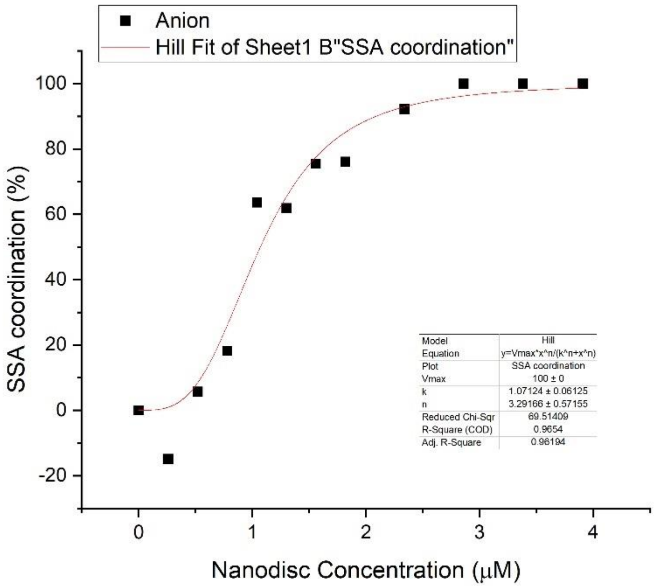

Figure S191 - Graph showing the percentage of SSA **9** anion coordinated to PG nanodiscs, with respect to increasing nanodisc concentration. Data was then fitted to the Hill model with V_{max} fixed at 100 %.

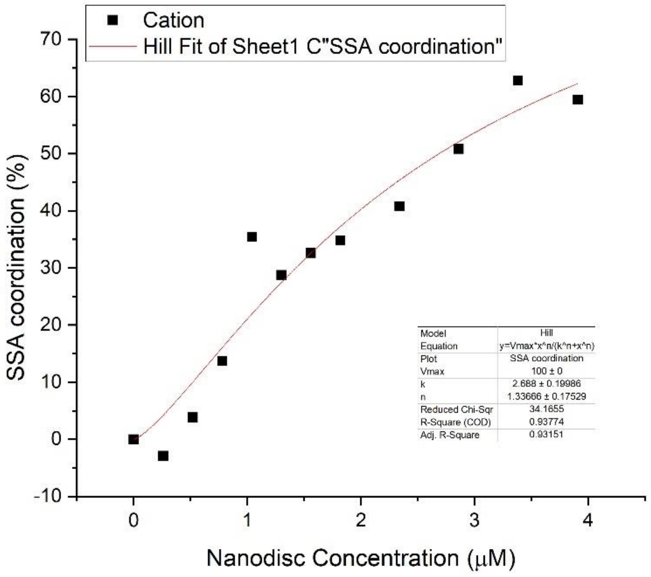

Figure S192 - Graph showing the percentage of SSA **9** cation coordinated to PG nanodiscs, with respect to increasing nanodisc concentration. Data was then fitted to the Hill model with V_{max} fixed at 100 %.

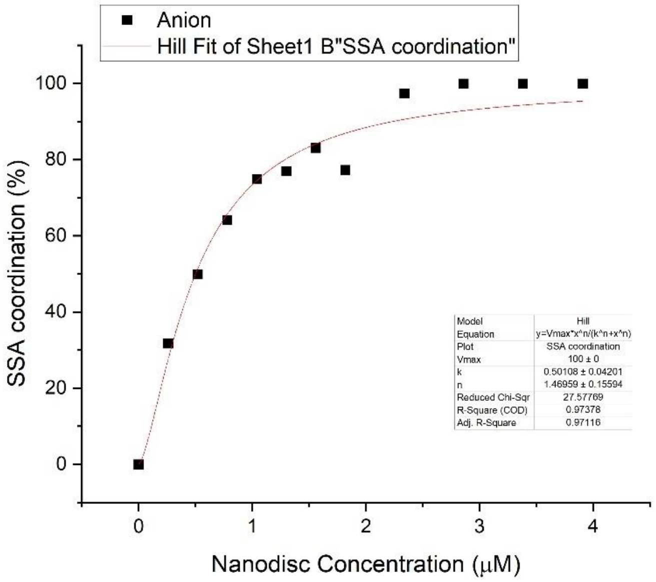

Figure S193 - Graph showing the percentage of SSA **10** anion coordinated to PG nanodiscs, with respect to increasing nanodisc concentration. Data was then fitted to the Hill model with V_{max} fixed at 100 %.

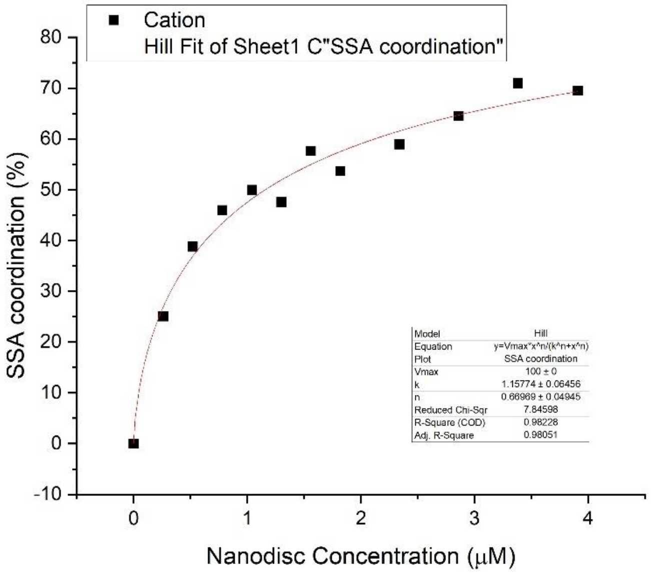

Figure S194 - Graph showing the percentage of SSA **10** cation coordinated to PG nanodiscs, with respect to increasing nanodisc concentration. Data was then fitted to the Hill model with V_{max} fixed at 100 %.

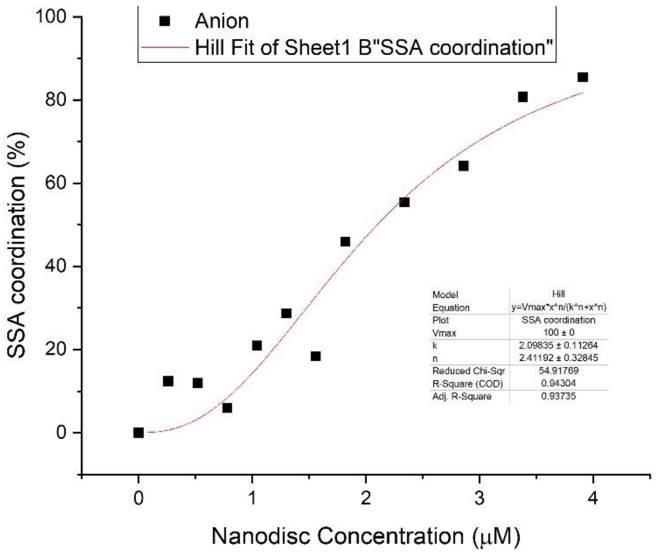

Figure S195 - Graph showing the percentage of SSA **11** anion coordinated to PG nanodiscs, with respect to increasing nanodisc concentration. Data was then fitted to the Hill model with V_{max} fixed at 100 %.

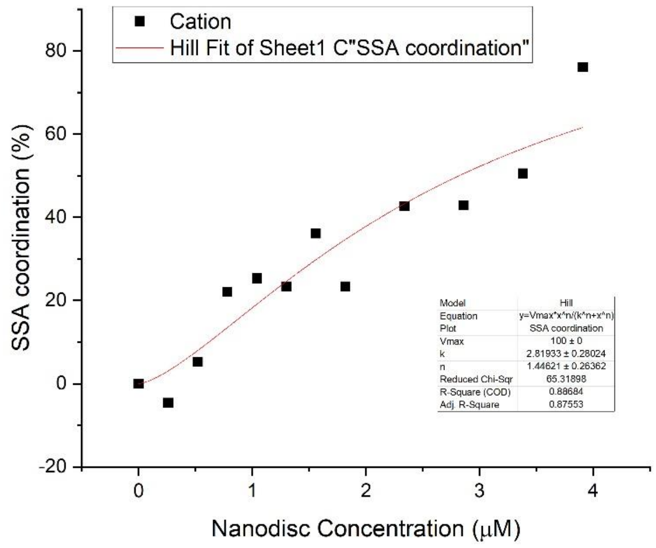

Figure S196 - Graph showing the percentage of SSA **11** cation coordinated to PG nanodiscs, with respect to increasing nanodisc concentration. Data was then fitted to the Hill model with V_{max} fixed at 100 %.

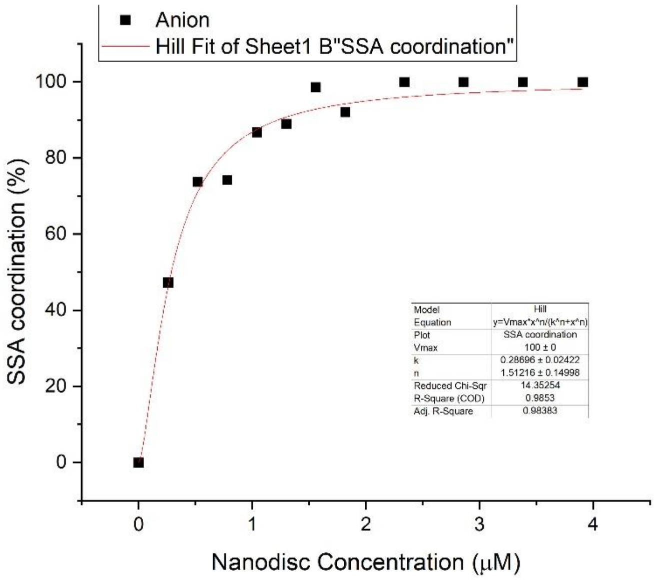

Figure S197 - Graph showing the percentage of SSA **12** anion coordinated to PG nanodiscs, with respect to increasing nanodisc concentration. Data was then fitted to the Hill model with V_{max} fixed at 100 %.

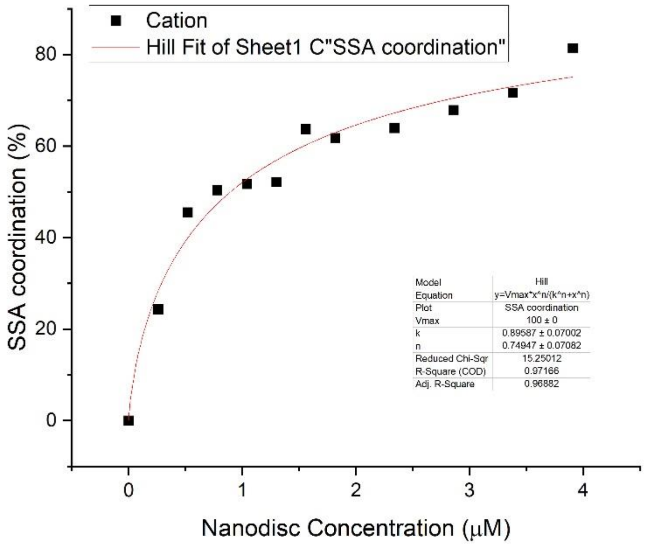

Figure S198 - Graph showing the percentage of SSA **12** cation coordinated to PG nanodiscs, with respect to increasing nanodisc concentration. Data was then fitted to the Hill model with V_{max} fixed at 100 %.

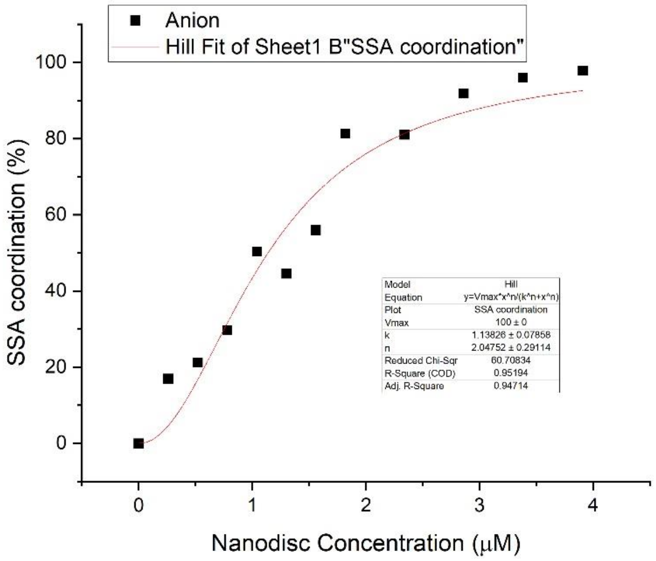

Figure S199 - Graph showing the percentage of SSA **5** anion coordinated to PE:PG 3:1 mix nanodiscs, with respect to increasing nanodisc concentration. Data was then fitted to the Hill model with V_{max} fixed at 100 %.

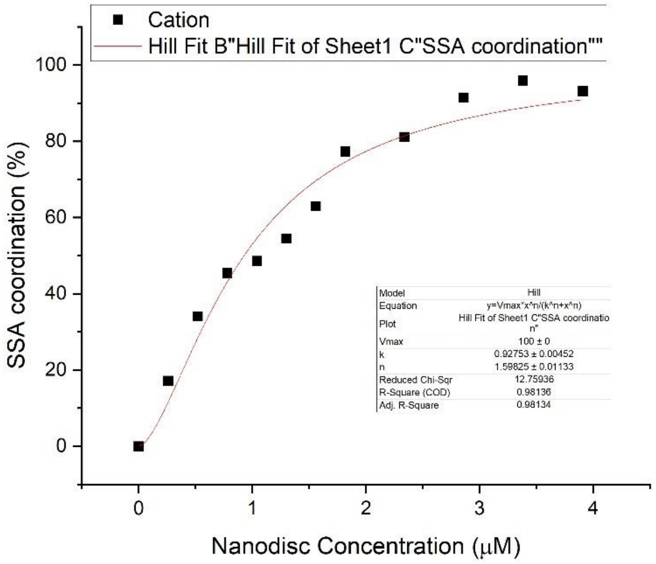

Figure S200 - Graph showing the percentage of SSA **5** cation coordinated to PE:PG 3:1 mix nanodiscs, with respect to increasing nanodisc concentration. Data was then fitted to the Hill model with V_{max} fixed at 100 %.

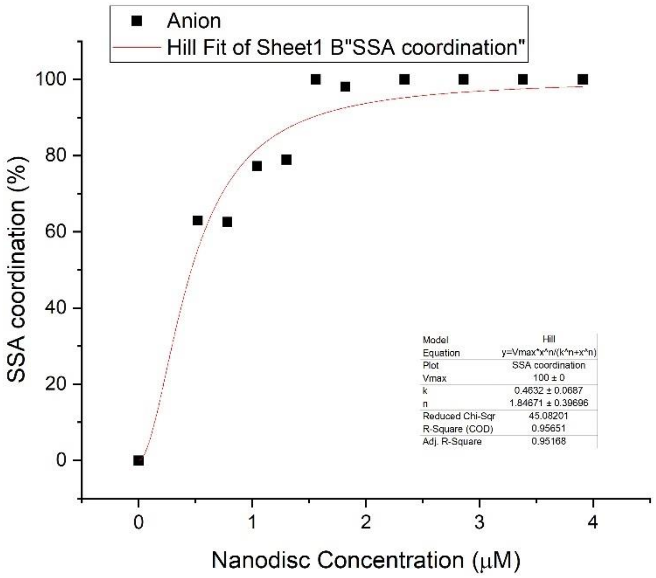

Figure S201 - Graph showing the percentage of SSA **6** anion coordinated to PE:PG 3:1 mix nanodiscs, with respect to increasing nanodisc concentration. Data was then fitted to the Hill model with V_{max} fixed at 100 %.

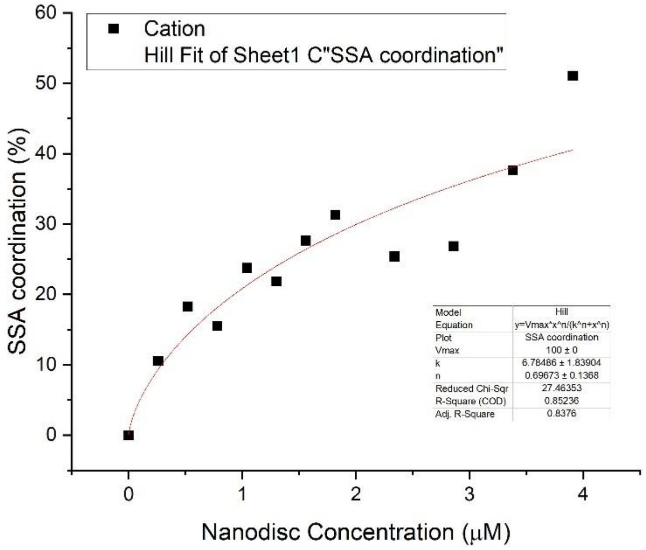

Figure S202 - Graph showing the percentage of SSA **6** cation coordinated to PE:PG 3:1 mix nanodiscs, with respect to increasing nanodisc concentration. Data was then fitted to the Hill model with V_{max} fixed at 100 %.

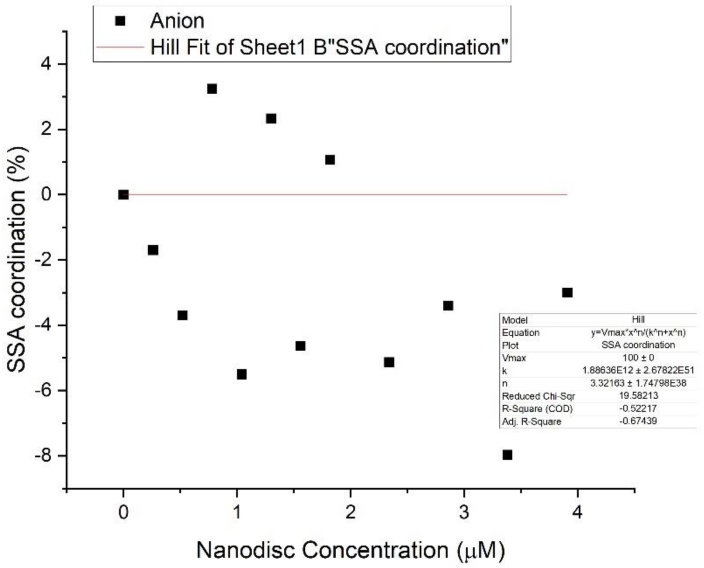

Figure S203 - Graph showing the percentage of SSA **7** anion coordinated to PE:PG 3:1 mix nanodiscs, with respect to increasing nanodisc concentration. Data was then fitted to the Hill model with V_{max} fixed at 100 %.

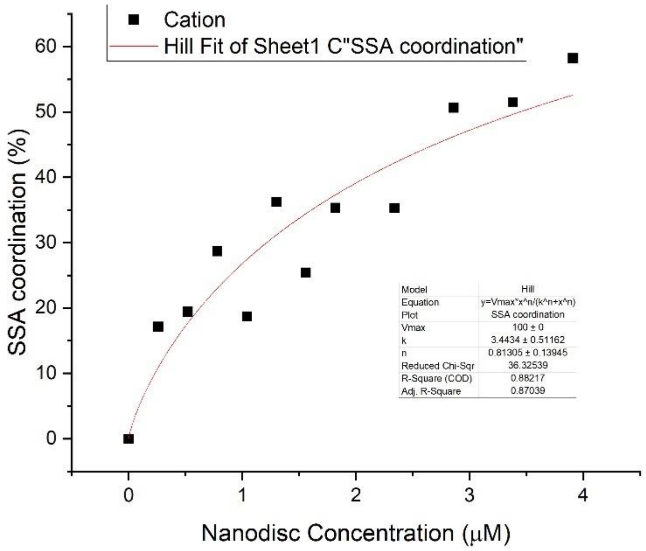

Figure S204 - Graph showing the percentage of SSA **7** cation coordinated to PE:PG 3:1 mix nanodiscs, with respect to increasing nanodisc concentration. Data was then fitted to the Hill model with V_{max} fixed at 100 %.

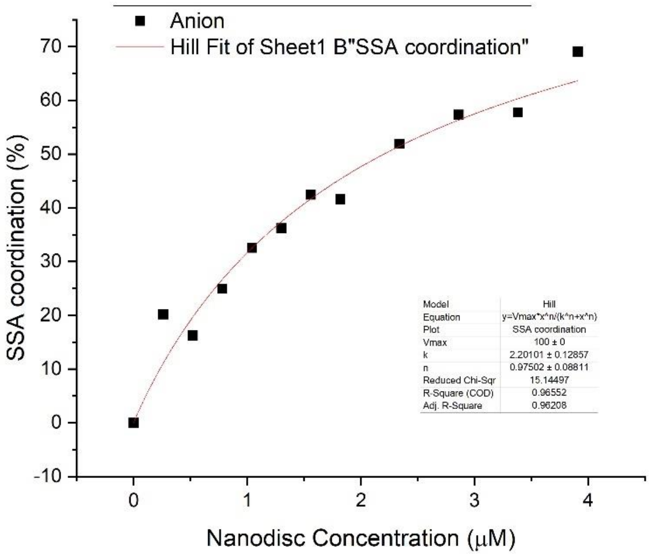

Figure S205 - Graph showing the percentage of SSA **8** anion coordinated to PE:PG 3:1 mix nanodiscs, with respect to increasing nanodisc concentration. Data was then fitted to the Hill model with V_{max} fixed at 100 %.

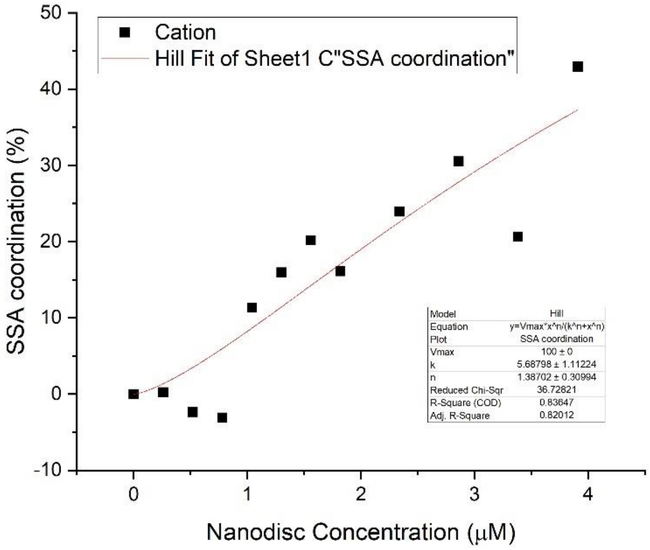

Figure S206 - Graph showing the percentage of SSA **8** cation coordinated to PE:PG 3:1 mix nanodiscs, with respect to increasing nanodisc concentration. Data was then fitted to the Hill model with V_{max} fixed at 100 %.

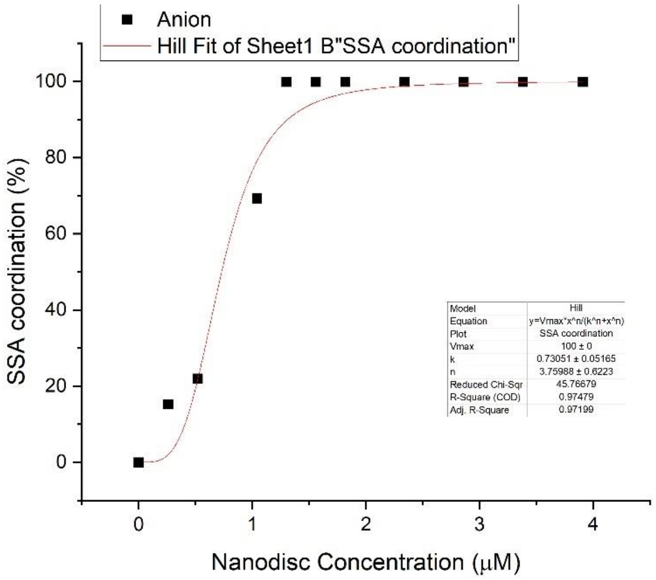

Figure S207 - Graph showing the percentage of SSA **9** anion coordinated to PE:PG 3:1 mix nanodiscs, with respect to increasing nanodisc concentration. Data was then fitted to the Hill model with V_{max} fixed at 100 %.

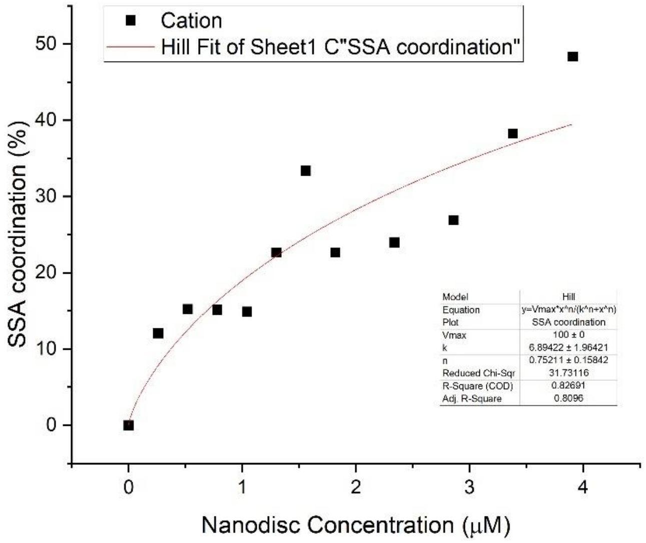

Figure S208 - Graph showing the percentage of SSA **9** cation coordinated to PE:PG 3:1 mix nanodiscs, with respect to increasing nanodisc concentration. Data was then fitted to the Hill model with V_{max} fixed at 100 %.

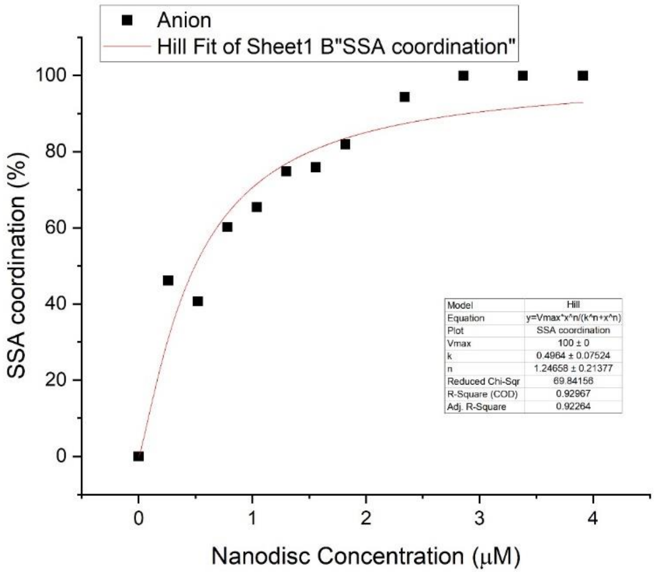

Figure S209 - Graph showing the percentage of SSA **10** anion coordinated to PE:PG 3:1 mix nanodiscs, with respect to increasing nanodisc concentration. Data was then fitted to the Hill model with V_{max} fixed at 100 %.

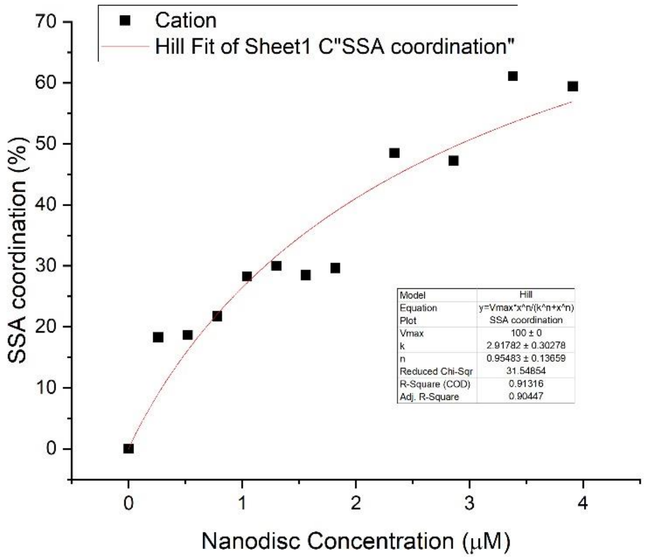

Figure S210 - Graph showing the percentage of SSA **10** cation coordinated to PE:PG 3:1 mix nanodiscs, with respect to increasing nanodisc concentration. Data was then fitted to the Hill model with V_{max} fixed at 100 %.

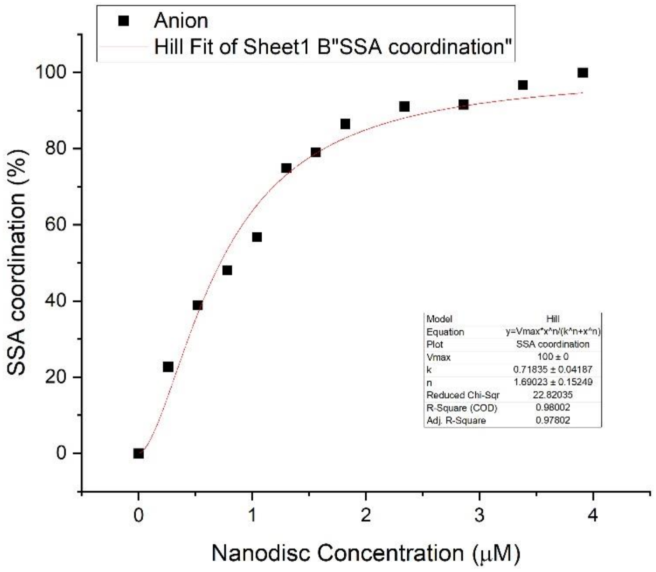

Figure S211 - Graph showing the percentage of SSA **11** anion coordinated to PE:PG 3:1 mix nanodiscs, with respect to increasing nanodisc concentration. Data was then fitted to the Hill model with V_{max} fixed at 100 %.

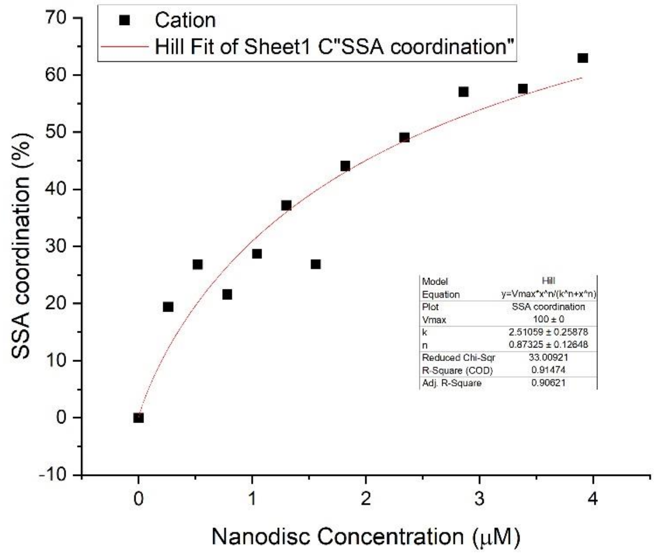

Figure S212 - Graph showing the percentage of SSA **11** cation coordinated to PE:PG 3:1 mix nanodiscs, with respect to increasing nanodisc concentration. Data was then fitted to the Hill model with V_{max} fixed at 100 %.

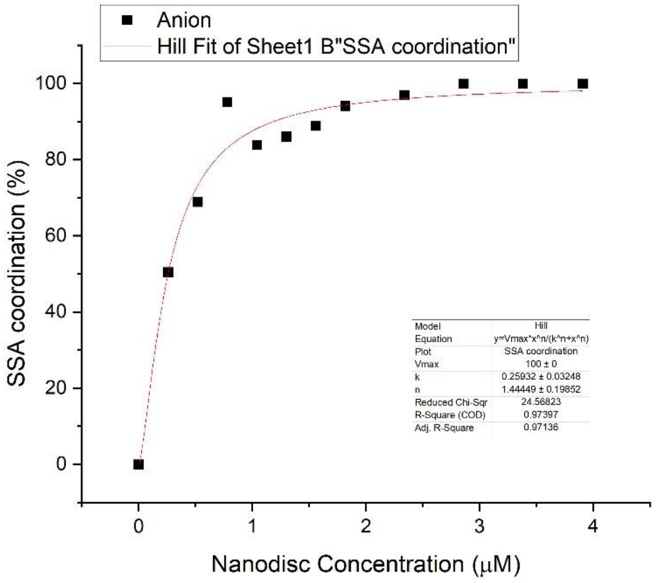

Figure S213 - Graph showing the percentage of SSA **12** anion coordinated to PE:PG 3:1 mix nanodiscs, with respect to increasing nanodisc concentration. Data was then fitted to the Hill model with V_{max} fixed at 100 %.

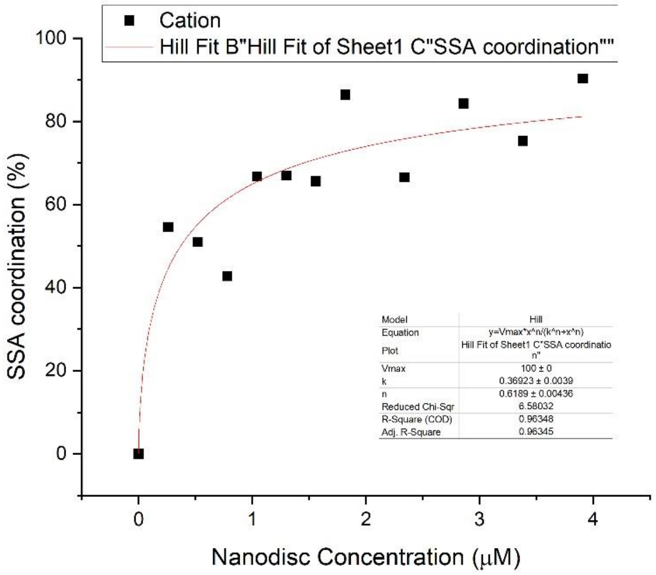

Figure S214 - Graph showing the percentage of SSA **11** cation coordinated to PE:PG 3:1 mix nanodiscs, with respect to increasing nanodisc concentration. Data was then fitted to the Hill model with V_{max} fixed at 100 %.

Table S5 - R^2 values obtained form the fitting of nanodisc titration data to either Michaelis-Menten (M. M.) or Hill Plot (H. P.) kinetics using Origin 2018 software, with V_{max} fixed to 100 % of the SSA component bound to the nanodisc. Here A = represents the fit of the anionic component of the SSA to the relevant model and C = represents the fit of the anionic component of the SSA to the relevant model.

SSA	PC		PG		PE:PG mix	
	M. M.	H. P.	M. M.	H. P.	M. M.	H. P.
**5 A**	0.31	0.66	0.21	0.67	0.85	0.95
**5 C**	0.45	0.45	0.97	0.97	0.92	0.98
**6 A**	0.94	0.99	0.93	0.99	0.92	0.96
**6 C**	0.73	0.81	0.97	0.97	0.80	0.85
**7 A**	0.15	0.47	a	0.00	a	a
**7 C**	0.57	0.60	0.83	0.97	0.87	0.88
**8 A**	0.88	0.88	0.56	0.75	0.97	0.97
**8 C**	0.35	0.35	0.88	0.91	0.80	0.84
**9 A**	0.97	0.99	0.74	0.97	0.79	0.97
**9 C**	0.53	0.99	0.91	0.94	0.79	0.83
**10 A**	0.92	0.97	0.94	0.97	0.92	0.93
**10 C**	0.77	1.00	0.91	0.98	0.91	0.91
**11 A**	0.98	0.98	0.78	0.94	0.92	0.98
**11 C**	0.35	0.66	0.84	0.87	0.91	0.91
**12 A**	0.99	1.00	0.96	0.99	0.96	0.97
**12 C**	0.61	0.94	0.94	0.97	0.81	0.96

a - No fit could be obtained for this data set.

Section 15: Computational data

Binding energy calculations using ab initio and DFT modelling

As the headgroup of a phospholipid is expected to be the main portion interacting with the SSAs, modelling of phospholipids was performed on simplified structures truncated to only include the headgroup of the phospholipid to minimise computational resource demand. The structures of the simplified model lipid headgroups is shown in Figure S216.

The lowest energy conformations of the SSAs and phospholipid headgroups were first optimised individually at the HF/3-21G level using Gaussian09 with a polarisable continuum model (PCM) solvent model for water. A range of SSA-headgroup interaction conformations were then optimised using the same computational parameters. The binding energy for each SSA-headgroup conformation was then calculated using Equation S2 – the energies were obtained from the output file and converted from Hartree to kJ/mol. The data shown in the main paper (Figure 3) represents the lowest energy binding conformations identified using these means. All calculated binding energies are shown in Figures S217–222. Cartesian coordinates for the minimised binding conformations are given in Tables S7–50 and 3D visualisations of the structures are shown in Figures S224-267.

Discussion relating to the choice of supporting molecular modelling studies

The results of our experimental nanodisc binding assay imply that the binding of the anionic and cationic components of the SSAs are not necessarily intrinsically linked. While both SSA components do bind to the nanodiscs, we often observed much higher EC_{50} values for the binding of the cationic component compared to the anionic component (see the data in Section 13, Table S5). This tells us that for a particular concentration of SSA, we would not necessarily expect the same proportion of the anionic and cationic components to be bound to a lipid bilayer, so estimating the stoichiometry that should ideally be studied is not straightforward.

The overall model we have selected simplifies the problem of SSA-bilayer interactions to the interactions between a single SSA and a single lipid headgroup. The simplified model already abstracts the whole of the membrane to a continuum solvent model in order to reduce the conformational space to one which can be reasonably explored by hand. Adding in a membrane (which would be necessary to limit the potential anion location relative to the SSA) would be prohibitively computationally expensive even at this low level. Likewise, the range of potential cation/anion/ lipid headgroup orientations is prohibitively large to sample manually.

SSA-headgroup binding energy = SSA-headgroup energy – (SSA energy + headgroup energy)

Equation S2 - Calculation of SSA-headgroup binding energy.

We considered more accurate, computationally demanding approaches, like density functional theory (DFT) and increased basis set size, for calculating binding energies but when using both ab initio (HF/3-21G) and DFT (M06-2X/6-31G) methods for the same conformations, we found that the correlation between these two methods is high (Figure S215). The M06-2X functional was chosen it includes a dispersion consideration and has been shown to be effective at modelling hydrogen bonding to anions.[12] As these are indicative calculations, neglecting both anion-cation interactions of the SSA and explicit solvation and the rest of the bilayer, only trends and differences rather than absolute energies are of interest. Given the strong correlation seen, we have opted to use the

results from the simplest, lowest cost calculations to facilitate rapid screening of the possible interaction geometries.

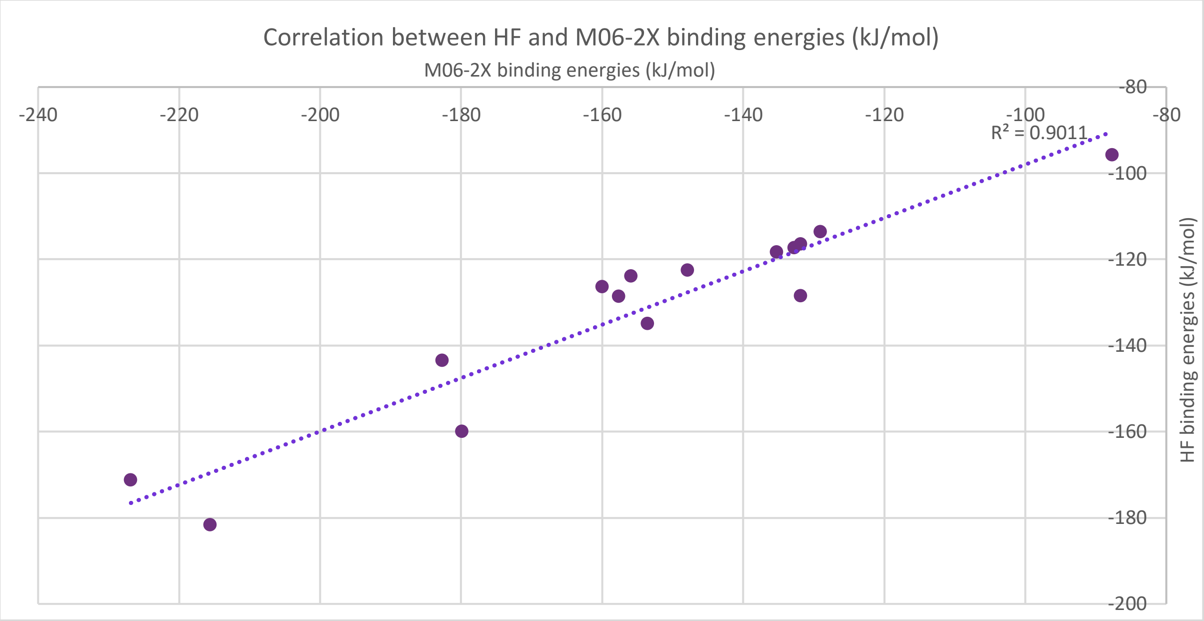

Figure S215 - Correlation data between ab initio and DFT binding energies (kJ/mol).

For SSA 4-headgroup conformations, binding energies were also calculated at the M06-2X/6-31G level by determining the individual SSA, headgroup and SSA-headgroup energies and using equation S1 again.

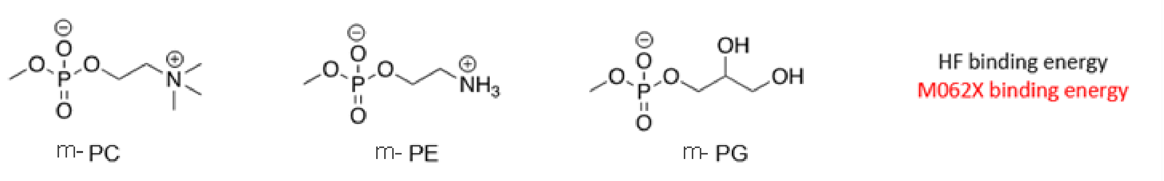

Figure S216 - The structure of the simplified model lipid headgroups (m-lipid) studied, and a key to the binding energies shown in Figures S217-222.

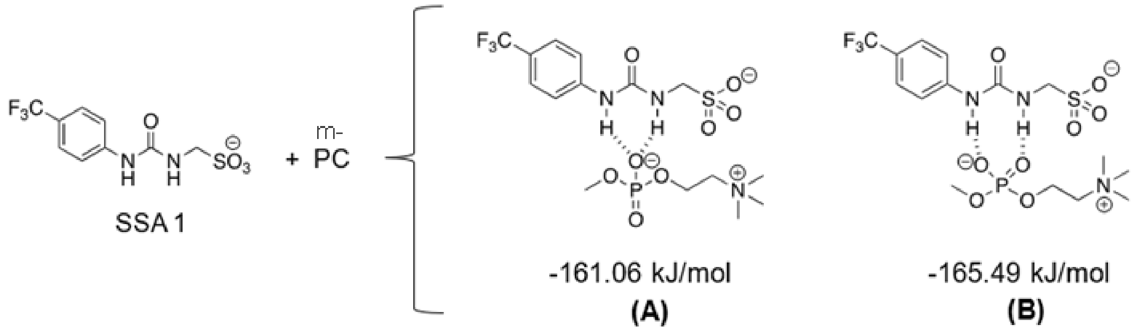

Figure S217 - Calculated binding energies for SSA **1**-PC conformations.

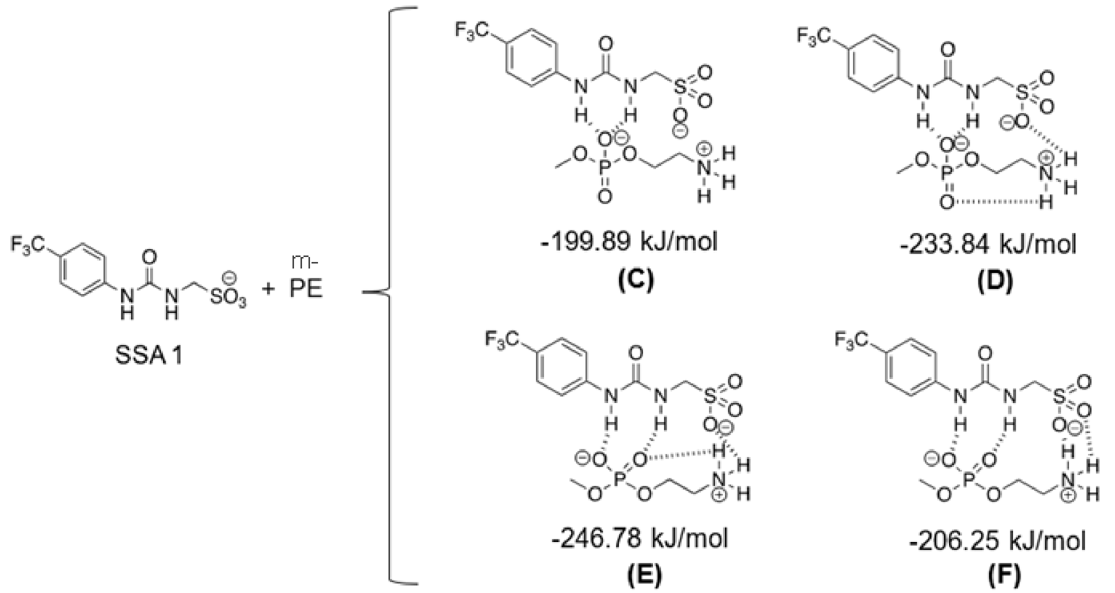

Figure S218 - Calculated binding energies for SSA **1**-PE conformations.

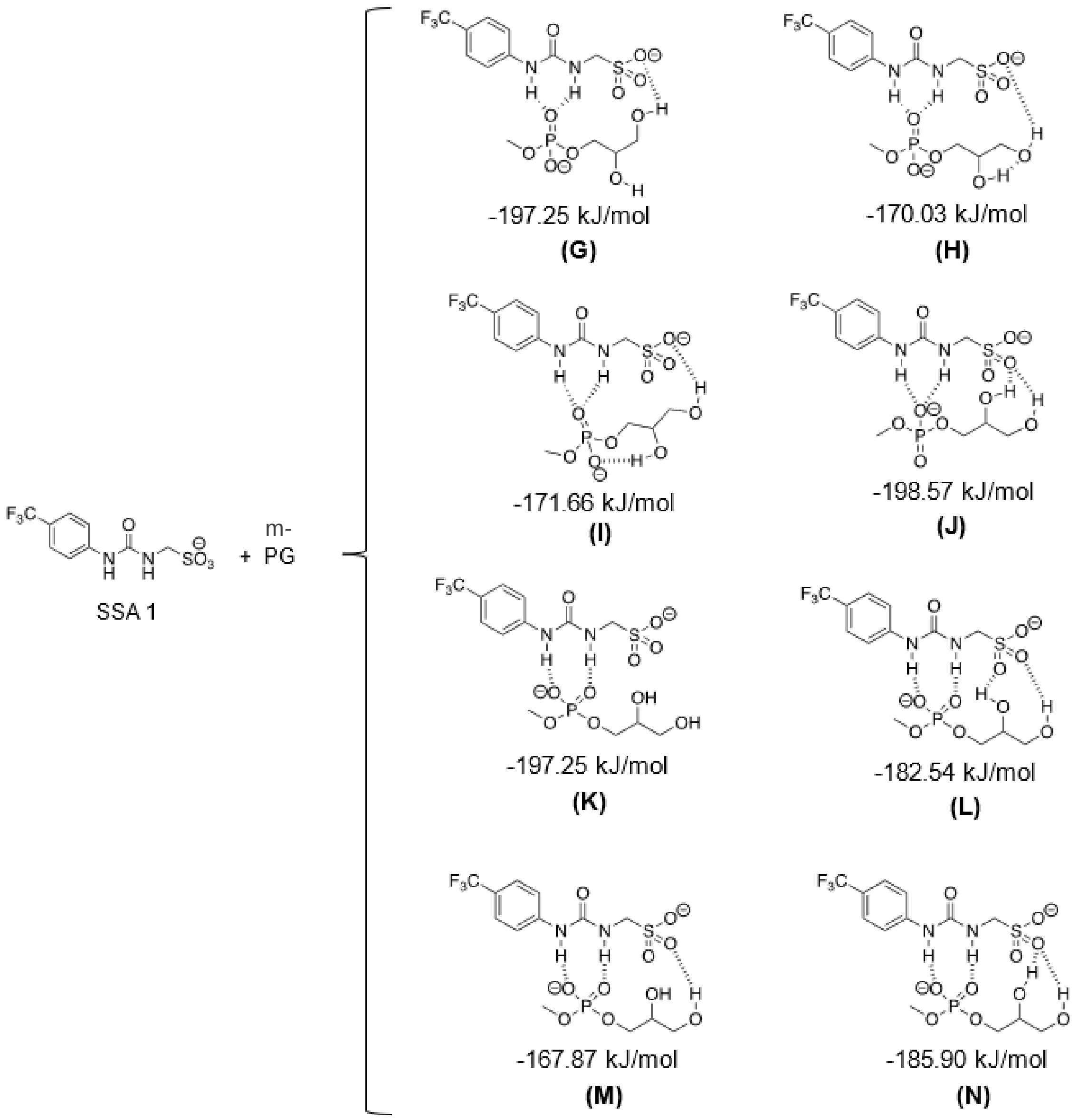

Figure S219 - Calculated binding energies for SSA **1**-PG conformations.

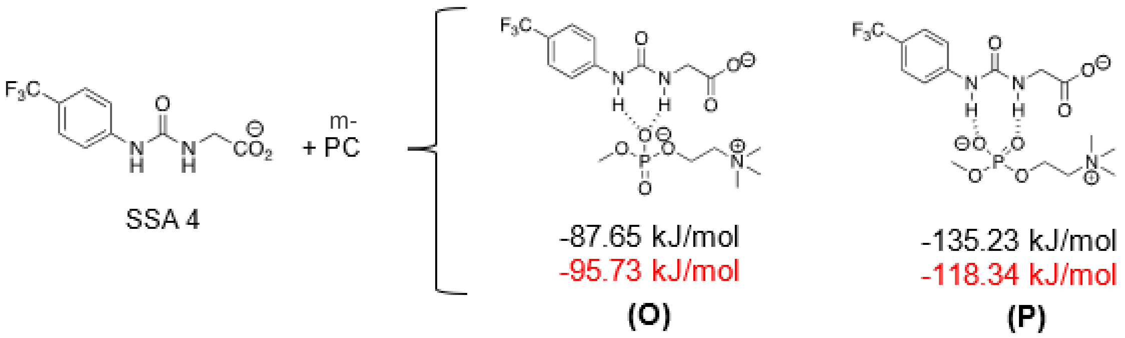

Figure S220 - Calculated binding energies for SSA **4**-PC conformations.

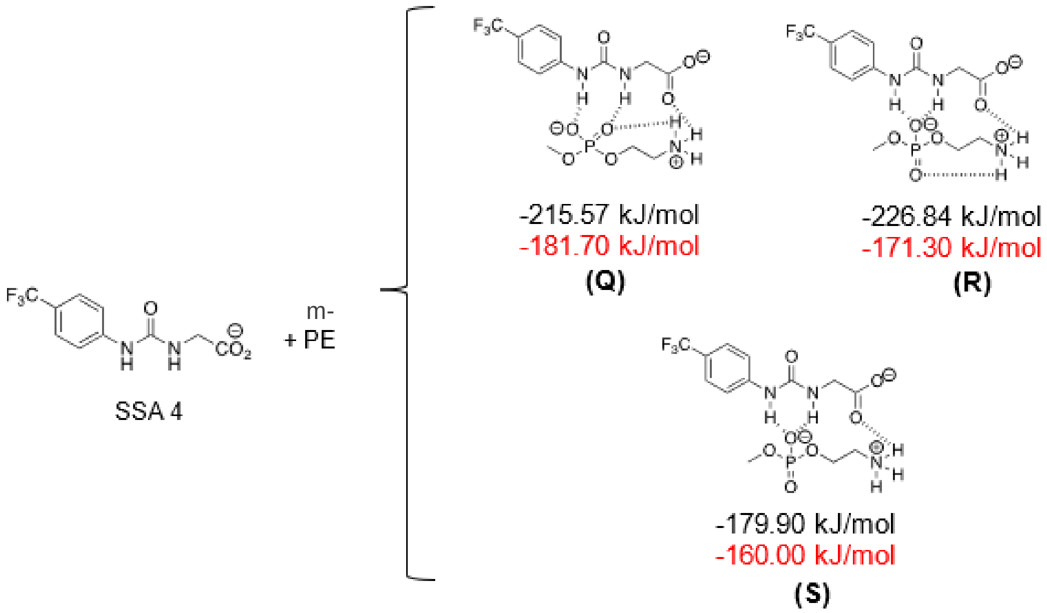

Figure S221 - Calculated binding energies for SSA **4**-PE conformations.

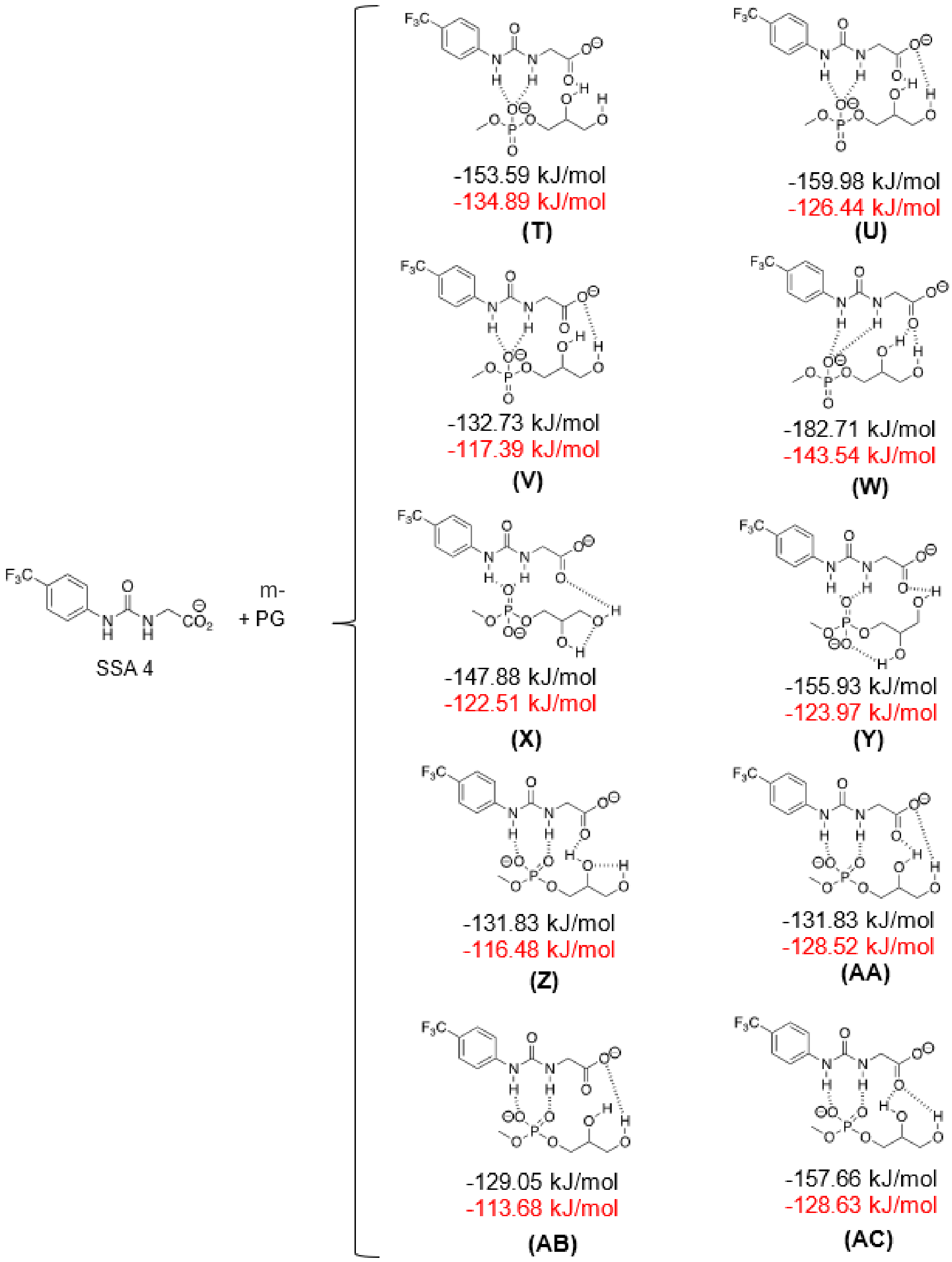

Figure S222 - Calculated binding energies for SSA **4**-PG conformations.

Solvent model comparison

Another two sets of calculations were run on conformation **T** (Figure S222) to compare the effect of using different PCM solvent models. SSA **1**, the model PG lipid headgroup and structure **T** were optimised in the gas phase at the HF/3-21G level using Gaussian09, as well as with a PCM solvent model for *n*-hexane. Binding energies (kJ/mol) were subsequently calculated using equation 1 and are shown in Table S6. As expected, calculated binding energies were significantly larger in magnitude in hexane and the gas phase by comparison to water. This is typical of hydrogen bonded complex formation and supports the choice of computational method.

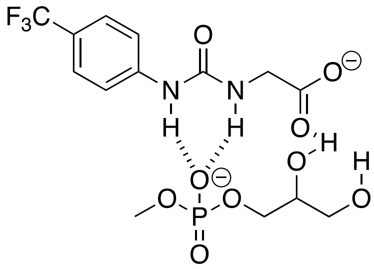

Figure S223 - Conformation **T**.

Table S6 - Conformation **T** binding energy in gas phase, PCM model for water and PCM model for n-hexane.

	Structure **T** binding energy (kJ/mol)
Water	-161
n-Hexane	-305819
Gas phase	-305761

Cartesian coordinates calculated at HF/3-21G level.

Table S7 - Cartesian coordinates (given in Angstroms) generated for structure **A** (Figure S217), calculated at HF/3-21G level, after energy minimisation calculations.

Atom		X	Y	Z
1	O	-3.3602690	1.7078880	0.1401930
2	S	-2.6684830	3.1330930	0.0515130
3	C	-1.5639000	2.9919140	-1.4006180
4	N	-0.6789990	1.9008690	-1.2147100
5	C	0.6257400	2.0926540	-0.8802630
6	N	1.2537320	0.9061900	-0.6274680
7	C	2.5828170	0.6958480	-0.3055050
8	C	3.5276190	1.7120310	-0.1589370
9	C	4.8312040	1.3996750	0.1651800
10	C	5.2173380	0.0871450	0.3478650
11	C	6.6016120	-0.2315860	0.7470240
12	F	7.4876160	0.6897310	0.2977730
13	F	6.7807620	-0.2834540	2.0945650
14	F	7.0003540	-1.4408310	0.2825830
15	C	4.2881550	-0.9284590	0.1986540
16	C	2.9860080	-0.6333860	-0.1247510
17	O	1.1549870	3.2044230	-0.8345150
18	O	-3.6801820	4.2836740	-0.3084310
19	O	-1.7728990	3.4406970	1.3096830
20	H	-1.0218290	3.9160410	-1.4854660
21	H	-2.2222540	2.8504110	-2.2412300
22	H	-1.0351490	0.9518140	-1.2090240
23	H	0.6415440	0.0878650	-0.6821810
24	H	3.2256590	2.7221630	-0.3035800
25	H	5.5511760	2.1861880	0.2673520
26	H	4.5870370	-1.9492450	0.3251600
27	H	2.2677360	-1.4199010	-0.2498290
28	C	-5.0251540	0.1085820	2.1663120
29	N	-5.0014030	-1.0173940	1.1427700
30	C	-5.8585450	-2.1542050	1.6423660
31	C	-5.5570650	-0.4708390	-0.1596810
32	C	-3.5505550	-1.4383530	0.9621750
33	C	-3.3502510	-2.6948120	0.1063540
34	O	-1.9221230	-2.9152720	0.0849990
35	P	-0.9581040	-2.3148610	-1.1131910
36	O	-0.7960840	-0.7836530	-0.8864180
37	O	0.4503390	-3.0039600	-0.6459410
38	C	0.5632380	-4.3935440	-0.2335380
39	O	-1.4535180	-2.8139870	-2.4834070
40	H	-3.1557930	-1.6354640	1.9466440
41	H	-3.0340480	-0.5982750	0.5257700
42	H	-3.7217880	-2.5800210	-0.9010200
43	H	-3.7873260	-3.5688410	0.5551480
44	H	0.2417620	-5.0495960	-1.0304430
45	H	-0.0368610	-4.5672890	0.6446140
46	H	1.6043200	-4.5638090	-0.0196780
47	H	-5.4090810	-2.5612720	2.5345800
48	H	-5.9364310	-2.9099960	0.8787900
49	H	-6.8388830	-1.7650610	1.8661530
50	H	-4.4193440	0.9059140	1.7681870
51	H	-4.6325730	-0.2711340	3.0966400
52	H	-6.0497450	0.4220610	2.2936230
53	H	-4.9434450	0.3703310	-0.4386710
54	H	-6.5764990	-0.1633310	0.0150320
55	H	-5.5343710	-1.2472760	-0.9064710

Table S8 - Cartesian coordinates (given in Angstroms) generated for structure **B** (Figure S217), calculated at HF/3-21G level, after energy minimisation calculations.

Atom	X	Y	Z
1 O	-3.7595850	-1.5487370	0.2603580
2 S	-2.7926390	-2.7834510	0.5068000
3 C	-1.8530430	-2.4171690	2.0461490
4 N	-0.9610130	-1.3430340	1.8029950
5 C	0.3062380	-1.6056560	1.3801220
6 N	0.9561500	-0.5017620	0.9037540
7 C	2.2956140	-0.4112780	0.5435700
8 C	3.2146220	-1.4575500	0.6253290
9 C	4.5273580	-1.2525220	0.2528810
10 C	4.9473810	-0.0208750	-0.2058470
11 C	6.3406540	0.1695720	-0.6526070
12 F	7.2031700	-0.6446320	0.0023700
13 F	6.5275840	-0.0895730	-1.9749910
14 F	6.7649870	1.4436810	-0.4700100
15 C	4.0427340	1.0241070	-0.2904170
16 C	2.7338030	0.8359430	0.0794660
17 O	0.8074150	-2.7294560	1.4481760
18 O	-3.5756980	-4.1211960	0.7894930
19 O	-1.7113810	-2.9060670	-0.6405470
20 H	-1.3297730	-3.3238280	2.2899760
21 H	-2.5862320	-2.1724850	2.7947650
22 H	-1.2904500	-0.3893170	1.9783830
23 H	0.4217620	0.3525830	0.7337410
24 H	2.8872800	-2.4080890	0.9722610
25 H	5.2271580	-2.0600600	0.3287360
26 H	4.3661530	1.9853860	-0.6349060
27 H	2.0224700	1.6337870	0.0245060
28 C	-2.7581130	1.0747060	-4.3112910
29 N	-2.8551440	0.4762570	-2.9236310
30 C	-4.2121060	-0.1827160	-2.7593450
31 C	-1.7848300	-0.5920410	-2.7897600
32 C	-2.6295520	1.5943030	-1.9061940
33 C	-3.3421820	1.4095030	-0.5609580
34 O	-2.8198870	2.4846150	0.2678870
35 P	-1.5067510	2.2406200	1.2283120
36 O	-0.2692300	1.9233630	0.3353700
37 O	-1.3581560	3.7335280	1.8585020
38 C	-2.4708120	4.4993460	2.3984130
39 O	-1.8569590	1.1800130	2.3074540
40 H	-2.9729780	2.5122240	-2.3542130
41 H	-1.5710450	1.6556860	-1.7181900
42 H	-3.1713970	0.4459500	-0.1089340
43 H	-4.4012760	1.5810190	-0.6417560
44 H	-2.9157230	3.9764550	3.2332240
45 H	-3.2127350	4.6625320	1.6333550
46 H	-2.0591520	5.4368460	2.7311060
47 H	-4.9758740	0.5769880	-2.8121340
48 H	-4.2291580	-0.7026690	-1.8137660
49 H	-4.3373260	-0.8836070	-3.5700580
50 H	-1.7974010	1.5548270	-4.4117110
51 H	-3.5526520	1.7930780	-4.4341480
52 H	-2.8561970	0.2809600	-5.0339970
53 H	-0.8212650	-0.1133020	-2.8664960
54 H	-1.9140450	-1.2880350	-3.6049940
55 H	-1.8870340	-1.1210370	-1.8569730

Table S9 - Cartesian coordinates (given in Angstroms) generated for structure **C** (Figure S218), calculated at HF/3-21G level, after energy minimisation calculations.

Atom		X	Y	Z
1	O	2.0500320	-3.5509280	1.1871810
2	S	3.0075620	-2.8846540	0.1311920
3	C	2.0287270	-2.4682110	-1.3617700
4	N	1.1043410	-1.4375750	-1.0676140
5	C	-0.2174200	-1.6907020	-0.8783140
6	N	-0.9040980	-0.5509290	-0.5589110
7	C	-2.2649740	-0.4194240	-0.3355440
8	C	-3.1787990	-1.4723360	-0.3995110
9	C	-4.5174150	-1.2383430	-0.1661320
10	C	-4.9714970	0.0308110	0.1321140
11	C	-6.3991650	0.2579980	0.4292610
12	F	-7.2015770	-0.6126780	-0.2290210
13	F	-6.7088940	0.1163060	1.7458540
14	F	-6.7949620	1.5101980	0.0956430
15	C	-4.0747750	1.0824910	0.1947350
16	C	-2.7383200	0.8621770	-0.0357720
17	O	-0.7150470	-2.8096880	-1.0007420
18	O	3.5423870	-1.4860190	0.6432960
19	O	4.1572750	-3.8272490	-0.3806910
20	H	1.5253500	-3.3669170	-1.6694750
21	H	2.7592970	-2.1638050	-2.0921450
22	H	1.4191110	-0.4833970	-0.9490600
23	H	-0.3345390	0.2904570	-0.4932810
24	H	-2.8266130	-2.4485710	-0.6333910
25	H	-5.2115260	-2.0518410	-0.2274760
26	H	-4.4224230	2.0715710	0.4127460
27	H	-2.0455220	1.6780520	0.0080050
28	H	5.0290430	0.5509450	2.6292560
29	H	5.9897650	0.6526220	0.4107830
30	C	4.6094050	1.2185030	1.8938940
31	H	5.0303070	2.2042350	2.0040660
32	N	4.9853400	0.6864180	0.5249770
33	H	4.5765640	-0.2716150	0.4296910
34	C	3.0906700	1.2629550	2.0533040
35	H	4.5511600	1.3050140	-0.2183960
36	H	2.8489450	1.4423740	3.0870050
37	H	2.6660380	0.3262750	1.7289370
38	O	2.5139770	2.3763000	1.3147500
39	O	3.5376320	2.2007920	-1.0552640
40	P	2.1863560	2.3383260	-0.2898770
41	O	1.1017950	1.2658010	-0.5828980
42	O	1.5951260	3.8436270	-0.4616830
43	H	2.8927760	4.5055620	-1.9584040
44	C	1.8807030	4.6732340	-1.6259070
45	H	1.7487800	5.6950420	-1.3141700
46	H	1.1887010	4.4377300	-2.4207970

Table S10 - Cartesian coordinates (given in Angstroms) generated for structure **D** (Figure S218), calculated at HF/3-21G level, after energy minimisation calculations.

Atom		X	Y	Z
1	O	2.5760190	-2.2585860	1.3095420
2	S	3.6775460	-2.2505030	0.1665310
3	C	2.7822360	-2.2724660	-1.4484460
4	N	1.7222230	-1.3429300	-1.3933680
5	C	0.4732100	-1.7444660	-1.0257610
6	N	-0.3505520	-0.6772100	-0.8017100
7	C	-1.7060410	-0.7005810	-0.5047280
8	C	-2.4643570	-1.8670240	-0.4019780
9	C	-3.8109400	-1.7899300	-0.1136920
10	C	-4.4221530	-0.5674030	0.0772840
11	C	-5.8517540	-0.5007050	0.4375700
12	F	-6.5555240	-1.5404350	-0.0715730
13	F	-6.0752180	-0.5308310	1.7789190
14	F	-6.4351480	0.6406120	-0.0019280
15	C	-3.6775390	0.5947530	-0.0238110
16	C	-2.3350390	0.5338560	-0.3111070
17	O	0.1466740	-2.9270570	-0.9371510
18	O	4.4618060	-0.8592770	0.1904960
19	O	4.6401420	-3.4950890	0.1891930
20	H	2.4190940	-3.2753800	-1.5786980
21	H	3.5218910	-2.0186490	-2.1875290
22	H	1.8975220	-0.3437360	-1.4432570
23	H	0.1106000	0.2269860	-0.8567680
24	H	-1.9899780	-2.8079960	-0.5483130
25	H	-4.3869650	-2.6905200	-0.0466210
26	H	-4.1495310	1.5464290	0.1127960
27	H	-1.7546820	1.4312150	-0.3894720
28	H	2.9777860	-0.8298280	2.5113380
29	N	3.4482370	0.0631260	2.3477810
30	H	3.9713260	0.3568030	3.1620440
31	H	4.0748580	-0.1732160	1.5430570
32	C	2.4363550	1.0928120	1.8846350
33	C	3.1556050	2.2021210	1.1199420
34	O	2.1622790	3.1246060	0.6194840
35	P	1.4070400	2.8173070	-0.8171790
36	O	1.5846590	1.2853500	-1.0282780
37	O	-0.1549850	3.0612400	-0.3789970
38	C	-0.5873610	4.1986990	0.4194250
39	O	1.8539210	3.7989930	-1.9151070
40	H	1.9048330	1.4738850	2.7396870
41	H	1.7741720	0.5814840	1.2103130
42	H	3.7144560	1.7578860	0.3106130
43	H	3.8128470	2.7651720	1.7603480
44	H	-0.0643110	4.2059600	1.3620350
45	H	-1.6451150	4.0763340	0.5757840
46	H	-0.3989450	5.1211670	-0.1113260

Table S11 - Cartesian coordinates (given in Angstroms) generated for structure **E** (Figure S218), calculated at HF/3-21G level, after energy minimisation calculations.

Atom	X	Y	Z
1 O	1.9687590	-3.2273150	1.2730920
2 S	3.1020920	-2.7980880	0.2695200
3 C	2.2961560	-2.2448760	-1.2841820
4 N	1.3677420	-1.2060880	-1.0200800
5 C	0.0490850	-1.5082200	-0.8456980
6 N	-0.7021030	-0.4472790	-0.4359970
7 C	-2.0825050	-0.4181620	-0.2662630
8 C	-2.9378930	-1.4914970	-0.5144400
9 C	-4.2965310	-1.3493130	-0.3192470
10 C	-4.8252680	-0.1539230	0.1222200
11 C	-6.2732780	-0.0324890	0.3802910
13 F	-6.6260710	-0.3508520	1.6548060
14 F	-6.7212230	1.2315840	0.1853840
15 C	-3.9845990	0.9182390	0.3688460
16 C	-2.6305350	0.7918460	0.1777240
17 O	-0.3954090	-2.6370170	-1.0610370
18 O	3.9003020	-1.5395580	0.8169180
19 O	4.0606530	-3.9653800	-0.1696780
20 H	1.8079010	-3.1137300	-1.6860040
21 H	3.0958800	-1.9197970	-1.9273450
22 H	1.7116760	-0.2509640	-1.0220910
23 H	-0.2307410	0.4240220	-0.1789890
24 H	-2.5284720	-2.4136280	-0.8505380
25 H	-4.9456060	-2.1773460	-0.5206870
26 H	-4.3911240	1.8519900	0.7006980
27 H	-1.9684700	1.6121380	0.3621870
28 H	5.6778580	0.9947930	1.7081000
29 H	5.6949580	0.6971690	-0.7477260
30 C	4.9673160	1.4903490	1.0661080
31 H	5.3212990	2.4758680	0.8129210
32 N	4.8462230	0.6703260	-0.2000860
33 H	4.6012760	-0.3134330	0.0766370
34 C	3.6120750	1.5789100	1.7630270
35 H	4.0172090	1.0124330	-0.7496450
36 H	3.7546250	1.8894670	2.7835630
37 H	3.1316570	0.6123800	1.7404850
38 O	2.7794660	2.6093650	1.1599510
39 O	2.4910660	1.3804930	-1.0771140
40 P	1.7741780	2.3832260	-0.1081980
41 O	0.3443500	1.9651870	0.3278800
42 O	1.7732770	3.8948420	-0.7037450
43 H	0.6236580	4.2106440	-2.4158950
44 C	0.6266710	4.4957490	-1.3745240
45 H	0.7442620	5.5617090	-1.2831220
46 H	-0.2879290	4.1739090	-0.9022260

Table S12 - Cartesian coordinates (given in Angstroms) generated for structure **F** (Figure S218), calculated at HF/3-21G level, after energy minimisation calculations.

Atom	X	Y	Z
1 O	-3.4772110	-4.0376160	-0.5359890
2 S	-3.1546650	-2.5254670	-0.2332080
3 C	-2.3690820	-1.8145880	-1.7773380
4 N	-1.4351410	-0.8075460	-1.4542530
5 C	-0.1552070	-1.1865990	-1.1622740
6 N	0.6468930	-0.1723700	-0.7306000
7 C	2.0077890	-0.2547490	-0.4365430
8 C	2.7899410	-1.3963050	-0.6048270
9 C	4.1347380	-1.3608680	-0.2961090
10 C	4.7187410	-0.2062260	0.1817780
11 C	6.1682220	-0.1674040	0.4577520
12 F	6.6492520	-1.3775370	0.8316360
13 F	6.9148680	0.2062080	-0.6159530
14 F	6.4766700	0.7145990	1.4390610
15 C	3.9474980	0.9293670	0.3612560
16 C	2.6080340	0.9095720	0.0578910
17 O	0.2143860	-2.3534670	-1.3018010
18 O	-4.4719090	-1.6610570	0.0479640
19 O	-2.1348840	-2.3147750	0.9738840
20 H	-3.1978890	-1.4353890	-2.3490190
21 H	-1.9077910	-2.6688570	-2.2377350
22 H	-1.7393440	0.1717220	-1.4619850
23 H	0.2507660	0.7467890	-0.5439060
24 H	2.3364100	-2.2891120	-0.9621630
25 H	4.7257950	-2.2453830	-0.4214600
26 H	4.3930850	1.8233490	0.7476990
27 H	1.9942560	1.7746320	0.2010880
28 H	-3.0881660	-1.5743710	2.4021690
29 N	-3.9242540	-0.9979190	2.5836020
30 H	-4.4941930	-1.2207110	1.7413080
31 H	-4.3869870	-1.2789600	3.4374100
32 C	-3.5536130	0.4755410	2.5841340
33 C	-2.9696340	0.7655600	1.2137580
34 O	-2.5030160	2.1384970	1.2114750
35 P	-1.6916730	2.5908610	-0.1527870
36 O	-2.3061230	1.7967200	-1.3432110
37 O	-2.1234080	4.1605090	-0.2234150
38 C	-2.3740340	4.8343920	-1.4912960
39 O	-0.1599280	2.4106790	0.0472170
40 H	-2.8272530	0.6428750	3.3610580
41 H	-4.4453270	1.0497700	2.7704210
42 H	-3.7048690	0.6203140	0.4455520
43 H	-2.1498460	0.0901330	1.0352650
44 H	-3.0206870	5.6677210	-1.2761580
45 H	-2.8486070	4.1542430	-2.1806530
46 H	-1.4428440	5.1891500	-1.9077720

Table S13 - Cartesian coordinates (given in Angstroms) generated for structure **G** (Figure S219), calculated at HF/3-21G level, after energy minimisation calculations.

Atom	X	Y	Z
1 O	4.2061910	-1.2960630	-0.2561230
2 S	3.1520740	-2.4766460	-0.1829840
3 C	2.1839990	-2.3767500	-1.7552530
4 N	1.2021860	-1.3704740	-1.6337350
5 C	-0.0636050	-1.7051590	-1.2410950
6 N	-0.8138330	-0.5984070	-0.9832200
7 C	-2.1471160	-0.5347730	-0.6129680
8 C	-2.9801830	-1.6475860	-0.4971600
9 C	-4.2998810	-1.4854670	-0.1306030
10 C	-4.8097270	-0.2289000	0.1254570
11 C	-6.2082270	-0.0716650	0.5691050
12 F	-7.0095760	-1.0536230	0.0899590
13 F	-6.3567890	-0.1067490	1.9208980
14 F	-6.7374740	1.1130250	0.1777210
15 C	-3.9893270	0.8808460	0.0127220
16 C	-2.6720060	0.7374590	-0.3515480
17 O	-0.4514910	-2.8708150	-1.1652320
18 O	3.8362200	-3.8929250	-0.1322820
19 O	2.1024500	-2.2307770	0.9780260
20 H	1.7306680	-3.3420010	-1.8850960
21 H	2.9119450	-2.1675680	-2.5197570
22 H	1.4487780	-0.3855780	-1.6283850
23 H	-0.2924270	0.2783160	-1.0606370
24 H	-2.5812460	-2.6146050	-0.6936690
25 H	-4.9346810	-2.3447660	-0.0531020
26 H	-4.3845710	1.8581580	0.2029610
27 H	-2.0365710	1.5995300	-0.4305620
28 C	3.8433790	0.1568750	2.7380380
29 C	3.4339240	1.3178300	1.7922250
30 C	1.9231180	1.3502290	1.6269990
31 O	1.5593580	2.5836720	0.9484160
32 P	0.9230680	2.6994840	-0.5537070
33 O	-0.4855340	3.3383880	-0.4953430
34 O	1.9494870	3.7366540	-1.2914510
35 C	3.2025650	3.2820350	-1.8820960
36 O	1.0426080	1.3089460	-1.2528480
37 O	4.0916190	1.2376070	0.5142640
38 H	3.7410670	2.2603740	2.2196900
39 H	1.4372230	1.3594320	2.5861900
40 H	1.5914060	0.4981900	1.0570520
41 H	3.0014780	2.7113660	-2.7769660
42 H	3.7422470	2.6757210	-1.1727260
43 H	3.7589860	4.1711170	-2.1300370
44 H	4.1962930	0.5648050	3.6737710
45 H	4.6585670	-0.3773120	2.2671820
46 O	2.7549760	-0.7208360	3.0713330
47 H	2.5365870	-1.3174280	2.3165700
48 H	4.0625810	0.3139600	0.1693260

Table S14 - Cartesian coordinates (given in Angstroms) generated for structure **H** (Figure S219), calculated at HF/3-21G level, after energy minimisation calculations.

Atom	X	Y	Z
1 O	-3.7282970	4.0108590	-0.1439650
2 S	-3.0604940	2.5846440	-0.1843250
3 C	-2.1103290	2.4881430	-1.7663270
4 N	-1.1361490	1.4677550	-1.6719970
5 C	0.1217360	1.7749740	-1.2378080
6 N	0.8648670	0.6545420	-1.0138780
7 C	2.1808900	0.5761370	-0.5846860
8 C	3.0215760	1.6797370	-0.4385580
9 C	4.3240180	1.5039350	-0.0203140
10 C	4.8094280	0.2425300	0.2584510
11 C	6.1882280	0.0710370	0.7556080
12 F	7.0195560	1.0388760	0.2994230
13 F	6.2863940	0.1152400	2.1116530
14 F	6.7160330	-1.1233960	0.3931960
15 C	3.9805060	-0.8575880	0.1203010
16 C	2.6796130	-0.6999220	-0.2942890
17 O	0.5133060	2.9333870	-1.0992860
18 O	-2.0299530	2.3188980	0.9855440
19 O	-4.1305800	1.4155140	-0.3033580
20 H	-1.6471750	3.4497090	-1.8889170
21 H	-2.8446410	2.2959490	-2.5288020
22 H	-1.3862120	0.4888250	-1.7405670
23 H	0.3597790	-0.2186200	-1.1747070
24 H	2.6414200	2.6509960	-0.6501350
25 H	4.9651260	2.3562970	0.0789810
26 H	4.3551530	-1.8383300	0.3331190
27 H	2.0345240	-1.5535110	-0.3797730
28 C	-3.8414540	0.0575490	2.7246070
29 C	-2.9199330	-1.1088530	2.3894650
30 C	-2.4383790	-1.0329410	0.9546820
31 O	-1.7518040	-2.3005150	0.7162360
32 P	-0.9423240	-2.5994770	-0.6777960
33 O	0.4265050	-3.2510940	-0.3702320
34 O	-1.9681870	-3.6565300	-1.3968180
35 C	-2.1431220	-3.6996400	-2.8392000
36 O	-0.9410760	-1.2831620	-1.5185970
37 O	-3.6360460	-2.3452490	2.5809970
38 H	-2.0588290	-1.0713980	3.0480310
39 H	-1.7785020	-0.1966800	0.8109250
40 H	-3.2770490	-0.9464490	0.2876000
41 H	-1.3148900	-4.2187220	-3.3009450
42 H	-2.2082800	-2.6981290	-3.2337540
43 H	-3.0572370	-4.2377670	-3.0253760
44 H	-3.2560010	0.9659890	2.6759270
45 H	-4.2106610	-0.0767120	3.7319900
46 O	-4.9738560	0.1291900	1.8453000
47 H	-4.7263610	0.6400310	1.0370570
48 H	-3.1430500	-3.0271520	2.0999970

Table S15 - Cartesian coordinates (given in Angstroms) generated for structure **I** (Figure S219), calculated at HF/3-21G level, after energy minimisation calculations.

Atom	X	Y	Z
1 O	-3.9974000	4.0997540	-0.0346190
2 S	-2.9183660	3.0197780	0.3467010
3 C	-2.0569580	2.6627080	-1.2321140
4 N	-1.0987480	1.6344090	-1.0653940
5 C	0.2218650	1.9156830	-0.8777440
6 N	0.9442000	0.7779420	-0.6601080
7 C	2.2987430	0.6616410	-0.4049390
8 C	3.2013070	1.7247860	-0.4372050
9 C	4.5377360	1.5013840	-0.1803000
10 C	4.9974300	0.2325800	0.1104110
11 C	6.4208140	0.0143760	0.4323630
12 F	7.2280720	0.9042160	-0.1939450
13 F	6.7034150	0.1352080	1.7574430
14 F	6.8367500	-1.2278510	0.0852190
15 C	4.1085070	-0.8281560	0.1482530
16 C	2.7741020	-0.6207510	-0.1033990
17 O	0.6819610	3.0565900	-0.9209920
18 O	-1.8488510	3.5203540	1.3875640
19 O	-3.5583140	1.6434130	0.7989410
20 H	-1.5910430	3.5831750	-1.5348390
21 H	-2.8372520	2.3820580	-1.9189290
22 H	-1.3780380	0.6623170	-1.0541050
23 H	0.3970540	-0.0862200	-0.7018020
24 H	2.8413880	2.7008270	-0.6621020
25 H	5.2255360	2.3217180	-0.2180440
26 H	4.4620860	-1.8147850	0.3686770
27 H	2.0808850	-1.4375830	-0.0585930
28 C	-5.6119090	-1.1416830	0.8624040
29 C	-4.3889550	-2.0391860	0.6536060
30 C	-3.1323910	-1.4145770	1.2577830
31 O	-1.9898780	-2.3139400	1.0971360
32 P	-1.2116460	-2.5083710	-0.3276700
33 O	-0.9121530	-1.1101130	-0.9421310
34 O	0.1926540	-3.1596440	0.2049970
35 C	0.7301360	-4.4202950	-0.2880810
36 O	-1.9737130	-3.5473810	-1.1993410
37 O	-4.2520240	-2.2421160	-0.7613310
38 H	-4.5723170	-2.9771110	1.1685980
39 H	-3.2521200	-1.2894090	2.3219290
40 H	-2.9168450	-0.4644860	0.8025440
41 H	1.1393630	-4.9470530	0.5581370
42 H	1.5102360	-4.2147100	-1.0050540
43 H	-0.0524700	-4.9984830	-0.7517190
44 H	-5.7250670	-0.9481950	1.9260750
45 H	-6.4808810	-1.6931000	0.5329810
46 O	-5.5899170	0.0656710	0.0961520
47 H	-4.8599410	0.6709270	0.3558110
48 H	-3.5758240	-2.9231710	-0.9789200

Table S16 - Cartesian coordinates (given in Angstroms) generated for structure **J** (Figure S219), calculated at HF/3-21G level, after energy minimisation calculations.

Atom		X	Y	Z
1	O	-3.5694980	-1.3640170	-0.3507670
2	S	-2.9909280	-2.8100650	-0.0363630
3	C	-1.9803090	-2.5369860	1.4709400
4	N	-1.0280540	-1.5241590	1.2193230
5	C	0.2809120	-1.8180220	0.9759700
6	N	0.9813040	-0.6919760	0.6561240
7	C	2.3290610	-0.5680900	0.3728040
8	C	3.2315980	-1.6316560	0.3690650
9	C	4.5600660	-1.4067860	0.0744940
10	C	5.0107730	-0.1354460	-0.2178570
11	C	6.4229370	0.0857400	-0.5832900
12	F	7.2485660	-0.8185310	-0.0029640
13	F	6.6604850	-0.0085670	-1.9195370
14	F	6.8551900	1.3195800	-0.2256770
15	C	4.1218880	0.9266490	-0.2125850
16	C	2.7951410	0.7205830	0.0785530
17	O	0.7456290	-2.9557170	1.0580560
18	O	-4.1179140	-3.8278400	0.3699270
19	O	-2.0472920	-3.3114550	-1.1909340
20	H	-1.5035610	-3.4712400	1.7060800
21	H	-2.6903330	-2.2591290	2.2314100
22	H	-1.3189650	-0.5557320	1.1178050
23	H	0.4064720	0.1565690	0.6280530
24	H	2.8782630	-2.6094370	0.5978460
25	H	5.2479480	-2.2278900	0.0820650
26	H	4.4717310	1.9160490	-0.4274100
27	H	2.1071230	1.5452070	0.0847660
28	C	-4.9657530	0.9746110	-2.0408760
29	C	-4.1293170	1.7729000	-1.0373140
30	C	-2.7015590	1.9061970	-1.5453950
31	O	-2.0030070	2.9264960	-0.7848850
32	P	-0.9442220	2.6317280	0.4288730
33	O	0.4313830	3.2635740	0.1071420
34	O	-1.6214470	3.4211660	1.6914140
35	C	-2.5060620	2.7464930	2.6329880
36	O	-0.9663400	1.1049960	0.7392370
37	O	-4.1555990	1.1824940	0.2791200
38	H	-4.5535220	2.7595640	-0.9298700
39	H	-2.6934300	2.2306010	-2.5731790
40	H	-2.1960560	0.9610200	-1.4526980
41	H	-1.9427640	2.0505910	3.2372070
42	H	-3.2800480	2.2233790	2.0969560
43	H	-2.9245420	3.5187180	3.2572900
44	H	-5.2160680	1.6108850	-2.8777870
45	H	-5.8856620	0.6714060	-1.5520950
46	O	-4.2477920	-0.1487170	-2.5793290
47	H	-3.9958670	-0.7532360	-1.8426910
48	H	-3.8446990	0.2534470	0.2336010

Table S17 - Cartesian coordinates (given in Angstroms) generated for structure **K** (Figure S219), calculated at HF/3-21G level, after energy minimisation calculations.

Atom	X	Y	Z
1 O	3.2117420	-1.2702600	0.4151150
2 S	2.6557860	-2.6846220	-0.0308770
3 C	1.7191220	-2.3695730	-1.5800920
4 N	0.7866930	-1.3276610	-1.3513070
5 C	-0.4951620	-1.6162200	-1.0008640
6 N	-1.1517460	-0.5454080	-0.4597800
7 C	-2.5097580	-0.4478510	-0.1944180
8 C	-3.4622110	-1.4199290	-0.5048700
9 C	-4.7930430	-1.1970190	-0.2167580
10 C	-5.1999790	-0.0216880	0.3826270
11 C	-6.6184950	0.1796100	0.7345050
12 F	-7.4520030	-0.4761540	-0.1089670
13 F	-6.9389310	-0.2631860	1.9802830
14 F	-6.9677590	1.4891100	0.7147980
15 C	-4.2628170	0.9478210	0.6973930
16 C	-2.9358800	0.7408170	0.4131370
17 O	-0.9950620	-2.7308710	-1.1686230
18 O	3.7957630	-3.6960970	-0.4206760
19 O	1.6316080	-3.2457670	1.0257370
20 H	1.2360240	-3.2943350	-1.8382910
21 H	2.4567860	-2.0944940	-2.3139900
22 H	1.1172820	-0.3644740	-1.4665000
23 H	-0.5916220	0.2473200	-0.1046200
24 H	-3.1453260	-2.3307070	-0.9531910
25 H	-5.5176340	-1.9449980	-0.4684460
26 H	-4.5747110	1.8652330	1.1538700
27 H	-2.1988280	1.4820110	0.6455550
28 C	5.3948280	0.6602960	2.1581540
29 C	4.7010300	1.4420340	1.0415000
30 C	3.2536790	1.7367090	1.3862190
31 O	2.6880230	2.5623120	0.3288400
32 P	1.3649560	2.1112910	-0.5186050
33 O	0.3098830	1.5231930	0.4670010
34 O	0.9097960	3.5874260	-1.0548690
35 C	0.4314470	3.8169730	-2.4068290
36 O	1.7132560	1.2160140	-1.7409270
37 O	4.8328450	0.6681460	-0.1659750
38 H	5.2250790	2.3710880	0.8750950
39 H	3.1933020	2.3000450	2.3051120
40 H	2.6994040	0.8161450	1.4804240
41 H	-0.5995840	3.5043950	-2.4961860
42 H	1.0333180	3.2727870	-3.1168230
43 H	0.5076350	4.8765520	-2.5844490
44 H	4.8109100	-0.2245240	2.3835530
45 H	5.5126960	1.2558470	3.0501220
46 O	6.7109180	0.2916750	1.6899420
47 H	6.5976810	0.0799630	0.7489200
48 H	4.2475260	-0.1277800	-0.0872220

Table S18 - Cartesian coordinates (given in Angstroms) generated for structure **L** (Figure S219), calculated at HF/3-21G level, after energy minimisation calculations.

Atom	X	Y	Z
1 O	4.0724620	-1.1581120	-0.5791910
2 S	2.9564960	-2.2779720	-0.6847100
3 C	1.9503840	-1.8523820	-2.1851950
4 N	1.0116800	-0.8506820	-1.8494480
5 C	-0.2331080	-1.2351880	-1.4419770
6 N	-0.9656130	-0.2326270	-0.8812360
7 C	-2.3072140	-0.2848230	-0.5191300
8 C	-3.1490670	-1.3766720	-0.7304930
9 C	-4.4738720	-1.3096120	-0.3500300
10 C	-4.9812570	-0.1723850	0.2438670
11 C	-6.3855740	-0.1335670	0.6950420
12 F	-7.1850650	-0.9309600	-0.0538850
13 F	-6.5558110	-0.5490500	1.9792030
14 F	-6.9015010	1.1188820	0.6488630
15 C	-4.1529970	0.9163120	0.4598330
16 C	-2.8331960	0.8649850	0.0845740
17 O	-0.6380730	-2.3896420	-1.5967360
18 O	3.5485570	-3.7204830	-0.9059790
19 O	1.9446630	-2.1892740	0.5412970
20 H	1.4681800	-2.7676940	-2.4733500
21 H	2.6667930	-1.5225350	-2.9171920
22 H	1.3220870	0.1260080	-1.8580260
23 H	-0.4981720	0.6389000	-0.6075840
24 H	-2.7544340	-2.2561990	-1.1795690
25 H	-5.1144550	-2.1499610	-0.5260420
26 H	-4.5443030	1.8049280	0.9122020
27 H	-2.1779480	1.6957480	0.2454930
28 C	3.5507660	-0.5269340	3.3184970
29 C	3.6844780	0.6669010	2.3620650
30 C	2.3690640	0.9394790	1.6433200
31 O	2.5724860	2.0977530	0.7828890
32 P	1.5327310	2.4261000	-0.4358070
33 O	0.0867890	2.1042380	0.0449780
34 O	1.7938710	4.0329730	-0.5558470
35 C	1.6892640	4.7511760	-1.8158240
36 O	1.9587370	1.7267420	-1.7595790
37 O	4.7651120	0.4528270	1.4522860
38 H	3.9286670	1.5444050	2.9447670
39 H	1.5922540	1.1646150	2.3585210
40 H	2.0740080	0.0827790	1.0600280
41 H	0.6599990	5.0238310	-2.0002750
42 H	2.0532760	4.1353740	-2.6232700
43 H	2.2909350	5.6392680	-1.7191800
44 H	2.6648340	-0.4040970	3.9355000
45 H	4.4101610	-0.5294020	3.9736350
46 O	3.5678820	-1.7940720	2.6470910
47 H	2.8596350	-1.8998980	1.9751560
48 H	4.5165950	-0.2240380	0.7818540

Table S19 - Cartesian coordinates (given in Angstroms) generated for structure **M** (Figure S219), calculated at HF/3-21G level, after energy minimisation calculations.

Atom	X	Y	Z
1 O	3.0556030	-4.2096070	-0.5394800
2 S	2.7543530	-2.6694740	-0.3889500
3 C	1.8222240	-2.1739460	-1.9185450
4 N	0.9423700	-1.1010850	-1.6410640
5 C	-0.3200950	-1.3953580	-1.2235170
6 N	-1.0516590	-0.3127190	-0.8305820
7 C	-2.3916310	-0.3089820	-0.4517200
8 C	-3.2324210	-1.4214070	-0.4845050
9 C	-4.5538030	-1.2983230	-0.1061720
10 C	-5.0591320	-0.0840180	0.3098300
11 C	-6.4602330	0.0222260	0.7604670
12 F	-7.2634310	-0.8809370	0.1480350
13 F	-6.6200140	-0.1926040	2.0941660
14 F	-6.9789070	1.2523950	0.5273030
15 C	-4.2318690	1.0256540	0.3480480
16 C	-2.9149410	0.9190910	-0.0266010
17 O	-0.7491780	-2.5523180	-1.2304140
18 O	1.8519920	-2.3130960	0.8602620
19 O	4.0825000	-1.7959370	-0.4197120
20 H	1.2888780	-3.0602510	-2.2080210
21 H	2.5840550	-1.9049810	-2.6291790
22 H	1.3052350	-0.1450420	-1.7097780
23 H	-0.6034520	0.6006260	-0.7458450
24 H	-2.8405510	-2.3580580	-0.7999050
25 H	-5.1934030	-2.1567020	-0.1457870
26 H	-4.6212750	1.9729090	0.6618380
27 H	-2.2613210	1.7661120	-0.0012730
28 C	4.1575150	-0.4679800	2.6850790
29 C	3.4214410	0.8323780	2.3781960
30 C	2.8714810	0.8099510	0.9721760
31 O	2.2996400	2.1399140	0.7390030
32 P	1.4472210	2.3753050	-0.6434940
33 O	-0.0795700	2.2475460	-0.3695970
34 O	1.8686860	3.9197970	-0.9664270
35 C	2.0204720	4.4023310	-2.3313620
36 O	2.0103930	1.4236220	-1.7385230
37 O	4.3321610	1.9424290	2.5159070
38 H	2.5979600	0.9364970	3.0760570
39 H	2.1231760	0.0429020	0.8743570
40 H	3.6568690	0.6353040	0.2626440
41 H	1.0544270	4.6555120	-2.7438510
42 H	2.4894200	3.6449380	-2.9393700
43 H	2.6382350	5.2828190	-2.2818670
44 H	3.4233880	-1.2643870	2.6996170
45 H	4.6098920	-0.3847530	3.6633830
46 O	5.2002610	-0.7492180	1.7412160
47 H	4.8199870	-1.1887980	0.9420130
48 H	3.9227520	2.6995630	2.0704380

Table S20 - Cartesian coordinates (given in Angstroms) generated for structure **N** (Figure S219), calculated at HF/3-21G level, after energy minimisation calculations.

Atom	X	Y	Z
1 O	3.7140740	-3.9132620	-0.7065010
2 S	2.7752820	-2.7479970	-0.2232300
3 C	1.8431180	-2.2017660	-1.7097870
4 N	0.9357240	-1.1771970	-1.3528450
5 C	-0.3540850	-1.4917160	-1.0511380
6 N	-1.0511860	-0.4459600	-0.5177710
7 C	-2.4143460	-0.4003280	-0.2529500
8 C	-3.3080620	-1.4446330	-0.4915760
9 C	-4.6482600	-1.2840210	-0.2052300
10 C	-5.1212450	-0.0992240	0.3205800
11 C	-6.5474380	0.0402450	0.6723420
12 F	-7.3444810	-0.7241930	-0.1128730
13 F	-6.8315970	-0.3301700	1.9501270
14 F	-6.9765280	1.3212250	0.5630480
15 C	-4.2419620	0.9434260	0.5610470
16 C	-2.9060370	0.7992480	0.2787250
17 O	-0.8286780	-2.6102590	-1.2628000
18 O	1.7357570	-3.1637490	0.8818910
19 O	3.6005070	-1.4664390	0.2250390
20 H	1.3391980	-3.0762670	-2.0796780
21 H	2.5903420	-1.8570840	-2.4034760
22 H	1.2692300	-0.2052100	-1.3938450
23 H	-0.5400110	0.3955100	-0.2298650
24 H	-2.9405120	-2.3590130	-0.8914930
25 H	-5.3275620	-2.0887900	-0.4014580
26 H	-4.6058860	1.8692410	0.9582780
27 H	-2.2125040	1.5952710	0.4542650
28 C	5.2822100	0.5055270	2.1998960
29 C	4.6139130	1.4199110	1.1679170
30 C	3.1534930	1.6288200	1.5377410
31 O	2.5853650	2.6402430	0.6590310
32 P	1.3864640	2.3301360	-0.4053860
33 O	0.1037340	1.8834430	0.3596100
34 O	1.2314390	3.8393440	-1.0121490
35 C	0.9198690	4.0821750	-2.4107490
36 O	1.8308870	1.3640790	-1.5400790
37 O	4.7419120	0.9066660	-0.1678400
38 H	5.1147740	2.3767410	1.1724480
39 H	3.0611280	1.9921360	2.5487380
40 H	2.6236770	0.6943700	1.4442940
41 H	-0.1481700	4.0259050	-2.5669940
42 H	1.4158620	3.3539140	-3.0323500
43 H	1.2689930	5.0749930	-2.6401780
44 H	5.4721910	1.0654880	3.1045950
45 H	6.2328460	0.1794790	1.7910810
46 O	4.4511220	-0.6072950	2.5742030
47 H	4.1525360	-1.0863340	1.7667480
48 H	4.2581190	0.0577550	-0.2412050

Table S21 - Cartesian coordinates (given in Angstroms) generated for structure **O** (Figure S220), calculated at HF/3-21G level, after energy minimisation calculations.

Atom		X	Y	Z
1	C	-7.2618430	-0.8990310	-0.4926540
2	N	-5.9626240	-1.5230690	-0.9700010
3	C	-6.2311440	-2.9680170	-1.3391780
4	C	-5.4836580	-0.7649570	-2.1909800
5	C	-4.9554100	-1.4238500	0.1655680
6	C	-3.5577690	-1.9561150	-0.1544130
7	O	-2.8644110	-1.8275300	1.1049090
8	P	-1.4322590	-1.0248000	1.2577730
9	O	-1.2540280	-0.1601030	-0.0289890
10	O	-1.8428140	-0.0754590	2.5221450
11	C	-0.9328130	0.9278460	3.0716170
12	O	-0.2880800	-1.9996600	1.6231260
13	H	-5.3598050	-1.9780280	0.9969190
14	H	-4.8836380	-0.3812050	0.4315880
15	H	-3.0559410	-1.3675130	-0.9020150
16	H	-3.5682540	-2.9961700	-0.4326770
17	H	-1.3968170	1.2869240	3.9757420
18	H	-0.7895610	1.7399680	2.3745160
19	H	0.0182560	0.4702630	3.3114300
20	H	-6.5482800	-3.4955640	-0.4538490
21	H	-5.3335240	-3.4071280	-1.7393010
22	H	-7.0098200	-2.9864790	-2.0845100
23	H	-7.0759070	0.1338200	-0.2464410
24	H	-7.6023660	-1.4380600	0.3764890
25	H	-7.9863410	-0.9711260	-1.2875630
26	H	-5.2693130	0.2515600	-1.9016990
27	H	-6.2674910	-0.7861110	-2.9308160
28	H	-4.6004280	-1.2413820	-2.5810880
29	C	4.8115930	0.2771620	-0.9666630
30	C	3.5986540	0.9221290	-1.0816690
31	C	2.4369400	0.3229760	-0.5900280
32	C	2.5360550	-0.9300830	0.0313090
33	C	3.7526410	-1.5602260	0.1357390
34	C	4.8969970	-0.9624220	-0.3649190
35	C	6.2065550	-1.6204780	-0.2001770
36	N	1.1762320	0.8815050	-0.6876110
37	C	0.8472150	2.1677410	-1.0477590
38	O	1.6727270	3.0336470	-1.3510850
39	N	-0.4899050	2.3647520	-1.0661230
40	C	-1.0085380	3.7147650	-0.9698130
41	C	-0.8497980	4.3133050	0.4499610
42	O	-0.2199310	3.6357650	1.2989740
43	O	-1.3613500	5.4464110	0.5984320
44	F	7.0858320	-1.2591840	-1.1659890
45	F	6.1059520	-2.9721960	-0.2270300
46	F	6.8197560	-1.3274970	0.9788540
47	H	5.6940660	0.7412950	-1.3583600
48	H	3.5289450	1.8823700	-1.5346930
49	H	1.6571820	-1.3810530	0.4510700
50	H	3.8143650	-2.5208420	0.6062520
51	H	0.3804150	0.3051450	-0.4189830
52	H	-1.0543950	1.6124370	-0.7051100
53	H	-2.0583450	3.7174650	-1.2305770
54	H	-0.4898890	4.3599510	-1.6650350

Table S22 - Cartesian coordinates (given in Angstroms) generated for structure **P** (Figure S220), calculated at HF/3-21G level, after energy minimisation calculations.

Atom		X	Y	Z
1	O	-2.5784940	2.3919150	0.2104090
2	C	-2.9200990	2.9764690	-0.8597980
3	C	-2.2936210	2.4876240	-2.1833500
4	N	-1.3958030	1.3639420	-2.0219090
5	C	-0.1429370	1.5710500	-1.5817940
6	N	0.5002190	0.4288870	-1.1576170
7	C	1.8412300	0.2980680	-0.8321220
8	C	2.7952130	1.3106370	-0.9533810
9	C	4.1114510	1.0623810	-0.6247890
10	C	4.5053680	-0.1798920	-0.1696120
11	C	5.9050800	-0.4153840	0.2306480
12	F	6.7728300	0.3632370	-0.4602110
13	F	6.1479050	-0.1534250	1.5438360
14	F	6.2791330	-1.7050000	0.0450610
15	C	3.5674630	-1.1909510	-0.0432740
16	C	2.2538990	-0.9601160	-0.3693310
17	O	0.3895580	2.6880080	-1.5727010
18	O	-3.7238370	3.9207550	-0.9728450
19	H	-1.7757140	3.3225250	-2.6324570
20	H	-3.1013850	2.2016030	-2.8441320
21	H	-1.8020880	0.4370460	-2.0742390
22	H	-0.0532060	-0.4075840	-0.9710820
23	H	2.4862870	2.2699200	-1.2927810
24	H	4.8359510	1.8443620	-0.7318810
25	H	3.8686260	-2.1599870	0.3000120
26	H	1.5171790	-1.7313140	-0.2780940
27	C	-1.1580930	0.0047470	4.3197670
28	N	-1.7267750	0.4531910	2.9930520
29	C	-2.8595800	1.4355970	3.2295230
30	C	-0.6384320	1.1628070	2.2037260
31	C	-2.2021410	-0.7824340	2.2390250
32	C	-3.1997910	-0.4981840	1.1083260
33	O	-3.2842910	-1.7414220	0.3582460
34	P	-2.2733560	-2.0396400	-0.9074810
35	O	-0.8018800	-1.9096720	-0.4100040
36	O	-2.6228100	-3.6113750	-1.1563800
37	C	-3.9705840	-4.1554860	-1.1439250
38	O	-2.6729460	-1.1521630	-2.1163270
39	H	-2.6537710	-1.4455940	2.9596180
40	H	-1.3334130	-1.2444350	1.8022100
41	H	-2.8961460	0.3283000	0.4857710
42	H	-4.1895180	-0.3117250	1.4876550
43	H	-4.5720500	-3.6915730	-1.9132290
44	H	-4.4262800	-3.9944010	-0.1804310
45	H	-3.8737630	-5.2091220	-1.3436130
46	H	-3.6908690	0.9066990	3.6690510
47	H	-3.1109100	1.8843890	2.2808520
48	H	-2.5029960	2.1919070	3.9117860
49	H	-0.3714940	-0.7096500	4.1335060
50	H	-1.9463910	-0.4471330	4.9005150
51	H	-0.7644170	0.8677420	4.8321600
52	H	0.1461880	0.4533820	1.9955150
53	H	-0.2597250	1.9660210	2.8179580
54	H	-1.0810190	1.5577990	1.3036790

Table S23 - Cartesian coordinates (given in Angstroms) generated for structure **Q** (Figure S221), calculated at HF/3-21G level, after energy minimisation calculations.

Atom	X	Y	Z
1 C	-2.8232400	3.3147680	0.0036420
2 C	-2.5841810	2.6571970	-1.3677540
3 N	-1.7277090	1.4874810	-1.2918650
4 C	-0.4224900	1.6721250	-1.0023780
5 N	0.2949440	0.5174280	-0.8249660
6 C	1.6472850	0.4128380	-0.5252380
7 C	2.5319880	1.4891500	-0.4444090
8 C	3.8621760	1.2688060	-0.1519550
9 C	4.3365420	-0.0087840	0.0643030
10 C	5.7500250	-0.2257730	0.4268280
11 F	6.5616550	0.7308360	-0.0851430
12 F	5.9755560	-0.2155780	1.7685060
13 F	6.2096280	-1.4246990	-0.0074810
14 C	3.4672490	-1.0839620	-0.0133240
15 C	2.1406000	-0.8801720	-0.3025490
16 O	0.0690040	2.8037040	-0.9178390
17 O	-2.7999330	2.5411190	1.0243820
18 O	-3.0710270	4.5257980	0.0119870
19 H	-2.1680270	3.4012240	-2.0267150
20 H	-3.5417680	2.3411990	-1.7652750
21 H	-2.1716170	0.5857920	-1.2433940
22 H	-0.1871920	-0.3807180	-0.8854050
23 H	2.1631110	2.4724000	-0.6103050
24 H	4.5339350	2.1018110	-0.1024100
25 H	3.8310660	-2.0789320	0.1454290
26 H	1.4562310	-1.7008980	-0.3601170
27 H	-3.8134670	0.4086420	3.2340200
28 H	-5.2805870	0.6517210	1.2481040
29 C	-3.8025980	-0.2864590	2.4093420
30 H	-4.4584730	-1.1158650	2.6172290
31 N	-4.2929060	0.4462680	1.1851830
32 H	-3.7246110	1.3611120	1.0931200
33 C	-2.3837640	-0.7939130	2.1781800
34 H	-4.0834380	-0.1209920	0.3298520
35 H	-1.9392820	-1.0672110	3.1196280
36 H	-1.7886390	-0.0199450	1.7129750
37 O	-2.3969940	-2.0115450	1.3822800
38 O	-3.2683290	-0.9652720	-0.8023190
39 P	-2.3046450	-2.0699040	-0.2517180
40 O	-0.8376150	-1.9827700	-0.7550720
41 O	-2.8969390	-3.5654960	-0.4829940
42 H	-2.9713290	-4.2413570	-2.4551030
43 C	-2.4347680	-4.4538430	-1.5424960
44 H	-2.6464490	-5.4570050	-1.2145890
45 H	-1.3759200	-4.3218440	-1.6992460

Table S24 - Cartesian coordinates (given in Angstroms) generated for structure **R** (Figure S221), calculated at HF/3-21G level, after energy minimisation calculations.

Atom	X	Y	Z
1 C	-3.5656650	2.8786250	-0.2767130
2 C	-2.0971440	3.3372950	-0.2472700
3 N	-1.2337840	2.1797180	-0.2237060
4 C	0.1034910	2.2679440	-0.1604980
5 N	0.7051450	1.0327220	-0.1341090
6 C	2.0536020	0.7481890	-0.0736760
7 C	3.0686600	1.7068250	-0.0296940
8 C	4.3880660	1.3115400	0.0286930
9 C	4.7266390	-0.0273730	0.0445370
10 C	6.1393890	-0.4347000	0.1610220
11 F	6.9794630	0.4666560	-0.4032030
12 F	6.5633990	-0.5711040	1.4467340
13 F	6.3742240	-1.6335250	-0.4262200
14 C	3.7289950	-0.9864080	-0.0028130
15 C	2.4110040	-0.6064840	-0.0611700
16 O	0.7120560	3.3441370	-0.1289950
17 O	-3.7453490	1.6160560	-0.2395240
18 O	-4.4326170	3.7558330	-0.3326100
19 H	-1.9546560	3.9630250	0.6266960
20 H	-1.9211990	3.9572460	-1.1190560
21 H	-1.6935030	1.2884750	-0.2482210
22 H	0.0677100	0.2349460	-0.1581110
23 H	2.8024120	2.7368030	-0.0452580
24 H	5.1589010	2.0552460	0.0531340
25 H	3.9851870	-2.0263540	-0.0032910
26 H	1.6338380	-1.3425250	-0.1016920
27 H	-5.8960050	-0.1343400	1.8011190
28 H	-6.2419350	-0.4292840	-0.5680730
29 C	-5.2760810	-0.8294600	1.2567630
30 H	-5.6740760	-1.8269960	1.3474170
31 N	-5.3004460	-0.4274520	-0.2010520
32 H	-4.8486420	0.5306610	-0.2901760
33 C	-3.8489590	-0.7735630	1.7990070
34 H	-4.6800050	-1.0767010	-0.7562060
35 H	-3.8715620	-0.8860020	2.8697430
36 H	-3.3961620	0.1668350	1.5308540
37 O	-3.0569510	-1.8847350	1.2986670
38 O	-3.3905840	-1.8971290	-1.2695990
39 P	-2.2998010	-1.9130100	-0.1560700
40 O	-1.1974890	-0.8263550	-0.2046450
41 O	-1.6476500	-3.4039950	-0.0521170
42 H	-2.4874190	-4.2097080	-1.7848450
43 C	-1.5992790	-4.3191370	-1.1830800
44 H	-1.5398990	-5.3135350	-0.7742830
45 H	-0.7237700	-4.1160560	-1.7825120

Table S25 - Cartesian coordinates (given in Angstroms) generated for structure **S** (Figure S221), calculated at HF/3-21G level, after energy minimisation calculations.

Atom		X	Y	Z
1	C	-3.4902090	-1.6466640	-0.1270090
2	C	-2.1620980	-1.8456610	-0.4818740
3	C	-1.3274880	-0.7372150	-0.6973130
4	C	-1.8542280	0.5589150	-0.5488550
5	C	-3.1807460	0.7429510	-0.1961750
6	C	-4.0064010	-0.3610970	0.0152150
7	C	-5.4228600	-0.1705780	0.4499120
8	N	0.0088340	-0.8253990	-1.0697670
9	C	0.7922070	-1.9580600	-1.1428770
10	O	0.4148510	-3.0901770	-0.8593880
11	N	2.0570910	-1.6808560	-1.6074870
12	C	3.1297130	-2.6128070	-1.3303560
13	C	3.7411410	-2.3384220	0.0408710
14	O	2.8405650	-2.3933200	1.0073070
15	O	4.9092280	-2.0461230	0.2075520
16	F	-6.2266480	-1.1471180	-0.0062720
17	F	-5.9315600	0.9980400	0.0233920
18	F	-5.5536910	-0.1731720	1.7918860
19	H	-4.1336710	-2.5067970	0.0318220
20	H	-1.7651110	-2.8437540	-0.5959320
21	H	-1.2060940	1.4177720	-0.7130550
22	H	-3.5777720	1.7474310	-0.0891170
23	H	0.4926720	0.0606370	-1.2727370
24	H	2.2975620	-0.6927380	-1.7028060
25	H	3.9113190	-2.5233420	-2.0857330
26	H	2.7141130	-3.6228230	-1.3515580
27	H	3.1839300	-1.8844220	1.8403540
28	N	3.3357930	-0.7384480	2.9746840
29	H	3.1053020	-1.0948380	3.9001810
30	H	4.2676310	-0.3317230	3.0480410
31	C	2.3587310	0.2863440	2.5728810
32	C	2.7491730	0.8602150	1.2223240
33	O	1.7292730	1.7607060	0.8137360
34	P	1.9146150	2.3727240	-0.7005020
35	O	1.7876750	1.2377560	-1.6916120
36	O	0.5114000	3.1887880	-0.7795180
37	C	0.3623510	4.3208280	0.0738040
38	O	3.0953670	3.2843430	-0.7459000
39	H	2.2698830	1.0978080	3.3047060
40	H	1.3807360	-0.1955390	2.4781300
41	H	2.8395020	0.0518420	0.4874430
42	H	3.7134660	1.3825580	1.2852800
43	H	-0.5803450	4.8016040	-0.1905600
44	H	1.1884170	5.0221600	-0.0727760
45	H	0.3317090	4.0082110	1.1220730

Table S26 - Cartesian coordinates (given in Angstroms) generated for structure **T** (Figure S222), calculated at HF/3-21G level, after energy minimisation calculations.

Atom		X	Y	Z
1	O	3.503412	1.54027	0.220174
2	C	3.356874	2.782669	0.435073
3	C	1.903637	3.270422	0.599069
4	N	1.008092	2.137568	0.626084
5	C	-0.318027	2.252941	0.447499
6	N	-0.943884	1.029228	0.431547
7	C	-2.278951	0.758525	0.215046
8	C	-3.272567	1.734656	0.102329
9	C	-4.584900	1.366361	-0.102334
10	C	-4.937433	0.034913	-0.199647
11	C	-6.335661	-0.344866	-0.472605
12	F	-7.217646	0.553363	0.030172
13	F	-6.628051	-0.436188	-1.798841
14	F	-6.647654	-1.556497	0.049243
15	C	-3.960012	-0.940625	-0.093194
16	C	-2.646840	-0.591832	0.109489
17	O	-0.894805	3.339071	0.304996
18	O	4.243640	3.643184	0.519133
19	H	1.669258	3.932701	-0.226599
20	H	1.852534	3.860871	1.507514
21	H	1.432875	1.227671	0.696683
22	H	-0.315180	0.232725	0.594887
23	H	-2.993404	2.758914	0.175448
24	H	-5.338927	2.124000	-0.176597
25	H	-4.229670	-1.975016	-0.165010
26	H	-1.892612	-1.353075	0.172386
27	C	4.972525	-0.154549	-2.242594
28	C	4.318205	-1.119812	-1.254032
29	C	2.808464	-1.139120	-1.411225
30	O	2.271272	-2.281358	-0.695029
31	P	1.153847	-2.203111	0.505285
32	O	-0.124244	-2.985101	0.122247
33	O	1.927265	-3.000347	1.707913
34	C	3.047918	-2.399548	2.418915
35	O	0.996086	-0.709797	0.917609
36	O	4.730532	-0.710298	0.069530
37	H	4.706132	-2.114667	-1.409024
38	H	2.544275	-1.258247	-2.452186
39	H	2.396751	-0.225767	-1.019303
40	H	2.684061	-1.662029	3.119686
41	H	3.729993	-1.935736	1.722884
42	H	3.536182	-3.200416	2.950131
43	H	4.515371	0.820520	-2.131328
44	H	4.866103	-0.495579	-3.260674
45	O	6.384534	-0.088435	-1.940829
46	H	6.445850	-0.130058	-0.972938
47	H	4.332482	0.187867	0.249493

Table S27 - Cartesian coordinates (given in Angstroms) generated for structure **U** (Figure S222), calculated at HF/3-21G level, after energy minimisation calculations.

Atom	X	Y	Z
1 O	4.1690690	-2.2013370	-0.3672220
2 C	2.9446980	-2.4784420	-0.3111400
3 C	2.1952380	-2.7737820	-1.6177380
4 N	1.1639910	-1.7508770	-1.7551740
5 C	-0.0919940	-1.9686140	-1.2906830
6 N	-0.7292280	-0.7835470	-1.0153790
7 C	-2.0365630	-0.5813140	-0.6215190
8 C	-2.9978620	-1.5909280	-0.5451840
9 C	-4.2870080	-1.2896490	-0.1609320
10 C	-4.6438140	0.0061290	0.1551930
11 C	-6.0120940	0.3077560	0.6156610
12 F	-6.9279570	-0.5399400	0.0869250
13 F	-6.1670770	0.2147210	1.9643760
14 F	-6.3923680	1.5686650	0.2948340
15 C	-3.6960360	1.0137170	0.0877370
16 C	-2.4068760	0.7312880	-0.2939420
17 O	-0.5971650	-3.0854120	-1.1553340
18 O	2.2285700	-2.4548320	0.7279720
19 H	1.7153160	-3.7380900	-1.5675000
20 H	2.8822990	-2.7357540	-2.4470410
21 H	1.4747290	-0.7880870	-1.7441050
22 H	-0.1162100	0.0337780	-1.0631700
23 H	-2.7140380	-2.5890880	-0.7819640
24 H	-5.0196110	-2.0700700	-0.1162030
25 H	-3.9706460	2.0209600	0.3278280
26 H	-1.6723690	1.5135410	-0.3327950
27 C	4.2607010	-0.5552520	2.7181040
28 C	4.0039260	0.5837990	1.7020370
29 C	2.5077500	0.8329130	1.5709290
30 O	2.3056690	2.0931450	0.8763480
31 P	1.4672980	2.3124230	-0.5116840
32 O	0.1761980	3.1273850	-0.2650860
33 O	2.5316110	3.2216590	-1.3597250
34 C	3.7742900	2.6472950	-1.8608710
35 O	1.3149050	0.9357420	-1.2262840
36 O	4.6157560	0.3337610	0.4221350
37 H	4.4598190	1.4918510	2.0673070
38 H	2.0587210	0.9235830	2.5456670
39 H	2.0418360	0.0283200	1.0288950
40 H	3.5867660	2.1349520	-2.7935940
41 H	4.1897340	1.9571750	-1.1417340
42 H	4.4483370	3.4721390	-2.0271300
43 H	4.5065760	-0.1185900	3.6758030
44 H	5.1237520	-1.1152780	2.3733960
45 O	3.1237050	-1.3964400	2.9491890
46 H	2.8312890	-1.8582430	2.1183230
47 H	4.4748290	-0.5983300	0.1300750

Table S28 - Cartesian coordinates (given in Angstroms) generated for structure **V** (Figure S222), calculated at HF/3-21G level, after energy minimisation calculations.

Atom	X	Y	Z
1 C	2.8151010	-2.7488900	-0.2942630
2 C	2.1740970	-2.7635610	-1.6976220
3 N	1.1620100	-1.7250210	-1.7905260
4 C	-0.0890630	-1.9500190	-1.3239310
5 N	-0.7382450	-0.7748090	-1.0296570
6 C	-2.0355460	-0.6065230	-0.5833490
7 C	-2.9926200	-1.6229390	-0.5568830
8 C	-4.2732730	-1.3554790	-0.1223160
9 C	-4.6260410	-0.0871130	0.2936520
10 C	-5.9843940	0.1757570	0.8045690
11 F	-6.9100810	-0.6355510	0.2373240
12 F	-6.1087750	-0.0134680	2.1463840
13 F	-6.3745320	1.4548700	0.5828880
14 C	-3.6812690	0.9251130	0.2810430
15 C	-2.3998570	0.6752980	-0.1476010
16 O	-0.5913910	-3.0706700	-1.2090310
17 O	2.0685970	-2.4745920	0.6694890
18 O	4.0562960	-2.9942710	-0.2421680
19 H	1.7069980	-3.7228840	-1.8721960
20 H	2.9452800	-2.6036660	-2.4342140
21 H	1.4746130	-0.7656060	-1.7792450
22 H	-0.1569430	0.0592150	-1.1192960
23 H	-2.7120260	-2.6008340	-0.8689980
24 H	-5.0027880	-2.1401070	-0.1178230
25 H	-3.9507610	1.9102140	0.6046070
26 H	-1.6625820	1.4553160	-0.1278800
27 C	4.3249510	-0.9038010	2.3968570
28 C	3.6310330	0.4387630	2.1966740
29 C	3.0267240	0.5336510	0.8149310
30 O	2.4970120	1.8918150	0.7052340
31 P	1.4251810	2.3050840	-0.4657560
32 O	0.1587780	2.9419200	0.1557850
33 O	2.3143540	3.4155540	-1.2834960
34 C	2.1665030	3.6045650	-2.7165660
35 O	1.2244750	1.0688560	-1.3966160
36 O	4.5940330	1.5028980	2.3601590
37 H	2.8431830	0.5365460	2.9357610
38 H	2.2585000	-0.2016130	0.6734990
39 H	3.8079740	0.3960400	0.0862490
40 H	1.2848560	4.1937820	-2.9275420
41 H	2.0887220	2.6481880	-3.2091240
42 H	3.0432050	4.1314320	-3.0542790
43 H	3.5577660	-1.6608210	2.4648500
44 H	4.8769970	-0.8637910	3.3275150
45 O	5.2296410	-1.2034320	1.3214360
46 H	4.8458180	-1.9160260	0.7474020
47 H	4.1951850	2.3009540	1.9814590

Table S29 - Cartesian coordinates (given in Angstroms) generated for structure **W** (Figure S222), calculated at HF/3-21G level, after energy minimisation calculations.

Atom	X	Y	Z
1 O	-4.1605700	3.9622320	-0.1373850
2 C	-3.3481750	3.0292620	-0.1902810
3 C	-1.8799370	3.4112740	-0.4390000
4 N	-1.0426520	2.2335600	-0.4616700
5 C	0.2954480	2.3105390	-0.3572120
6 N	0.8914240	1.0747270	-0.3102570
7 C	2.2344040	0.7907550	-0.1483140
8 C	3.2516610	1.7476480	-0.1927650
9 C	4.5679130	1.3672190	-0.0407320
10 C	4.9007290	0.0423390	0.1583680
11 C	6.3067020	-0.3465680	0.3732040
12 F	7.1704780	0.4876150	-0.2553830
13 F	6.6813270	-0.3383720	1.6816820
14 F	6.5625260	-1.6031630	-0.0664960
15 C	3.8998290	-0.9132410	0.2111400
16 C	2.5824540	-0.5516600	0.0634000
17 O	0.9050200	3.3865230	-0.2988530
18 O	-3.5969480	1.7876620	-0.0692970
19 H	-1.5709030	4.0912770	0.3450730
20 H	-1.8367970	3.9626730	-1.3730050
21 H	-1.5041810	1.3414700	-0.4830170
22 H	0.2424680	0.2830630	-0.4040340
23 H	2.9891590	2.7680080	-0.3406790
24 H	5.3393600	2.1091870	-0.0884650
25 H	4.1535900	-1.9427290	0.3655470
26 H	1.8109350	-1.2947130	0.1322710
27 C	-5.6258950	-0.2946090	1.5382790
28 C	-4.7314660	-1.5083620	1.2033480
29 C	-3.2670000	-1.1188840	1.2933670
30 O	-2.4450000	-2.2591490	0.9225590
31 P	-1.1887470	-2.1553720	-0.1217730
32 O	0.0781980	-2.7835970	0.5098610
33 O	-1.7322310	-3.0808440	-1.3652270
34 C	-1.4985690	-2.7166230	-2.7507980
35 O	-1.0832420	-0.6864370	-0.6357320
36 O	-5.0693380	-1.9241470	-0.1337570
37 H	-4.9297720	-2.3291340	1.8759460
38 H	-3.0255230	-0.8546650	2.3142290
39 H	-3.0588310	-0.2841100	0.6491230
40 H	-0.4987840	-3.0016170	-3.0487290
41 H	-1.6233760	-1.6539860	-2.8819550
42 H	-2.2184750	-3.2570000	-3.3428190
43 H	-5.2150050	0.2750550	2.3635780
44 H	-6.6115980	-0.6383000	1.8164100
45 O	-5.7746010	0.5013340	0.3489680
46 H	-4.9647360	1.0763680	0.1864560
47 H	-5.4155980	-1.1161510	-0.5551840

Table S30 - Cartesian coordinates (given in Angstroms) generated for structure **X** (Figure S222), calculated at HF/3-21G level, after energy minimisation calculations.

Atom	X	Y	Z
1 O	-4.2888410	3.9190560	0.0165920
2 C	-3.4495810	3.0204800	-0.1367220
3 C	-1.9815420	3.4705040	-0.2472910
4 N	-1.1132470	2.3227680	-0.3819390
5 C	0.2197370	2.4124450	-0.2345190
6 N	0.8399400	1.1904870	-0.3097980
7 C	2.1839560	0.9122540	-0.1686800
8 C	3.1878640	1.8779000	-0.0633450
9 C	4.5065290	1.4935610	0.0546190
10 C	4.8548080	0.1574490	0.0718970
11 C	6.2632890	-0.2412780	0.2504630
12 F	7.1229650	0.6827060	-0.2432910
13 F	6.6213390	-0.4120820	1.5522430
14 F	6.5407900	-1.4208790	-0.3572190
15 C	3.8671560	-0.8085750	-0.0251300
16 C	2.5490850	-0.4415060	-0.1413890
17 O	0.8065670	3.4848420	-0.0417420
18 O	-3.6659140	1.7709320	-0.2224230
19 H	-1.7368760	4.0429140	0.6389170
20 H	-1.9031860	4.1440960	-1.0946010
21 H	-1.5599570	1.4347220	-0.5214850
22 H	0.2159670	0.3941110	-0.4853650
23 H	2.9130540	2.9057850	-0.0736730
24 H	5.2690040	2.2430630	0.1233240
25 H	4.1316410	-1.8466030	-0.0137550
26 H	1.7818260	-1.1878010	-0.1931310
27 C	-5.8868130	-0.7016900	0.9818140
28 C	-4.6688710	-1.5854300	0.6881430
29 C	-3.3917120	-0.9695060	1.2606600
30 O	-2.2602660	-1.8846680	1.0902140
31 P	-1.4568840	-2.0332540	-0.3274900
32 O	-1.0130300	-0.6301580	-0.8259940
33 O	-0.1450920	-2.8676380	0.1881130
34 C	0.3012020	-4.1095210	-0.4261250
35 O	-2.2899100	-2.9241050	-1.2953590
36 O	-4.6027240	-1.7490060	-0.7351170
37 H	-4.8229860	-2.5372210	1.1884260
38 H	-3.4914350	-0.8340330	2.3257610
39 H	-3.1791310	-0.0312440	0.7783490
40 H	0.6674200	-4.7432970	0.3646010
41 H	1.0976090	-3.8963580	-1.1231730
42 H	-0.5193880	-4.5822440	-0.9407630
43 H	-5.9126650	-0.5058880	2.0518590
44 H	-6.7707860	-1.2758150	0.7377170
45 O	-5.9605860	0.5053560	0.2223880
46 H	-5.0903760	0.9543720	0.0754930
47 H	-3.8614190	-2.3396380	-1.0093110

Table S31 - Cartesian coordinates (given in Angstroms) generated for structure **Y** (Figure S222), calculated at HF/3-21G level, after energy minimisation calculations.

Atom		X	Y	Z
1	O	3.6311130	1.6467910	-0.2269950
2	C	3.4248370	2.8820230	0.0411730
3	C	1.9903530	3.2726490	0.4294160
4	N	1.1122100	2.1313720	0.3347880
5	C	-0.2265690	2.2523780	0.3170040
6	N	-0.8481520	1.0367020	0.1650930
7	C	-2.1963250	0.7705530	0.0633060
8	C	-3.2002760	1.7374700	0.1606360
9	C	-4.5251170	1.3733690	0.0506070
10	C	-4.8798910	0.0551780	-0.1560820
11	C	-6.2965190	-0.3159290	-0.3257440
12	F	-7.1288850	0.5250230	0.3353880
13	F	-6.7150220	-0.2970450	-1.6208450
14	F	-6.5526350	-1.5718550	0.1162010
15	C	-3.8925620	-0.9117160	-0.2534870
16	C	-2.5672070	-0.5672940	-0.1475500
17	O	-0.8149810	3.3367730	0.4191330
18	O	4.2611970	3.7889570	0.0159930
19	H	1.6711530	4.0673510	-0.2333080
20	H	2.0191720	3.6880330	1.4324390
21	H	1.5361080	1.2245010	0.2168230
22	H	-0.1971820	0.2415670	0.1243530
23	H	-2.9191660	2.7512130	0.3216000
24	H	-5.2860860	2.1226590	0.1363930
25	H	-4.1643270	-1.9369310	-0.4054180
26	H	-1.8077650	-1.3219270	-0.2304140
27	C	5.7678370	-0.7060090	-1.0095490
28	C	4.6524350	-1.4724940	-0.2950220
29	C	3.5155760	-1.7619060	-1.2638950
30	O	2.5683050	-2.6842870	-0.6636850
31	P	1.2071170	-2.2126900	0.1194980
32	O	-0.0229510	-2.9446910	-0.4719450
33	O	1.4607900	-2.7478940	1.6465510
34	C	1.8217580	-1.8484900	2.7327680
35	O	1.1634180	-0.6567710	0.1334030
36	O	4.1742640	-0.7505960	0.8571680
37	H	5.0462620	-2.4078200	0.0743740
38	H	3.8898430	-2.2452410	-2.1522310
39	H	3.0234380	-0.8415080	-1.5218070
40	H	1.0487010	-1.1091340	2.8834390
41	H	2.7534130	-1.3645550	2.4997500
42	H	1.9150980	-2.4638860	3.6126980
43	H	6.3017690	-1.3801930	-1.6649130
44	H	6.4595690	-0.3421050	-0.2563680
45	O	5.2609150	0.3585050	-1.8303140
46	H	4.7090500	0.9677800	-1.2784710
47	H	3.8281160	0.1259520	0.5767810

Table S32 - Cartesian coordinates (given in Angstroms) generated for structure **Z** (Figure S222), calculated at HF/3-21G level, after energy minimisation calculations.

Atom		X	Y	Z
1	O	-3.1010370	1.8009090	-0.1431400
2	C	-3.1799140	2.4432990	-1.2346200
3	C	-1.9857070	2.3449870	-2.2031360
4	N	-1.0542220	1.2930220	-1.8551080
5	C	0.1813650	1.5763870	-1.4068730
6	N	0.8419870	0.4955110	-0.8716980
7	C	2.1702960	0.4401290	-0.4808660
8	C	3.0923210	1.4786910	-0.6275520
9	C	4.4006750	1.3028410	-0.2277230
10	C	4.8179900	0.1085860	0.3241320
11	C	6.2064040	-0.0479650	0.7956520
12	F	7.0736670	0.7263960	0.0992470
13	F	6.3813620	0.2937550	2.1012930
14	F	6.6374110	-1.3296680	0.6981810
15	C	3.9112100	-0.9272090	0.4781460
16	C	2.6063510	-0.7682750	0.0828720
17	O	0.6799920	2.7093150	-1.4717730
18	O	-4.1165790	3.1684200	-1.6087610
19	H	-1.4588130	3.2879200	-2.1984590
20	H	-2.3891950	2.1961720	-3.1969360
21	H	-1.4267480	0.3510480	-1.8537060
22	H	0.3107610	-0.3540280	-0.6593290
23	H	2.7659280	2.4012500	-1.0436850
24	H	5.1010810	2.1033950	-0.3556610
25	H	4.2307830	-1.8596670	0.8978570
26	H	1.8928350	-1.5581320	0.1954850
27	C	-3.6415000	0.6676570	3.3947240
28	C	-3.8854020	-0.2688510	2.2055410
29	C	-2.5751500	-0.7049710	1.5809150
30	O	-2.8763150	-1.7523090	0.6133750
31	P	-1.8392520	-2.0673140	-0.6147930
32	O	-0.3891630	-1.8269770	-0.1005070
33	O	-2.1581500	-3.6613720	-0.7938050
34	C	-2.0738220	-4.3353160	-2.0781680
35	O	-2.2298990	-1.3084560	-1.9133580
36	O	-4.7348490	0.4363060	1.2838560
37	H	-4.4277620	-1.1405380	2.5415660
38	H	-1.9075680	-1.1025790	2.3308440
39	H	-2.1218840	0.1371300	1.0850800
40	H	-1.0546430	-4.6398240	-2.2700090
41	H	-2.4101030	-3.6771520	-2.8641990
42	H	-2.7085530	-5.2037280	-2.0185790
43	H	-2.9377750	1.4427930	3.1070560
44	H	-3.2487840	0.1327170	4.2459470
45	O	-4.9086650	1.2491740	3.7647710
46	H	-5.3833010	1.3501150	2.9212580
47	H	-4.1771770	1.0287150	0.6929880

Table S33 - Cartesian coordinates (given in Angstroms) generated for structure **AA** (Figure S222), calculated at HF/3-21G level, after energy minimisation calculations.

Atom		X	Y	Z
1	O	4.1515740	-2.0095050	-1.0632230
2	C	2.9056620	-2.1223210	-1.0039540
3	C	2.0842940	-1.9240010	-2.2858920
4	N	1.0570640	-0.9274450	-2.0190480
5	C	-0.1610710	-1.3099050	-1.5830530
6	N	-0.8323580	-0.3175810	-0.9084190
7	C	-2.1656160	-0.3151530	-0.5352670
8	C	-3.0883010	-1.3101760	-0.8637500
9	C	-4.4037540	-1.1900130	-0.4669310
10	C	-4.8261820	-0.0956330	0.2607420
11	C	-6.2229820	-0.0059150	0.7252060
12	F	-7.0798810	-0.6514560	-0.1028290
13	F	-6.4252150	-0.5516200	1.9553520
14	F	-6.6457040	1.2781510	0.8270280
15	C	-3.9180790	0.8950710	0.5965440
16	C	-2.6063850	0.7908500	0.2056380
17	O	-0.6323950	-2.4338200	-1.7877240
18	O	2.2332900	-2.3375940	0.0506210
19	H	1.6060030	-2.8507170	-2.5636900
20	H	2.7365910	-1.5910230	-3.0765940
21	H	1.3918100	0.0263950	-1.9156430
22	H	-0.2902740	0.4690880	-0.5377850
23	H	-2.7568010	-2.1593890	-1.4119430
24	H	-5.1053470	-1.9542410	-0.7342920
25	H	-4.2418070	1.7504180	1.1544820
26	H	-1.8911900	1.5465410	0.4573590
27	C	4.1647460	-1.4136280	2.8519310
28	C	4.2521160	-0.1390340	1.9971770
29	C	2.8602720	0.3498720	1.6071590
30	O	3.0077410	1.6161140	0.9015160
31	P	1.9104960	2.1074450	-0.2081230
32	O	0.4805450	1.7644630	0.3053800
33	O	2.2056670	3.7140710	-0.1361960
34	C	2.1008550	4.5781120	-1.2995470
35	O	2.2558310	1.5801820	-1.6299490
36	O	5.1135820	-0.3352160	0.8717830
37	H	4.7067400	0.6360760	2.5985300
38	H	2.2728050	0.5195310	2.4976520
39	H	2.3652540	-0.3794290	0.9885710
40	H	1.0786640	4.9062000	-1.4251060
41	H	2.4235610	4.0497590	-2.1829880
42	H	2.7363200	5.4286310	-1.1167960
43	H	3.4340650	-1.2589970	3.6419830
44	H	5.1261880	-1.5638270	3.3246480
45	O	3.8948000	-2.6021230	2.1008960
46	H	3.2143010	-2.4889460	1.3891610
47	H	4.7814130	-1.0395530	0.2697670

Table S34 - Cartesian coordinates (given in Angstroms) generated for structure **AB** (Figure S222), calculated at HF/3-21G level, after energy minimisation calculations.

Atom	X	Y	Z
1 O	-3.4990670	3.8379020	-1.0559680
2 C	-2.6861090	2.9107330	-0.9045180
3 C	-2.1116540	2.3038230	-2.2002590
4 N	-1.2144700	1.1922590	-1.9692750
5 C	0.0356430	1.4427770	-1.5366950
6 N	0.7036160	0.3414900	-1.0555010
7 C	2.0265710	0.2840260	-0.6430170
8 C	2.9401470	1.3362290	-0.7349820
9 C	4.2413850	1.1606590	-0.3121680
10 C	4.6601400	-0.0472020	0.2078220
11 C	6.0392800	-0.2045930	0.7057410
12 F	6.9141650	0.5979690	0.0521190
13 F	6.1814020	0.1004560	2.0243720
14 F	6.4850220	-1.4787920	0.5807250
15 C	3.7620850	-1.0970800	0.3061010
16 C	2.4640630	-0.9382690	-0.1114920
17 O	0.5452800	2.5690760	-1.5941820
18 O	-2.2834410	2.4184320	0.1960180
19 H	-1.6076110	3.0912870	-2.7411080
20 H	-2.9448250	1.9649190	-2.8016200
21 H	-1.6457600	0.2791540	-1.8889950
22 H	0.1926300	-0.5315800	-0.9155700
23 H	2.6128430	2.2680120	-1.1286670
24 H	4.9352380	1.9725210	-0.3977160
25 H	4.0826450	-2.0397260	0.7015630
26 H	1.7568800	-1.7382320	-0.0380250
27 C	-3.3002600	1.1546230	3.2430200
28 C	-3.3127850	-0.2692180	2.6847140
29 C	-2.3527470	-0.4287800	1.5161430
30 O	-2.6820630	-1.7203580	0.9073450
31 P	-2.0233990	-2.1185610	-0.5446780
32 O	-0.4724310	-2.0778710	-0.4278010
33 O	-2.5815670	-3.6504720	-0.6370950
34 C	-2.9661730	-4.2600230	-1.9002130
35 O	-2.6399740	-1.2555960	-1.6803150
36 O	-4.6474280	-0.5789710	2.2448530
37 H	-3.0114560	-0.9481660	3.4751060
38 H	-1.3275190	-0.4417490	1.8550350
39 H	-2.4870690	0.3563440	0.7917860
40 H	-2.0991960	-4.6913480	-2.3798280
41 H	-3.4096190	-3.5197560	-2.5474910
42 H	-3.6786620	-5.0342640	-1.6698810
43 H	-2.2935320	1.3669670	3.5966020
44 H	-3.9574220	1.1758920	4.1019220
45 O	-3.7770260	2.1450070	2.3349030
46 H	-3.1807280	2.2852570	1.5511550
47 H	-4.5709640	-1.3334890	1.6411410

Table S35 - Cartesian coordinates (given in Angstroms) generated for structure **AC** (Figure S222), calculated at HF/3-21G level, after energy minimisation calculations.

Atom	X	Y	Z
1 O	-3.6148440	3.9172690	-0.6162300
2 C	-3.0482650	2.8188920	-0.6046460
3 C	-1.9966940	2.5684880	-1.6959690
4 N	-1.0976450	1.4746910	-1.3872290
5 C	0.1753480	1.7155240	-1.0249310
6 N	0.8422350	0.6061920	-0.5603930
7 C	2.1960600	0.5051270	-0.2813120
8 C	3.1294570	1.5285550	-0.4608330
9 C	4.4603530	1.3088180	-0.1732560
10 C	4.8903460	0.0845150	0.2969880
11 C	6.3071930	-0.1197530	0.6506010
12 F	7.1367470	0.6525390	-0.0927350
13 F	6.5955980	0.1807250	1.9462710
14 F	6.6943570	-1.4083110	0.4846510
15 C	3.9734250	-0.9373530	0.4808000
16 C	2.6458220	-0.7348290	0.1966620
17 O	0.7009550	2.8354920	-1.1052780
18 O	-3.2866270	1.8517170	0.2010890
19 H	-1.4283970	3.4719620	-1.8350720
20 H	-2.5286060	2.3605340	-2.6194130
21 H	-1.4870960	0.5363080	-1.4010520
22 H	0.3040420	-0.2329780	-0.3237540
23 H	2.7940600	2.4733060	-0.8150770
24 H	5.1676890	2.0992000	-0.3247750
25 H	4.3024300	-1.8931030	0.8357570
26 H	1.9242930	-1.5137580	0.3310400
27 C	-5.1234130	0.2967170	2.4311550
28 C	-4.6813870	-0.7497790	1.4031200
29 C	-3.2324940	-1.1403030	1.6524700
30 O	-2.8745290	-2.2213270	0.7460290
31 P	-1.7363320	-2.0650880	-0.4170220
32 O	-0.3705460	-1.7124930	0.2459740
33 O	-1.7596680	-3.6137470	-0.9463210
34 C	-1.5391210	-3.9532510	-2.3409370
35 O	-2.1707240	-1.1221300	-1.5725740
36 O	-4.8575680	-0.2745610	0.0601700
37 H	-5.3045120	-1.6266660	1.5060080
38 H	-3.0989960	-1.5046310	2.6590780
39 H	-2.6011940	-0.2810710	1.4942270
40 H	-0.4793970	-4.0265770	-2.5419090
41 H	-1.9767990	-3.2014050	-2.9783050
42 H	-2.0071150	-4.9089220	-2.5096110
43 H	-5.2597180	-0.1790080	3.3928620
44 H	-6.0812770	0.6919970	2.1074730
45 O	-4.1515710	1.3393240	2.6166120
46 H	-3.8605610	1.6928840	1.7382950
47 H	-4.2451070	0.4829120	-0.0860110

Cartesian coordinates calculated at M06-2X/6-31G level

Table S36 - Cartesian coordinates (given in Angstroms) generated for structure **O** (Figure S218), calculated at M06-2X/6-31G level, after energy minimisation calculations.

Atom		X	Y	Z
1	C	-5.7261150	-0.5694850	1.8820220
2	N	-4.9287770	-0.5565200	0.6151430
3	C	-5.8063700	-0.0728990	-0.4956800
4	C	-3.7661550	0.3791840	0.7949410
5	C	-4.4502080	-1.9644300	0.3483250
6	C	-3.7622670	-2.1541360	-1.0144850
7	O	-2.5707050	-2.9108530	-0.8708790
8	P	-1.1782350	-2.1018190	-0.5842420
9	O	-1.1998000	-0.7994630	-1.3547100
10	O	-1.4251840	-1.7590320	0.9945370
11	C	-0.5969810	-0.7258450	1.5388820
12	O	-0.0482890	-3.0586520	-0.7623850
13	H	-5.3299120	-2.6043750	0.4417050
14	H	-3.7414520	-2.2033220	1.1432820
15	H	-3.5247100	-1.2011770	-1.4963550
16	H	-4.4135090	-2.7179230	-1.6849260
17	H	-0.7911030	-0.6989720	2.6129970
18	H	-0.8422160	0.2472020	1.0990090
19	H	0.4634330	-0.9592040	1.3792410
20	H	-6.5982940	-0.8045460	-0.6593370
21	H	-5.2111470	0.0530450	-1.3989510
22	H	-6.2318870	0.8869450	-0.2027580
23	H	-5.1088220	-0.9909860	2.6752860
24	H	-6.6179970	-1.1778060	1.7306690
25	H	-6.0033650	0.4560640	2.1252780
26	H	-3.2398400	0.1053100	1.7083070
27	H	-4.1445450	1.4003170	0.8656180
28	H	-3.0811480	0.2816830	-0.0495760
29	C	4.5807310	1.2167850	-0.0264980
30	C	3.3014780	1.6237670	-0.3820590
31	C	2.3078140	0.6609220	-0.6283950
32	C	2.6363590	-0.7041070	-0.5207270
33	C	3.9168620	-1.0954790	-0.1658790
34	C	4.8970290	-0.1352660	0.0842400
35	C	6.2611750	-0.5472550	0.5302090
36	N	1.0051420	0.9633830	-0.9968250
37	C	0.3694800	2.1968420	-0.9497570
38	O	0.9277590	3.2447100	-0.6346450
39	N	-0.9313130	2.1195870	-1.3624640
40	C	-1.8504850	3.1661860	-0.9699470
41	C	-2.3336710	3.0777240	0.5037160
42	O	-1.6929220	2.3251470	1.2744530
43	O	-3.3413360	3.7739980	0.7708040
44	F	7.2145920	0.2913510	0.0869960
45	F	6.5851670	-1.7798900	0.1036390
46	F	6.3770280	-0.5686920	1.8736450
47	H	5.3448290	1.9661560	0.1576300
48	H	3.0595130	2.6727200	-0.4698410
49	H	1.8711230	-1.4552920	-0.7131910
50	H	4.1551390	-2.1514860	-0.0866330
51	H	0.3852570	0.1657360	-1.1714450
52	H	-1.3148480	1.1755570	-1.3755690
53	H	-2.7242440	3.1370350	-1.6271660
54	H	-1.3613830	4.1326380	-1.1199980

Table S37 - Cartesian coordinates (given in Angstroms) generated for structure **P** (Figure S220), calculated at M06-2X/6-31G level, after energy minimisation calculations.

Atom	X	Y	Z
1 O	2.7495590	-2.3803560	0.4335420
2 C	3.1436980	-2.9699330	-0.6070680
3 C	2.7605660	-2.3505110	-1.9790730
4 N	1.8636030	-1.2173270	-1.9459580
5 C	0.5594810	-1.4201770	-1.6347680
6 N	-0.1556950	-0.2474140	-1.4145190
7 C	-1.5113620	-0.1481540	-1.1153750
8 C	-2.4407490	-1.1852010	-1.3012820
9 C	-3.7768260	-0.9836510	-0.9730090
10 C	-4.2109750	0.2373430	-0.4669200
11 C	-5.6361630	0.4403920	-0.0691190
12 F	-6.4580150	-0.4573460	-0.6363560
13 F	-5.8165030	0.3376030	1.2632410
14 F	-6.0832510	1.6628830	-0.4106350
15 C	-3.2945440	1.2744760	-0.2865540
16 C	-1.9606030	1.0873440	-0.6053550
17 O	0.0492860	-2.5402480	-1.5695410
18 O	3.8370650	-4.0040040	-0.6813500
19 H	2.3219400	-3.1431150	-2.5909650
20 H	3.6838890	-2.0332750	-2.4736570
21 H	2.2834220	-0.2916800	-1.8167890
22 H	0.3649340	0.6326960	-1.3117480
23 H	-2.1101870	-2.1339590	-1.6963640
24 H	-4.4890860	-1.7884760	-1.1242890
25 H	-3.6278430	2.2328750	0.1005810
26 H	-1.2379020	1.8871630	-0.4729490
27 C	-0.4980730	-0.4535540	3.6844040
28 N	0.5847520	-0.7663770	2.7019910
29 C	1.6683350	-1.5516130	3.3773480
30 C	0.0132850	-1.6033400	1.5960600
31 C	1.1128680	0.5436450	2.1663980
32 C	2.4454170	0.4520120	1.4257240
33 O	2.7843120	1.7794820	1.0383720
34 P	2.4526090	2.1770200	-0.5211350
35 O	0.9626990	2.2809300	-0.7029760
36 O	3.0523640	3.6769070	-0.5113790
37 C	4.4678450	3.8235280	-0.4047990
38 O	3.2323450	1.2720230	-1.4341200
39 H	1.2244640	1.2123290	3.0238380
40 H	0.3483380	0.9497250	1.4962640
41 H	2.3987270	-0.2352480	0.5744550
42 H	3.2404940	0.0981920	2.0865330
43 H	4.9702180	3.3004590	-1.2224980
44 H	4.8225920	3.4300920	0.5525160
45 H	4.6831980	4.8909340	-0.4622810
46 H	2.1809580	-0.9042120	4.0904090
47 H	2.3461060	-1.9282600	2.6078900
48 H	1.1975220	-2.3840380	3.9015010
49 H	-1.2458140	0.1661570	3.1874210
50 H	-0.0635910	0.0808530	4.5294170
51 H	-0.9451250	-1.3899150	4.0179150
52 H	-0.8056960	-1.0502790	1.1354170
53 H	-0.3652690	-2.5251950	2.0414000
54 H	0.8116410	-1.8330590	0.8861000

Table S38 - Cartesian coordinates (given in Angstroms) generated for structure **Q** (Figure S221), calculated at M06-2X/6-31G level, after energy minimisation calculations.

Atom	X	Y	Z
1 C	-2.6689970	3.5241850	-0.1659850
2 C	-2.6913550	2.4872960	-1.2915410
3 N	-1.8388910	1.3530450	-1.0347330
4 C	-0.5026500	1.5684150	-0.8875000
5 N	0.2366050	0.4139930	-0.7310420
6 C	1.6055790	0.3128480	-0.4957900
7 C	2.4903160	1.4026480	-0.4532570
8 C	3.8412010	1.1815670	-0.2134410
9 C	4.3314960	-0.1066480	-0.0185780
10 C	5.7757400	-0.3183920	0.2989980
11 F	6.5639070	0.5984440	-0.2886810
12 F	6.0267120	-0.2386160	1.6216010
13 F	6.2085600	-1.5288820	-0.0944840
14 C	3.4567110	-1.1924600	-0.0622520
15 C	2.1083090	-0.9868410	-0.2985060
16 O	-0.0285910	2.7051750	-0.8873890
17 O	-2.7283120	3.0538180	1.0654940
18 O	-2.6281720	4.7148020	-0.4004030
19 H	-2.4151090	3.0113660	-2.2110490
20 H	-3.7143520	2.1111090	-1.3971690
21 H	-2.2533150	0.4157480	-1.0592570
22 H	-0.2645280	-0.4871650	-0.7526910
23 H	2.1154710	2.4032480	-0.6090610
24 H	4.5214690	2.0273520	-0.1865970
25 H	3.8331350	-2.2000110	0.0838090
26 H	1.4160460	-1.8232490	-0.3337300
27 H	-2.7254020	0.7608780	3.2557870
28 H	-4.7372760	0.9935280	1.7315880
29 C	-3.1472920	0.0080760	2.5809100
30 H	-3.8487880	-0.6059450	3.1582380
31 N	-3.8043380	0.6915280	1.4553620
32 H	-3.0343950	2.0635700	1.1075000
33 C	-2.0306500	-0.8883770	2.0832210
34 H	-3.9166390	0.0405080	0.6700440
35 H	-1.4374620	-1.2526700	2.9256730
36 H	-1.3630930	-0.3315910	1.4113920
37 O	-2.5856970	-2.0321310	1.4247720
38 O	-3.2991870	-1.0942560	-0.8623130
39 P	-2.4220430	-2.1271310	-0.1902630
40 O	-0.9623830	-2.1287780	-0.5692190
41 O	-3.0713000	-3.6026820	-0.3264790
42 H	-4.0362190	-3.3757110	-2.1554310
43 C	-3.3293400	-4.0461230	-1.6583920
44 H	-3.7592860	-5.0449510	-1.5842540
45 H	-2.4011200	-4.0922560	-2.2369130

Table 39 - Cartesian coordinates (given in Angstroms) generated for structure **R** (Figure S221), calculated at M06-2X/6-31G level, after energy minimisation calculations.

Atom	X	Y	Z
1 C	-3.7849500	2.3981670	-0.2974220
2 C	-2.4184860	3.0638070	-0.5342490
3 N	-1.4387600	2.0373520	-0.8282090
4 C	-0.1400360	2.1662350	-0.4526910
5 N	0.5475650	0.9638460	-0.5181310
6 C	1.8984890	0.7420930	-0.3014830
7 C	2.8555060	1.7577860	-0.1368530
8 C	4.1911690	1.4211310	0.0420920
9 C	4.5986140	0.0894080	0.0604710
10 C	6.0324290	-0.2509100	0.3033020
11 F	6.8640610	0.6839050	-0.1895920
12 F	6.3231380	-0.3574730	1.6156820
13 F	6.3746940	-1.4248450	-0.2545200
14 C	3.6528380	-0.9237850	-0.0987010
15 C	2.3191310	-0.6008110	-0.2776510
16 O	0.3602870	3.2325490	-0.0962850
17 O	-3.9880810	1.3477110	-0.9938750
18 O	-4.5579950	2.8940710	0.5320950
19 H	-2.1223010	3.6216440	0.3566170
20 H	-2.5111240	3.7823420	-1.3608010
21 H	-1.8118470	1.1153800	-1.0568140
22 H	-0.0356790	0.1319620	-0.6616090
23 H	2.5444770	2.7922330	-0.1541090
24 H	4.9273210	2.2105980	0.1596450
25 H	3.9606620	-1.9645210	-0.0894390
26 H	1.5788820	-1.3862430	-0.4067710
27 H	-4.7427800	0.8021550	2.1273170
28 H	-6.1426880	-0.4709950	0.5938190
29 C	-4.4598770	-0.2005880	1.8002820
30 H	-4.8199580	-0.9410100	2.5168420
31 N	-5.1274020	-0.4401690	0.4910230
32 H	-4.8376640	0.3583170	-0.1930960
33 C	-2.9460740	-0.2861050	1.6763550
34 H	-4.7938050	-1.3254100	0.0491330
35 H	-2.5135900	-0.0423160	2.6498360
36 H	-2.5630580	0.4279570	0.9402960
37 O	-2.5212920	-1.6049710	1.3485700
38 O	-3.6198930	-2.3607540	-0.8423320
39 P	-2.2959020	-1.9964450	-0.2214720
40 O	-1.4456210	-0.9582270	-0.9097360
41 O	-1.4238820	-3.3290860	0.0868790
42 H	-1.8883950	-4.3955590	-1.6391390
43 C	-1.0171920	-4.0713680	-1.0614140
44 H	-0.4717610	-4.9447660	-0.7036270
45 H	-0.3625480	-3.4672870	-1.6976960

Table S40 - Cartesian coordinates (given in Angstroms) generated for structure **S** (Figure S221), calculated at M06-2X/6-31G level, after energy minimisation calculations.

Atom	X	Y	Z
1 C	-3.4902090	-1.6466640	-0.1270090
2 C	-2.1620980	-1.8456610	-0.4818740
3 C	-1.3274880	-0.7372150	-0.6973130
4 C	-1.8542280	0.5589150	-0.5488550
5 C	-3.1807460	0.7429510	-0.1961750
6 C	-4.0064010	-0.3610970	0.0152150
7 C	-5.4228600	-0.1705780	0.4499120
8 N	0.0088340	-0.8253990	-1.0697670
9 C	0.7922070	-1.9580600	-1.1428770
10 O	0.4148510	-3.0901770	-0.8593880
11 N	2.0570910	-1.6808560	-1.6074870
12 C	3.1297130	-2.6128070	-1.3303560
13 C	3.7411410	-2.3384220	0.0408710
14 O	2.8405650	-2.3933200	1.0073070
15 O	4.9092280	-2.0461230	0.2075520
16 F	-6.2266480	-1.1471180	-0.0062720
17 F	-5.9315600	0.9980400	0.0233920
18 F	-5.5536910	-0.1731720	1.7918860
19 H	-4.1336710	-2.5067970	0.0318220
20 H	-1.7651110	-2.8437540	-0.5959320
21 H	-1.2060940	1.4177720	-0.7130550
22 H	-3.5777720	1.7474310	-0.0891170
23 H	0.4926720	0.0606370	-1.2727370
24 H	2.2975620	-0.6927380	-1.7028060
25 H	3.9113190	-2.5233420	-2.0857330
26 H	2.7141130	-3.6228230	-1.3515580
27 H	3.1839300	-1.8844220	1.8403540
28 N	3.3357930	-0.7384480	2.9746840
29 H	3.1053020	-1.0948380	3.9001810
30 H	4.2676310	-0.3317230	3.0480410
31 C	2.3587310	0.2863440	2.5728810
32 C	2.7491730	0.8602150	1.2223240
33 O	1.7292730	1.7607060	0.8137360
34 P	1.9146150	2.3727240	-0.7005020
35 O	1.7876750	1.2377560	-1.6916120
36 O	0.5114000	3.1887880	-0.7795180
37 C	0.3623510	4.3208280	0.0738040
38 O	3.0953670	3.2843430	-0.7459000
39 H	2.2698830	1.0978080	3.3047060
40 H	1.3807360	-0.1955390	2.4781300
41 H	2.8395020	0.0518420	0.4874430
42 H	3.7134660	1.3825580	1.2852800
43 H	-0.5803450	4.8016040	-0.1905600
44 H	1.1884170	5.0221600	-0.0727760
45 H	0.3317090	4.0082110	1.1220730

Table S41 - Cartesian coordinates (given in Angstroms) generated for structure **T** (Figure S222), calculated at M06-2X/6-31G level, after energy minimisation calculations.

Atom	X	Y	Z
1 O	-3.7766150	0.8500320	-1.1784820
2 C	-3.6663020	2.0513820	-0.8124970
3 C	-2.3465680	2.7483860	-1.1909890
4 N	-1.3132690	1.7537800	-1.3980040
5 C	-0.0366810	1.9758190	-0.9783650
6 N	0.7075220	0.8111250	-0.9085860
7 C	2.0431750	0.6794150	-0.5569670
8 C	2.9457620	1.7521800	-0.4640710
9 C	4.2720970	1.5092910	-0.1300360
10 C	4.7220990	0.2134870	0.1093610
11 C	6.1374240	-0.0252430	0.5204510
12 F	6.9758640	0.8941730	0.0105580
13 F	6.3010160	0.0187140	1.8587310
14 F	6.5817060	-1.2324940	0.1282030
15 C	3.8293270	-0.8553620	0.0188030
16 C	2.5036390	-0.6285730	-0.3098900
17 O	0.3969570	3.0942450	-0.7004060
18 O	-4.5018040	2.7269810	-0.1709160
19 H	-2.0529470	3.4302200	-0.3890530
20 H	-2.5123330	3.3564470	-2.0912150
21 H	-1.6540850	0.7940910	-1.4770460
22 H	0.1753530	-0.0644720	-1.0333050
23 H	2.6000040	2.7579940	-0.6543610
24 H	4.9670560	2.3411070	-0.0662250
25 H	4.1743500	-1.8680750	0.2038170
26 H	1.8019730	-1.4598120	-0.3676730
27 C	-3.5649660	1.0878160	2.3239740
28 C	-3.5268250	-0.3858390	1.8555310
29 C	-2.1145950	-0.6741290	1.3623470
30 O	-1.9711070	-2.0413700	1.0014290
31 P	-1.1254070	-2.4681780	-0.3334660
32 O	0.1273670	-3.1866550	0.0517580
33 O	-2.1431880	-3.5777270	-0.9497890
34 C	-3.3977160	-3.0951870	-1.4275190
35 O	-1.0564910	-1.2835540	-1.2726700
36 O	-4.5027240	-0.6746920	0.8839510
37 H	-3.7541980	-1.0406460	2.7065640
38 H	-1.3887380	-0.4286970	2.1508280
39 H	-1.9118530	-0.0376420	0.4950180
40 H	-3.2536690	-2.4793630	-2.3207460
41 H	-3.8992960	-2.5009080	-0.6561940
42 H	-4.0034380	-3.9674810	-1.6795090
43 H	-2.9013840	1.6909870	1.6816480
44 H	-3.1640240	1.1495400	3.3426510
45 O	-4.8596850	1.6357080	2.3250360
46 H	-5.0115820	1.9243790	1.3993370
47 H	-4.3180480	-0.1172650	0.0860760

Table S42 - Cartesian coordinates (given in Angstroms) generated for structure **U** (Figure S222), calculated at M06-2X/6-31G level, after energy minimisation calculations.

Atom	X	Y	Z
1 O	4.0828140	-2.5197110	-0.1362980
2 C	2.8291720	-2.5378530	-0.2187050
3 C	2.1941930	-2.7863590	-1.6019360
4 N	1.1757500	-1.7872140	-1.8731000
5 C	-0.0854150	-1.9817290	-1.3613710
6 N	-0.7215840	-0.7840530	-1.0876100
7 C	-2.0305020	-0.5896990	-0.6669130
8 C	-3.0136380	-1.5918780	-0.6565890
9 C	-4.3037660	-1.2869510	-0.2382690
10 C	-4.6351810	0.0024920	0.1663500
11 C	-6.0070400	0.3186500	0.6635960
12 F	-6.9275920	-0.5528530	0.2187170
13 F	-6.0831610	0.2983100	2.0099630
14 F	-6.4072790	1.5474660	0.2876670
15 C	-3.6604140	1.0021710	0.1566960
16 C	-2.3710260	0.7131000	-0.2539940
17 O	-0.6009760	-3.0866650	-1.2180370
18 O	2.0195730	-2.3314400	0.7216480
19 H	1.7190840	-3.7706040	-1.6206100
20 H	2.9646040	-2.7499880	-2.3752400
21 H	1.5100220	-0.8274880	-1.7771830
22 H	-0.1130030	0.0446950	-1.0972690
23 H	-2.7592070	-2.5929710	-0.9731430
24 H	-5.0609500	-2.0649540	-0.2375890
25 H	-3.9149380	2.0112130	0.4674430
26 H	-1.6058100	1.4894160	-0.2567410
27 C	4.2629650	-0.5394670	2.7289120
28 C	4.0738790	0.5224720	1.6309200
29 C	2.6091660	0.9235160	1.5045760
30 O	2.5296360	2.1399640	0.7627550
31 P	1.4981730	2.3038630	-0.4884250
32 O	0.2273160	2.9686090	-0.0632460
33 O	2.3537830	3.3879690	-1.3487110
34 C	3.5558690	2.9099680	-1.9482450
35 O	1.4283660	1.0034650	-1.2574530
36 O	4.6145660	0.1370030	0.3814250
37 H	4.6338910	1.4201190	1.9251920
38 H	2.1739080	1.0889600	2.4971250
39 H	2.0538530	0.1233600	1.0029740
40 H	3.3265780	2.3121310	-2.8352770
41 H	4.1269700	2.2977910	-1.2421100
42 H	4.1429590	3.7830730	-2.2385070
43 H	4.4409900	-0.0294170	3.6828180
44 H	5.1775490	-1.1042350	2.4843790
45 O	3.1583600	-1.3860420	2.9399450
46 H	2.8642300	-1.7881140	2.0808990
47 H	4.4127170	-0.8141880	0.2306910

Table S43 - Cartesian coordinates (given in Angstroms) generated for structure **V** (Figure S222), calculated at M06-2X/6-31G level, after energy minimisation calculations.

Atom	X	Y	Z
1 C	3.2038190	-2.5900630	-0.1836890
2 C	2.3404100	-2.9957220	-1.3974560
3 N	1.3295560	-1.9716600	-1.6166400
4 C	0.0711180	-2.1186490	-1.1073950
5 N	-0.6399620	-0.9291660	-1.1300350
6 C	-1.9340130	-0.7017820	-0.6791830
7 C	-2.8418320	-1.7195450	-0.3444870
8 C	-4.1221510	-1.3882440	0.0821600
9 C	-4.5218240	-0.0583330	0.1749530
10 C	-5.8835150	0.2899100	0.6795420
11 F	-6.7695070	-0.7004420	0.4770510
12 F	-5.8933280	0.5416890	2.0045370
13 F	-6.3755380	1.3922850	0.0865460
14 C	-3.6240810	0.9566860	-0.1588810
15 C	-2.3438430	0.6415680	-0.5803030
16 O	-0.3911430	-3.1861500	-0.7104150
17 O	2.8230480	-2.9464540	0.9482310
18 O	4.1891630	-1.8428130	-0.4572610
19 H	1.8224850	-3.9371870	-1.2147200
20 H	2.9617640	-3.0799860	-2.2934110
21 H	1.6579280	-1.0149140	-1.7584920
22 H	-0.1031920	-0.1089710	-1.4295520
23 H	-2.5372910	-2.7528170	-0.4216370
24 H	-4.8202310	-2.1804930	0.3341480
25 H	-3.9288370	1.9966280	-0.0912880
26 H	-1.6407440	1.4355400	-0.8271860
27 C	3.9081740	-0.3771660	2.6643200
28 C	2.8979220	0.6828070	2.2316910
29 C	2.1896240	0.3024030	0.9443620
30 O	1.2402240	1.3388010	0.6455660
31 P	1.2789500	2.0337160	-0.8199050
32 O	0.1929870	3.0585780	-0.8769860
33 O	2.7500150	2.7435590	-0.6699680
34 C	3.2941720	3.3360270	-1.8455250
35 O	1.3607310	0.9670840	-1.8911140
36 O	3.5699440	1.9214120	2.0805270
37 H	2.1330920	0.7639430	3.0238550
38 H	1.6586420	-0.6486210	1.0563970
39 H	2.9217810	0.1951750	0.1370900
40 H	2.6385990	4.1323450	-2.2136800
41 H	3.4252400	2.5821040	-2.6271630
42 H	4.2622930	3.7607940	-1.5783470
43 H	3.3672380	-1.3183590	2.8360330
44 H	4.3410840	-0.0507180	3.6185780
45 O	4.9667150	-0.5495270	1.7574420
46 H	4.6472350	-1.0928510	0.9872510
47 H	3.1094870	2.4213180	1.3853920

Table S44 - Cartesian coordinates (given in Angstroms) generated for structure **W** (Figure S222), calculated at M06-2X/6-31G level, after energy minimisation calculations.

Atom		X	Y	Z
1	O	4.3861970	3.4550360	0.0306870
2	C	3.5725260	2.6730280	0.5478210
3	C	2.1227480	3.1763890	0.6860880
4	N	1.2003530	2.0812300	0.8890630
5	C	-0.1114620	2.2095000	0.5601730
6	N	-0.7991460	1.0102580	0.6079680
7	C	-2.1408400	0.7909170	0.3346660
8	C	-3.1009210	1.8086840	0.1961840
9	C	-4.4258700	1.4754550	-0.0528280
10	C	-4.8203150	0.1441130	-0.1639890
11	C	-6.2398360	-0.1828300	-0.4906700
12	F	-7.1030570	0.6692020	0.0920960
13	F	-6.4908520	-0.1200860	-1.8148060
14	F	-6.5804080	-1.4227220	-0.0994740
15	C	-3.8714700	-0.8697640	-0.0285620
16	C	-2.5461510	-0.5529400	0.2178840
17	O	-0.6199950	3.2890950	0.2515720
18	O	3.8007930	1.5061040	0.9899490
19	H	1.8545260	3.7316200	-0.2169780
20	H	2.0855860	3.8917450	1.5199710
21	H	1.6111780	1.1639070	1.0578960
22	H	-0.2296570	0.1725610	0.8130690
23	H	-2.7992610	2.8420610	0.2862070
24	H	-5.1643860	2.2655950	-0.1530840
25	H	-4.1703320	-1.9096930	-0.1154090
26	H	-1.8012780	-1.3424160	0.3074680
27	C	4.8195980	0.4755390	-1.8161420
28	C	3.8948330	-0.7425100	-1.9072960
29	C	2.7905940	-0.6461660	-0.8707160
30	O	1.8888580	-1.7458420	-1.0062560
31	P	1.1182320	-2.2092830	0.3471050
32	O	-0.0665940	-3.0325580	-0.0470070
33	O	2.2985170	-3.1487330	0.9842020
34	C	2.1114570	-3.5466290	2.3369270
35	O	0.9343630	-1.0196140	1.2666330
36	O	4.6737240	-1.9108270	-1.7069380
37	H	3.4542800	-0.8135140	-2.9091250
38	H	2.2352410	0.2935510	-0.9872350
39	H	3.2396980	-0.6360730	0.1260270
40	H	1.1825330	-4.1170160	2.4521380
41	H	2.0811940	-2.6737900	2.9957530
42	H	2.9550390	-4.1825240	2.6097500
43	H	4.2479370	1.4123220	-1.8636210
44	H	5.5134460	0.4512570	-2.6639870
45	O	5.5846800	0.4016570	-0.6291420
46	H	5.0329090	0.8358960	0.0797840
47	H	5.2845820	-1.6554680	-0.9916010

Table S45 - Cartesian coordinates (given in Angstroms) generated for structure **X** (Figure S222), calculated at M06-2X/6-31G level, after energy minimisation calculations.

Atom	X	Y	Z
1 O	-4.7621460	2.9608390	0.5096580
2 C	-3.8395770	2.5505330	-0.2137550
3 C	-2.4174550	3.0417960	0.1317170
4 N	-1.4262420	2.1266460	-0.3865160
5 C	-0.1182770	2.2235470	-0.0449890
6 N	0.6405850	1.1682220	-0.5244100
7 C	1.9959640	0.9446520	-0.3340660
8 C	2.8884000	1.8658690	0.2402710
9 C	4.2306750	1.5317020	0.3693420
10 C	4.7080450	0.2971260	-0.0650040
11 C	6.1439330	-0.0554970	0.1419160
12 F	6.9571430	0.9996070	-0.0465200
13 F	6.3904820	-0.4948730	1.3928290
14 F	6.5541490	-1.0284140	-0.6894650
15 C	3.8280240	-0.6186820	-0.6429500
16 C	2.4896530	-0.2956150	-0.7803810
17 O	0.3305650	3.1589830	0.6190480
18 O	-3.9271150	1.7720940	-1.2096310
19 H	-2.3297990	3.1307020	1.2190430
20 H	-2.2813620	4.0493110	-0.2851930
21 H	-1.7751780	1.3527730	-0.9442230
22 H	0.1106890	0.3705870	-0.9090520
23 H	2.5221410	2.8231530	0.5808990
24 H	4.9171430	2.2490650	0.8096880
25 H	4.1912700	-1.5803150	-0.9915910
26 H	1.7923000	-0.9962490	-1.2301610
27 C	-5.6212510	-0.5127740	0.6539050
28 C	-4.3667360	-1.3319400	0.9632900
29 C	-3.1254220	-0.5161000	0.6465410
30 O	-1.9290430	-1.2212010	0.9928340
31 P	-1.1391120	-1.9729270	-0.2175320
32 O	-0.9150200	-1.0052530	-1.3584710
33 O	0.2433000	-2.1939830	0.6110950
34 C	1.0665180	-3.2813180	0.2017060
35 O	-1.7902050	-3.2967660	-0.5141980
36 O	-4.4188110	-2.5261200	0.2103820
37 H	-4.3661840	-1.5540250	2.0452250
38 H	-3.1315030	0.4149310	1.2248940
39 H	-3.1237230	-0.2591500	-0.4153420
40 H	2.0011450	-3.2008880	0.7588780
41 H	1.2901510	-3.2350340	-0.8706950
42 H	0.5811040	-4.2360350	0.4211330
43 H	-5.6268950	0.3781810	1.3003080
44 H	-6.4932220	-1.1233570	0.9166870
45 O	-5.7461690	-0.1620580	-0.7038410
46 H	-5.1490260	0.6065230	-0.8866610
47 H	-3.5165260	-2.8992990	0.1237700

Table S46 - Cartesian coordinates (given in Angstroms) generated for structure **Y** (Figure S222), calculated at M06-2X/6-31G level, after energy minimisation calculations.

Atom	X	Y	Z
1 O	3.8951440	1.4290000	-0.6196770
2 C	3.7093130	2.6648050	-0.3654780
3 C	2.2494080	3.0900080	-0.1253740
4 N	1.3370310	1.9854830	-0.2975240
5 C	0.0024840	2.1515250	-0.1333220
6 N	-0.7205570	1.0050800	-0.4304040
7 C	-2.0771030	0.7820940	-0.2558220
8 C	-2.9889600	1.7453980	0.2088080
9 C	-4.3312050	1.4142150	0.3512920
10 C	-4.7901820	0.1379520	0.0393560
11 C	-6.2412700	-0.1965660	0.1456400
12 F	-6.8789670	0.5756400	1.0418670
13 F	-6.8949790	-0.0382720	-1.0237070
14 F	-6.4366910	-1.4761420	0.5114950
15 C	-3.8902680	-0.8230050	-0.4249900
16 C	-2.5516030	-0.5067650	-0.5703570
17 O	-0.5028180	3.2125100	0.2382960
18 O	4.5675500	3.5504480	-0.2899120
19 H	2.0139290	3.9123940	-0.8126790
20 H	2.1769130	3.5034070	0.8879030
21 H	1.7170900	1.0922350	-0.6166110
22 H	-0.1530160	0.2064730	-0.7417410
23 H	-2.6359350	2.7355000	0.4564490
24 H	-5.0266080	2.1630110	0.7178490
25 H	-4.2374850	-1.8234780	-0.6643780
26 H	-1.8492760	-1.2601380	-0.9158080
27 C	5.7409150	-0.9515600	0.8091060
28 C	4.2895860	-1.3763540	1.0494770
29 C	3.7208140	-2.0726020	-0.1788250
30 O	2.4602350	-2.6608410	0.1337390
31 P	1.0912330	-2.1639530	-0.6034110
32 O	0.5372230	-3.2483550	-1.4699530
33 O	0.0954600	-2.0554020	0.6816370
34 C	0.4593770	-1.1885690	1.7512580
35 O	1.3122960	-0.7821250	-1.1810820
36 O	3.4695230	-0.2965220	1.4464210
37 H	4.2805540	-2.0870070	1.8870360
38 H	4.3963340	-2.8711340	-0.5085080
39 H	3.6120080	-1.3434650	-0.9861280
40 H	0.8587800	-0.2430060	1.3749160
41 H	1.2235320	-1.6580290	2.3771240
42 H	-0.4457120	-1.0039980	2.3349330
43 H	6.3794710	-1.8430290	0.8048640
44 H	6.0507370	-0.3248980	1.6601430
45 O	5.9507210	-0.2983090	-0.4249450
46 H	5.3242710	0.4621090	-0.4859870
47 H	3.4358360	0.3306820	0.6895870

Table S47 - Cartesian coordinates (given in Angstroms) generated for structure **Z** (Figure S222), calculated at M06-2X/6-31G level, after energy minimisation calculations.

Atom		X	Y	Z
1	O	2.3712590	-2.1842030	-0.1821660
2	C	3.1222560	-2.3572620	-1.1885200
3	C	2.6774090	-1.6721360	-2.5045270
4	N	1.7165710	-0.6051810	-2.3284520
5	C	0.4146400	-0.9444480	-2.0898360
6	N	-0.3143150	0.0763470	-1.4987190
7	C	-1.6412110	0.0344660	-1.0885890
8	C	-2.5468870	-0.9899230	-1.4111430
9	C	-3.8579050	-0.9200750	-0.9541050
10	C	-4.2917170	0.1557660	-0.1853590
11	C	-5.6859500	0.1978780	0.3465670
12	F	-6.5380370	-0.5314100	-0.3934050
13	F	-5.7722300	-0.2800440	1.6051960
14	F	-6.1711030	1.4522230	0.3914390
15	C	-3.3973080	1.1785780	0.1356270
16	C	-2.0885010	1.1203320	-0.3088950
17	O	-0.0734100	-2.0245720	-2.4185660
18	O	4.1663420	-3.0291730	-1.2458470
19	H	2.2467650	-2.4314690	-3.1629900
20	H	3.5666390	-1.2724220	-2.9989600
21	H	2.1037740	0.1951590	-1.8192170
22	H	0.2008490	0.9106510	-1.1822420
23	H	-2.2182560	-1.8247890	-2.0117710
24	H	-4.5522290	-1.7142370	-1.2106720
25	H	-3.7280400	2.0239200	0.7315660
26	H	-1.3811470	1.9087740	-0.0652890
27	C	0.8399370	-1.8704930	2.8395470
28	C	1.8875670	-0.7801590	2.6061180
29	C	1.3976860	0.2179110	1.5747960
30	O	2.2960330	1.3304240	1.5264520
31	P	2.3420120	2.0973300	0.0925160
32	O	0.9327430	2.3757100	-0.3784310
33	O	3.0343620	3.4542080	0.6632350
34	C	3.3822430	4.4197220	-0.3240240
35	O	3.2628110	1.4008070	-0.8732320
36	O	3.1040470	-1.3965880	2.2279350
37	H	2.0823000	-0.2617290	3.5540430
38	H	0.3909120	0.5753770	1.8295860
39	H	1.3575130	-0.2931410	0.6052720
40	H	2.4939800	4.7445580	-0.8763780
41	H	4.1139190	4.0084970	-1.0261060
42	H	3.8156880	5.2743550	0.1966450
43	H	0.5327210	-2.2791380	1.8630640
44	H	-0.0466240	-1.4685310	3.3405400
45	O	1.3893070	-2.8822820	3.6607530
46	H	2.3151490	-2.9401000	3.3629370
47	H	2.9509390	-1.7763620	1.3163420

Table S48 - Cartesian coordinates (given in Angstroms) generated for structure **AA** (Figure S222), calculated at M06-2X/6-31G level, after energy minimisation calculations.

Atom	X	Y	Z
1 O	4.0671950	-2.0890920	-0.8892910
2 C	2.8176260	-2.1272730	-0.9549990
3 C	2.1593620	-1.8880110	-2.3301380
4 N	1.1228540	-0.8786340	-2.2296360
5 C	-0.1283710	-1.2457940	-1.8215670
6 N	-0.8051180	-0.2151530	-1.1882840
7 C	-2.1185880	-0.2276490	-0.7374950
8 C	-3.0676080	-1.2075360	-1.0726440
9 C	-4.3634890	-1.1089770	-0.5799930
10 C	-4.7386000	-0.0470740	0.2382250
11 C	-6.1166310	0.0206970	0.8086740
12 F	-7.0143820	-0.6325520	0.0516940
13 F	-6.1919460	-0.5234140	2.0407860
14 F	-6.5483780	1.2886660	0.9349540
15 C	-3.8011940	0.9322460	0.5708090
16 C	-2.5070590	0.8448140	0.0894400
17 O	-0.6185280	-2.3526230	-2.0379410
18 O	2.0169850	-2.3177000	0.0039780
19 H	1.7037450	-2.8152750	-2.6873570
20 H	2.9168800	-1.5637160	-3.0469980
21 H	1.4882480	0.0273260	-1.9161580
22 H	-0.2405520	0.5775280	-0.8464290
23 H	-2.7815340	-2.0333010	-1.7071160
24 H	-5.0924420	-1.8685900	-0.8454550
25 H	-4.0875780	1.7667690	1.2036210
26 H	-1.7660600	1.5984310	0.3418930
27 C	3.6835400	-1.5168190	2.9970910
28 C	3.9255330	-0.2276900	2.1985980
29 C	2.6281920	0.3666340	1.6600070
30 O	2.9030210	1.6675060	1.1302740
31 P	2.0924110	2.0631040	-0.2181200
32 O	0.6034910	1.9267620	0.0026130
33 O	2.5254880	3.6322370	-0.1927030
34 C	2.0232660	4.4170770	-1.2689190
35 O	2.6730410	1.3766240	-1.4264410
36 O	4.8783010	-0.4280490	1.1817390
37 H	4.3516510	0.5167600	2.8852800
38 H	1.8961850	0.4687490	2.4709190
39 H	2.2039640	-0.2933950	0.8939070
40 H	0.9282550	4.4201470	-1.2663670
41 H	2.3815530	4.0316780	-2.2288220
42 H	2.3890730	5.4346940	-1.1268910
43 H	2.8244930	-1.3677150	3.6735130
44 H	4.5618170	-1.6968390	3.6277210
45 O	3.5318980	-2.6589850	2.1911090
46 H	2.9142950	-2.4840660	1.4314370
47 H	4.5670450	-1.1648240	0.6044060

Table S49 - Cartesian coordinates (given in Angstroms) generated for structure **AB** (Figure S222), calculated at M06-2X/6-31G level, after energy minimisation calculations.

Atom	X	Y	Z
1 O	3.9647360	-3.4090940	-0.9706170
2 C	2.9031200	-2.7667860	-0.8663970
3 C	2.3024380	-2.2275820	-2.1873430
4 N	1.3421460	-1.1599320	-2.0290190
5 C	0.0811730	-1.4602670	-1.6151160
6 N	-0.6190250	-0.3577950	-1.1447310
7 C	-1.9434240	-0.3132030	-0.7276330
8 C	-2.8428380	-1.3913880	-0.7995830
9 C	-4.1501450	-1.2286020	-0.3583620
10 C	-4.5876630	-0.0097770	0.1536380
11 C	-5.9791710	0.1255240	0.6768000
12 F	-6.8540670	-0.6348230	-0.0053520
13 F	-6.0849190	-0.2509150	1.9677020
14 F	-6.4229570	1.3934490	0.6173530
15 C	-3.7007060	1.0648930	0.2249460
16 C	-2.3954740	0.9173630	-0.2109090
17 O	-0.4062490	-2.5873920	-1.6972930
18 O	2.2577400	-2.5218110	0.1938910
19 H	1.8230390	-3.0609370	-2.7093160
20 H	3.1225940	-1.8726940	-2.8168710
21 H	1.7445870	-0.2463590	-1.8006780
22 H	-0.1128200	0.5339250	-1.0510530
23 H	-2.5096160	-2.3378730	-1.1979190
24 H	-4.8400630	-2.0648580	-0.4225030
25 H	-4.0351190	2.0204300	0.6167390
26 H	-1.6955950	1.7467630	-0.1618930
27 C	2.8096310	-1.3799880	3.0443370
28 C	2.7403730	0.0960390	2.6692360
29 C	2.5339730	0.2964670	1.1814940
30 O	2.5448970	1.7131570	0.9488510
31 P	2.0809390	2.1460820	-0.5534640
32 O	0.5741980	2.1823620	-0.6428610
33 O	2.6750960	3.6560670	-0.4721480
34 C	2.4411010	4.4650830	-1.6215130
35 O	2.8154020	1.3199830	-1.5743210
36 O	3.9474640	0.7246850	3.0709890
37 H	1.8862030	0.5453510	3.2032730
38 H	1.5889520	-0.1512290	0.8496100
39 H	3.3476590	-0.1734000	0.6224340
40 H	1.3679850	4.6120340	-1.7790690
41 H	2.8788820	4.0042130	-2.5130560
42 H	2.9176370	5.4291740	-1.4414310
43 H	1.8035440	-1.8090160	2.9221480
44 H	3.0683890	-1.4387550	4.1091530
45 O	3.7652780	-2.1058540	2.3134730
46 H	3.3026960	-2.3792090	1.4736440
47 H	3.9397810	1.6024270	2.6563700

Table S50 - Cartesian coordinates (given in Angstroms) generated for structure **AC** (Figure S222), calculated at M06-2X/6-31G level, after energy minimisation calculations.

Atom	X	Y	Z
1 O	4.2523900	-3.6949370	-0.0126030
2 C	3.4702420	-2.7526330	-0.2129030
3 C	1.9796270	-3.1084840	-0.3576820
4 N	1.1402110	-1.9422790	-0.2105110
5 C	-0.2060880	-2.0789530	-0.1758840
6 N	-0.8745950	-0.8719540	-0.0053340
7 C	-2.2482830	-0.6807000	0.0379450
8 C	-3.1953650	-1.6264520	-0.3919780
9 C	-4.5495540	-1.3222880	-0.3363970
10 C	-4.9868770	-0.0865080	0.1341860
11 C	-6.4469950	0.2023440	0.2455110
12 F	-7.1688670	-0.4624180	-0.6735260
13 F	-6.9523840	-0.1493890	1.4458400
14 F	-6.7180860	1.5112540	0.0954960
15 C	-4.0519760	0.8592000	0.5571210
16 C	-2.6995180	0.5682160	0.5099020
17 O	-0.7783980	-3.1686770	-0.2562480
18 O	3.7638910	-1.5243400	-0.3343260
19 H	1.7230620	-3.8573490	0.3973960
20 H	1.8368540	-3.5923350	-1.3357480
21 H	1.5661200	-1.0280720	-0.4060250
22 H	-0.3155000	-0.0635410	0.2979950
23 H	-2.8620210	-2.5852180	-0.7606090
24 H	-5.2736100	-2.0568090	-0.6759390
25 H	-4.3842900	1.8262830	0.9218940
26 H	-1.9606420	1.2937190	0.8399650
27 C	6.1099250	0.6474710	0.7848210
28 C	4.9737600	1.4803030	0.1904420
29 C	3.7569520	1.4759400	1.1040600
30 O	2.7856290	2.4190420	0.6405360
31 P	1.4479850	1.8233970	-0.0620390
32 O	0.3967680	1.4988200	0.9734920
33 O	1.0564680	3.1982040	-0.8430200
34 C	-0.1011730	3.1013210	-1.6673360
35 O	1.7855910	0.7354570	-1.0462950
36 O	4.6357910	1.0489250	-1.1137860
37 H	5.3314350	2.5159670	0.0980410
38 H	4.0427850	1.7677640	2.1195270
39 H	3.3413480	0.4617230	1.1367300
40 H	-0.9747890	2.7997390	-1.0783420
41 H	0.0588190	2.3743590	-2.4699590
42 H	-0.2774220	4.0877630	-2.0976780
43 H	6.5326320	1.1789970	1.6475360
44 H	6.9006820	0.5815010	0.0197290
45 O	5.7080590	-0.6251600	1.2338180
46 H	5.1069440	-1.0254870	0.5509160
47 H	3.9049830	0.4033180	-1.0376200

3D visualisations – HF/3-21G level

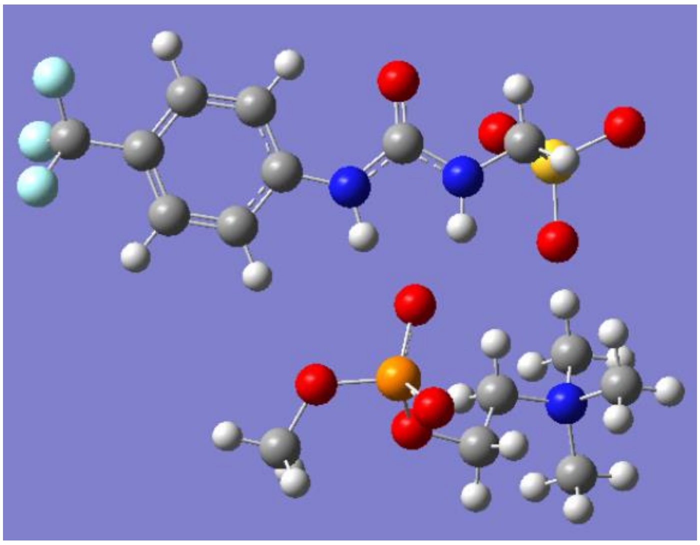

Figure S224 - Output file generated for conformation **A** (Figure S217) calculated at HF/3-21G level.

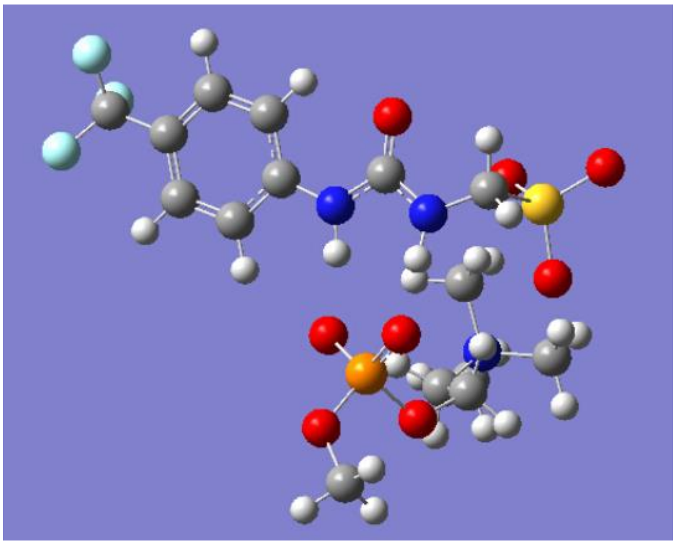

Figure S225 - Output file generated for conformation **B** (Figure S217) calculated at HF/3-21G level.

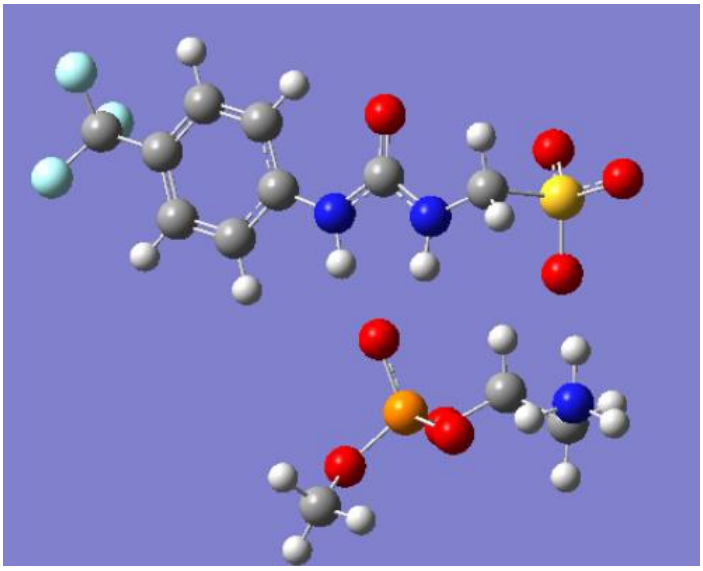

Figure S226 - Output file generated for conformation **C** (Figure S218) calculated at HF/3-21G level.

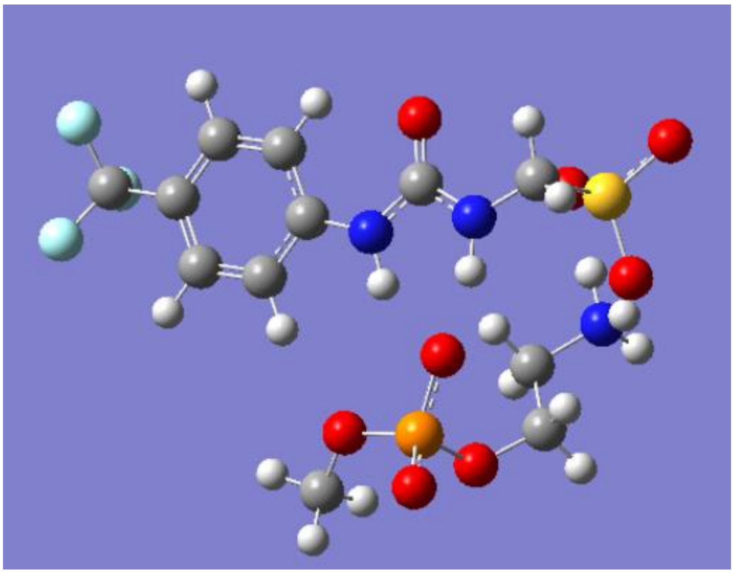

Figure S227 - Output file generated for conformation **D** (Figure S211) calculated at HF/3-21G level.

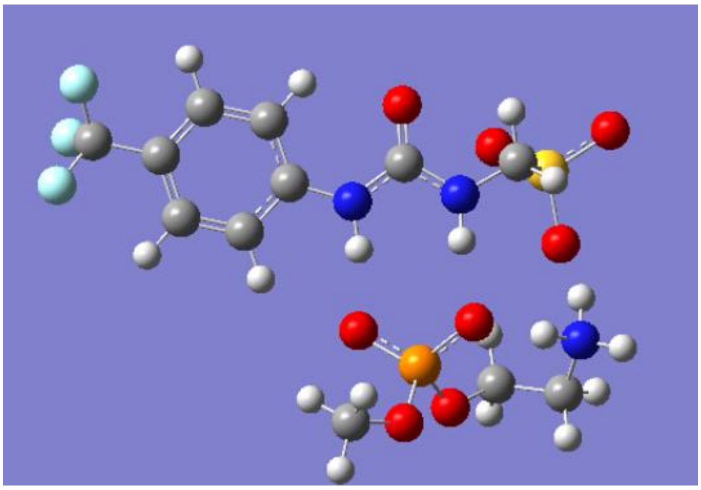

Figure S228 - Output file generated for conformation **E** (Figure S218) calculated at HF/3-21G level.

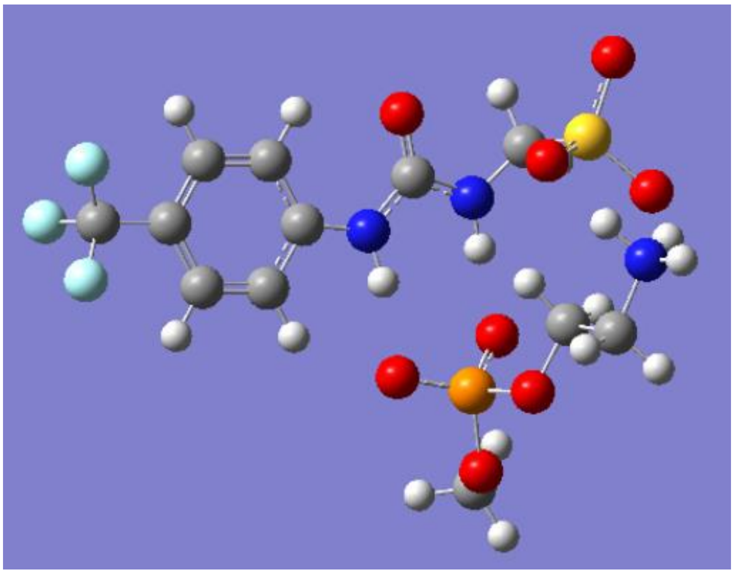

Figure S229 - Output file generated for conformation **F** (Figure S218) calculated at HF/3-21G level.

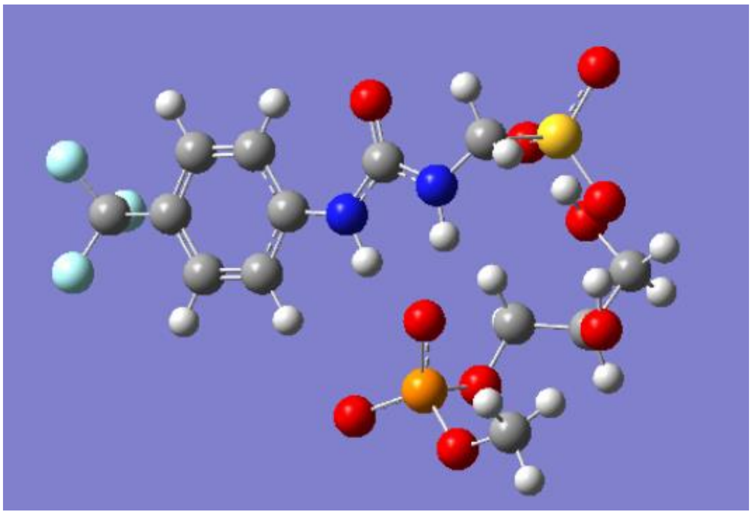

Figure S230 - Output file generated for conformation **G** (Figure S219) calculated at HF/3-21G level.

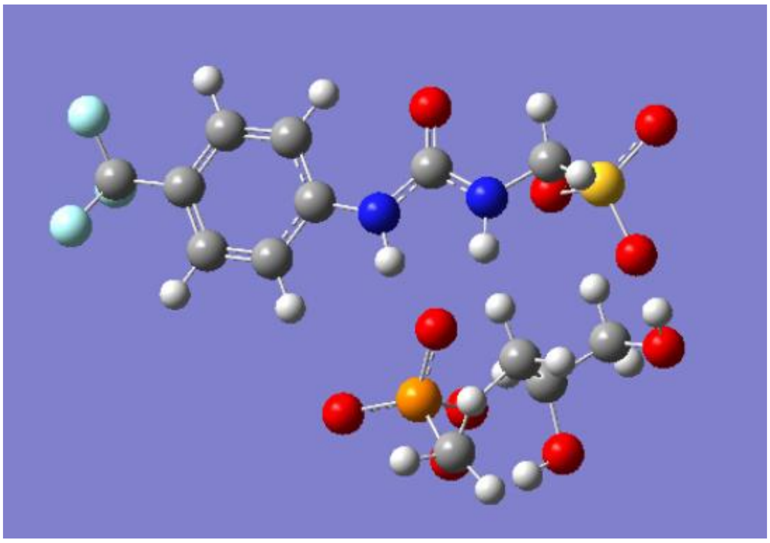

Figure S231 - Output file generated for conformation **H** (Figure S219) calculated at HF/3-21G level.

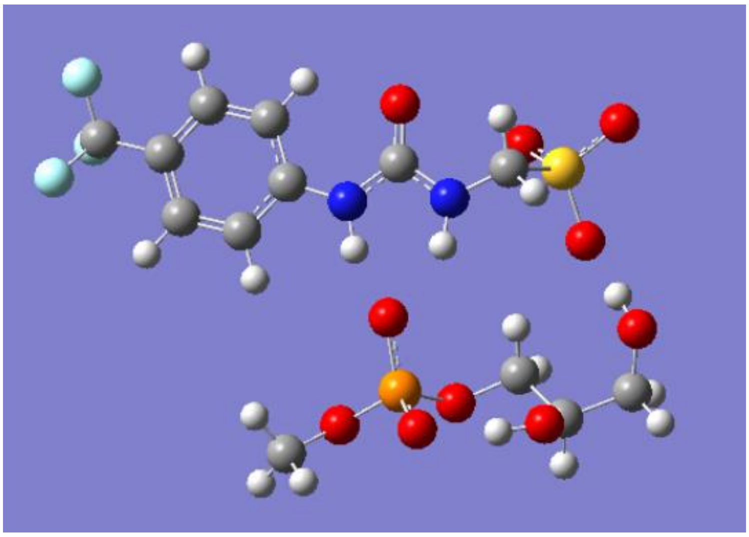

Figure S232 - Output file generated for conformation **I** (Figure S219) calculated at HF/3-21G level.

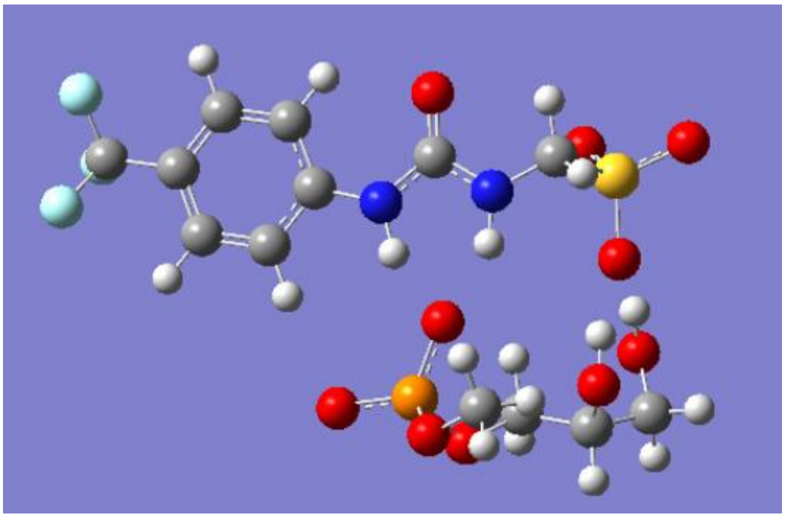

Figure S233 - Output file generated for conformation **J** (Figure S219) calculated at HF/3-21G level.

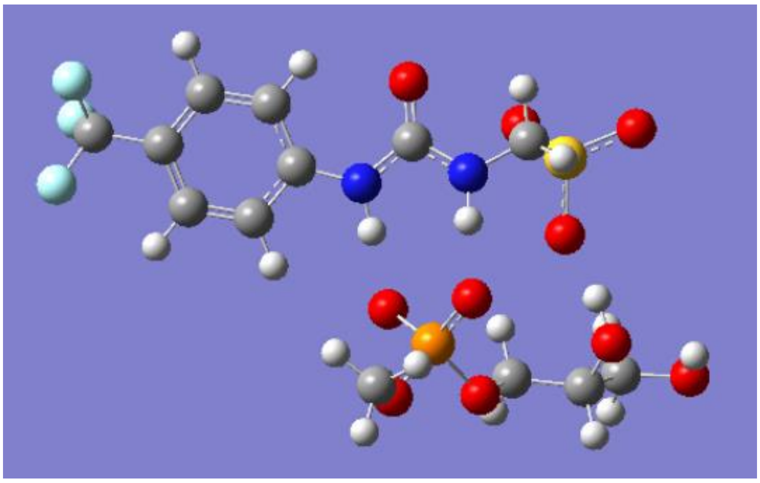

Figure S234 - Output file generated for conformation **K** (Figure S219) calculated at HF/3-21G level.

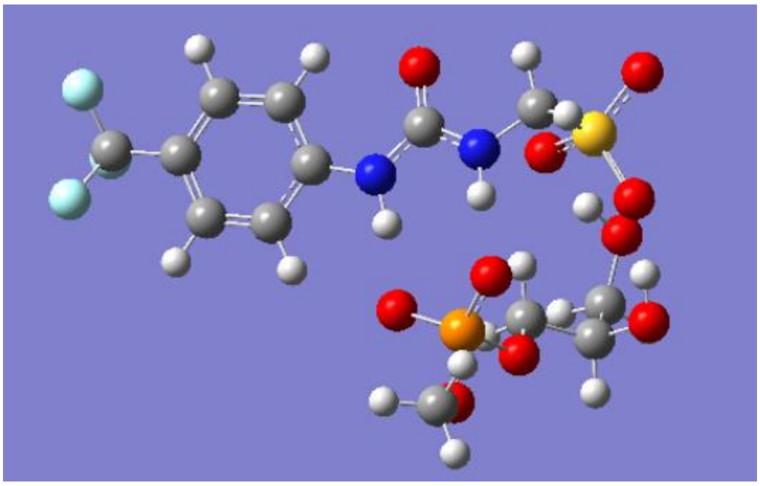

Figure S235 - Output file generated for conformation **L** (Figure S219) calculated at HF/3-21G level.

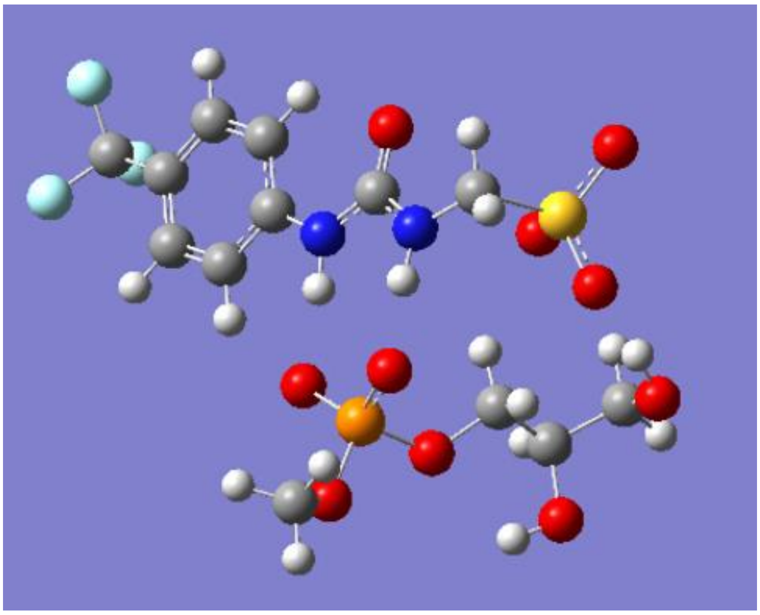

Figure S236 - Output file generated for conformation **M** (Figure S219) calculated at HF/3-21G level.

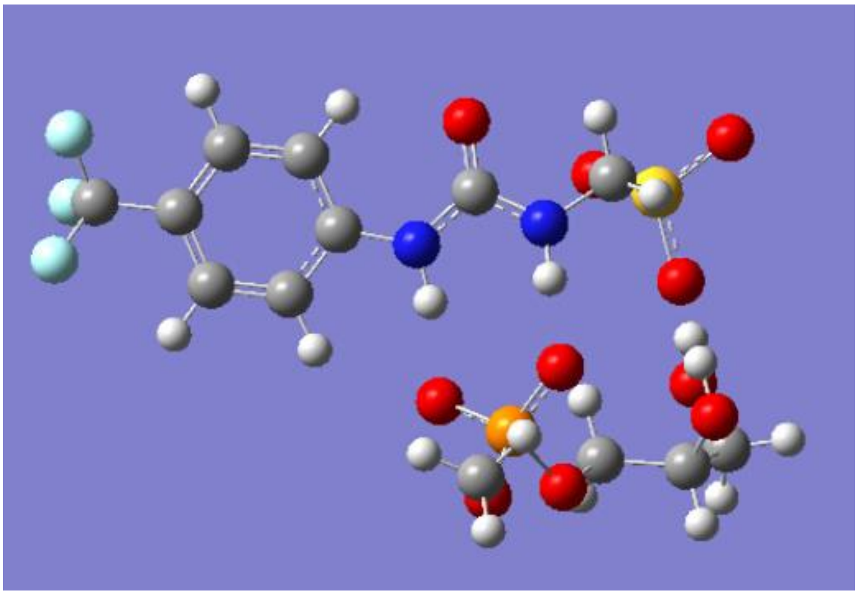

Figure S237 - Output file generated for conformation **N** (Figure S219) calculated at HF/3-21G level.

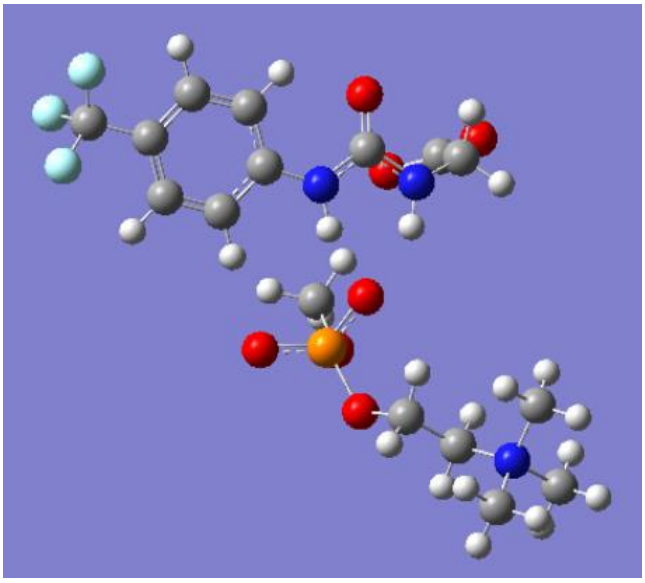

Figure S238 - Output file generated for conformation **O** (Figure S219) calculated at HF/3-21G level.

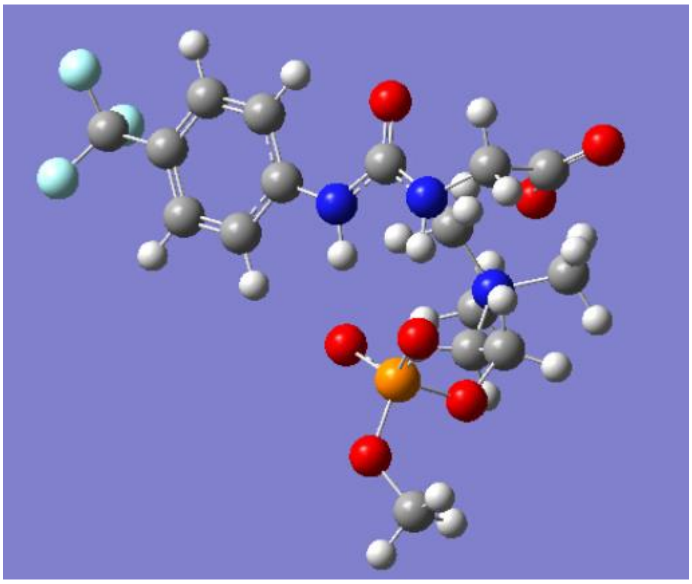

Figure S239 - Output file generated for conformation **P** (Figure S218) calculated at HF/3-21G level.

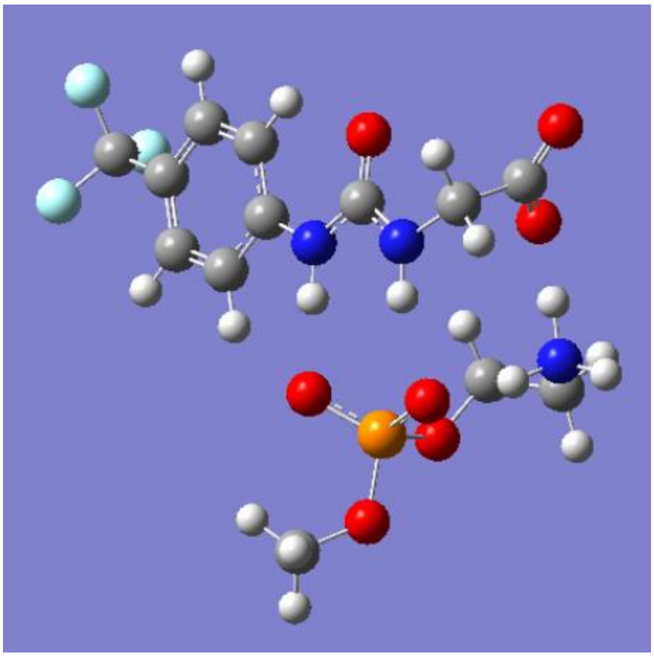

Figure S240 - Output file generated for conformation **Q** (Figure S221) calculated at HF/3-21G level.

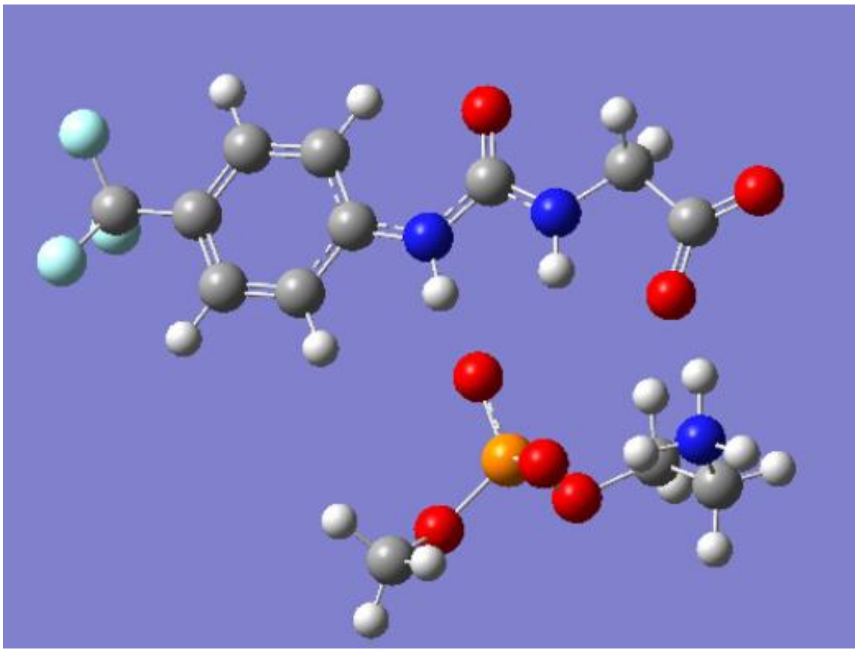

Figure S241 - Output file generated for conformation **R** (Figure S221) calculated at HF/3-21G level.

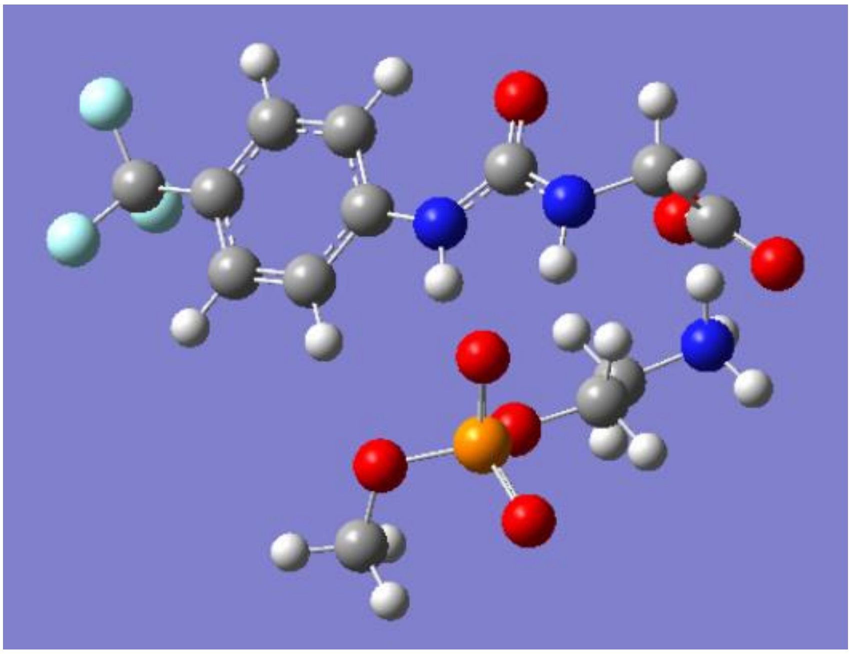

Figure S242 - Output file generated for conformation **S** (Figure S221) calculated at HF/3-21G level.

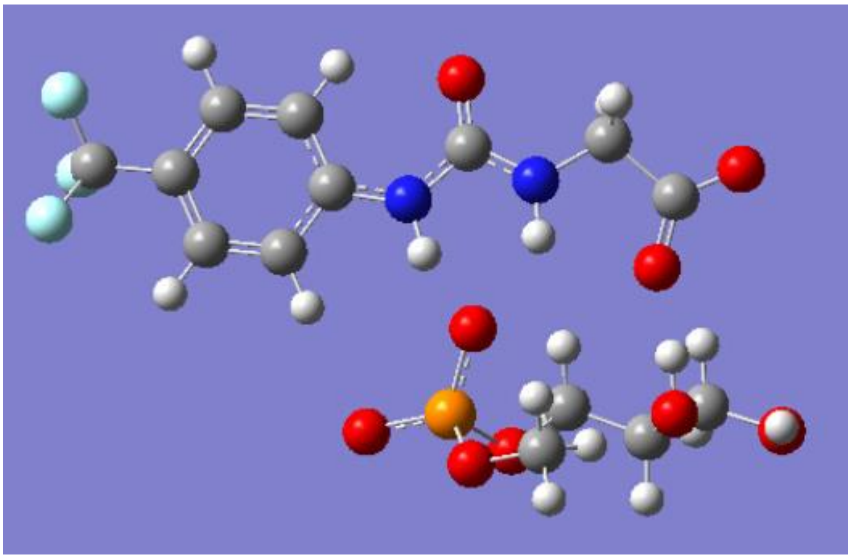

Figure S243 - Output file generated for conformation **T** (Figure S222) calculated at HF/3-21G level.

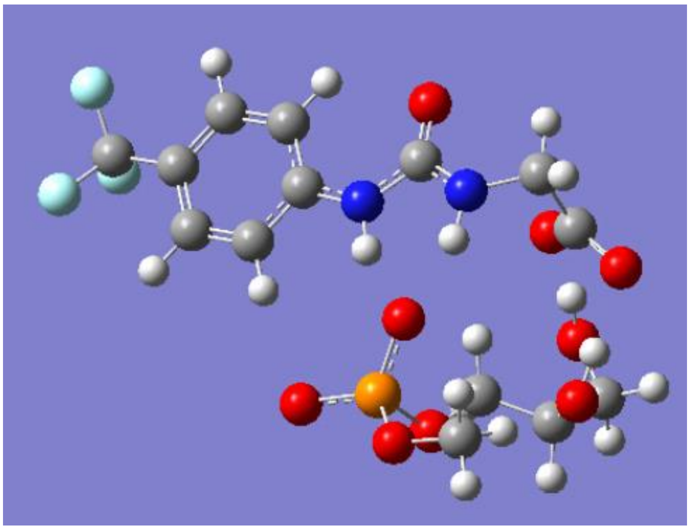

Figure S244 - Output file generated for conformation **U** (Figure S222) calculated at HF/3-21G level.

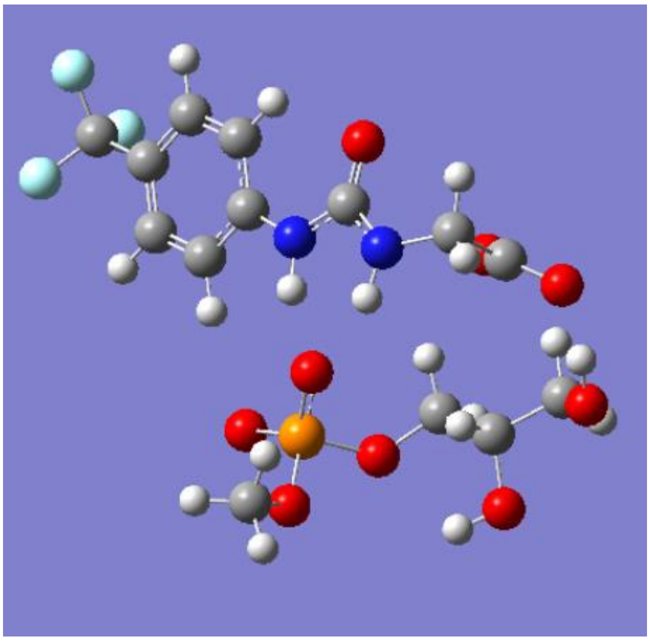

Figure S245 - Output file generated for conformation **V** (Figure S222) calculated at HF/3-21G level.

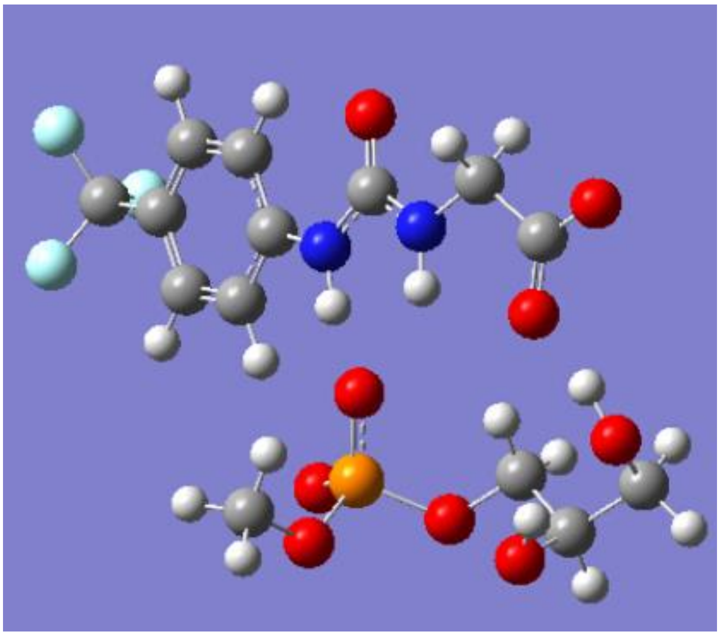

Figure S246 - Output file generated for conformation **W** (Figure S222) calculated at HF/3-21G level.

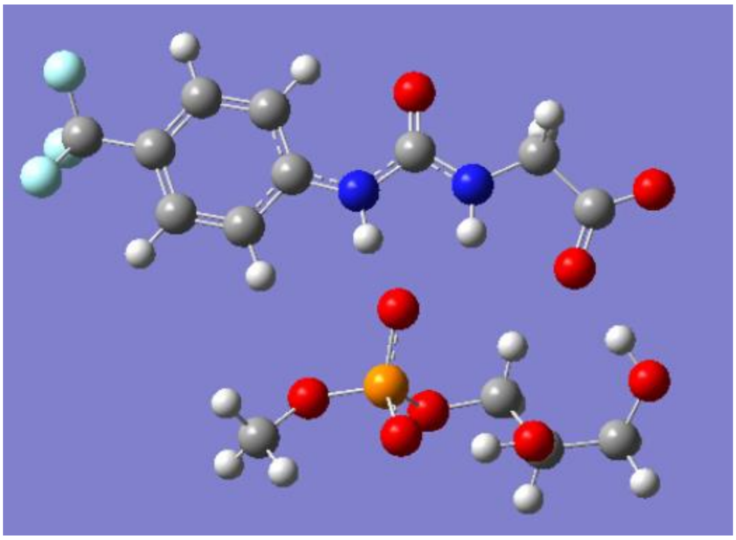

Figure S247 - Output file generated for conformation **X** (Figure S222) calculated at HF/3-21G level.

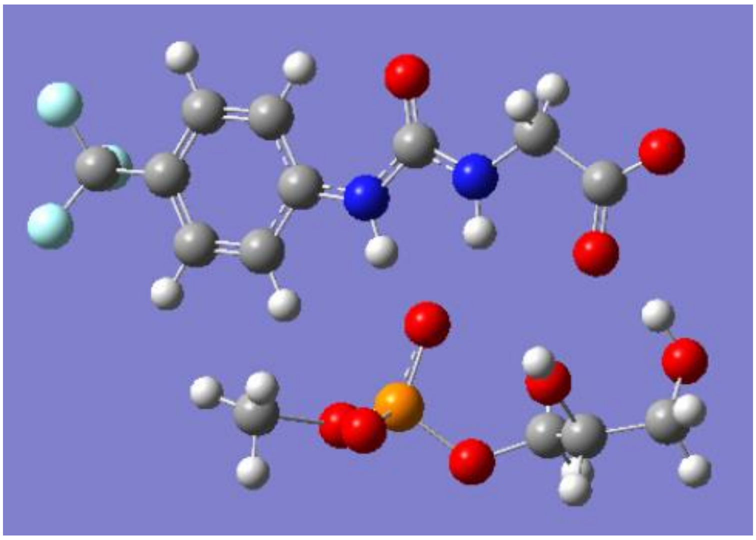

Figure S248 - Output file generated for conformation **Y** (Figure S222) calculated at HF/3-21G level.

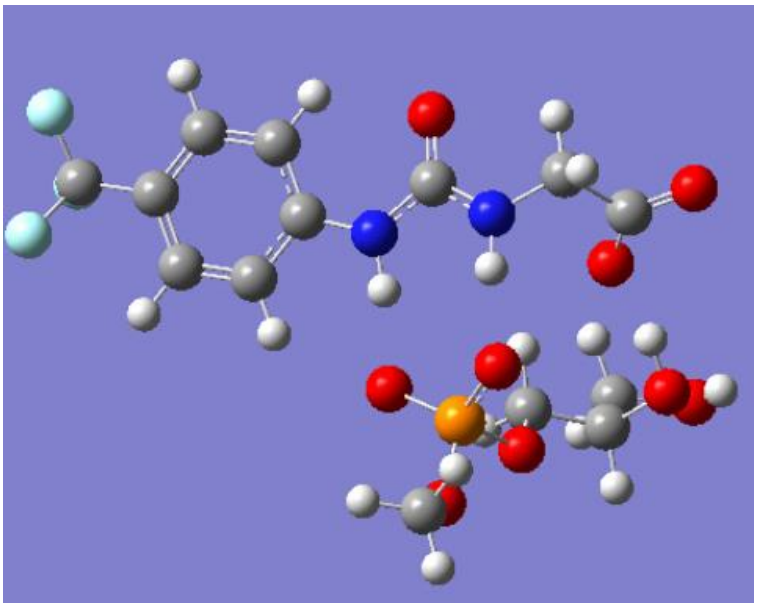

Figure S249 - Output file generated for conformation **Z** (Figure S222) calculated at HF/3-21G level.

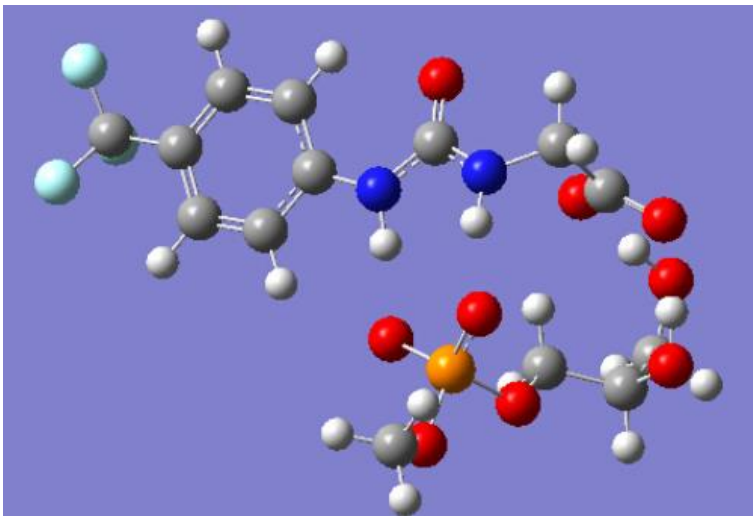

Figure S250 - Output file generated for conformation **AA** (Figure S222) calculated at HF/3-21G level.

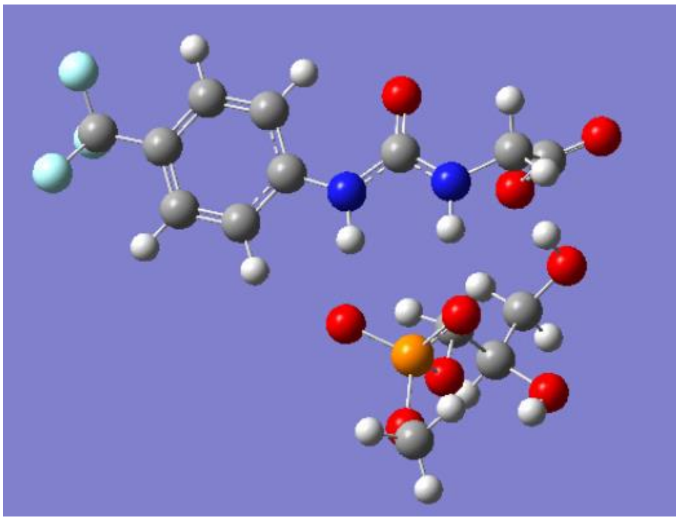

Figure S251 - Output file generated for conformation **AB** (Figure S222) calculated at HF/3-21G level.

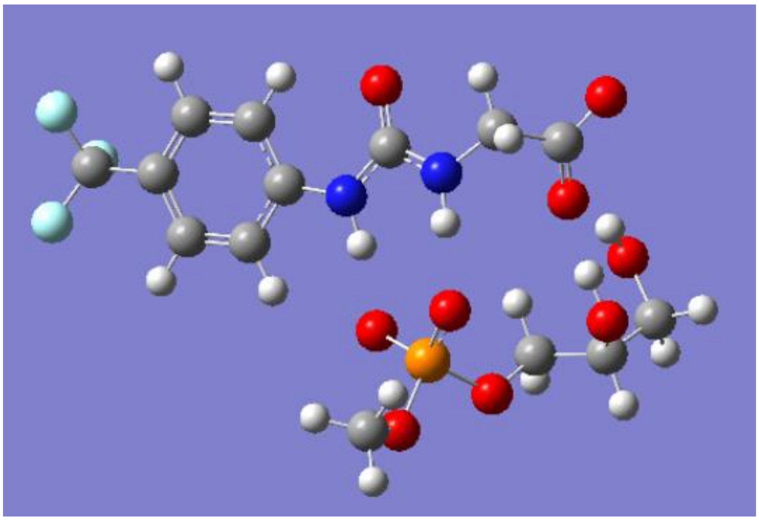

Figure S252 - Output file generated for conformation **AC** (Figure S222) calculated at HF/3-21G level.

3D visualisations – M06-2X/6-31G level

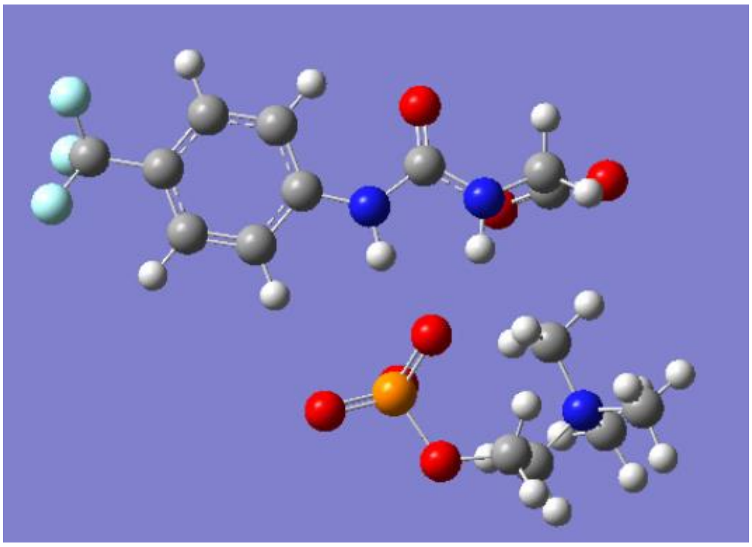

Figure S253 - Output file generated for conformation **O** (Figure S220) calculated at M06-2X/6-31G level.

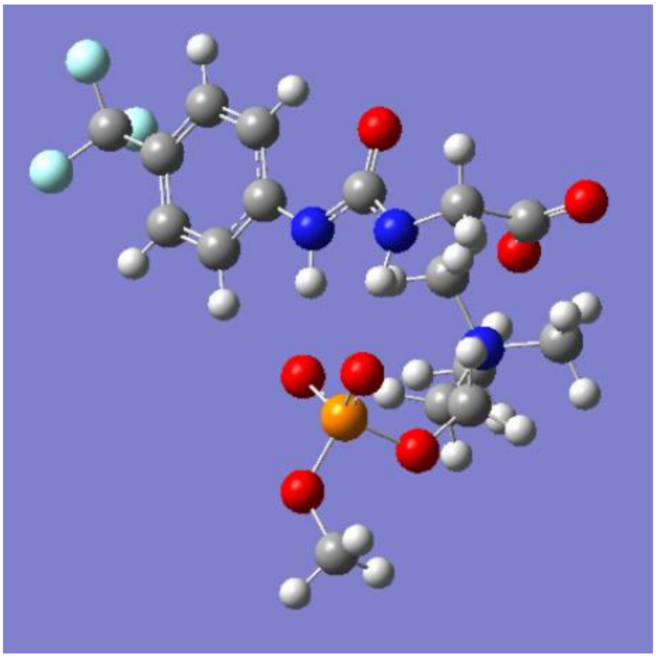

Figure S254 - Output file generated for conformation **P** (Figure S220) calculated at M06-2X/6-31G level.

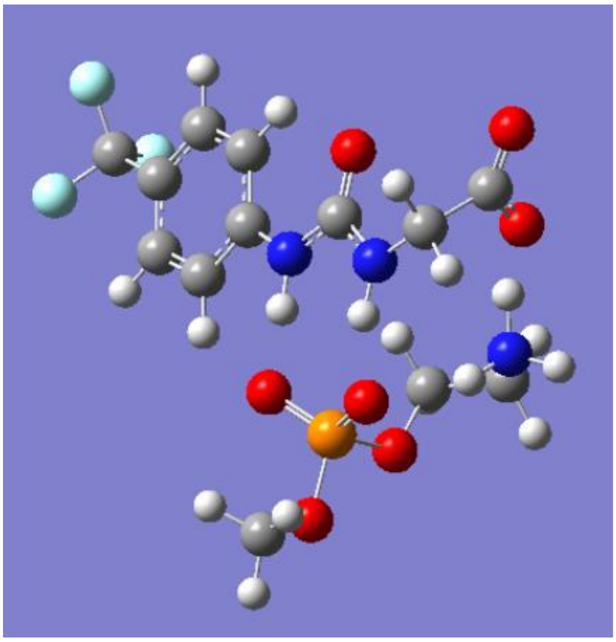

Figure S255 - Output file generated for conformation **Q** (Figure S221) calculated at M06-2X/6-31G level.

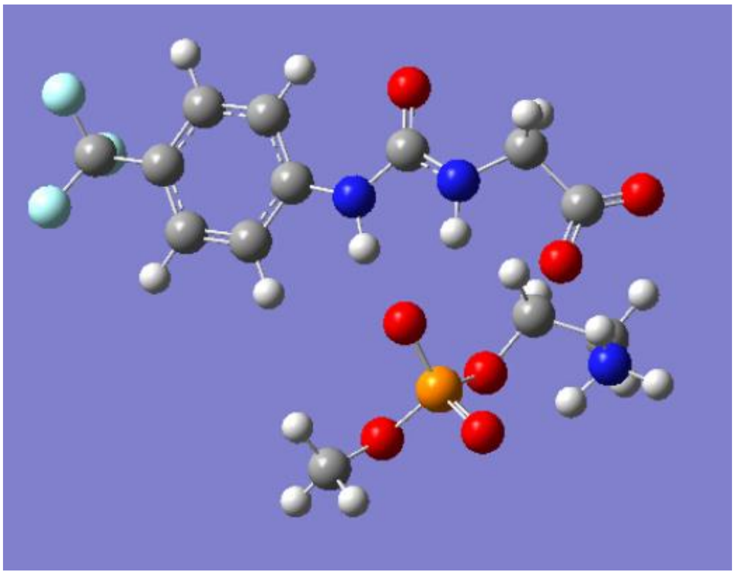

Figure S256 - Output file generated for conformation **R** (Figure S221) calculated at M06-2X/6-31G level.

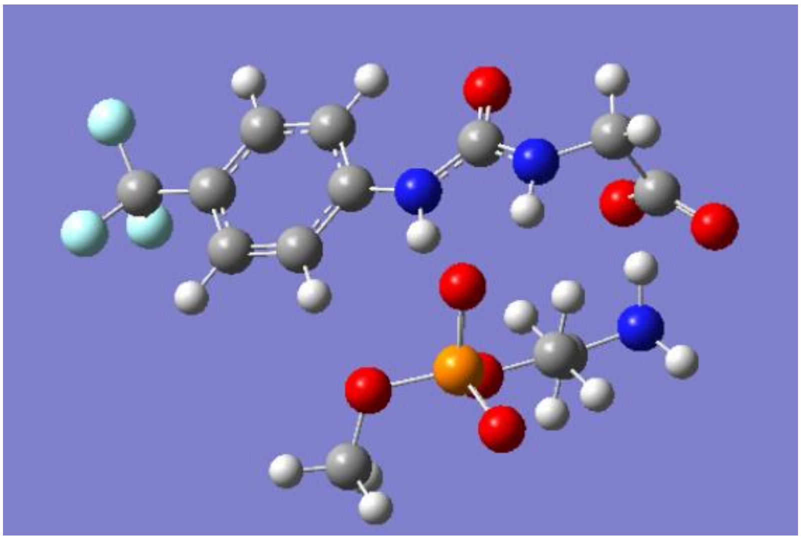

Figure S257 - Output file generated for conformation **S** (Figure S221) calculated at M06-2X/6-31G level.

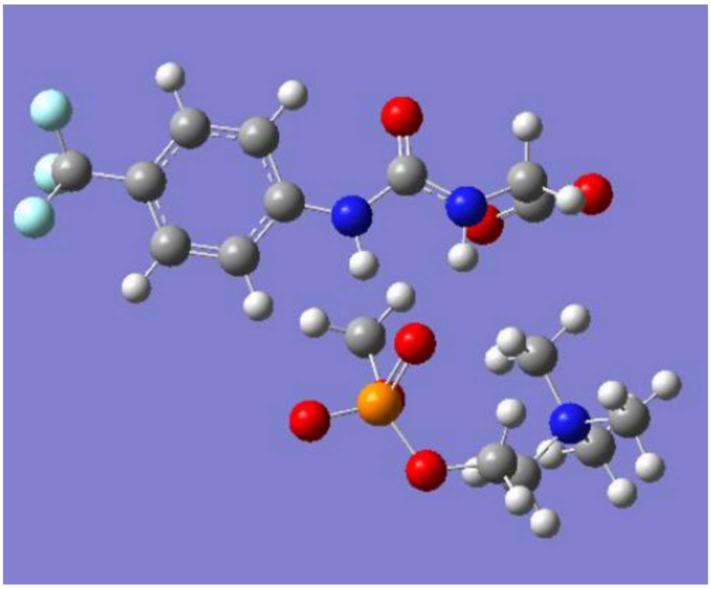

Figure S258 - Output file generated for conformation **T** (Figure S222) calculated at M06-2X/6-31G level.

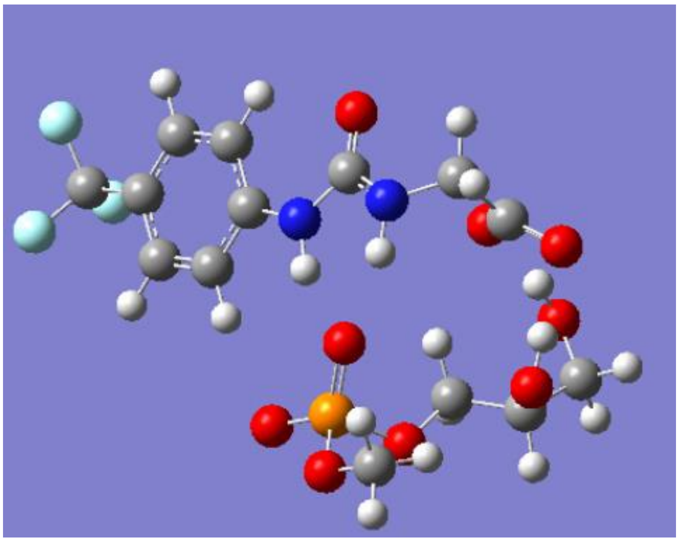

Figure S259 - Output file generated for conformation **U** (Figure S222) calculated at M06-2X/6-31G level.

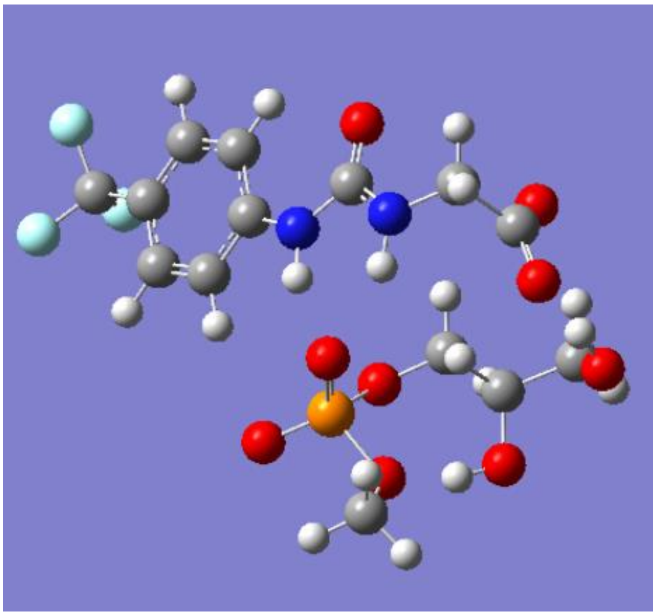

Figure S260 - Output file generated for conformation **V** (Figure S222) calculated at M06-2X/6-31G level.

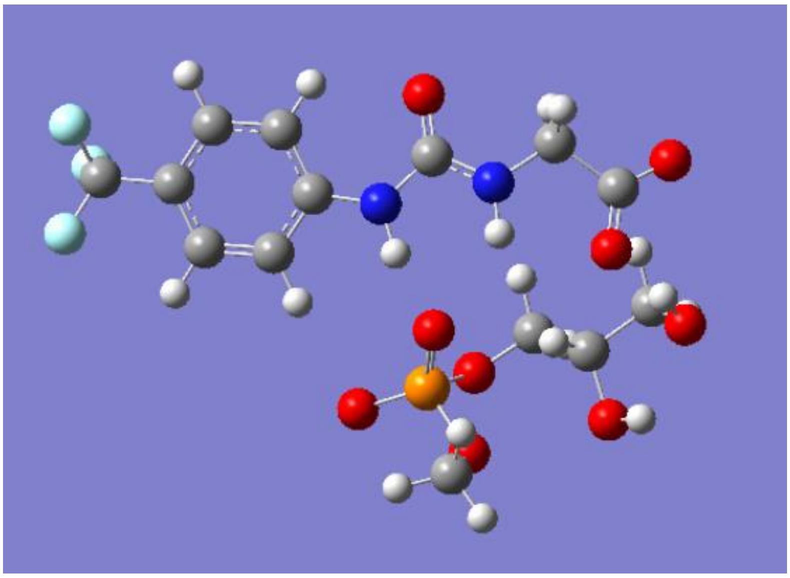

Figure S261 - Output file generated for conformation **W** (Figure S222) calculated at M06-2X/6-31G level.

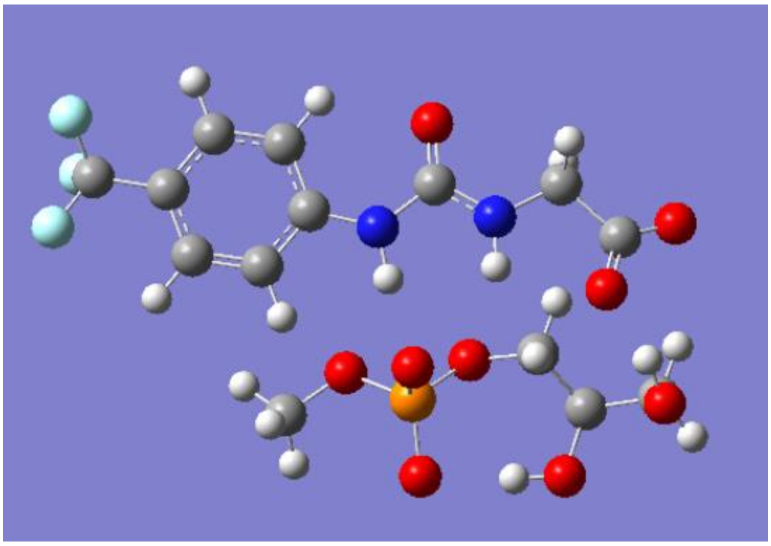

Figure S262 - Output file generated for conformation **X** (Figure S222) calculated at M06-2X/6-31G level.

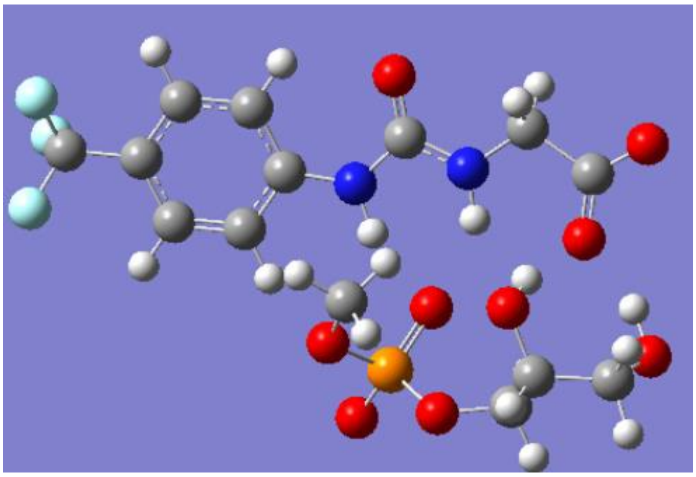

Figure S263 - Output file generated for conformation **Y** (Figure S222) calculated at M06-2X/6-31G level.

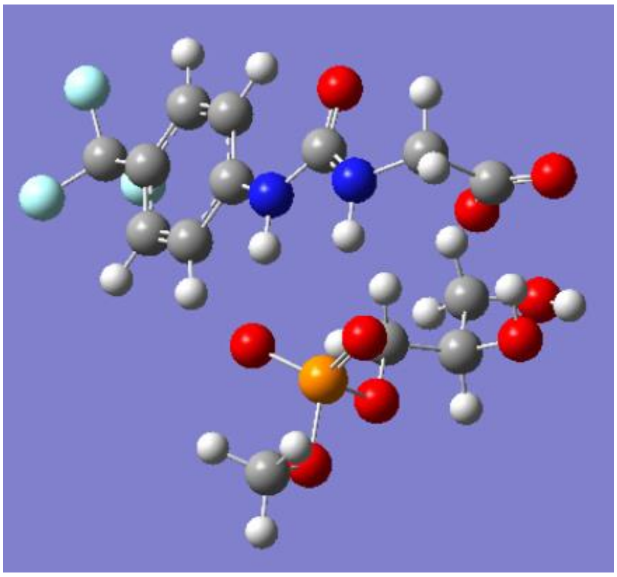

Figure S264 - Output file generated for conformation **Z** (Figure S222) calculated at M06-2X/6-31G level.

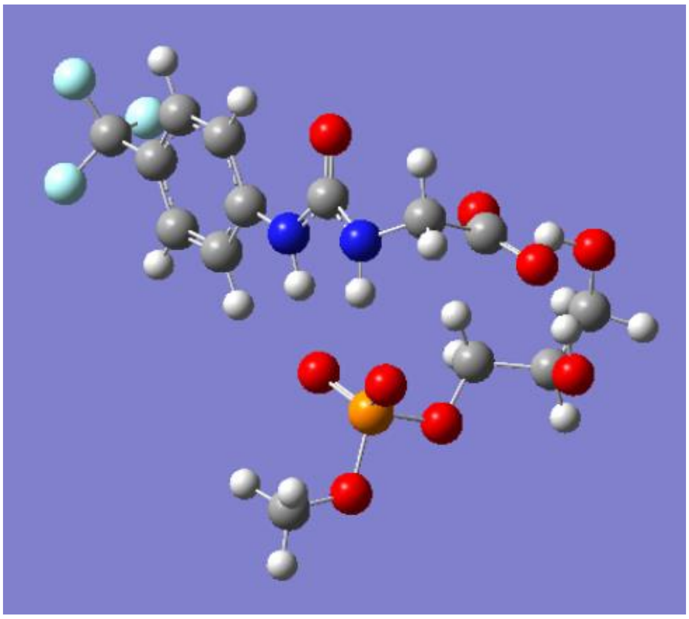

Figure S265 - Output file generated for conformation **AA** (Figure S222) calculated at M06-2X/6-31G level.

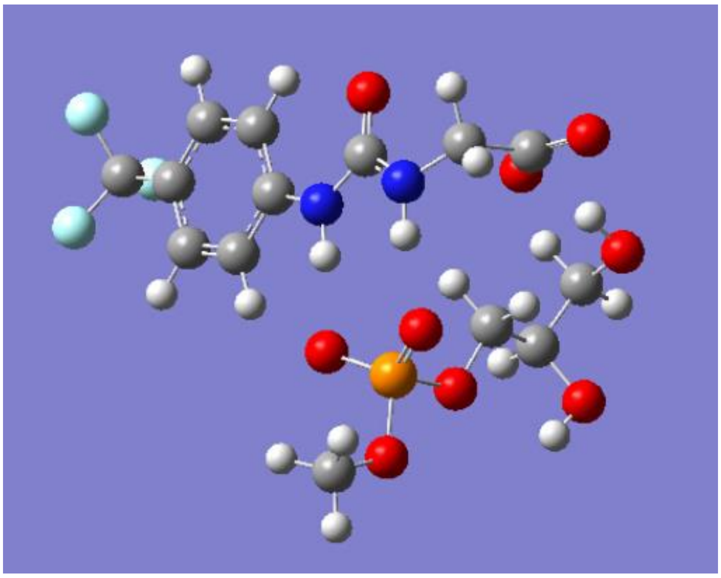

Figure S266 - Output file generated for conformation **AB** (Figure S222) calculated at M06-2X/6-31G level.

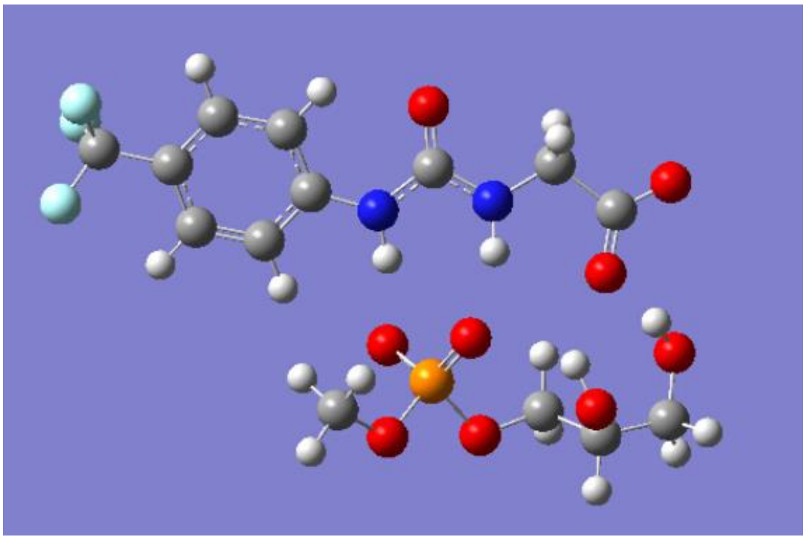

Figure S267 - Output file generated for conformation **AC** (Figure S222) calculated at M06-2X/6-31G level.

Section 16: References

1 Joos, J. P. Clare and A. P. Davis, *Angew. Chem.*, 2003, **42**, 4931–4933.

2 G. Townshend, G. S. Thompson, L. J. White, J. R. Hiscock and J. L. Ortega-Roldan, *Chem. Commun.*, 2020, **56**, 4015–4018.

3 H. Y. Carr and E. M. Purcell, *Phys. Rev.*, 1954, **94**, 630–638.

4 S. Meiboom and D. Gill, *Rev. Sci. Instrum.*, 1958, **29**, 688–691.

5 *OriginPro*, OriginLab Corporation, Northhampton, MA, USA.

6 J. R. Hiscock, G. P. Bustone, B. Wilson, K. E. Belsey and L. R. Blackholly, *Soft Matter*, 2016, **12**, 4221–4228.

7 L. J. White, S. N. Tyuleva, B. Wilson, H. J. Shepherd, K. K. L. Ng, S. J. Holder, E. R. Clark and J. R. Hiscock, *Chem-Eur. J.*, 2018, **24**, 7761–7773.

8 L. R. Blackholly, H. J. Shepherd and J. R. Hiscock, *CrystEngComm*, 2016, **18**, 7021–7028.

9 L. J. White, N. J. Wells, L. R. Blackholly, H. J. Shepherd, B. Wilson, G. P. Bustone, T. J. Runacres and J. R. Hiscock, *Chem. Sci.*, 2017, **8**, 7620–7630.

10 A. Rutkauskaite, L. J. White, K. L. F. Hilton, G. Picci, L. Croucher, C. Caltagirone and J. R. Hiscock, *Org. Biomol. Chem.*, 2020.

11 N. Allen, L. J. White, J. E. Boles, Dr. G. T. Williams, Dr. D. F. Chu, R. J. Ellaby, Dr. H. J. Shepherd, K. K. L. Ng, L. R. Blackholly, B. Wilson, Prof. D. P. Mulvihill and Dr. J. R. Hiscock, *Chemmedchem*, 2020, **15**, 2193–2193.

12 M. Walker, A. J. A. Harvey, A. Sen and C. E. H. Dessent, *J. Phys. Chem. A*, 2013, **117**, 12590–12600.
